# Different Types and Acceptability of Psychotherapies for Acute Anxiety Disorders in Children and Adolescents: A Network Meta-analysis

**DOI:** 10.1001/jamapsychiatry.2018.3070

**Published:** 2018-10-31

**Authors:** Xinyu Zhou, Yuqing Zhang, Toshiaki A. Furukawa, Pim Cuijpers, Juncai Pu, John R. Weisz, Lining Yang, Sarah E. Hetrick, Cinzia Del Giovane, David Cohen, Anthony C. James, Shuai Yuan, Craig Whittington, Xiaofeng Jiang, Teng Teng, Andrea Cipriani, Peng Xie

**Affiliations:** 1 Department of Psychiatry, The First Affiliated Hospital of Chongqing Medical University, Chongqing, China; 2 Department of Neurology, The Second Affiliated Hospital of Chongqing Medical University, Chongqing, China; 3 Department of Neurology, The First Affiliated Hospital of Chongqing Medical University, Chongqing, China; 4 Department of Health Promotion and Human Behavior, Kyoto University Graduate School of Medicine/School of Public Health, Kyoto, Japan; 5 Department of Clinical, Neuro and Developmental Psychology, Amsterdam Public Health Research Institute, Vrije Universiteit Amsterdam, Amsterdam, the Netherlands; 6 Department of Psychology, Harvard University, Cambridge, Massachusetts; 7 Department of Psychological Medicine, University of Auckland, Auckland, New Zealand; 8 Centre of Youth Mental Health, University of Melbourne, Melbourne, Australia; 9 Institute of Primary Health Care, University of Bern, Bern, Switzerland; 10 Department of Child and Adolescent Psychiatry, Hôpital Pitié–Salpétrière, Institut des Systèmes Intelligents et Robotiques, Université Pierre et Marie Curie, Paris, France; 11 Department of Psychiatry, Warneford Hospital, University of Oxford, Oxford, United Kingdom; 12 Oxford Health NHS Foundation Trust, Warneford Hospital, Oxford, United Kingdom; 13 Doctor Evidence, Santa Monica, California

## Abstract

**Importance:**

Anxiety disorders are common in children and adolescents, and uncertainty remains regarding the optimal strategy of psychotherapies in this population.

**Objective:**

To compare and rank the different types of psychotherapies and the different ways of delivering psychological treatments for anxiety disorders in children and adolescents.

**Data Sources:**

PubMed, Cochrane Central Register of Controlled Trials, EMBASE, PsycINFO, Web of Science, CINAHL (Cumulative Index to Nursing and Allied Health Literature), ProQuest Dissertations, LILACS (Literatura Latino Americana em Ciências da Saúde), international trial registers, and US Food and Drug Administration reports were searched from inception to November 30, 2017.

**Study Selection:**

Randomized clinical trials that compared any structured psychotherapy with another psychotherapy or a control condition for anxiety disorders in children and adolescents were selected.

**Data Extraction and Synthesis:**

Four researchers independently performed data extraction and quality assessment. Pairwise meta-analyses and Bayesian network meta-analysis within the random-effects model were used to synthesize data.

**Main Outcomes and Measures:**

Efficacy (change in anxiety symptoms) posttreatment and at follow-up, acceptability (all-cause discontinuation), and quality of life and functional improvement were measured. The certainty of evidence was assessed using the Grading of Recommendations Assessment, Development and Evaluation framework.

**Results:**

A total of 101 unique trials including 6625 unique participants compared 11 different psychotherapies with 4 specific control conditions. The certainty of evidence was rated as low or very low for most comparisons. For efficacy, most psychotherapies were significantly more effective than the wait list condition posttreatment (standardized mean difference [SMD], −1.43 to −0.61) and at the longest follow-up (SMD, −1.84 to −1.64). However, only group cognitive behavioral therapy (CBT) was significantly more effective than the other psychotherapies and all control conditions posttreatment. For acceptability, bibliotherapy CBT had significantly more all-cause discontinuations than some psychotherapies and control conditions (range of odds ratios, 2.48-9.32). In terms of quality of life and functional improvement, CBT (delivered in different ways) was significantly beneficial compared with psychological placebo and the wait list condition (SMDs, 0.73 to 1.99).

**Conclusions and Relevance:**

Group CBT would be the more appropriate choice of psychotherapy for anxiety disorders in children and adolescents, based on these findings. Other types of psychotherapies and different ways of delivering psychological treatment can be alternative options. Further research is needed to explore specific anxiety disorders, disorder-specific psychotherapy, and moderators of treatment effect.

**Trial Registration:**

PROSPERO Identifier: CRD42015016283

## Key Points

**Question:**

What is the best psychotherapeutic approach for anxiety disorders in children and adolescents in terms of efficacy and acceptability?

**Findings:**

This network meta-analysis included 101 unique trials with 6625 unique participants who received 11 different psychotherapies and 4 control conditions. Only group cognitive behavioral therapy was significantly more effective in reducing anxiety symptoms than other psychotherapies and all control conditions posttreatment and at short-term follow-up.

**Meaning:**

Group cognitive behavioral therapy may be the initial choice of psychotherapy for anxiety disorders in children and adolescents, after replication in future research with focus on disorder-specific psychotherapies and identification of moderators of treatment effect.

## Teaser

This network meta-analysis compares and ranks the different types of psychotherapies and the different ways of delivering psychological treatments for anxiety disorders in children and adolescents.

## Introduction

The lifetime prevalence of anxiety disorders in children and adolescents ranges from approximately 15% to 20%.^1^ Generalized anxiety disorder, social anxiety disorder, and specific phobia share common clinical features,^2,3^ often occur with depressive disorders,^4^ and had have a negative association with educational achievement, family life, and leisure activities.^5^

Psychological treatments, especially cognitive behavioral therapy (CBT), are commonly used to treat anxiety disorders in children and adolescents.^6^ Recent meta-analyses found evidence to support the effectiveness of CBT in reducing anxiety symptoms and improving function among children with or without autistic spectrum conditions, with recovery rates increased to 37% and 66% respectively, compared with 21% for a wait list control condition.^7,8^ However, other psychotherapies are also in use, such as BT without the cognitive restructuring component and bibliotherapy.^9,10^ Nevertheless, debate regarding the different components and format of psychotherapy is ongoing,^11^ for instance, whether cognitive maturity is required for successful engagement in CBT for young children and whether differences exist in efficacy between psychotherapy delivered individually or in a group setting.^12^ These issues lead to uncertainty in the decision making for health care professionals and patients. However, previous pairwise meta-analyses could not answer these clinical questions,^13,14^ because few trials have directly compared different types of psychotherapies.^15^ Network meta-analysis allows for a better data synthesis because indirect comparisons can be made. Using network meta-analysis, we aimed to comprehensively compare and rank psychological interventions for the acute treatment of anxiety disorders in children and adolescents.

## Methods

### Search Strategy and Selection Criteria

We performed a comprehensive literature search for published and unpublished randomized clinical trials in PubMed, Cochrane Central Register of Controlled Trials, EMBASE, PsycINFO, Web of Science, CINAHL (Cumulative Index to Nursing and Allied Health Literature), ProQuest Dissertations, LILACS (Literatura Latino Americana em Ciências da Saúde), international trial registers, and US Food and Drug Administration reports from inception until November 30, 2017. Eligible studies included any structured psychotherapy for the acute treatment of children and adolescents (18 years or younger when enrolled in the trials) with a primary diagnosis of anxiety disorders according to standardized diagnostic criteria assessed by trained staff via clinical interview.^16^ A psychotherapy was considered structured when it was accompanied by an explicit manual for therapists to follow and/or laid out in a manual for the participants. No restrictions on language were used. Study authors were contacted to supplement incomplete reports of the original papers or provide data for unpublished studies.

According to *DSM-5*, anxiety disorders include generalized anxiety disorder, social anxiety disorder, specific phobia, panic disorder, agoraphobia, separation anxiety disorder, and selective mutism but not posttraumatic stress disorder or obsessive-compulsive disorder. Trials of combination therapies, treatment-resistant anxiety disorder, a treatment duration of less than 6 weeks, or an overall sample size of less than 10 patients were exclusion criteria.

Psychotherapies can be delivered in different modalities (face-to-face or Internet-assisted), different conditions (childhood psychotherapy, parental involvement therapy, or parent-only therapy), and different formats (group, individual, or both). Because different treatment effects may occur across different types of treatment and different delivery formats of psychotherapies, we a priori decided to consider them as independent nodes in the network meta-analysis. In addition, we defined parental involvement in therapy as including parent attendance in at least 40% of total sessions of children and parents and at least 40% involvement of each session.^16^ The control conditions included no treatment, psychological placebo, treatment as usual, and the wait list condition, which were viewed as independent nodes in this study. Psychological placebo was defined as a control condition that was regarded as inactive by the researchers but was presented to the participants as being an active therapy, whereas treatment as usual included any nonstructured psychotherapy, which may have some treatment effects. Further descriptions of the included psychotherapeutic interventions and control conditions are shown in eMethods 3 in the Supplement.

### Data Extraction and Quality Assessment

Four researchers (Y.Z., J.P., L.Y., and S.Y.) independently screened eligible trials, extracted the relevant information, and assessed risk of bias according to the Cochrane risk of bias tool (κ range for interrater reliability, 0.88-0.92).^17^ Any discrepancies of data extraction and risk of bias assessment were resolved by consensus and arbitration by a panel of other investigators within the review team (T.A.F., A.C., and P.X.).

### Outcomes

We assessed efficacy posttreatment and at follow-up as the mean change scores in anxiety symptoms from baseline to end point and from baseline to the end of follow-up (≤12 months). Anxiety symptoms were measured using various psychometrically continuous scales, such as the Revised Children’s Manifest Anxiety Scale and Spence Children’s Anxiety Scale.^18,19^ For the same scale with different informants, we prioritized self-rated scales, then the parent report, teacher report, and health care professional’s report.^15^ We also assessed acceptability, measured as the proportion of patients who discontinued treatment for any reason during the acute phase of treatment, and quality of life and functional improvement (QOL/functioning), measured as mean change scores from baseline to end point. When a study used 2 or more scales to measure a similar construct, we chose the single best available outcome measure according to a hierarchy based on psychometric properties and appropriateness for use with children and adolescents (eMethods 4 in the Supplement).

### Statistical Analysis

Details of the applied statistical approaches are provided in eMethods 1 through 5 in the Supplement. First, the pairwise meta-analysis was conducted using the random-effects model with Stata software (version 13.0; StataCorp). Odds ratios were used for dichotomous outcomes, and standardized mean differences (SMDs) were used for continuous outcomes, with 95% CIs. For studies with multiple intervention groups, we combined groups to create a single pairwise comparison.^17^ Statistical heterogeneity was assessed using the *I^2^* statistic and the *P* value of the *Q* statistic, with *P* < .05 indicating significance. *P* values were 2 sided. Potential publication bias or small-study effect was detected using the Egger test if at least 10 studies were available.^20^

The network meta-analysis was conducted based on a Bayesian framework random-effects model^21^ with WinBUGS software (version 1.4.3; MRC Biostatistics Unit). For each comparison, a mean effect estimate (SMD or odds ratio) along with its 95% credible interval (CrI) were calculated using the Markov chains Monte Carlo method.^22^ Two Markov chains were run simultaneously with different arbitrarily chosen initial values. Convergence was assessed by running 2 chains, inspecting the sampling trace plots and the Brooks-Gelman-Rubin statistic. Model fit was assessed using deviance information criterion and mean posterior deviance of the network model. A common heterogeneity parameter was assumed for all comparisons, and we assessed the global heterogeneity using the *I*^2^ statistic with the gemtc R package (version 0.8-2; CRAN). The estimated common τ^2^ values were compared with the empirical ones for continuous and dichotomous outcomes. The estimated distribution for continuous outcomes (mental health, nonpharmacologic) was 0.058,^23^ and the estimated distribution for dichotomous outcomes (subjective, nonpharmacologic) was 0.13.^24^ We used the design-by-treatment inconsistency model to evaluate the global inconsistency, the loop-specific approach to evaluate the local inconsistency, and the node-splitting approach to calculate the inconsistency for each comparison.^25^ We estimated the ranking probabilities for all interventions and reported the surface under the cumulative ranking curves.^26^ A Hasse diagram was drawn using R (version 3.2.2; CRAN) with the netmeta package to integrate rankings from different outcomes.^27^ Comparison-adjusted funnel plots for the network meta-analysis were plotted by comparing all active psychotherapies against all control conditions (no treatment, psychological placebo, treatment as usual, or wait list) to detect the presence of any dominant publication bias.^28^ These analyses were performed with Stata (version 13.0) and R (version 3.2.2) software. The certainty of the evidence for efficacy outcomes was assessed using the Grading of Recommendations Assessment, Development and Evaluation (GRADE) framework across the following 5 domains: study limitations, imprecision, heterogeneity and inconsistency, indirectness, and publication bias.^29^

The following subgroup analyses (considering publication year, sample size, sex ratio, mean age, treatment duration, number of sessions, and source of outcome information) and sensitivity analyses (excluding studies with a high risk of bias or trials with maternal anxiety disorder) were performed. We also conducted network meta-regression analyses of all variables in subgroup analyses by calculating the Somer D value (a correlation coefficient for a dichotomous and an ordinal variable).^30^ The full data set is available online in Mendeley (doi:10.17632/7t7rfrb272.2).

## Results

**Figure 1.**
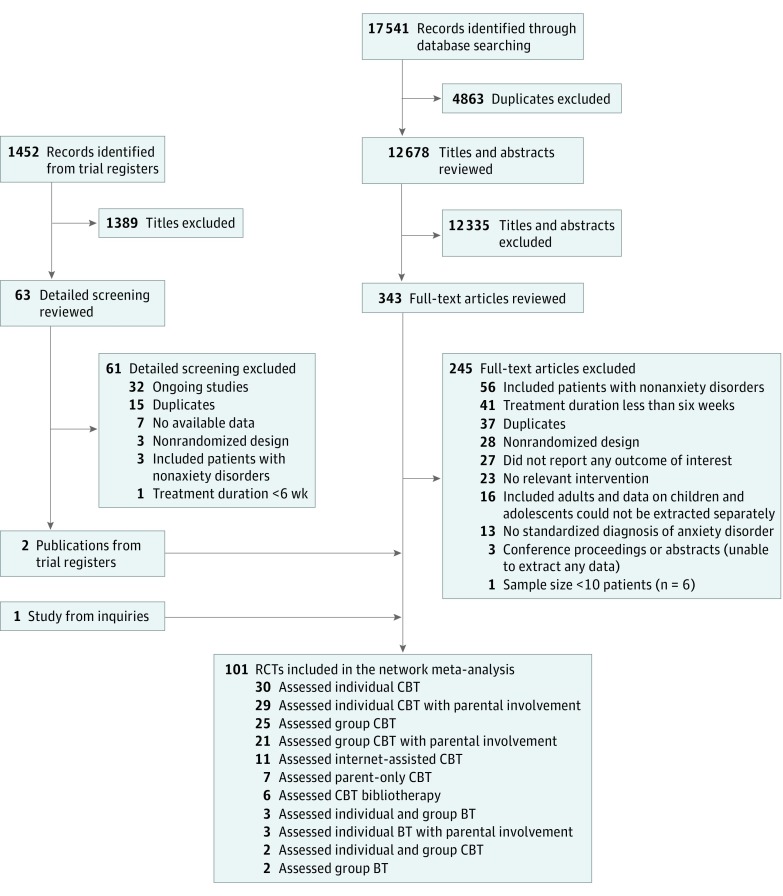
Flowchart of Study Selection Some studies assessed more than 1 type of psychotherapy. BT indicates behavioral therapy without cognitive restructuring; CBT, cognitive BT; RCT, randomized clinical trial.

Figure 1 shows the process of study selection. In total, we included 101 unique randomized clinical trials involving 6625 unique patients. A complete list of the included trials appears in eMethods 6 in the Supplement; the list of full-text excluded studies, online in Mendeley (doi:10.17632/bkr2gtjmyf.1). Eleven different psychotherapies, including group BT, individual and group BT, individual BT with parental involvement, group CBT, group CBT with parental involvement, individual CBT, individual and group CBT, individual CBT with parental involvement, Internet-assisted CBT, parent-only CBT, and bibliotherapy CBT, and the 4 control conditions (wait list, psychological placebo, no treatment, and treatment as usual) were assessed. The hallmark distinction between BT and CBT was the inclusion of cognitive restructuring in the latter.

The clinical and methodologic characteristics of included trials are shown in eTable 1 in the Supplement. The studies were published from 1994 and 2017 and were conducted in 14 countries. Seventy-five studies (74.3%) included patients with mixed anxiety disorders. The median study sample size was 54 patients (range, 11-267 patients). Approximately half of total participants (3350 [50.6%]) were girls, and the median proportion of female participants was 52% (range, 8%-100%). Twenty trials enrolled only children; 49, only adolescents; and the remainder, children and adolescents. The mean (SD) age of participants was 10.8 (3.0) years. The median duration of the acute treatment was 12 weeks (range, 6-32 weeks), the median number of sessions was 12 (range, 6-32), and the median number of sessions with family involvement was 4 (range, 0-20). The median duration of the longest follow-up was 6 months (range, 1-12 months). For the study quality, 72 trials (71.3%) were rated as at moderate risk of bias; 21 (20.8%), at high risk of bias; and 8 (7.9%), at low risk of bias (eMethods 7 in the Supplement).

**Figure 2.**
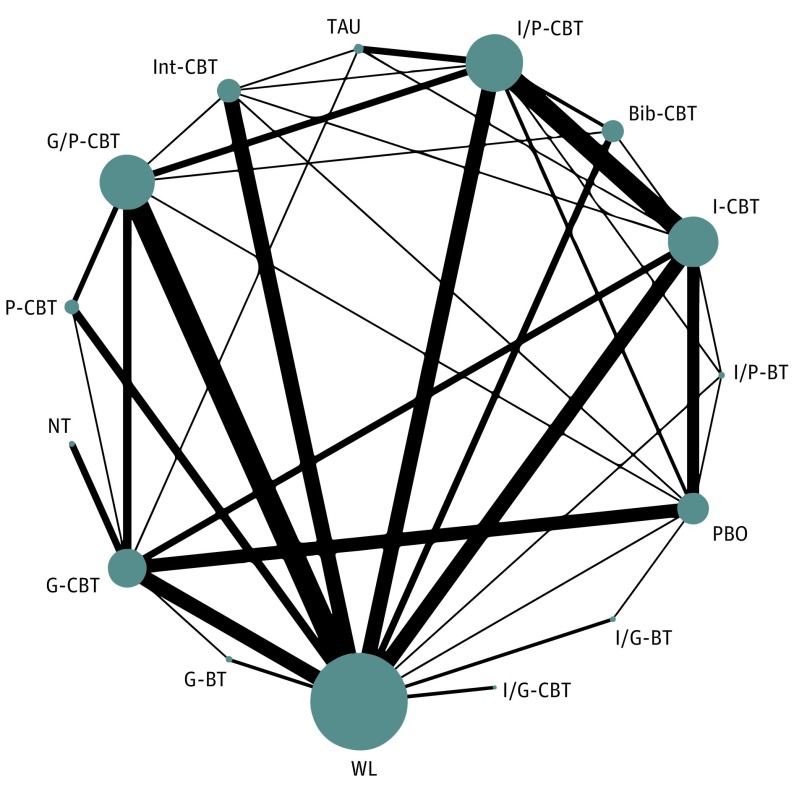
Network of Eligible Comparisons for Efficacy Posttreatment The width of the lines is proportional to the number of trials comparing every pair of treatments, and the size of every node is proportional to the number of randomized participants. Bib-CBT indicates bibliotherapy cognitive behavioral therapy; G-BT, group BT without cognitive restructuring; G-CBT, group CBT; G/P-CBT, group CBT with parental involvement; I-CBT, individual CBT; I/G-BT, individual and group BT; I/G-CBT, individual and group CBT; Int-CBT, Internet-assisted CBT; I/P-BT, individual BT with parental involvement; I/P-CBT, individual CBT with parental involvement; NT, no treatment; PBO, psychological placebo; P-CBT, parent-only CBT; TAU, treatment as usual; and WL, wait list.

The network of treatment comparisons for efficacy posttreatment is shown in Figure 2. Networks for other outcomes are displayed in eFigure 1 in the Supplement.

### Pairwise Meta-analysis

For efficacy, group CBT, individual CBT, and parental involvement CBT were statistically significantly more efficacious than the wait list condition posttreatment and at follow-up (eResults in the Supplement). For acceptability, bibliotherapy CBT was less acceptable than group CBT with parental involvement and the wait list condition. For QOL/functioning, group CBT with parental involvement, individual CBT, individual and group BT, Internet-assisted CBT, and parent-only CBT were significantly more beneficial than the wait list condition or psychological placebo (eFigure 2 in the Supplement).

### Network Meta-analysis

**Figure 3.**
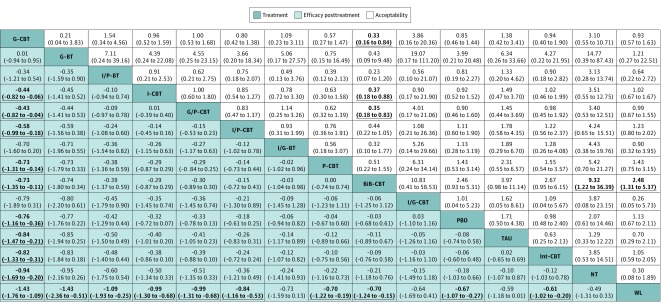
Network Meta-analysis of Efficacy and Acceptability Posttreatment Treatments are reported in order of efficacy posttreatment with ranking according to surface under the cumulative ranking curves. Comparisons between treatments should be read from left to right, and the estimate is in the cell in common between the column-defining treatment and the row-defining treatment. Efficacy posttreatment values are given as mean overall change in symptoms (standardized mean differences [SMDs]); SMDs of less than 0 favor the column-defining treatment. Acceptability values are given as all-cause discontinuation (odds ratios [ORs]); an OR of less than 1.00 favors the row-defining treatment. Data in parentheses represent 95% credible intervals. To obtain ORs for comparisons in the opposing direction, reciprocals should be taken. To obtain SMDs for comparisons in the opposite direction, negative values should be converted into positive values, and vice versa. Significant results are set in boldface. Bib-CBT indicates bibliotherapy cognitive behavioral therapy; G-BT, group BT without cognitive restructuring; G-CBT, group CBT; G/P-CBT, group CBT with parental involvement; I-CBT, individual CBT; I/G-BT, individual and group BT; I/G-CBT, individual and group CBT; Int-CBT, Internet-assisted CBT; I/P-BT, individual BT with parental involvement; I/P-CBT, individual CBT with parental involvement; NT, no treatment; PBO, psychological placebo; P-CBT, parent-only CBT; TAU, treatment as usual; and WL, wait list.

**Figure 4.**
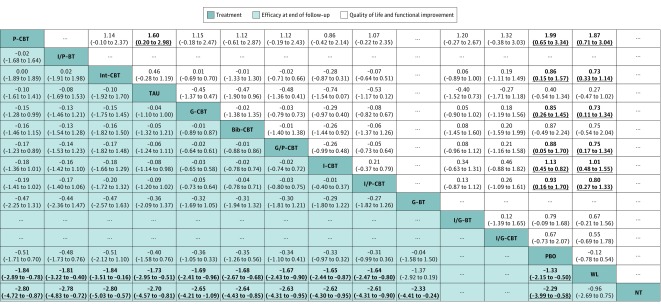
Network Meta-analysis of Efficacy at End of Follow-Up and Quality of Life and Functional Improvement Treatments are reported in order of acceptability ranking according to surface under the cumulative ranking curves. Comparisons between treatments should be read from left to right, and the estimate is in the cell in common between the column-defining treatment and the row-defining treatment. Efficacy at end of follow-up values are given as mean overall change in symptoms (standardized mean differences [SMDs]); SMDs of less than 0 favor the column-defining treatment. For quality of life and functional improvement at post-treatment, SMDs more than 0 favor the row-defining treatment. Data in parentheses represent 95% credible intervals. To obtain SMDs for comparisons in the opposite direction, negative values should be converted into positive values, and vice versa. Significant results are set in boldface. Bib-CBT indicates bibliotherapy cognitive behavioral therapy; ellipsis, no data about efficacy; G-BT, group BT without cognitive restructuring; G-CBT, group CBT; G/P-CBT, group CBT with parental involvement; I-CBT, individual CBT; I/G-BT, individual and group BT; I/G-CBT, individual and group CBT; Int-CBT, Internet-assisted CBT; I/P-BT, individual BT with parental involvement; I/P-CBT, individual CBT with parental involvement; NT, no treatment; PBO, psychological placebo; P-CBT, parent-only CBT; TAU, treatment as usual; and WL, wait list.

In terms of efficacy posttreatment, all psychotherapies were more beneficial than the wait list control condition, but only group CBT was significantly more effective than all neutral control conditions (SMD range, −1.43 to −0.76) and most other psychotherapies (SMD range, −0.82 to −0.43) (Figure 3). In terms of efficacy at the end of follow-up, almost all investigated psychotherapies were significantly more effective than the wait list condition and no treatment (SMD range, −2.80 to −1.64) (Figure 4), but only group CBT was significantly more effective than group CBT with parental involvement and all control conditions at short-term follow-up (SMD range, −0.43 to −0.82) (Figure 3). Psychological placebo was significantly more effective than the wait list condition in efficacy posttreatment and at follow-up. In terms of acceptability, only bibliotherapy CBT had significantly more all-cause discontinuations than some other psychotherapies and control conditions (range of odds ratios, 2.48-9.32) (Figure 3). In terms of QOL/functioning, almost all CBT, but not BT, showed significantly more benefit compared with psychological placebo and the wait list condition (SMD range, 0.73-1.99) (Figure 4).

### Heterogeneity and Sensitivity Analyses

The common heterogeneity SD was 0.65 (95% CrI, 0.54-0.77) for efficacy posttreatment, 0.63 (95% CrI, 0.43-0.89) for efficacy at follow-up, 0.49 (95% CrI, 0.20-0.75) for acceptability, and 0.51 (95% CrI, 0.33-0.76) for QOL/functioning. These heterogeneity SDs are relatively high but are within the empirically estimated distributions. The test of global inconsistency did not show a significant difference between the consistency and inconsistency models for efficacy posttreatment (*P* = .50), but a significant difference was found for efficacy at follow-up (*P* < .001) (eFigure 3 in the Supplement). Tests of local inconsistency showed small percentages of inconsistent loops for the efficacy posttreatment within the empirically expected range (6 of 52 comparison loops) but not for efficacy at follow-up (6 of 16 comparison loops) (eFigure 4 in the Supplement). The test of inconsistency from the node-splitting model showed significant differences between some comparisons in efficacy posttreatment (3 of 39) and at follow-up (4 of 23) (eFigure 4 in the Supplement). Egger tests for the comparison-adjusted funnel plot suggested potential publication bias or small-study effect for efficacy posttreatment and at follow-up (eFigure 5 in the Supplement). The ranking of treatments is presented in eFigure 6 in the Supplement. In terms of efficacy posttreatment, the most effective treatments were group CBT (93.4%) and group BT (86.1%), whereas the least effective was the wait list condition (2.4%). In terms of efficacy at follow-up, the most effective treatments were parent-only CBT (67.9%), individual BT with parental involvement (66.1%), and Internet-assisted CBT (65.6%), whereas the least effective was no treatment (1.5%). The full details of the subgroup and sensitivity analyses and the network meta-regression are reported in eFigures 7 and 8 and eTable 2 in the Supplement. According to the GRADE framework, the certainty of the evidence for efficacy was low for most comparisons and very low for some comparisons (eFigure 9 in the Supplement).

## Discussion

This network meta-analysis presents an up-to-date and comprehensive synthesis of data for structured psychotherapy for children and adolescents with acute anxiety disorders. We found that CBT and BT were significantly more beneficial than the wait list condition in reducing anxiety symptoms posttreatment and at follow-up. However, only group CBT was significantly more effective than some other psychotherapies posttreatment and at short-term follow-up. Overall, the clinical interpretation of these findings is limited, not only by the small number of trials in each node, but also by the poor methodology, risk of bias of individual studies, large inconsistency of the network, and potential selective reporting.

The magnitude of the effect of group CBT over active interventions involving human contact, such as individual CBT or parent-only CBT, was in the range of 0.4 to 0.7 in terms of SMD, and that over interventions without human contact, such as Internet-assisted CBT or bibliotherapy CBT, was even greater, with SMDs of 0.7 or 0.8.^31^ When converted into numbers needed to treat, the efficacy of group CBT over other active human interventions may correspond with numbers needed to treat of approximately 5 and over interventions without human contact may correspond with numbers needed to treat of approximately 3.^32^

The delivery formats of psychotherapy for anxiety disorders in children vs adolescents in still under wide debate.^13,14^ In our subgroup analyses (eTable 2 in the Supplement), we found different point estimates for group CBT for adolescents (mean age, ≥13 years; SMD, −0.82) vs younger patients (mean age, <13 years; SMD, −0.50); however, the corresponding test for subgroup difference was nonsignificant (*P* = .45). Previous studies^33,34^ suggested that a certain level of cognitive maturity is required for successful engagement in CBT, which children may not yet have acquired. For instance, the only 2 trials involving a group BT arm^35,36^ included children aged 10 to 14 years, showing that group BT may be especially helpful for this age range. However, whether age is associated with treatment effect remains unclear, because other factors, such as depression or parental symptoms, may also interact with age.^35,36^ The results of our analysis suggest that psychotherapy delivered in a group format may generally result in better outcomes than when delivered individually, which, even if not necessarily true for all the patients, may be attributed to the additional exposure of social stimuli and interaction in the group format and thus increasing the efficacy of psychotherapy.^37^ These results are not replicated in adults, especially for depression.^38,39^ Future work should properly examine whether and how the group format may be of particular benefit for younger people with anxiety disorders.

We found significant inconsistencies in several loops involving group CBT, and its efficacy might be overestimated by publication bias. Health care professionals should interpret the findings about group interventions being better than individual interventions with caution. Moreover, in agreement with previous meta-analyses,^40,41^ we also found that some self-help psychotherapies (such as Internet-assisted CBT and bibliotherapy CBT) are effective in reducing anxiety symptoms when compared with the wait list condition and can be useful clinical tools, especially in consideration of accessibility and cost-effectiveness issues. However, self-help psychotherapies may be associated with higher rates of treatment discontinuation and may only apply to people with higher literacy.

This network meta-analysis also showed that children and adolescents may benefit from psychotherapy with the involvement of parents, but previous analyses did not suggest that the role of the involvement of parents in psychotherapy is more beneficial than psychotherapy alone.^42,43^ With the exception of bibliotherapy CBT, no significant differences were detected among other psychotherapies in the outcome of all-cause discontinuation.

In our analysis, we have shown that CBT, but not BT, may have a positive association with various domains of a patient’s life, such as mental functioning, social and study-related relationships, level of discomfort, and engagement in everyday activities.^44^ One theory that may explain the difference is that the cognitive restructuring included in CBT compared with BT interventions enables a young person to more readily accept the emotions associated with an anxiety disorder.^45^

### Limitations

Our study has many limitations. First, because the numbers of trials for several nodes in this network meta-analysis were very small, the statistical power for some comparisons was limited, and we did not have enough trials to analyze specific anxiety disorders. Second, the certainty of evidence was rated as low or very low, and although the global test of inconsistency was not significant for efficacy posttreatment, the test was significant for efficacy at follow-up. Third, according to our protocol, we excluded participants with subsyndromal anxiety symptoms or treatment-resistant anxiety disorder. This exclusion was aimed at preserving the transitivity across the network but may limit the generalizability of results from this study because such patients represent a considerable proportion of the people seen in real-world clinical settings. Finally, without access to individual patient-level data, we cannot analyze the moderating effect of some participant characteristics (eg, ethnicity, baseline anxiety symptom severity, and comorbid diagnoses), which may explain the heterogeneity and inconsistency in the network. Having access to individual patient-level data will also contribute to a precision medicine approach that will enable researchers and health care professionals to individualize treatment indications for children and adolescents with anxiety disorders.^46^

## Conclusions

This network meta-analysis suggests that group CBT might be considered as the initial choice of psychotherapy for anxiety disorders in children and adolescents; however, more research is needed to confirm such conclusions. Health care professionals, patients, and families should carefully interpret these findings, bearing in mind the limited amount of information and the low quality of available evidence. The use of a group setting may play a role in moderating the effect of psychological treatments: group CBT appeared to produce more robust effects in adolescents and group BT, in children. Only CBT may have a significant benefit in improving QOL/functioning. The use of a wait list control condition may inflate the apparent treatment effect of psychotherapies, whereas psychological placebo is likely to provide a more robust comparison in psychotherapy trials.

## Notes

Supplement. **eMethods 1. **Published Protocol and Changes Made to the Protocol **eMethods 2.** Search Strategy and Results **eMethods 3.** Description of Psychotherapeutic Interventions and Control Conditions **eMethods 4.** Hierarchy of Anxiety Scales **eMethods 5.** Network Meta-analysis Model **eMethods 6.** References for Included Trials **eMethods 7.** Risk of Bias Assessment **eFigure 1.** Network Plot for Each Outcome **eFigure 2.** The Forest Plots of Network Meta-analysis Results for Efficacy Posttreatment and End of Follow-up **eFigure 3.** The Results of Network Meta-analysis for Secondary Outcomes **eFigure 4.** Assessment of Inconsistency Results for Each Outcome: Global, Local, and From the Node-Splitting Model **eFigure 5.** Comparison-Adjusted Funnel Plot and Egger Test for Each Outcome From the Network Meta-analysis **eFigure 6.** Treatment Ranking, SUCRA Plot, and Hasse Diagrams **eFigure 7.** Sensitivity Network Meta-analyses **eFigure 8.** Contribution Matrix and Contributions Summary of Risk of Bias Assessments for Each Outcome **eFigure 9.** Evaluation of the Quality of Evidence Using GRADE Framework for Primary Outcomes **eResults.** Results from Pairwise Meta-analysis for Each Outcome: Numbers, Estimates, and Heterogeneity **eTable 1.** Subgroup Network Meta-analyses and Network Metaregression for Efficacy Outcomes **eTable 2.** Clinical and Methodological Characteristics of Included Studies
