# Supplementary material for: Different Types and Acceptability of Psychotherapies for Acute Anxiety Disorders in Children and Adolescents: A Network Meta-analysis
Source: JAMA Psychiatry. 2018 Oct 31;76(1):41–50. doi: 10.1001/jamapsychiatry.2018.3070 (PMC6583467; doi:10.1001/jamapsychiatry.2018.3070)
Supplement: Supplement. — eMethods 1. Published Protocol and Changes Made to the Protocol eMethods 2. Search Strategy and Results eMethods 3. Description of Psychotherapeutic Interventions and Control Conditions eMethods 4. Hierarchy of Anxiety Scales eMethods 5. Network Meta-analysis Model eMethods 6. References for Included Trials eMethods 7. Risk of Bias Assessment eFigure 1. Network Plot for Each Outcome eFigure 2. The Forest Plots of Network Meta-analysis Results for Efficacy Posttreatment and End of Follow-up eFigure 3. The Results of Network Meta-analysis for Secondary Outcomes eFigure 4. Assessment of Inconsistency Results for Each Outcome: Global, Local, and From the Node-Splitting Model eFigure 5. Comparison-Adjusted Funnel Plot and Egger Test for Each Outcome From the Network Meta-analysis eFigure 6. Treatment Ranking, SUCRA Plot, and Hasse Diagrams eFigure 7. Sensitivity Network Meta-analyses eFigure 8. Contribution Matrix and Contributions Summary of Risk of Bias Assessments for Each Outcome eFigure 9. Evaluation of the Quality of Evidence Using GRADE Framework for Primary Outcomes eResults. Results from Pairwise Meta-analysis for Each Outcome: Numbers, Estimates, and Heterogeneity eTable 1. Subgroup Network Meta-analyses and Network Metaregression for Efficacy Outcomes eTable 2. Clinical and Methodological Characteristics of Included Studies [file jamapsychiatry-76-41-s001.pdf]

Supplementary Online Content

Zhou X, Zhang Y, Furukawa TA, et al. Different types and acceptability of psychotherapies for acute anxiety disorders in children and adolescents: a network meta-analysis. *JAMA Psychiatry*. Published online October 31, 2018.
doi:10.1001/jamapsychiatry.2018.3070

eMethods 1. Published Protocol and Changes Made to the Protocol
eMethods 2. Search Strategy and Results
eMethods 3. Description of Psychotherapeutic Interventions and Control Conditions
eMethods 4. Hierarchy of Anxiety Scales
eMethods 5. Network Meta-analysis Model
eMethods 6. References for Included Trials
eMethods 7. Risk of Bias Assessment
eFigure 1. Network Plot for Each Outcome
eFigure 2. The Forest Plots of Network Meta-analysis Results for Efficacy Post Treatment and End of Follow-up
eFigure 3. The Results of Network Meta-analysis for Secondary Outcomes
eFigure 4. Assessment of Inconsistency Results for Each Outcome: Global, Local, and From the Node-Splitting Model
eFigure 5. Comparison-Adjusted Funnel Plot and Egger Test for Each Outcome From the Network Meta-analysis
eFigure 6. Treatment Ranking, SUCRA Plot, and Hasse Diagrams
eFigure 7. Sensitivity Network Meta-analyses
eFigure 8. Contribution Matrix and Contributions Summary of Risk of Bias Assessments for Each Outcome
eFigure 9. Evaluation of the Quality of Evidence Using GRADE Framework for Primary Outcomes
eResults. Results from Pairwise Meta-analysis for Each Outcome: Numbers, Estimates, and Heterogeneity
eTable 1. Clinical and Methodological Characteristics of Included Studies
eTable 2. Subgroup Network Meta-analyses and Network Metaregression for Efficacy Outcomes

This supplementary material has been provided by the authors to give readers additional information about their work.

© 2018 Zhou X et al. *JAMA Psychiatry*.

eMethods 1. Published Protocol and Changes Made to the Protocol

The protocol has been registered in PROSPERO (No. CRD42015016283) and published in BMJ Open –available at http://bmjopen.bmj.com/content/5/10/e008572.full.pdf+html

Here below some changes and clarifications to the published protocol:

1. In order to obtain more exact results from databases, we adjusted some of the text words in search strategy. For example, we added the words “phobias”, “CBT”, “systematic desensitization”, “eye movement desensitization and reprocessing”, “EMDR”, “self-talk”, “self talk”, “emotive imagery”, “contingency management”, “preschool, and “pre-school”. Besides, we also searched additional sources of clinical trials, including Trial Register (Netherlands) and ISRCTN registry (UN).

2. We added the assessment of the quality of evidence of network estimates for primary outcomes by using the GRADE framework, which characterizes the quality of a body of evidence on the basis of the study limitations, imprecision, inconsistency, indirectness, and publication bias.

3. We had planned to conduct subgroup analyses, sensitivity analyses and meta-regressions. While given the limitation of the number of trials in some subgroups, we adjusted some analyses with changing the cut-off point of duration of psychotherapy in subgroups (short-term treatment of 10 or fewer weeks vs. long-term treatment of more than 10 weeks). In addition, we added to perform two sensitivity network meta-analyses for primary outcomes by omitting the trial with maternal anxiety disorder, and omitting the trial with rated as high risk studies. Since most of trials recuited patients with comorbid general psychiatric disorders, we did not perform the subgroup analyses and meta-regression analyses (with or without comorbid general psychiatric disorders).

© 2018 Zhou X et al. JAMA Psychiatry.

eMethods 2. Search Strategy and Results

Number of citations by each database and trial register searched*

Databases and Trial registers:	Citations
**Databases:**	
PubMed	2302
Cochrane	5228
Embase and PsycINFO	2812
Web of Science	4054
CINAHL	1204
ProQuest Dissertations	1185
LILACS	756
**Total (databases)**	**17541**
**Trial registers:**	
Australian (ANZCTR)	62
China (ChiCTR)	27
USA (ClinicalTrials.gov)	569
Netherlands (Trial Register)	76
UN (ISRCTN)	33
World Health Organization (ICTRP)	442
USA Food and Drug Administration (FDA)	243
**Total (trial registers)**	**1452**

Full search strategy for each database

PubMed

#1 "Anxiety Disorders"[Mesh]

#2 anxiety[Title/Abstract] OR anxious[Title/Abstract] OR phobic[Title/Abstract] OR fear[Title/Abstract] OR fears[Title/Abstract] OR phobia[Title/Abstract] OR phobias[Title/Abstract] OR panic disorder*[Title/Abstract] OR overanxious disorder*[Title/Abstract] OR avoidant disorder*[Title/Abstract] OR agoraphobia[Title/Abstract] OR selective mutism[Title/Abstract] OR panic attack specifier[Title/Abstract] OR combat disorder*[Title/Abstract] OR mixed disorder*[Title/Abstract] OR neurosis[Title/Abstract] OR neuroses[Title/Abstract] OR neurotic[Title/Abstract] OR school refusal[Title/Abstract]

#3 #1 or #2

#4 "Psychotherapy"[Mesh]

#5 psychother*[Title/Abstract] OR behavio*[Title/Abstract] OR cogniti*[Title/Abstract] OR

© 2018 Zhou X et al. JAMA Psychiatry.

CBT[Title/Abstract] OR interpersonal[Title/Abstract] OR relaxation[Title/Abstract] OR bibliotherap*[Title/Abstract] OR counsel*[Title/Abstract] OR “family treatment”[Title/Abstract] OR supportive[Title/Abstract] OR problem-solving[Title/Abstract] OR “problem solving”[Title/Abstract] OR psychodynamic[Title/Abstract] OR exposure[Title/Abstract] OR modeling[Title/Abstract] OR “reinforced practice”[Title/Abstract] OR “systematic desensitization”[Title/Abstract] OR “eye movement desensitization and reprocessing”[Title/Abstract] OR EMDR[Title/Abstract] OR self-talk[Title/Abstract] OR “self talk”[Title/Abstract] OR “emotive imagery”[Title/Abstract] OR “contingency management”[Title/Abstract] OR therapy[Title/Abstract]

#6 #4 or #5

#7 (adolesc*[Title/Abstract] OR child*[Title/Abstract] OR boy*[Title/Abstract] OR girl*[Title/Abstract] OR juvenil*[Title/Abstract] OR minors[Title/Abstract] OR paediatri*[Title/Abstract] OR pediatri*[Title/Abstract] OR pubescen*[Title/Abstract] OR school*[Title/Abstract] OR student*[Title/Abstract] OR teen*[Title/Abstract] OR young[Title/Abstract] OR youth*[Title/Abstract] OR class*[Title/Abstract] OR preschool[Title/Abstract] OR pre-school[Title/Abstract])

#8 "Child"[Majr]) OR "Adolescent"[Majr]

#9 #7 OR #8

#10 #3 AND #6 AND #9

#11 Filters: Clinical Trial

Cochrane

#1 MeSH descriptor: [Anxiety Disorders] explode all trees

#2 anxiety or anxious or phobic or fear or fears or phobia or phobias or "panic disorder*" or "overanxious disorder*" or "avoidant disorder*" or agoraphobia or "selective mutism" or "panic attack specifier" or "combat disorder*" or "mixed disorder*" or neurosis or neuroses or neurotic or "school refusal":ti (Word variations have been searched)

#3 anxiety or anxious or phobic or fear or fears or phobia or phobias or "panic disorder*" or "overanxious disorder*" or "avoidant disorder*" or agoraphobia or "selective mutism" or "panic attack specifier" or "combat disorder*" or "mixed disorder*" or neurosis or neuroses or neurotic or "school refusal":ab (Word variations have been searched)

#4 #1 or #2 or #3

#5 MeSH descriptor: [Psychotherapy] explode all trees

#6 psychother* or behavio* or cogniti* or CBT or interpersonal or relaxation or bibliotherap* or counsel* or "family treatment" or supportive or problem-solving or "problem solving" or psychodynamic

© 2018 Zhou X et al. JAMA Psychiatry.

or exposure or modeling or "reinforced practice" or "systematic desensitization" or "eye movement desensitization and reprocessing" or EMDR or self-talk or "self talk" or "emotive imagery" or "contingency management" or therapy:ti (Word variations have been searched)

#7 psychother* or behavio* or cogniti* or CBT or interpersonal or relaxation or bibliotherap* or counsel* or "family treatment" or supportive or problem-solving or "problem solving" or psychodynamic or exposure or modeling or "reinforced practice" or "systematic desensitization" or "eye movement desensitization and reprocessing" or EMDR or self-talk or "self talk" or "emotive imagery" or "contingency management" or therapy:ab (Word variations have been searched)

#8 #5 or #6 or #7

#9 adolesc* or child* or boy* or girl* or juvenil* or minors or paediatri* or pediatri* or pubescen* or school* or student* or teen* or young or youth* or class* or preschool or pre-school:ti (Word variations have been searched)

#10 adolesc* or child* or boy* or girl* or juvenil* or minors or paediatri* or pediatri* or pubescen* or school* or student* or teen* or young or youth* or class* or preschool or pre-school:ab (Word variations have been searched)

#11 MeSH descriptor: [Child] explode all trees

#12 MeSH descriptor: [Adolescent] explode all trees

#13 #9 or #10 or #11 or #12

#14 #4 and #8 and #13

OVID (Embase and PsycINFO)

1 exp anxiety disorder/

2 (anxiety or anxious or phobic or fear or fears or phobia or phobias or panic disorder$ or overanxious disorder$ or avoidant disorder$ or agoraphobia or selective mutism or panic attack or combat disorder$ or mixed disorder$ or neurosis or neuroses or neurotic or school refusal).ti.

3 (anxiety or anxious or phobic or fear or fears or phobia or phobias or panic disorder$ or overanxious disorder$ or avoidant disorder$ or agoraphobia or selective mutism or panic attack or combat disorder$ or mixed disorder$ or neurosis or neuroses or neurotic or school refusal).ab.

4 1 or 2 or 3

5 exp psychotherapy/

6 (psychother$ or behavio$ or cogniti$ or CBT or interpersonal or relaxation or bibliotherap$ or counsel$ or family treatment$ or supportive or problem-solving or problem solving or psychodynamic or exposure or modeling or reinforced practice or systematic desensitization or (eye movement

© 2018 Zhou X et al. JAMA Psychiatry.

desensitization and reprocessing) or EMDR or self-talk or self talk or emotive imagery or contingency management or therapy).ti

7 (psychother$ or behavio$ or cogniti$ or CBT or interpersonal or relaxation or bibliotherap$ or counsel$ or family treatment$ or supportive or problem-solving or problem solving or psychodynamic or exposure or modeling or reinforced practice or systematic desensitization or (eye movement desensitization and reprocessing) or EMDR or self-talk or self talk or emotive imagery or contingency management or therapy).ab

8 5 or 6 or 7

9 (adolesc$ or child$ or boy$ or girl$ or juvenil$ or minors or paediatri$ or pediatri$ or pubescen$ or school$ or student$ or teen$ or young or youth$ or class$ or preschool or pre-school).ti.

10 (adolesc$ or child$ or boy$ or girl$ or juvenil$ or minors or paediatri$ or pediatri$ or pubescen$ or school$ or student$ or teen$ or young or youth$ or class$ or preschool or pre-school).ab.

11 *child/

12 *adolescent/

13 9 or 10 or 11 or 12

14 exp controlled clinical trial/ or exp "controlled clinical trial (topic)"/

15 14 use emez

16 exp clinical trials/

17 16 use psyh

18 (random$ or allocate$ or assign$ or cross over$ or crossover$ or controlled).ti.

19 (random$ or allocate$ or assign$ or cross over$ or crossover$ or controlled).ab.

20 15 or 16 or 18 or 19

21 4 and 8 and 13 and 20

22 limit 21 to clinical trial [Limit not valid in PsycINFO; records were retained]

23 limit 22 to human

24 limit 23 to ("0451 prospective study" or "2000 treatment outcome/clinical trial") [Limit not valid in Embase; records were retained]

25 remove duplicates from 24

© 2018 Zhou X et al. JAMA Psychiatry.

Web of Science

#1 TS=(anxiety OR anxious OR phobic OR fear OR fears OR phobia OR phobias OR "panic disorder*" OR "overanxious disorder*" OR "avoidant disorder*" OR agoraphobia OR "selective mutism" OR "panic attack specifier" OR "combat disorder*" OR "mixed disorder*" OR neurosis OR neuroses OR neurotic OR "school refusal") Indexes=SCI-EXPANDED, SSCI, CPCI-S, CPCI-SSH Timespan=All years

#2 TS=(psychother* OR behavio* OR cogniti* OR CBT OR interpersonal OR relaxation OR bibliotherap* OR counsel* OR "family treatment" OR supportive OR problem-solving OR "problem solving" OR psychodynamic OR exposure OR modeling OR "reinforced practice" OR "systematic desensitization" OR "eye movement desensitization and reprocessing" OR EMDR OR self-talk OR "self talk" OR "emotive imagery" OR "contingency management" OR therapy) Indexes=SCI-EXPANDED, SSCI, CPCI-S, CPCI-SSH Timespan=All years

#3 TS=(adolesc* OR child* OR boy* OR girl* OR juvenil* OR minors OR paediatri* OR pediatri* OR pubescen* OR school* OR student* OR teen* OR young OR youth* OR class* OR preschool OR pre-school) Indexes=SCI-EXPANDED, SSCI, CPCI-S, CPCI-SSH Timespan=All years

#4 TS=(random* OR allocate* OR assign* OR "cross over*" OR crossover* OR controlled) Indexes=SCI-EXPANDED, SSCI, CPCI-S, CPCI-SSH Timespan=All years

#5 #1 AND #2 AND #3 AND #4

#6 #5 Refined by: DOCUMENT TYPES: (ARTICLE OR MEETING ABSTRACT) AND WEB OF SCIENCE CATEGORIES: (PSYCHIATRY) Indexes=SCI-EXPANDED, SSCI, CPCI-S, CPCI-SSH Timespan=All years

EBSCO (CINAHL)

S1 MH "Anxiety Disorders+"

S2 TI (anxiety OR anxious OR phobic OR fear OR fears OR phobia OR phobias OR "panic disorder*" OR "overanxious disorder*" OR "avoidant disorder*" OR agoraphobia OR "selective mutism" OR "panic attack specifier" OR "combat disorder*" OR "mixed disorder*" OR neurosis OR neuroses OR neurotic OR "school refusal")

S3 AB (anxiety OR anxious OR phobic OR fear OR fears OR phobia OR phobias OR "panic disorder*" OR "overanxious disorder*" OR "avoidant disorder*" OR agoraphobia OR "selective mutism" OR "panic attack specifier" OR "combat disorder*" OR "mixed disorder*" OR neurosis OR neuroses OR neurotic OR "school refusal")

S4 S1 or S2 or S3

S5 MH "Psychotherapy+"

© 2018 Zhou X et al. JAMA Psychiatry.

S6 TI (psychother* OR behavio* OR cogniti* OR CBT OR interpersonal OR relaxation OR bibliotherap* OR counsel* OR “family treatment” OR supportive OR problem-solving OR “problem solving” OR psychodynamic OR exposure OR modeling OR “reinforced practice” OR “systematic desensitization” OR “eye movement desensitization and reprocessing” OR EMDR OR self-talk OR “self talk” OR “emotive imagery” OR “contingency management” OR therapy)

S7 AB (psychother* OR behavio* OR cogniti* OR CBT OR interpersonal OR relaxation OR bibliotherap* OR counsel* OR “family treatment” OR supportive OR problem-solving OR “problem solving” OR psychodynamic OR exposure OR modeling OR “reinforced practice” OR “systematic desensitization” OR “eye movement desensitization and reprocessing” OR EMDR OR self-talk OR “self talk” OR “emotive imagery” OR “contingency management” OR therapy)

S8 S5 or S6 or S7

S9 TI (adolesc* OR child* OR boy* OR girl* OR juvenil* OR minors OR paediatri* OR pediatri* OR pubescen* OR school* OR student* OR teen* OR young OR youth* OR class* OR preschool OR pre-school)

S10 AB (adolesc* OR child* OR boy* OR girl* OR juvenil* OR minors OR paediatri* OR pediatri* OR pubescen* OR school* OR student* OR teen* OR young OR youth* OR class* OR preschool OR pre-school)

S11 (MM "Child") OR (MM "Adolescence")

S12 S9 or S10 or S11

S13 MH "Clinical Trials+"

S14 TI (random* OR allocate* OR assign* OR “cross over*” OR crossover* OR controlled)

S15 AB (random* OR allocate* OR assign* OR “cross over*” OR crossover* OR controlled)

S16 S13 or S14 or S15

S17 S4 and S8 and S12 and S16

ProQuest Dissertations

S1 TI,AB(anxiety OR anxious OR phobic OR fear OR fears OR phobia OR phobias OR "panic disorder*" OR "overanxious disorder*" OR "avoidant disorder*" OR agoraphobia OR "selective mutism" OR "panic attack speci?er" OR "combat disorder*" OR "mixed disorder*" OR neurosis OR neuroses OR neurotic OR "school refusal")

S2 TI,AB(psychother* OR behavio* OR cogniti* OR CBT OR interpersonal OR relaxation OR bibliotherap* OR counsel* OR “family treatment” OR supportive OR problem-solving OR “problem solving” OR psychodynamic OR exposure OR modeling OR “reinforced practice” OR “systematic desensitization” OR “eye movement desensitization and reprocessing” OR EMDR OR self-talk OR “self

© 2018 Zhou X et al. JAMA Psychiatry.

talk” OR “emotive imagery” OR “contingency management” OR therapy)

S3 TI(adolesc* OR child* OR boy* OR girl* OR juvenil* OR minors OR paediatri* OR pediatri* OR pubescen* OR school* OR student* OR teen* OR young OR youth* OR class* OR preschool OR pre-school)

S4 TI,AB(random* OR allocate* OR assign* OR “cross over*” OR crossover* OR controlled)

S5 S1 AND S2 AND S3 AND S4

LILACS

#1 (anxiety or anxious or phobic or fear or fears or phobia or phobias or "panic disorder$" or "overanxious disorder$" or "avoidant disorder$" or agoraphobia or "selective mutism" or "panic attack specifier" or "combat disorder$" or "mixed disorder$" or neurosis or neuroses or neurotic or "school refusal") and (psychother$ or behavio$ or cogniti$ or CBT or interpersonal or relaxation or bibliotherap$ or counsel$ or "family treatment" or supportive or problem-solving or "problem solving" or psychodynamic or exposure or modeling or "reinforced practice" or "systematic desensitization" or "eye movement desensitization and reprocessing" or EMDR or self-talk or "self talk" or "emotive imagery" or "contingency management" or therapy) and (adolesc$ or child$ or boy$ or girl$ or juvenil$ or minors or paediatri$ or pediatri$ or pubescen$ or school$ or student$ or teen$ or young or youth$ or class$ or preschool or pre-school) [Title words] or (anxiety or anxious or phobic or fear or fears or phobia or phobias or "panic disorder$" or "overanxious disorder$" or "avoidant disorder$" or agoraphobia or "selective mutism" or "panic attack specifier" or "combat disorder$" or "mixed disorder$" or neurosis or neuroses or neurotic or "school refusal") and (psychother$ or behavio$ or cogniti$ or CBT or interpersonal or relaxation or bibliotherap$ or counsel$ or "family treatment" or supportive or problem-solving or "problem solving" or psychodynamic or exposure or modeling or "reinforced practice" or "systematic desensitization" or "eye movement desensitization and reprocessing" or EMDR or self-talk or "self talk" or "emotive imagery" or "contingency management" or therapy) and (adolesc$ or child$ or boy$ or girl$ or juvenil$ or minors or paediatri$ or pediatri$ or pubescen$ or school$ or student$ or teen$ or young or youth$ or class$ or preschool or pre-school) [Abstract words]

Other sources: Some key journals and conference proceedings, including *J Anxiety Disord, J Child Adolesc Psychopharmacol, J Clin Child Adolesc Psychol, Clin Psychol Rev, J Child Psychol Psychiatry, Clin Child Psychol Psychiatry, J Am Acad Child Adolesc Psychiatry, J Consult Clin Psychol, Cognit Ther Res, Psychopharmacol Bull, Psychother Psychosom, Arch Gen Psychiatry, Am J Psychiatry, Depress Anxiety*, were hand-searched. Additional relevant studies were obtained by scanning relevant systematic reviews, meta-analyses, and reviews as well as reference lists of eligible trials.

© 2018 Zhou X et al. JAMA Psychiatry.

eMethods 3. Description of Psychotherapeutic Interventions and Control Conditions

Description of Psychotherapeutic interventions and control conditions

Interventions	Abbreviation	Description
Psychotherapeutic Intervention:		
Behavioral therapy	BT	BT uses some kind of behavioral training and psychoeducation. BT programs provide parents and youths information about MDD and interventions; teach youths to monitor their mood, thoughts and behaviors; proposed pleasant activity scheduling and behavioral activation. It should not include cognitive restructuring.
Cognitive-behavioral therapy	CBT	CBT is a combination of BT and CT. It therefore should include cognitive restructuring. Additional CBT skill-building techniques are used in many programs by teaching relaxation techniques to cope with environmental stressors, providing social skills and resolution training, and teaching general problem-solving.
Bibliotherapy	BIB	BIB is an unstructured therapy in which parents are provided with a self-help book to be used for guided parent-delivered psychotherapy.
Control conditions:		
No-treatment	NT	NT is a control condition in which the participants receive no active treatment during the study and in which they do not expect to receive such after the study is over.
Psychological placebo	PBO	PBO is a control condition that was regarded as inactive by the researchers but was presented to the participants as being an active therapy.
Treatment-as-usual	TAU	TAU is not considered to be structured psychotherapy but may have some treatment effects.
Waitlist	WL	WL is a control condition in which the participants receive no active treatment during the study but are forewarned that they can receive one after the study period is over.

© 2018 Zhou X et al. JAMA Psychiatry.

eMethods 4. Hierarchy of Anxiety Scales

1. Hierarchy of anxiety symptom severity measurement scales

Hierarchy	Anxiety symptom severity rating scales	Abbreviation
1	Revised Children's Manifest Anxiety Scale	RCMAS
2	Spence Children's Anxiety Scale	SCAS
3	Multidimensional Anxiety Scale for Children	MASC
4	State-Trait Anxiety Inventory for Children	STAIC
5	Screen for Anxiety and Related Disorders	SCARED
6	Revised Child Anxiety and Depression Scale	RCADS
7	Clinician severity ratings	CSR
8	Fear Survey Schedule for Children	FSSC
9	Childhood Anxiety Sensitivity Index	CASI
10	Beck Anxiety Inventory	BAI
11	Child Behavior Checklist-Internalising	CBCL-Int
12	Pediatric Anxiety Rating Scale	PARS

Note: where different anxiety symptom severity rating scales were used, for the purpose of pooling results, we chose the single best available outcome measure according to a hierarchy based on psychometric properties and appropriateness for use with children and adolescents.

2. Hierarchy of quality of life and functional improvement measurement scales

Hierarchy	Anxiety symptom severity rating scales	Abbreviation
1	Quality of Life Inventory	QoLI
2	Pediatric Quality of Life Inventory	QoL Child Report
3	Children's Global Assessment Scale	CGAS
4	The Inventory for the Assessment of Quality of Life	IQL
5	Sheehan Disability Scale	SDS
6	Columbia Impairment Scale	CIS

Note: where different quality of life and functional improvement rating scales were used, for the purpose of pooling results, we chose the single best available outcome measure according to a hierarchy based on psychometric properties and appropriateness for use with children and adolescents.

© 2018 Zhou X et al. JAMA Psychiatry.

eMethods 5. Network Meta-analysis Model

NMA model description

1. Random Effects Model for Continuous Data in WinBUGS

y=a table of the arm-means, sd=a table of the arm sd, n=a table of the arm sample size, t=a table with the names (numbers) of treatments, na=a vector with the number of arms in each study, ref=a number specifying which is the reference treatment

```
model{
for(i in 1:ns){
w[i,1] <- 0
delta[i,t[i,1]]<-0
u[i] ~ dnorm(0,.0001)

for (k in 1:na[i])    {
se[i,t[i,k]]<- sd[i,t[i,k]]/sqrt(n[i,t[i,k]])
var[i,t[i,k]]<- se[i,t[i,k]]*se[i,t[i,k]]
prec[i,t[i,k]]<- 1/var[i,t[i,k]]

#normal likelihood
y[i,t[i,k]] ~ dnorm(phi[i,t[i,k]],prec[i,t[i,k]])
phi[i,t[i,k]]<- (u[i]+delta[i,t[i,k]])*pooled.sd[i]

#calculate the pooled SD
nom1[i,k]<- n[i,t[i,k]]*sd[i,t[i,k]]*sd[i,t[i,k]]    #nominator for the pooled sd
        }

ss[i]<- sum(n[i,1:nt])-nt+na[i]    #total sample size in a study
nom[i]<- sum(nom1[i,1:na[i]])    #nominator for the pooled sd
pooled.sd[i]<- sqrt(nom[i]/(ss[i]-na[i]))    #pooled sd

for (k in 2:na[i]) {
delta[i,t[i,k]] ~ dnorm(md[i,t[i,k]],taud[i,t[i,k]])    # trial-specific SMD distributions
md[i,t[i,k]]<- d[t[i,k]]-d[t[i,1]]+sw[i,k]    # mean of SMD distributions
taud[i,t[i,k]]<- tau*2*(k-1)/k    #precision of SMD distributions
w[i,k] <- (delta[i,t[i,k]]-d[t[i,k]]+d[t[i,1]])    #adjustment, multi-arm RCTs
sw[i,k] <- sum(w[i,1:k-1])/(k-1) }    # cumulative adjustment for multi-arm trials
        }

d[ref]<-0
for (k in 2:nt) {d[k] ~ dnorm(0,.0001) }
```


© 2018 Zhou X et al. JAMA Psychiatry.

```
SD~dunif(0,1)    #vague prior for random effects standard deviation
tau<-1/pow(SD,2)

# Collection of results#
# pairwise SMDs
# for all comparisons
for (c in 1:(nt-1)) { for (k in (c+1):nt) { SMD[c,k] <- d[c] - d[k] }    #to have negative values
        }

#Fit of the Model#
for(i in 1:ns) {
for(k in 1:na[i]) {
Darm[i,k]<-(y[i,t[i,k]]-phi[i,t[i,k]])*(y[i,t[i,k]]-phi[i,t[i,k]])/var[i,t[i,k]]
        }
D[i]<- sum(Darm[i,1:na[i]])
        }
D.bar<- sum(D[])
}
```

2. Random Effects Model for Dichotomous Data in WinBUGS

r= a table of the number of events, n=a table of the arm sample size, t=a table with the names (numbers) of treatments, na=a vector with the number of arms in each study, ref=a number specifying which is the reference treatment

```
model {
for(i in 1:ns) {
w[i,1]<- 0
delta[i,t[i,1]]<- 0

#Binomial Likelihood#
for (k in 1:na[i]) {
r[i,t[i,k]] ~ dbin(p[i,t[i,k]],n[i,t[i,k]])
        }
#Parameterization of the model#
logit(p[i,t[i,1]])<- mu[i]
for (k in 2:na[i]) {
logit(p[i,t[i,k]])<- mu[i] + delta[i,t[i,k]]
delta[i,t[i,k]] ~ dnorm(md[i,t[i,k]],taud[i,t[i,k]])
taud[i,t[i,k]]<- tau *2*(k-1)/k
md[i,t[i,k]]<-d[t[i,k]] - d[t[i,1]]    + sw[i,k]
w[i,k]<- (delta[i,t[i,k]]    - d[t[i,k]] + d[t[i,1]])
sw[i,k]<- sum(w[i,1:k-1])/(k-1)
        }
        }
```


© 2018 Zhou X et al. JAMA Psychiatry.

```
#Priors#
sd ~ dnorm(0,1)I(0,1)
tau<- 1/pow(sd,2)

for(k in 1:(ref-1)) {
d[k] ~ dnorm(0,.0001)
        }
for(k in (ref+1):nt) {
d[k] ~ dnorm(0,.0001)
        }
for(i in 1:ns) {
mu[i] ~ dnorm(0,.0001)
        }

# Collection of results#
#Estimated & Predicted Odds Ratios#
d[ref]<- 0
for(i in 1:(nt-1)) {
for (j in (i+1):nt) {
OR[i,j]<- exp(d[i] - d[j])
LOR[i,j]<- d[i] - d[j]
        }
        }

#Fit of the Model#

for(i in 1:ns) {
for (k in 1:na[i]) {
Darm[i,k]<- -2*( r[i,t[i,k]] *log(n[i,t[i,k]]*p[i,t[i,k]]/ r[i,t[i,k]])+(n[i,t[i,k]] -
        r[i,t[i,k]])*log((n[i,t[i,k]]-n[i,t[i,k]]* p[i,t[i,k]])/(n[i,t[i,k]]- r[i,t[i,k]])))
        }
D[i]<- sum(Darm[i,1:na[i]])
        }
D.bar<- sum(D[])
}
```


© 2018 Zhou X et al. JAMA Psychiatry.

eMethods 6. References for Included Trials

1. Afshari A, Neshat-Doost HT, Maracy MR et al. The effective comparison between emotion-focused cognitive behavioral group therapy and cognitive behavioral group therapy in children with separation anxiety disorder. J Res Med Sci 2014;19:221-7.
2. Arendt K, Thastum M, Hougaard E. Efficacy of a Danish version of the Cool Kids program: a randomized wait-list controlled trial. Acta Psychiatr Scand. 2015 May 27. doi: 10.1111/acps.
3. Azadeh SM, Kazemi-Zahrani H, Besharat MA. Effectiveness of acceptance and commitment therapy on interpersonal problemsand psychological flexibility in female high school students with social anxiety disorder. Glob J Health Sci 2015;8:131-8.
4. Baer S, Garland EJ. Pilot study of community-based cognitive behavioral group therapy for adolescents with social phobia. J Am Acad Child Adolesc Psychiatry 2005;44:258-64.
5. Barrett PM, Dadds MR, Rapee RM. Family treatment of childhood anxiety: a controlled trial. J Consult Clin Psychol 1996;64:333-42.
6. Barrett PM. Evaluation of cognitive-behavioral group treatments for childhood anxiety disorders. J Clin Child Psychol 1998;27:459-68.
7. Barrington J, Prior M, Richardson M et al. Effectiveness of CBT versus standard treatment for childhood anxiety disorders in a community clinic setting. Behav Change 2005;22:29-43.
8. Beidel DC, Turner SM, Morris TL. Behavioral treatment of childhood social phobia. J Consult Clin Psychol 2000;68:1072-80.
9. Bergman RL, Gonzalez A, Piacentini J et al. Integrated behavior therapy for selective mutism: a randomized controlled pilot study. Behav Res Ther 2013;51:680-9.
10. Bodden DH, Bögels SM, Nauta MH et al. Child versus family cognitive-behavioral therapy in clinically anxious youth: an efficacy and partial effectiveness study. J Am Acad Child Adolesc Psychiatry 2008;47:1384-94.
11. Cartwright-Hatton S, McNally D, Field AP et al. A new parenting-based group intervention for young anxious children: results of a randomized controlled trial. J Am Acad Child Adolesc Psychiatry 2011;50:242-51.
12. Chalfant AM, Rapee R, Carroll L. Treating anxiety disorders in children with high functioning autism spectrum disorders: a controlled trial. J Autism Dev Disord 2007;37:1842-57.
13. Chavira DA, Drahota A, Garland AF et al. Feasibility of two modes of treatment delivery for child anxiety in primary care. Behav Res Ther 2014;60:60-6.
14. Chiu AW, Langer DA, McLeod BD et al. Effectiveness of modular CBT for child anxiety in elementary schools. Sch Psychol Q 2013;28:141-53.
15. Chu BC, Crocco ST, Esseling P et al. Transdiagnostic group behavioral activation and exposure therapy for youth anxiety and depression: initial randomized controlled trial. Behav Res Ther 2016:76:65-75.
16. Cobham VE, Dadds MR, Spence SH. The role of parental anxiety in the treatment of childhood anxiety. J Consult Clin Psychol 1998;66:893-905.
17. Cobham VE. Do anxiety-disordered children need to come into the clinic for efficacious treatment?

© 2018 Zhou X et al. JAMA Psychiatry.

J Consult Clin Psychol 2012;80:465-76.
18. Cobham VE, Filus A, Sanders MR. Working with parents to treat anxiety-disordered children: A proof of concept RCT evaluating Fear-less Triple P. Behav Res Ther 2017;95:128-38.
19. Conaughton RJ, Donovan CL, March S. Efficacy of an Internet-based CBT program for children with comorbid high functioning autism spectrum disorder and anxiety: A randomised controlled trial. J Affect Disord 2017;218:260-8.
20. Cornwall E, Spence SH, Schotte D. The effectiveness of emotive imagery in the treatment of darkness phobia in children. Behav Change 1996;13:223-9.
21. Creswell C, Cruddace S, Gerry S et al. Treatment of childhood anxiety disorder in the context of maternal anxiety disorder: a randomised controlled trial and economic analysis. Health Technol Assess 2015;19:1-184.
22. de Groot J, Cobham V, Leong J et al. Individual versus group family-focused cognitive-behaviour therapy for childhood anxiety: pilot randomized controlled trial. Aust N Z J Psychiatry 2007;41:990-7.
23. Donovan CL, March S. Online CBT for preschool anxiety disorders: a randomised control trial. Behav Res Ther 2014;58:24-35.
24. Ebrahiminejad S, Poursharifi H, Bakhshiour Roodsari A et al. The Effectiveness of Mindfulness-Based Cognitive Therapy on Iranian Female Adolescents Suffering From Social Anxiety. Iran Red Crescent Med J 2016;18:e25116.
25. Esbjørn BH, Reinholdt-Dunne ML, Nielsen SK et al. Exploring the effect of case formulation driven CBT for children with anxiety disorders: a feasibility study. Behav Cogn Psychother 2015;43:20-30.
26. Flannery-Schroeder EC, Kendall PC. Group and individual cognitive-behavioral treatments for youth with anxiety disorders: a randomized clinical trial. Cogn Ther Res 2000;24:251-78.
27. Fujii C, Renno P, McLeod BD et al. Intensive cognitive behavioral therapy for anxiety disorders in school-aged children with autism: a preliminary comparison with treatment-as-usual. Sch Ment Health 2013;5:25-37.
28. Garcia-Lopez LJ, Díaz-Castela Mdel M, Muela-Martinez JA et al. Can parent training for parents with high levels of expressed emotion have a positive effect on their child's social anxiety improvement? J Anxiety Disord 2014;28:812-22.
29. Ginsburg GS, Drake KL. School-based treatment for anxious african-american adolescents: a controlled pilot study. J Am Acad Child Adolesc Psychiatry 2002;41:768-75.
30. Ginsburg GS, Becker KD, Drazdowski TK et al. Treating anxiety disorders in inner city schools: results from a pilot randomized controlled trial comparing CBT and usual care. Child Youth Care Forum 2012;41:1-19.
31. Hancock KM, Swain J, Hainsworth CJ et al. Acceptance and commitment therapy versus cognitive behavior therapy for children with anxiety: outcomes of a randomized controlled trial. J Clin Child Adolesc Psychol 2016;21:1-16.
32. Hayward C, Varady S, Albano AM et al. Cognitive-behavioral group therapy for social phobia in female adolescents: results of a pilot study. J Am Acad Child Adolesc Psychiatry 2000;39:721-6.
33. Herbert JD, Gaudiano BA, Rheingold AA et al. Cognitive behavior therapy for generalized social

© 2018 Zhou X et al. JAMA Psychiatry.

anxiety disorder in adolescents: a randomized controlled trial. J Anxiety Disord 2009;23:167-77.
34. Hirshfeld-Becker DR, Masek B, Henin A et al. Cognitive behavioral therapy for 4- to 7-year-old children with anxiety disorders: a randomized clinical trial. J Consult Clin Psychol 2010;78:498-510.
35. Holmes MC, Donovan CL, Farrell LJ et al. The efficacy of a group-based, disorder-specific treatment program for childhood GAD–a randomized controlled trial. Behav Res Ther 2014;61:122-35.
36. Hudson JL, Rapee RM, Deveney C et al. Cognitive-behavioral treatment versus an active control for children and adolescents with anxiety disorders: a randomized trial. J Am Acad Child Adolesc Psychiatry 2009;48:533-44.
37. Ingul JM, Aune T, Nordahl HM. A randomized controlled trial of individual cognitive therapy, group cognitive behaviour therapy and attentional placebo for adolescent social phobia. Psychother Psychosom 2014;83:54-61.
38. Kendall PC. Treating anxiety disorders in children: results of a randomized clinical trial. J Consult Clin Psychol 1994;62:100-10.
39. Kendall PC, Flannery-Schroeder E, Panichelli-Mindel SM et al. Therapy for youths with anxiety disorders: a second randomized clinical trial. J Consult Clin Psychol 1997;65:366-80.
40. Kendall PC, Hudson JL, Gosch E et al. Cognitive-behavioral therapy for anxiety disordered youth: a randomized clinical trial evaluating child and family modalities. J Consult Clin Psychol 2008;76:282-97.
41. Khanna MS, Kendall PC. Computer-assisted cognitive behavioral therapy for child anxiety: results of a randomized clinical trial. J Consult Clin Psychol 2010;78:737-45.
42. Last CG, Hansen C, Franco N. Cognitive-behavioral treatment of school phobia. J Am Acad Child Adolesc Psychiatry 1998;37:404-11.
43. Lau WY, Chan CK, Li JC et al. Effectiveness of group cognitive-behavioral treatment for childhood anxiety in community clinics. Behav Res Ther 2010;48:1067-77.
44. Leong J, Cobham VE, de Groot J et al. Comparing different modes of delivery: a pilot evaluation of a family-focused, cognitive-behavioral intervention for anxiety-disordered children. Eur Child Adolesc Psychiatry 2009;18:231-9.
45. Liber JM, Van Widenfelt BM, Utens EM et al. No differences between group versus individual treatment of childhood anxiety disorders in a randomised clinical trial. J Child Psychol Psychiatry 2008;49:886-93.
46. Lyneham HJ, Rapee RM. Evaluation of therapist-supported parent-implemented CBT for anxiety disorders in rural children. Behav Res Ther 2006;44:1287-300.
47. Manassis K, Mendlowitz SL, Scapillato D et al. Group and individual cognitive-behavioral therapy for childhood anxiety disorders: a randomized trial. J Am Acad Child Adolesc Psychiatry 2002;41:1423-30.
48. March S, Spence SH, Donovan CL. The efficacy of an internet-based cognitive-behavioral therapy intervention for child anxiety disorders. J Pediatr Psychol 2009;34:474-87.
49. Masia Warner C, Klein RG, Dent HC et al. School-based intervention for adolescents with social anxiety disorder: results of a controlled study. J Abnorm Child Psychol 2005;33:707-22.
50. Masia Warner C, Fisher PH, Shrout PE et al. Treating adolescents with social anxiety disorder in

© 2018 Zhou X et al. JAMA Psychiatry.

school: an attention control trial. J Child Psychol Psychiatry 2007;48:676-86.
51. Masia Warner C, Colognori D, Kim RE et al. Cognitive-behavioral treatment of persistent functional somatic complaints and pediatric anxiety: an initial controlled trial. Depress Anxiety 2011;28:551-9.
52. Masia Warner C, Colognori D, Brice C et al. Can school counselors deliver cognitive-behavioral treatment for social anxiety effectively? A randomized controlled trial. J Child Psychol Psychiatry 2016. doi: 10.1111/jcpp.12550.
53. McConachie H, McLaughlin E, Grahame V et al. Group therapy for anxiety in children with autism spectrum disorder. Autism 2014;18:723-32.
54. Melfsen S, Kühnemund M, Schwieger J et al. Cognitive behavioral therapy of socially phobic children focusing on cognition: a randomised wait-list control study. Child Adolesc Psychiatry Ment Health 2011;5:5.
55. Mendlowitz SL, Manassis K, Bradley S et al. Cognitive-behavioral group treatments in childhood anxiety disorders: the role of parental involvement. J Am Acad Child Adolesc Psychiatry 1999;38:1223-9.
56. Monga S, Rosenbloom BN, Tanha A et al. Comparison of child–parent and parent-only cognitive-behavioral therapy programs for anxious children aged 5 to 7 years: short- and long-term outcomes. J Am Acad Child Adolesc Psychiatry 2015;54:138-46.
57. Muris P, Mayer B, Bartelds E et al. The revised version of the Screen for Child Anxiety Related Emotional Disorders (SCARED-R): treatment sensitivity in an early intervention trial for childhood anxiety disorders. Br J Clin Psychol 2001;40:323-36.
58. Muris P, Meesters C, van Melick M. Treatment of childhood anxiety disorders: a preliminary comparison between cognitive-behavioral group therapy and a psychological placebo intervention. J Behav Ther Exp Psychiatry 2002;33:143-58.
59. Muris P, Meesters C, Gobel M. Cognitive coping vs emotional disclosure in the treatment of anxious children: a pilot-study. Cogn Behav Ther 2002;31:59-67.
60. Nauta MH, Scholing A, Emmelkamp PM et al. Cognitive-behavioural therapy for anxiety disordered children in a clinical setting: does additional cognitive parent training enhance treatment effectiveness? Clin Psychol Psychother 2001;8:330-40.
61. Nauta MH, Scholing A, Emmelkamp PM et al. Cognitive-behavioral therapy for children with anxiety disorders in a clinical setting: no additional effect of a cognitive parent training. J Am Acad Child Adolesc Psychiatry 2003;42:1270-8.
62. Oerbeck B, Stein MB, Wentzel-Larsen T et al. A randomized controlled trial of a home and school-based intervention for selective mutism–defocused communication and behavioural techniques. Child Adolesc Ment Health 2014;19:192-8.
63. Olivares J, Olivares-Olivares PJ, Rosa-Alcázar AI et al. The contribution of the therapist's competence in the treatment of adolescents with generalized social phobia. Psicothema 2014;26:483-9.
64. Ortbandt C, Petermann U. Effects of a cognitive-behavioral training program for children with social anxiety. Kindheit Und Entwicklung 2009;18:21-9.
65. Öst LG, Cederlund R, Reuterskiöld L. Behavioral treatment of social phobia in youth: does parent

© 2018 Zhou X et al. JAMA Psychiatry.

education training improve the outcome? Behav Res Ther 2015;67:19-29.
66. Özyurt G, Gencer Ö, Öztürk Y et al. Is triple p positive parenting program effective on anxious children and their parents? 4th month follow up results. J Child Fam Stud 2016;25:1646-55.
67. Pina AA, Zerr AA, Villalta IK et al. Indicated prevention and early intervention for childhood anxiety: a randomized trial with Caucasian and Hispanic/Latino Youth. J Consult Clin Psychol 2012;80:940-6.
68. Pincus DB, May JE, Whitton SW et al. Cognitive-behavioral treatment of panic disorder in adolescence. J Clin Child Adolesc Psychol 2010;39:638-49.
69. Rapee RM, Abbott MJ, Lyneham HJ. Bibliotherapy for children with anxiety disorders using written materials for parents: a randomized controlled trial. J Consult Clin Psychol 2006;74:436-44.
70. Rodríguez JO, Alcázar AIR, Rodríguez JAP. Early detection and treatment of adolescents with generalized social phobia. Psicothema 2005;17:1-8.
71. Rosa-Alcázar AI, Olivares-Olivares PJ, Rodríguez JO. The role of non-specific effects in the psychological treatment of adolescents with social phobia. Anuario de psicología 2009;40:43-61.
72. Sánchez-García R, Olivares J. Effectiveness of a program for early detection/intervention in children/adolescents with generalized social phobia. Anales de Psicología 2009;25:241-9.
73. Schneider S, Blatter-Meunier J, Herren C et al. Disorder-specific cognitive-behavioral therapy for separation anxiety disorder in young children: a randomized waiting-list-controlled trial. Psychother Psychosom 2011;80:206-15.
74. Schneider S, Blatter-Meunier J, Herren C et al. The efficacy of a family-based cognitive-behavioral treatment for separation anxiety disorder in children aged 8-13: a randomized comparison with a general anxiety program. J Consult Clin Psychol 2013;81:932-40.
75. Sciberras E, Mulraney M, Anderson V et al. Managing anxiety in children with ADHD using cognitive-behavioral therapy: a pilot randomized controlled trial. J Atten Disord 2015. doi: 10.1177/1087054715584054
76. Shortt AL, Barrett PM, Fox TL. Evaluating the FRIENDS program: a cognitive-behavioral group treatment for anxious children and their parents. J Clin Child Psychol 2001;30:525-35.
77. Silk JS, Sheeber L, Tan PZ et al. “You can do it!”: The role of parental encouragement of bravery in child anxiety treatment. J Anxiety Disord 2013;27:439-46.
78. Silverman WK, Kurtines WM, Ginsburg GS et al. Contingency management, self-control, and education support in the treatment of childhood phobic disorders: a randomized clinical trial. J Consult Clin Psychol 1999;67:675-87.
79. Silverman WK, Kurtines WM, Ginsburg GS et al. Treating anxiety disorders in children with group cognitive-behaviorial therapy: a randomized clinical trial. J Consult Clin Psychol 1999;67:995-1003.
80. Silverman WK, Kurtines WM, Jaccard J et al. Directionality of change in youth anxiety treatment involving parents: an initial examination. J Consult Clin Psychol 2009;77:474-85.
81. Siqueland L, Rynn M, Diamond GS. Cognitive behavioral and attachment based family therapy for anxious adolescents: Phase I and II studies. J Anxiety Disord 2005;19:361-81.
82. Smith AM, Flannery-Schroeder EC, Gorman KS et al. Parent cognitive-behavioral intervention for the treatment of childhood anxiety disorders: a pilot study. Behav Res Ther 2014;61:156-61.

© 2018 Zhou X et al. JAMA Psychiatry.

83. Spence SH, Donovan C, Brechman-Toussaint M. The treatment of childhood social phobia: the effectiveness of a social skills training-based, cognitive-behavioural intervention, with and without parental involvement. J Child Psychol Psychiatry 2000;41:713-26.
84. Spence SH, Holmes JM, March S et al. The feasibility and outcome of clinic plus internet delivery of cognitive-behavior therapy for childhood anxiety. J Consult Clin Psychol 2006;74:614-21.
85. Spence SH, Donovan CL, March S et al. A randomized controlled trial of online versus clinic-based CBT for adolescent anxiety. J Consult Clin Psychol 2011;79:629-42.
86. Spence SH, Donovan CL, March S et al. Generic versus disorder specific cognitive behavior therapy for social anxiety disorder in youth: A randomized controlled trial using internet delivery. Behav Res Ther 2017;90:41-57.
87. Storch EA, Arnold EB, Lewin AB et al. The effect of cognitive-behavioral therapy versus treatment as usual for anxiety in children with autism spectrum disorders: a randomized, controlled trial. J Am Acad Child Adolesc Psychiatry 2013;52:132-42.
88. Storch EA, Lewin AB, Collier AB et al. A randomized controlled trial of cognitive-behavioral therapy versus treatment as usual for adolescents with autism spectrum disorders and comorbid anxiety. Depress Anxiety 2015, 32:174-81.
89. Storch EA, Salloum A, King MA et al. A randomized controlled trial in community mental health centers of computer-assisted cognitive behavioral therapy versus treatment as usual for children with anxiety. Depress Anxiety 2015;32:843-52.
90. Thirlwall K, Cooper PJ, Karalus J et al. Treatment of child anxiety disorders via guided parent-delivered cognitive-behavioural therapy: randomised controlled trial. Br J Psychiatry 2013;203:436-44.
91. Tillfors M, Andersson G, Ekselius L et al. A randomized trial of Internet-delivered treatment for social anxiety disorder in high school students. Cogn Behav Ther 2011;40:147-57.
92. Treadwell KR, Kendall PC. Self-talk in youth with anxiety disorders: states of mind, content specificity, and treatment outcome. J Consult Clin Psychol 1996;64:941-50.
93. Vigerland S, Ljótsson B, Thulin U et al. Internet-delivered cognitive behavioural therapy for children with anxiety disorders: a randomised controlled trial. Behav Res Ther 2016;76:47-56.
94. Waters AM, Ford LA, Wharton TA et al. Cognitive-behavioural therapy for young children with anxiety disorders: comparison of a child + parent condition versus a parent only condition. Behav Res Ther 2009;47:654-62.
95. Wergeland GJ, Fjermestad KW, Marin CE et al. An effectiveness study of individual vs. group cognitive behavioral therapy for anxiety disorders in youth. Behav Res Ther 2014;57:1-12.
96. White SW, Ollendick T, Albano AM et al. Randomized controlled trial: multimodal anxiety and social skill intervention for adolescents with autism spectrum disorder. J Autism Dev Disord 2013;43:382-94.
97. Whiteside SP, Ale CM, Young B et al. The feasibility of improving CBT for childhood anxiety disorders through a dismantling study. Behav Res Ther 2015;73:83-9.
98. Wood JJ, Piacentini JC, Southam-Gerow M et al. Family cognitive behavioral therapy for child anxiety disorders. J Am Acad Child Adolesc Psychiatry 2006;45:314-21.
99. Wood JJ, Drahota A, Sze K et al. Cognitive behavioral therapy for anxiety in children with autism

© 2018 Zhou X et al. JAMA Psychiatry.

spectrum disorders: a randomized, controlled trial. J Child Psychol Psychiatry 2009;50:224-34.
100. Wood JJ, Ehrenreich-May J, Alessandri M et al. Cognitive behavioral therapy for early adolescents with autism spectrum disorders and clinical anxiety: a randomized, controlled trial. Behav Ther 2015;46:7-19.
101. Wuthrich VM, Rapee RM, Cunningham MJ et al. A randomized controlled trial of the cool teens cd-rom computerized program for adolescent anxiety. J Am Acad Child Adolesc Psychiatry 2012;51:261-70.

© 2018 Zhou X et al. JAMA Psychiatry.

eMethods 7. Risk of Bias Assessment

Risk of bias graph: it is a plot of the distribution of judgments (Yes, No, Unclear) across studies for each risk of bias entry

Each domain in the tool includes one or more specific entries in a 'Risk of bias' table. Within each entry, the first part of the tool describes what was reported to have happened in the study, in sufficient detail to support a judgement about the risk of bias. The second part of the tool assigns a judgement relating to the risk of bias for that entry. This is achieved by assigning a judgement of 'Low risk' of bias, 'High risk' of bias, or 'Unclear risk' of bias.

The domains of sequence generation, allocation concealment and selective outcome reporting should each be addressed in the tool by a single entry for each study. For blinding of participants and personnel, blinding of outcome assessment and for incomplete outcome data, two or more entries may be used because assessments generally need to be made separately for different outcomes (or for the same outcome at different time points).

**Domain**	**Support for judgement**	**Review authors' judgement**
Selection bias.		
**Random sequence generation.**	Describe the method used to generate the allocation sequence in sufficient detail to allow an assessment of whether it should produce comparable groups.	Selection bias (biased allocation to interventions) due to inadequate generation of a randomised sequence.
**Allocation concealment.**	Describe the method used to conceal the allocation sequence in sufficient detail to determine whether intervention allocations could have been foreseen in advance of, or during, enrolment.	Selection bias (biased allocation to interventions) due to inadequate concealment of allocations prior to assignment.
Performance bias.		
**Blinding of participants and personnel**	Describe all measures used, if any, to blind study participants and personnel from knowledge of which intervention a participant received. Provide any information relating to whether the intended blinding was effective.	Performance bias due to knowledge of the allocated interventions by participants and personnel during the study.
Detection bias.		
**Blinding of outcome assessment**	Describe all measures used, if any, to blind outcome assessors from knowledge of which	Detection bias due to knowledge of the

© 2018 Zhou X et al. JAMA Psychiatry.

	intervention a participant received. Provide any information relating to whether the intended blinding was effective.	allocated interventions by outcome assessors.
Attrition bias.		
**Incomplete outcome data**	Describe the completeness of outcome data for each main outcome, including attrition and exclusions from the analysis. State whether attrition and exclusions were reported, the numbers in each intervention group (compared with total randomized participants), reasons for attrition/exclusions where reported, and any re-inclusions in analyses performed by the review authors.	Attrition bias due to amount, nature or handling of incomplete outcome data.
Reporting bias.		
**Selective reporting.**	State how the possibility of selective outcome reporting was examined by the review authors, and what was found.	Reporting bias due to selective outcome reporting.
Other bias.		
**Other sources of bias.**	State any important concerns about bias not addressed in the other domains in the tool. If particular questions/entries were pre-specified in the review's protocol, responses should be provided for each question/entry.	Bias due to problems not covered elsewhere in the table.

© 2018 Zhou X et al. JAMA Psychiatry.

Study	Random sequence generation (selection bias)	Allocation concealment (selection bias)	Blinding of participants and personnel (performance bias)	Blinding of outcome assessment (detection bias)	Incomplete outcome data (attrition bias)	Selective reporting (reporting bias)	Other bias
Afshari 2014	?	?	−	−	?	+	?
Arendt 2016	+	+	−	−	+	+	+
Azadeh 2016	?	?	−	−	?	+	+
Baer 2005	+	?	−	−	+	+	?
Barrett 1996	?	?	−	−	+	+	+
Barrett 1998	?	?	−	−	+	+	?
Barrington 2005	?	?	−	−	?	+	+
Beidel 2000	+	?	−	−	+	+	+
Bergman 2013	+	?	−	+	+	+	−
Bodden 2008	?	?	−	−	+	+	?
Cartwright-Hatton 2011	+	+	−	−	+	+	?
Chalfant 2007	?	?	−	−	+	+	+
Chavira 2014	?	?	−	−	+	+	+
Chiu 2013	?	?	−	−	+	+	+
Chu 2016	+	?	−	−	+	+	+
Cobham 1998	?	?	−	−	?	+	+
Cobham 2012	?	?	−	−	+	+	−
Cobham 2017	+	?	−	−	+	+	+
Conaughton 2017	+	?	−	−	?	+	?
Cornwall 1996	?	?	−	−	−	?	?
Creswell 2015	+	+	−	−	+	+	+
de Groot 2007	?	?	−	−	+	+	+
Donovan 2014	+	+	−	+	+	+	+
Ebrahiminejad 2016	−	?	−	−	+	+	+
Esbjørn 2015	+	?	−	−	?	?	+
Flannery-Schroeder 2000	+	+	−	−	+	+	−
Fujii 2013	?	?	−	+	−	?	−
Garcia-Lopez 2014	?	?	−	+	?	+	?
Ginsburg 2002	+	?	−	−	+	+	+
Ginsburg 2012	+	?	−	−	+	+	−
Hancock 2016	+	?	−	−	+	+	+
Hayward 2000	−	?	−	+	+	+	+
Herbert 2009	+	?	−	−	+	+	+

© 2018 Zhou X et al. JAMA Psychiatry.

	Random sequence generation (selection bias)	Allocation concealment (selection bias)	Blinding of participants and personnel (performance bias)	Blinding of outcome assessment (detection bias)	Incomplete outcome data (attrition bias)	Selective reporting (reporting bias)	Other bias
Hirshfeld-Becker 2010	+	+	−	+	+	+	+
Holmes 2014	+	?	−	−	+	+	−
Hudson 2009	+	?	−	−	+	+	−
Ingul 2014	+	?	−	−	−	?	−
Kendall 1994	?	?	−	−	−	+	−
Kendall 1997	?	?	−	−	?	+	−
Kendall 2008	+	?	−	−	+	+	+
Khanna 2010	+	?	−	−	+	+	+
Last 1998	?	?	−	−	+	+	+
Lau 2010	?	?	−	−	+	+	−
Leong 2009	?	?	−	−	?	?	?
Liber 2008	?	?	−	−	+	+	+
Lyneham 2006	?	?	−	−	?	+	+
Manassis 2002	?	?	−	−	?	+	+
March 2009	+	+	−	−	+	+	+
Masia-Warner 2005	?	?	−	+	+	?	+
Masia-Warner 2007	?	+	−	+	+	?	+
Masia-Warner 2011	+	+	−	+	+	+	−
Masia-Warner 2016	+	?	−	+	+	+	+
McConachie 2014	+	+	−	−	+	+	?
Melfsen 2011	+	+	−	+	+	+	−
Mendlowitz 1999	?	?	−	−	+	+	+
Monga 2015	?	?	−	+	+	+	+
Muris 2001	?	?	−	−	+	+	+
Muris 2002a	?	?	−	−	?	+	+
Muris 2002b	?	?	−	−	+	+	?
Nauta 2001	?	?	−	−	+	+	+
Nauta 2003	?	?	−	−	?	?	?
Oerbeck 2014	+	+	−	+	+	?	+
Olivares 2005	+	?	−	−	+	+	+
Olivares 2014	?	?	−	−	?	+	+
Ortbandt 2009	?	?	−	?	−	−	?
Öst 2015	+	+	−	−	+	+	+
Özyurt 2016	+	?	−	−	?	−	+

© 2018 Zhou X et al. JAMA Psychiatry.

	Random sequence generation (selection bias)	Allocation concealment (selection bias)	Blinding of participants and personnel (performance bias)	Blinding of outcome assessment (detection bias)	Incomplete outcome data (attrition bias)	Selective reporting (reporting bias)	Other bias
Pina 2012	?	?	−	−	+	+	+
Pincus 2010	?	?	−	−	+	+	−
Rapee 2006	+	?	−	−	+	+	+
Rosa-Alcázar 2009	?	?	−	−	+	+	+
Sánchez-García 2009	?	?	−	−	+	+	+
Schneider 2011	+	?	−	−	+	+	+
Schneider 2013	+	+	−	−	+	+	?
Sciberras 2015	+	+	−	−	+	+	−
Shortt 2001	?	?	−	−	+	+	+
Silk 2013	?	?	−	?	+	+	?
Silverman 1999a	?	?	−	−	+	+	+
Silverman 1999b	?	?	−	−	−	+	−
Silverman 2009	?	?	−	−	+	+	+
Siqueland 2005	?	?	−	−	?	+	?
Smith 2014	+	?	−	−	−	+	?
Spence 2000	?	?	−	−	+	+	+
Spence 2006	+	?	−	−	+	+	+
Spence 2011	+	?	−	−	+	+	−
Spence 2017	+	?	−	−	+	+	+
Storch 2013	+	?	−	+	+	+	+
Storch 2015a	+	?	−	+	+	+	+
Storch 2015b	?	?	−	−	+	+	+
Thirlwall 2013	+	+	−	−	+	+	?
Tillfors 2011	?	?	−	−	+	+	?
Treadwell 1996	?	?	−	−	−	?	−
Vigerland 2016	?	?	−	−	+	+	+
Waters 2009	?	?	−	+	+	+	+
Wergeland 2014	?	?	−	−	+	?	+
White 2013	+	+	−	+	+	+	+
Whiteside 2015	+	?	−	−	+	+	?
Wood 2006	+	+	−	−	+	+	+
Wood 2009	+	+	−	−	?	+	−
Wood 2015	+	+	−	+	+	+	+
Wuthrich 2012	+	?	−	−	+	+	+

© 2018 Zhou X et al. JAMA Psychiatry.

Risk of bias summary: it is a summary table of review authors' judgments for each risk of bias entriy for each study

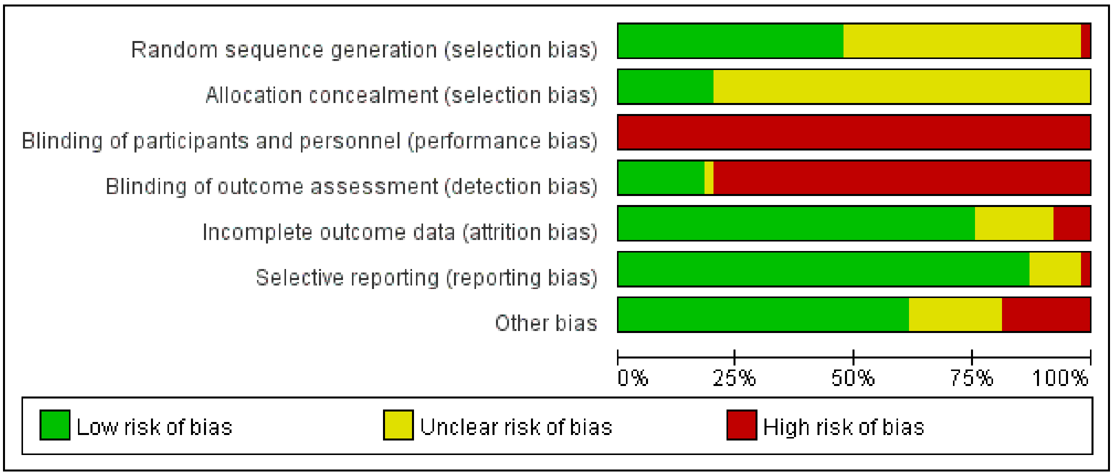

© 2018 Zhou X et al. JAMA Psychiatry.

eFigure 1. Network Plot for Each Outcome

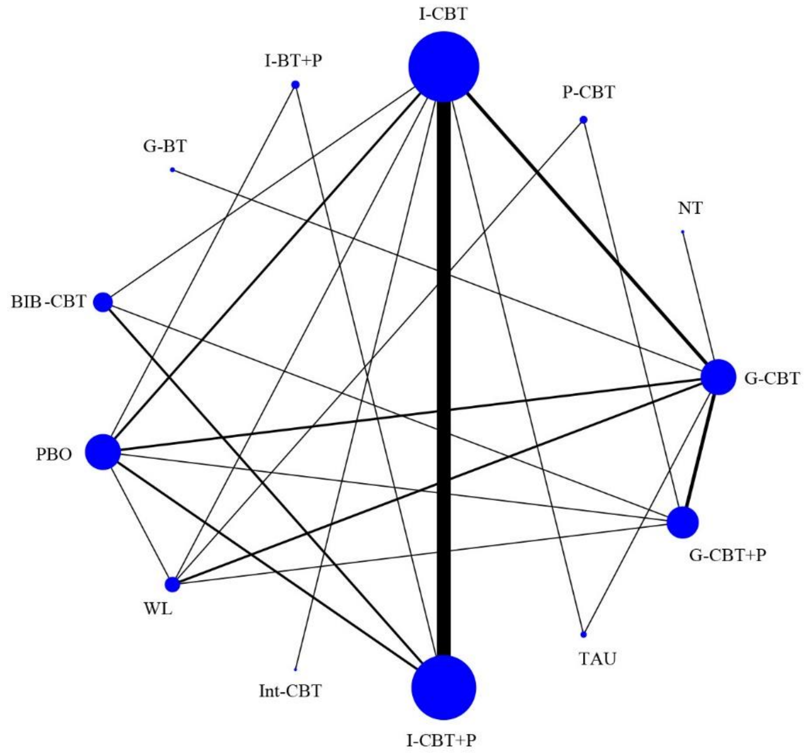

Network of eligible comparisons for mean overall change in symptoms at follow-up

© 2018 Zhou X et al. JAMA Psychiatry.

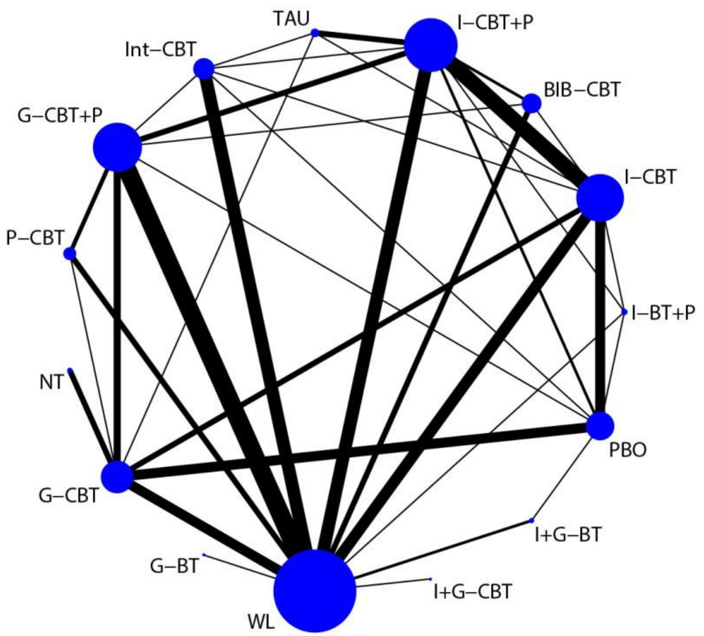

Network of eligible comparisons for acceptability (all-cause discontinuation)

© 2018 Zhou X et al. JAMA Psychiatry.

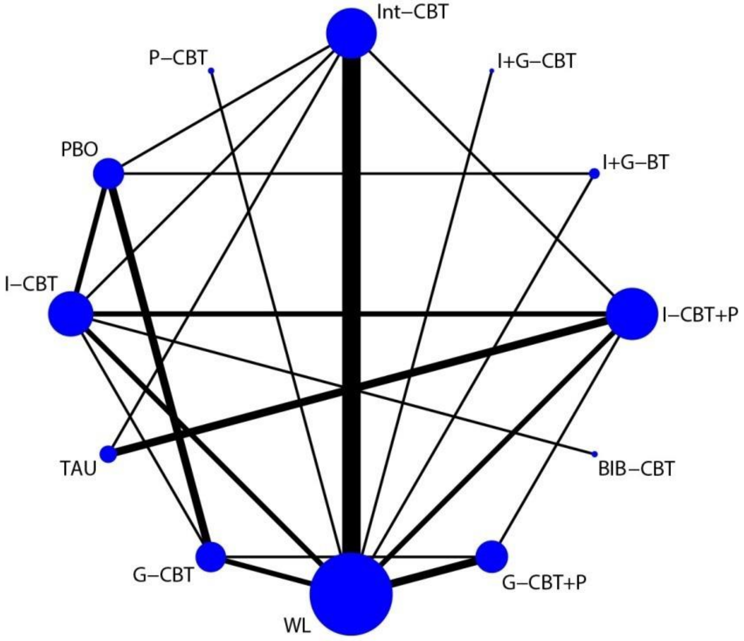

Network of eligible comparisons for mean overall change in quality of life and functional improvement

© 2018 Zhou X et al. JAMA Psychiatry.

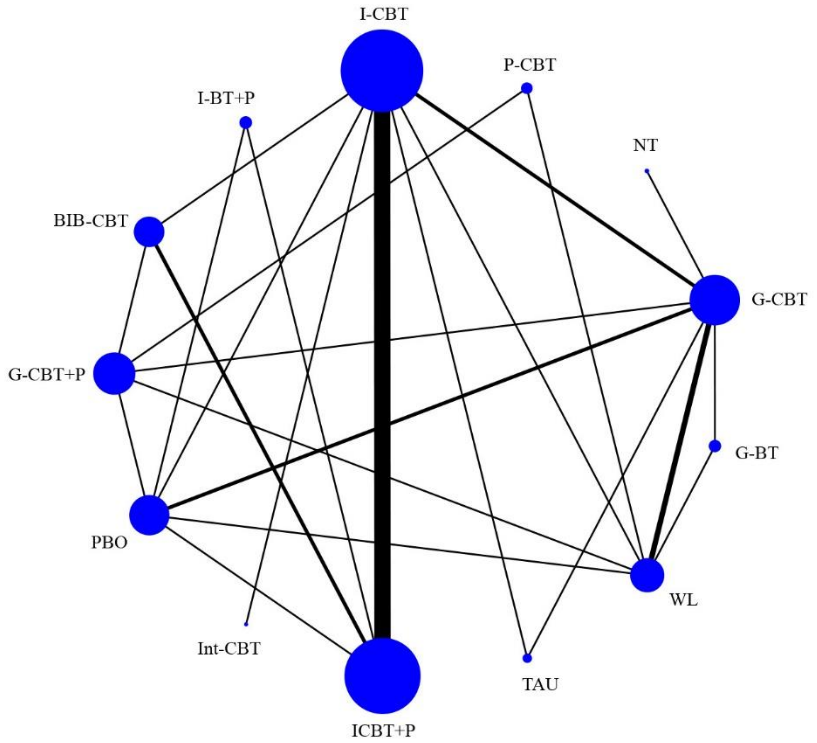

Network of eligible comparisons for mean overall change in symptoms at short-term follow-up (<6 months)

© 2018 Zhou X et al. JAMA Psychiatry.

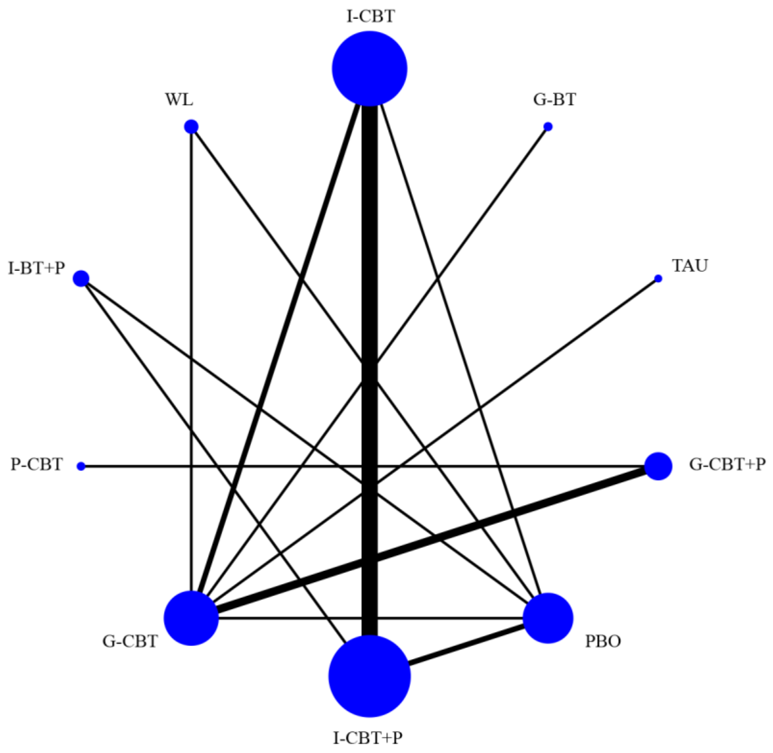

Network of eligible comparisons for mean overall change in symptoms at long-term follow-up (6-12 months)

Legend: BIB-CBT=bibliotherapy cognitive-behavioral therapy, G-BT=group behavioral therapy, G-CBT=group cognitive-behavioral therapy, G-CBT+P=group cognitive-behavioral therapy with parental involvement, I-BT+P=individual behavioral therapy with parental involvement, I-CBT=individual cognitive-behavioral therapy, I-CBT+P=individual cognitive-behavioral therapy with parental involvement, I+G-BT=individual and group behavioral therapy, I+G-CBT=individual and group cognitive-

© 2018 Zhou X et al. JAMA Psychiatry.

behavioral therapy, Int-CBT=internet-assisted cognitive-behavioral therapy, NT=no-treatment, PBO=psychological placebo, P-CBT=parent-only cognitive-behavioral therapy, TAU=treatment as usual, WL=waitlist.

© 2018 Zhou X et al. JAMA Psychiatry.

eResults. Results from Pairwise Meta-analysis for Each Outcome: Numbers, Estimates, and Heterogeneity

a. Summary numbers of studies and patients from pair-wise meta-analysis of direct comparisons

	Mean overall change in symptoms at post-treatment (N / n)*	Mean overall change in symptoms at follow-up (N / n)	All-cause discontinuation (N / n)	Mean overall change in quality of life and functional improvement (N / n)	Mean overall change in symptoms at short-term follow-up (N / n)	Mean overall change in symptoms at long-term follow-up (N / n)
**BIB-CBT vs.**						
G-CBT+P	1/180	1/180	1/180	NA	1/180	NA
I-CBT	1/48	1/48	1/48	1/48	1/48	NA
I-CBT+P	2/70	2/70	2/73	NA	2/70	NA
WL	4/453	NA	4/503	NA	NA	NA
**G-BT vs.**						
G-CBT	1/57	1/57	NA	NA	1/57	1/57
WL	2/89	NA	1/35	NA	1/54	NA
**G-CBT vs.**						
G-CBT+P	5/230	3/148	5/244	1/36	1/66	3/148
I-CBT	4/147	3/102	4/208	1/41	2/74	2/57
NT	4/118	1/30	4/129	NA	1/30	NA

© 2018 Zhou X et al. JAMA Psychiatry.

PBO	8/359	2/102	7/371	3/204	2/102	1/57
TAU	1/48	1/48	1/54	NA	1/48	1/48
WL	9/408	2/73	7/341	2/68	3/126	1/40
**G-CBT+P vs.**						
I-CBT+P	4/378	NA	4/386	1/78	NA	NA
Int-CBT	1/45	NA	1/49	NA	NA	NA
PBO	1/95	1/95	1/112	NA	1/95	NA
WL	13/948	1/24	13/987	3/260	1/24	NA
**I-BT+P vs.**						
I-CBT	1/12	NA	1/14	NA	NA	NA
I-CBT+P	1/65	1/65	1/81	NA	1/65	1/65
PBO	1/49	1/49	1/63	NA	1/49	1/49
WL	1/21	NA	1/21	NA	NA	NA
**I-CBT vs.**						
I-CBT+P	12/844	12/767	11/962	2/257	9/583	6/422
Int-CBT	1/33	1/26	1/33	1/33	1/26	NA

© 2018 Zhou X et al. JAMA Psychiatry.

PBO	7/344	2/129	7/387	2/70	1/46	1/83
TAU	1/32	1/32	1/32	NA	1/32	NA
WL	9/419	1/24	8/396	2/83	1/24	NA
**I-CBT+P vs.**						
Int-CBT	1/88	NA	1/88	1/88	NA	NA
PBO	2/154	2/128	2/170	NA	1/48	2/128
TAU	4/98	NA	4/104	3/86	NA	NA
WL	9/449	NA	9/492	2/108	NA	NA
**I+G-BT vs.**						
PBO	1/50	NA	1/67	1/50	NA	NA
WL	2/66	NA	2/67	1/55	NA	NA
**I+G-CBT vs.**						
WL	2/49	NA	1/30	1/30	NA	NA
**Int-CBT vs.**						
PBO	1/32	NA	1/32	1/32	NA	NA
TAU	1/100	NA	1/100	1/100	NA	NA

© 2018 Zhou X et al. JAMA Psychiatry.

WL	9/517	NA	9/568	7/344	NA	NA
**PBO vs.**						
WL	1/57	1/57	NA	NA	1/57	1/57
**P-CBT vs.**						
G-CBT	1/44	NA	1/44	NA	NA	NA
G-CBT+P	3/178	1/54	3/185	NA	1/53	1/54
WL	5/237	1/50	4/260	1/50	1/50	NA

* N= number of studies; n= number of patients;

BIB-CBT=bibliotherapy cognitive-behavioral therapy, G-BT=group behavioral therapy, G-CBT=group cognitive-behavioral therapy, G-CBT+P=group cognitive-behavioral therapy with parental involvement, I-BT+P=individual behavioral therapy with parental involvement, I-CBT=individual cognitive-behavioral therapy, I-CBT+P=individual cognitive-behavioral therapy with parental involvement, I+G-BT=individual and group behavioral therapy, I+G-CBT=individual and group cognitive-behavioral therapy, Int-CBT=internet-assisted cognitive-behavioral therapy, NA= not available, NT=no-treatment, PBO=psychological placebo, P-CBT=parent-only cognitive-behavioral therapy, TAU=treatment as usual, WL=waitlist.

© 2018 Zhou X et al. JAMA Psychiatry.

b. Summary estimates from pair-wise meta-analysis of direct comparisons*

	Mean overall change in symptoms at post-treatment SMD (95% CI)	Mean overall change in symptoms at follow-up SMD (95% CI)	All-cause discontinuation OR (95% CI)	Mean overall change in quality of life and functional improvement SMD (95% CI)	Mean overall change in symptoms at short-term follow-up SMD (95% CI)	Mean overall change in symptoms at long-term follow-up SMD (95% CI)
**BIB-CBT vs.**						
G-CBT+P	0.06 (-0.23 to 0.36)	0.02 (-0.28 to 0.31)	**2.58 (1.25 to 5.31)**	NA	0.02 (-0.28 to 0.31)	NA
I-CBT	0.29 (-0.28 to 0.86)	0.27 (-0.30 to 0.84)	1.40 (0.28 to 7.06)	-0.27 (-0.84 to 0.30)	0.27 (-0.30 to 0.84)	NA
I-CBT+P	-0.45 (-0.93 to 0.03)	-0.19 (-0.66 to 0.28)	2.15 (0.17 to 26.67)	NA	-0.19 (-0.66 to 0.28)	NA
WL	-0.37 (-0.90 to 0.17)	NA	**3.03 (1.72 to 5.32)**	NA	NA	NA
**G-BT vs.**						
G-CBT	-0.25 (-0.78 to 0.27)	0.32 (-0.20 to 0.84)	NA	NA	0.32 (-0.20 to 0.85)	0.32 (-0.20 to 0.84)
WL	-1.51 (-3.37 to 0.36)	NA	1.88 (0.31 to 11.37)	NA	**-1.91 (-2.56 to -1.26)**	NA
**G-CBT vs.**						
G-CBT+P	-0.12 (-0.38 to 0.14)	**0.56 (0.12 to 1.00)**	0.86 (0.20 to 3.72)	-0.45 (-1.11 to 0.22)	-0.25 (-0.73 to 0.24)	**0.56 (0.12 to 1.00)**
I-CBT	0.35 (-0.31 to 1.01)	0.46 (-0.19 to 1.11)	1.20 (0.36 to 3.95)	**-1.23 (-1.90 to -0.56)**	0.02 (-0.44 to 0.48)	**0.79 (0.24 to 1.34)**
NT	**-0.97 (-1.58 to -0.36)**	**-2.77 (-3.82 to -1.72)**	2.16 (0.52 to 8.95)	NA	**-2.77 (-3.82 to -1.72)**	NA
PBO	**-0.69 (-1.19 to -0.18)**	-0.92 (-2.30 to 0.47)	1.34 (0.77 to 2.34)	0.64 (-0.33 to 1.61)	-0.92 (-2.30 to 0.46)	**-1.63 (-2.25 to -1.01)**

© 2018 Zhou X et al. JAMA Psychiatry.

TAU	-0.01 (-0.59 to 0.56)	0.04 (-0.53 to 0.61)	1.67 (0.36 to 7.80)	NA	-0.03 (-0.60 to 0.54)	0.04 (-0.53 to 0.61)
WL	**-2.03 (-2.83 to -1.23)**	**-3.38 (-4.10 to -2.65)**	0.56 (0.28 to 1.13)	1.33 (-0.22 to 2.88)	**-3.00 (-3.60 to -2.39)**	**-3.30 (-4.26 to -2.33)**
**G-CBT+P vs.**						
I-CBT+P	0.04 (-0.16 to 0.24)	NA	0.78 (0.37 to 1.65)	**-0.53 (-0.98 to -0.08)**	NA	NA
Int-CBT	-0.26 (-0.85 to 0.33)	NA	1.25 (0.16 to 9.67)	NA	NA	NA
PBO	0.03 (-0.37 to 0.44)	-0.17 (-0.58 to 0.23)	1.01 (0.32 to 3.23)	NA	-0.17 (-0.58 to 0.23)	NA
WL	**-1.03 (-1.50 to -0.55)**	0.27 (-0.53 to 1.08)	1.08 (0.58 to 1.75)	**0.84 (0.57 to 1.11)**	0.27 (-0.53 to 1.08)	NA
**I-BT+P vs.**						
I-CBT	-0.97 (-2.18 to 0.24)	NA	1.00 (0.05 to 19.96)	NA	NA	NA
I-CBT+P	0.24 (-0.25 to 0.72)	-0.23 (-0.72 to 0.26)	0.75 (0.25 to 2.27)	NA	0.09 (-0.40 to 0.58)	-0.23 (-0.72 to 0.26)
PBO	-0.26 (-0.86 to 0.34)	-0.43 (-1.04 to 0.17)	0.49 (0.15 to 1.62)	NA	0.10 (-0.50 to 0.70)	-0.43 (-1.04 to 0.17)
WL	**-0.98 (-1.90 to -0.06)**	NA	NA	NA	NA	NA
**I-CBT vs.**						
I-CBT+P	-0.04 (-0.18 to 0.09)	0.06 (-0.12 to 0.24)	0.86 (0.50 to 1.45)	0.23 (-0.03 to 0.48)	0.07 (-0.14 to 0.29)	0.06 (-0.19 to 0.31)
Int-CBT	0.17 (-0.52 to 0.85)	0.19 (-0.58 to 0.96)	3.00 (0.11 to 79.14)	0.15 (-0.53 to 0.84)	0.19 (-0.58 to 0.96)	NA
PBO	**-0.41 (-0.73 to -0.09)**	-0.07 (-0.42 to 0.28)	0.97 (0.44 to 2.11)	**1.04 (0.54 to 1.55)**	-0.12 (-0.70 to 0.46)	-0.04 (-0.48 to 0.39)

© 2018 Zhou X et al. JAMA Psychiatry.

TAU	0.38 (-0.32 to 1.08)	0.08 (-0.61 to 0.78)	0.41 (0.03 to 5.00)	NA	0.08 (-0.61 to 0.78)	NA
WL	**-1.00 (-1.40 to -0.60)**	**-0.98 (-1.83 to -0.12)**	0.94 (0.37 to 2.35)	**0.88 (0.43 to 1.33)**	**-0.98 (-1.83 to -0.12)**	NA
**I-CBT+P vs.**						
Int-CBT	0.17 (-0.25 to 0.59)	NA	1.37 (0.29 to 6.50)	-0.03 (-0.44 to 0.39)	NA	NA
PBO	**-0.33 (-0.65 to -0.01)**	-0.01 (-0.37 to 0.35)	0.56 (0.26 to 1.22)	NA	0.01 (-0.60 to 0.61)	-0.01 (-0.37 to 0.35)
TAU	-0.51 (-1.28 to 0.26)	NA	2.96 (0.42 to 21.04)	**0.51 (0.08 to 0.94)**	NA	NA
WL	**-0.38 (-0.60 to -0.15)**	NA	1.31 (0.65 to 2.63)	0.71 (-0.29 to 1.70)	NA	NA
**I+G-BT vs.**						
PBO	0.09 (-0.47 to 0.66)	NA	0.36 (0.12 to 1.14)	**1.07 (0.47 to 1.68)**	NA	NA
WL	**-0.69 (-1.24 to -0.15)**	NA	5.39 (0.58 to 50.43)	0.43 (-0.11 to 0.97)	NA	NA
**I+G-CBT vs.**						
WL	-0.61 (-1.27 to 0.04)	NA	0.62 (0.09 to 4.34)	0.57 (-0.16 to 1.30)	NA	NA
**Int-CBT vs.**						
PBO	-0.43 (-1.13 to 0.27)	NA	0.12 (0.01 to 2.47)	**1.09 (0.35 to 1.84)**	NA	NA
TAU	-0.31 (-0.71 to 0.08)	NA	1.04 (0.25 to 4.43)	**0.65 (0.25 to 1.05)**	NA	NA
WL	**-0.38 (-0.57 to -0.18)**	NA	1.42 (0.75 to 2.69)	**0.70 (0.20 to 1.19)**	NA	NA

© 2018 Zhou X et al. JAMA Psychiatry.

**PBO vs.**						
WL	**-1.07 (-1.65 to -0.49)**	**-1.16 (-1.74 to -0.57)**	NA	NA	**-1.17 (-1.76 to -0.59)**	**-1.16 (-1.74 to -0.57)**
**P-CBT vs.**						
G-CBT	0.25 (-0.34 to 0.85)	NA	NA	NA	NA	NA
G-CBT+P	0.21 (-0.09 to 0.51)	-0.06 (-0.62 to 0.50)	**2.46 (1.10 to 5.46)**	NA	0.01 (-0.54 to 0.55)	-0.06 (-0.62 to 0.50)
WL	**-0.63 (-1.22 to -0.04)**	**-1.98 (-2.66 to -1.30)**	0.98 (0.42 to 2.27)	**1.91 (1.23 to 2.58)**	**-1.98 (-2.66 to -1.30)**	NA

Significant results are bolded and underscored. BIB-CBT=bibliotherapy cognitive-behavioral therapy, CI=confidence interval, G-BT=group behavioral therapy, G-CBT=group cognitive-behavioral therapy, G-CBT+P=group cognitive-behavioral therapy with parental involvement, I-BT+P=individual behavioral therapy with parental involvement, I-CBT=individual cognitive-behavioral therapy, I-CBT+P=individual cognitive-behavioral therapy with parental involvement, I+G-BT=individual and group behavioral therapy, I+G-CBT=individual and group cognitive-behavioral therapy, Int-CBT=internet-assisted cognitive-behavioral therapy, NA=not available, NT=no-treatment, OR=odds ratio, PBO=psychological placebo, P-CBT=parent-only cognitive-behavioral therapy, SMD=standardized mean difference, TAU=treatment as usual, WL=waitlist.

[*] DerSimonian R, Laird N. Metaanalysis in clinical trials. *Control Clin Trials* 1986; **7**: 177–87.

© 2018 Zhou X et al. JAMA Psychiatry.

c. Heterogeneity test result, I^2 and heterogeneity estimate

Mean overall change in symptoms at post-treatment

	No. of studies	P-value	I^2	τ^2
I-CBT vs WL*	9	0.0005	71.20%	0.2565
I-CBT vs G-CBT*	4	0.0092	73.90%	0.3324
G-CBT vs WL*	9	<0.0001	89.80%	1.3302
I-CBT vs I-CBT+P	12	0.5522	0.00%	0.0000
I-CBT vs PBO*	7	0.0753	47.60%	0.0843
I-CBT+P vs PBO	2	0.5002	0.00%	0.0000
I-CBT+P vs WL	9	0.2363	23.30%	0.0274
G-CBT vs G-CBT+P	5	0.6537	0.00%	0.0000
G-CBT+P vs WL*	13	<0.0001	90.44%	0.6613
G-CBT vs PBO*	8	0.0001	76.50%	0.3757
Int-CBT vs WL	9	0.3458	10.70%	0.0095
BIB-CBT vs WL*	4	0.0003	84.00%	0.2345
I-CBT+P vs TAU*	4	0.0398	64.00%	0.3686
I-CBT+P vs G-CBT+P	4	0.8503	0.00%	0.0000
I-CBT+P vs BIB-CBT	2	0.7463	0.00%	0.0000
G-CBT+P vs P-CBT	3	0.8841	0.00%	0.0000
I+G-CBT vs WL	2	0.2647	19.60%	0.0461
P-CBT vs WL*	5	0.0013	77.58%	0.3454
G-BT vs WL*	2	0.0002	92.90%	1.6872
G-CBT vs NT*	4	0.0702	57.47%	0.2237
I+G-BT vs WL	2	0.3044	5.20%	0.0148

* The comparisons between I-CBT and WL, between I-CBT and G-CBT, between G-CBT and WL, between I-CBT and PBO, between G-CBT+P and WL, between G-CBT and PBO, between BIB-CBT and WL, between I-CBT+P and TAU, between P-CBT and WL, between G-BT and WL, and between G-CBT and NT

© 2018 Zhou X et al. JAMA Psychiatry.

had higher I^2 values than the other comparisons.

Mean overall change in symptoms at follow-up

	No. of studies	P-value	I^2	τ^2
I-CBT vs G-CBT*	3	0.0806	60.30%	0.2004
I-CBT vs I-CBT+P	12	0.1507	30.16%	0.0290
I-CBT vs PBO	2	0.8425	0.00%	0.0000
I-CBT+P vs PBO	2	0.4542	0.00%	0.0000
G-CBT vs G-CBT+P	3	0.1902	39.80%	0.0604
I-CBT+P vs BIB-CBT	2	0.6578	0.00%	0.0000
G-CBT vs WL	2	0.8083	0.00%	0.0000
G-CBT vs PBO*	2	0.0012	90.40%	0.9016

* The comparisons between I-CBT and G-CBT, and between G-CBT and PBO had higher I^2 values than the other comparisons.

All-cause discontinuation

	No. of studies	P-value	I^2	τ^2
I-CBT vs WL*	7	0.0572	50.90%	0.7139
I-CBT vs G-CBT*	3	0.0932	57.90%	0.6243
G-CBT vs WL	7	0.8962	0.00%	0.0000
I-CBT vs I-CBT+P	10	0.1372	33.80%	0.2231
I-CBT vs PBO	6	0.0986	46.10%	0.4116
I-CBT+P vs PBO	2	0.7634	0.00%	0.0000
I-CBT+P vs WL	8	0.3251	13.40%	0.1369
G-CBT vs G-CBT+P*	4	0.0867	54.40%	1.2006
G-CBT+P vs WL	13	0.1692	27.29%	0.2458
G-CBT vs PBO	6	0.9342	0.00%	0.0000
Int-CBT vs WL	8	0.4536	0.00%	0.0000
BIB-CBT vs WL	3	0.9898	0.00%	0.0000

© 2018 Zhou X et al. JAMA Psychiatry.

I-CBT+P vs TAU	2	0.6931	0.00%	0.0000
I-CBT+P vs G-CBT+P	3	0.9175	0.00%	0.0000
G-CBT+P vs P-CBT	2	0.3845	0.00%	0.0000
P-CBT vs WL	4	0.3166	15.07%	0.1248
G-CBT vs NT	3	0.8450	0.00%	0.0000
I+G-BT vs WL	2	0.7452	0.00%	0.0000

* The comparisons between I-CBT and WL, between I-CBT and G-CBT, and between G-CBT and G-CBT+P had higher I^2 values than the other comparisons.

Mean overall change in quality of life and functional improvement

	No. of studies	P-value	I^2	τ^2
I-CBT vs WL	2	0.5498	0.00%	0.0000
I-CBT vs PBO	2	0.8755	0.00%	0.0000
Int-CBT vs WL*	7	0.0001	78.71%	0.3452
I-CBT+P vs WL*	2	0.0172	82.40%	0.4274
I-CBT+P vs TAU	3	0.7432	0.00%	0.0000
I-CBT vs I-CBT+P	2	0.4674	0.00%	0.0000
G-CBT+P vs WL	3	0.9837	0.00%	0.0000
G-CBT vs WL*	2	0.0046	87.50%	1.0968
G-CBT vs PBO*	3	0.0004	87.10%	0.6285

* The comparisons between Int-CBT and WL, between I-CBT+P and WL, between G-CBT and WL, and between G-CBT and PBO had higher I^2 values than the other comparisons.

© 2018 Zhou X et al. JAMA Psychiatry.

Mean overall change in symptoms at short-term follow-up

	No. of studies	P-value	I^2	τ^2
I-CBT vs G-CBT	2	0.5787	0.00%	0.0000
I-CBT vs I-CBT+P	9	0.1617	32.00%	0.0321
I-CBT+P vs BIB-CBT	2	0.6578	0.00%	0.0000
G-CBT vs WL	3	0.2637	25.00%	0.0737
G-CBT vs PBO*	2	0.0013	90.40%	0.8962

* The comparisons between G-CBT and PBO had higher I^2 values than the other comparisons.

Mean overall change in symptoms at long-term follow-up

	No. of studies	P-value	I^2	τ^2
I-CBT vs G-CBT	2	0.4756	0.00%	0.0000
I-CBT vs I-CBT+P	6	0.1737	35.00%	0.0331
I-CBT+P vs PBO	2	0.4542	0.00%	0.0000
G-CBT vs G-CBT+P	3	0.1902	39.80%	0.0604

Abbreviation: BIB-CBT=bibliotherapy cognitive-behavioral therapy, G-BT=group behavioral therapy, G-CBT=group cognitive-behavioral therapy, G-CBT+P=group cognitive-behavioral therapy with parental involvement, I-BT+P=individual behavioral therapy with parental involvement, I-CBT=individual cognitive-behavioral therapy, I-CBT+P=individual cognitive-behavioral therapy with parental involvement, I+G-BT=individual and group behavioral therapy, I+G-CBT=individual and group cognitive-behavioral therapy, Int-CBT=internet-assisted cognitive-behavioral therapy, NT=no-treatment, PBO=psychological placebo, P-CBT=parent-only cognitive-behavioral therapy, TAU=treatment as usual, WL=waitlist.

© 2018 Zhou X et al. JAMA Psychiatry.

eFigure 2. The Forest Plots of Network Meta-analysis Results for Efficacy Post Treatment and End of Follow-up

The forest plot of network meta-analysis result for efficacy at post-treatment

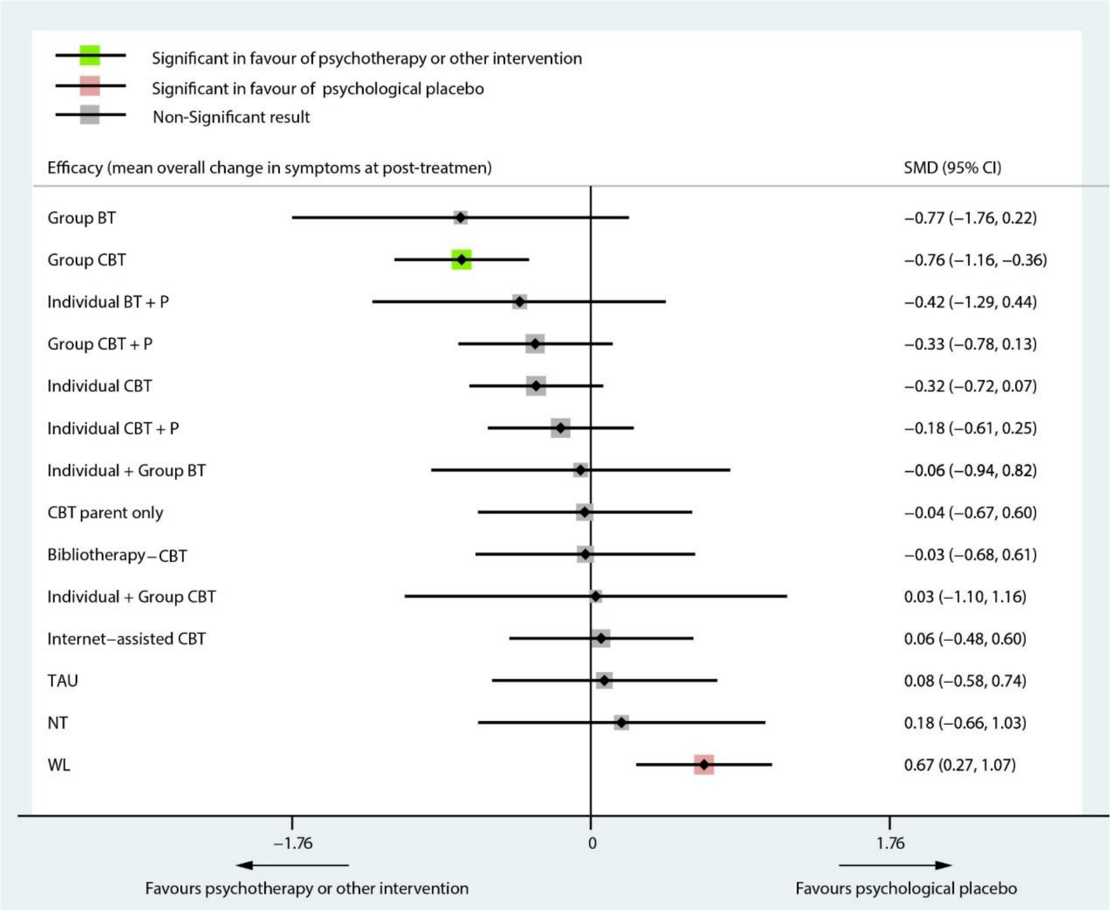

© 2018 Zhou X et al. JAMA Psychiatry.

The forest plot of network meta-analysis result for efficacy at end of follow-up

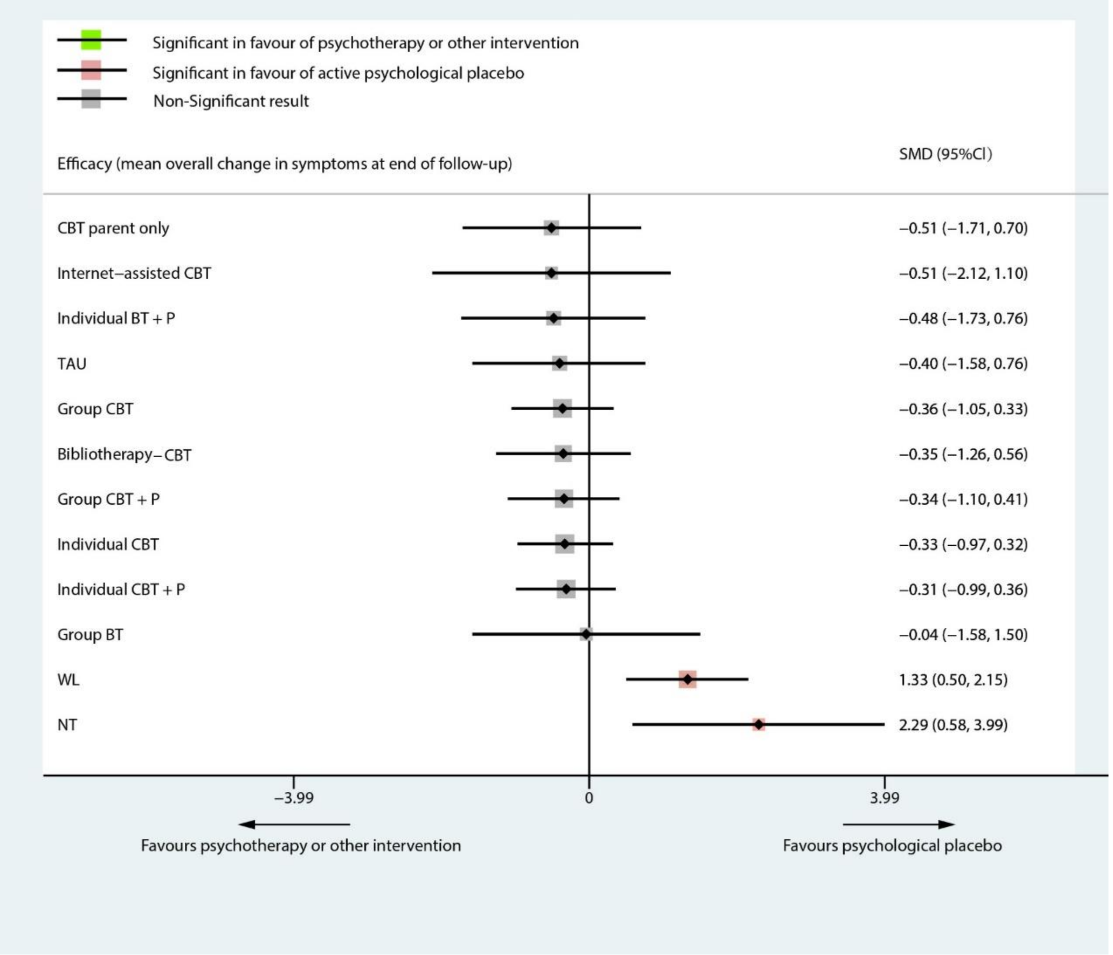

© 2018 Zhou X et al. JAMA Psychiatry.

eFigure 3. The Results of Network Meta-analysis for Secondary Outcomes

G-CBT	0.04 (-1.34 to 1.42)	-0.32 (-1.68 to 1.04)	…	0.62 (-0.97 to 2.21)	0.08 (-0.78 to 0.97)	0.05 (-0.91 to 1.03)	…	0.10 (-1.37 to 1.58)	0.56 (-0.24 to 1.35)	-0.51 (-1.49 to 0.49)	**-2.20 (-3.48 to -0.94)**	…
-0.25 (-1.19 to 0.69)	TAU	-0.36 (-2.31 to 1.57)	…	0.58 (-1.53 to 2.68)	0.04 (-1.58 to 1.69)	0.01 (-1.67 to 1.70)	…	0.06 (-1.95 to 2.10)	0.52 (-1.08 to 2.11)	-0.55 (-2.24 to 1.15)	**-2.24 (-4.12 to -0.38)**	…
-0.27 (-1.37 to 0.83)	-0.02 (-1.45 to 1.42)	G-BT	…	0.94 (-1.16 to 3.04)	0.40 (-1.21 to 2.03)	0.36 (-1.30 to 2.06)	…	0.42 (-1.57 to 2.43)	0.88 (-0.70 to 2.45)	-0.19 (-1.87 to 1.50)	**-1.88 (-3.76 to -0.03)**	…
-0.39 (-1.86 to 1.07)	-0.14 (-1.78 to 1.48)	-0.12 (-1.92 to 1.68)	Int-CBT	…	…	…	…	…	…	…	…	…
-0.53 (-1.56 to 0.48)	-0.28 (-1.63 to 1.06)	-0.26 (-1.68 to 1.14)	-0.14 (-1.84 to 1.56)	P-CBT	-0.54 (-2.34 to 1.30)	-0.57 (-2.42 to 1.32)	…	-0.52 (-2.68 to 1.68)	-0.06 (-1.44 to 1.32)	-1.13 (-3.00 to 0.75)	**-2.82 (-4.85 to -0.77)**	…
-0.57 (-1.20 to 0.06)	-0.32 (-1.27 to 0.63)	-0.30 (-1.52 to 0.92)	-0.18 (-1.50 to 1.14)	-0.04 (-1.12 to 1.04)	I-CBT	-0.03 (-0.57 to 0.51)	…	0.03 (-1.30 to 1.32)	0.48 (-0.72 to 1.65)	-0.58 (-1.44 to 0.25)	**-2.28 (-3.67 to -0.95)**	…
-0.61 (-1.31 to 0.09)	-0.36 (-1.37 to 0.65)	-0.34 (-1.60 to 0.91)	-0.22 (-1.59 to 1.16)	-0.08 (-1.19 to 1.03)	-0.03 (-0.42 to 0.35)	I-CBT+P	…	0.06 (-1.20 to 1.29)	0.51 (-0.76 to 1.75)	-0.55 (-1.39 to 0.26)	**-2.25 (-3.66 to -0.89)**	…
-0.64 (-1.48 to 0.20)	-0.39 (-1.53 to 0.74)	-0.37 (-1.71 to 0.95)	-0.25 (-1.74 to 1.24)	-0.11 (-1.27 to 1.06)	-0.07 (-0.76 to 0.62)	-0.04 (-0.71 to 0.64)	BIB-CBT	…	…	…	…	…
-0.81 (-2.01 to 0.38)	-0.56 (-2.00 to 0.87)	-0.54 (-2.13 to 1.04)	-0.42 (-2.16 to 1.32)	-0.28 (-1.75 to 1.19)	-0.24 (-1.37 to 0.88)	-0.21 (-1.30 to 0.89)	-0.17 (-1.42 to 1.08)	I-BT+P	0.46 (-1.23 to 2.13)	-0.61 (-1.86 to 0.65)	**-2.31 (-4.05 to -0.60)**	…
**-0.82 (-1.53 to -0.14)**	-0.57 (-1.69 to 0.52)	-0.55 (-1.80 to 0.67)	-0.43 (-1.96 to 1.09)	-0.29 (-1.23 to 0.63)	-0.25 (-1.02 to 0.49)	-0.22 (-1.01 to 0.56)	-0.18 (-1.00 to 0.62)	-0.01 (-1.26 to 1.23)	G-CBT+P	-1.07 (-2.33 to 0.22)	**-2.76 (-4.26 to -1.27)**	…
**-0.86 (-1.54 to -0.20)**	-0.61 (-1.69 to 0.46)	-0.59 (-1.83 to 0.64)	-0.47 (-1.97 to 1.01)	-0.33 (-1.41 to 0.75)	-0.29 (-0.99 to 0.40)	-0.26 (-0.98 to 0.46)	-0.22 (-1.09 to 0.65)	-0.05 (-1.17 to 1.06)	-0.04 (-0.77 to 0.71)	PBO	**-1.70 (-2.97 to -0.46)**	…
**-2.17 (-2.75 to -1.57)**	**-1.92 (-2.97 to -0.85)**	**-1.90 (-3.00 to -0.80)**	**-1.78 (-3.26 to -0.27)**	**-1.64 (-2.57 to -0.69)**	**-1.59 (-2.30 to -0.88)**	**-1.56 (-2.32 to -0.79)**	**-1.52 (-2.40 to -0.64)**	**-1.35 (-2.58 to -0.12)**	**-1.34 (-2.04 to -0.62)**	**-1.30 (-2.02 to -0.57)**	WL	…
**-2.65 (-4.07 to -1.23)**	**-2.40 (-4.11 to -0.70)**	**-2.39 (-4.18 to -0.59)**	**-2.26 (-4.31 to -0.22)**	**-2.12 (-3.86 to -0.38)**	**-2.08 (-3.63 to -0.53)**	**-2.05 (-3.62 to -0.46)**	**-2.01 (-3.66 to -0.36)**	-1.84 (-3.69 to 0.02)	**-1.83 (-3.40 to -0.24)**	**-1.79 (-3.35 to -0.22)**	-0.49 (-2.02 to 1.05)	NT

Relative effect sizes of efficacy at short-term follow-up and long-term follow-up according to network meta-analysis.

© 2018 Zhou X et al. JAMA Psychiatry.

Treatment	Efficacy (mean overall change in symptoms at short-term follow-up, SMD [95% Crl])	Efficacy (mean overall change in symptoms at long-term follow-up, SMD [95% Crl])

Comparisons between treatments should be read from left to right, and the estimate is in the cell in common between the column-defining treatment and the row-defining treatment. For efficacy in short-term follow-up, standardized mean differences (SMDs) less than 0 favor the column-defining treatment. For efficacy in long-term follow-up, SMDs lower than 0 favor the row-defining treatment. To obtain SMDs for comparisons in the opposite direction, negative values should be converted into positive values, and vice versa. Significant results are in bold and underlined. BIB-CBT=bibliotherapy cognitive-behavioral therapy, G-BT=group behavioral therapy, G-CBT=group cognitive-behavioral therapy, G-CBT+P=group cognitive-behavioral therapy with parental involvement, I-BT+P=individual behavioral therapy with parental involvement, I-CBT=individual cognitive-behavioral therapy, I-CBT+P=individual cognitive-behavioral therapy with parental involvement, I+G-BT=individual and group behavioral therapy, I+G-CBT=individual and group cognitive-behavioral therapy, Int-CBT=internet-assisted cognitive-behavioral therapy, NT=no-treatment, PBO=psychological placebo, P-CBT=parent-only cognitive-behavioral therapy, TAU=treatment as usual, WL=waitlist.

© 2018 Zhou X et al. JAMA Psychiatry.

eFigure 4. Assessment of Inconsistency Results for Each Outcome: Global, Local, and From the Node-Splitting Model

a. Evaluation of the global inconsistency

For evaluating the global inconsistency, we present the mean posterior deviance (D), the number of data points and the Deviance Information Criterion (DIC) of the NMA model. The mean posterior deviance should approximate the number of data points for models with good fit to the data. The DIC is a Bayesian model evaluation criterion that measures model fit adjusted with complexity of the model; smaller DIC values correspond to more preferable models.

Model assumption	D	# of data points	DIC
Mean overall change in symptoms at post-treatment [Test of global inconsistency: P = 0.4962]			
Consistency	223.10	220	1072.65
Inconsistency	224.20	220	1077.40
Mean overall change in symptoms at follow-up [Test of global inconsistency: P < 0.0001]			
Consistency	76.13	74	387.594
Inconsistency	76.16	74	381.472
All-cause discontinuation [Test of global inconsistency: P = 0.5742]			
Consistency	196.30	206	881.184
Inconsistency	206.30	206	900.451
Mean overall change in quality of life and functional improvement [Test of global inconsistency: P = 0.1109]			
Consistency	64.53	64	246.426
Inconsistency	64.33	64	248.643
Mean overall change in symptoms at short-term follow-up [Test of global inconsistency: P < 0.0001]			
Consistency	64.29	62	327.363
Inconsistency	63.67	62	324.606
Mean overall change in symptoms at long-term follow-up [Test of global inconsistency: P < 0.0001]			
Consistency	35.58	35	160.525
Inconsistency	35.20	35	157.542

© 2018 Zhou X et al. JAMA Psychiatry.

b. Evaluation of the local inconsistency

Tests of local inconsistency revealed that the percentages for inconsistent loops were to be expected according to empirical data with the methods of Veroniki et al (Int J Epidemiol 2013; 42:332 vs 45).

Mean overall change in symptoms at post-treatment

Loop	IF	z-value	P-value	95%CI	τ^2
G-CBT vs I-CBT+P vs TAU vs WL	2.108	2.0210	0.0433	(0.06,4.15)	0.5007
G-CBT vs Int-CBT vs TAU vs WL	1.874	1.5573	0.1194	(0.00,4.23)	0.4633
G-CBT vs I-CBT vs WL	1.301	1.9629	0.0497	(0.00,2.60)	0.6660
I-BT+P vs I-CBT vs I-CBT+P	1.247	1.8651	0.0622	(0.00,2.56)	0.0000
P-CBT vs G-CBT vs WL	1.141	0.9828	0.3257	(0.00,3.42)	0.9053
Int-CBT vs PBO vs WL	1.123	2.2697	0.0232	(0.15,2.09)	0.0095
I-BT+P vs I-CBT vs PBO	1.115	1.3171	0.1878	(0.00,2.77)	0.0843
I-CBT+P vs PBO vs WL	1.041	2.6400	0.0083	(0.27,1.81)	0.0182
I-BT+P vs I-CBT vs WL	0.983	0.8071	0.4196	(0.00,3.37)	0.2565
G-CBT vs G-CBT+P vs WL	0.976	1.3833	0.1666	(0.00,2.36)	0.6742
G-CBT vs I-CBT vs PBO	0.924	2.1608	0.0307	(0.09,1.76)	0.1812
G-CBT vs G-CBT+P vs I-CBT vs Int-CBT	0.882	1.2130	0.2251	(0.00,2.31)	0.0999
I-BT+P vs I-CBT+P vs WL	0.841	1.4209	0.1553	(0.00,2.00)	0.0274
G-CBT vs I-CBT+P vs PBO vs TAU	0.785	0.7914	0.4287	(0.00,2.73)	0.3064
G-BT vs G-CBT vs WL	0.758	0.4576	0.6472	(0.00,4.01)	1.5935
G-CBT vs I-CBT vs TAU	0.747	0.7506	0.4529	(0.00,2.70)	0.3324
G-CBT+P vs Int-CBT vs PBO	0.727	1.4214	0.1552	(0.00,1.73)	0.0000
I-CBT vs Int-CBT vs WL	0.714	1.2154	0.2242	(0.00,1.87)	0.1166
I-CBT vs I-CBT+P vs TAU	0.712	1.5095	0.1312	(0.00,1.64)	0.0209
G-CBT+P vs I-CBT+P vs WL	0.707	1.2704	0.2039	(0.00,1.80)	0.3534
BIB-CBT vs I-CBT vs I-CBT+P	0.692	1.7992	0.0720	(0.00,1.45)	0.0000
G-CBT vs G-CBT+P vs Int-CBT vs TAU	0.681	1.4090	0.1588	(0.00,1.63)	0.0000
BIB-CBT vs G-CBT+P vs I-CBT vs Int-CBT	0.652	1.1539	0.2485	(0.00,1.76)	0.0000
BIB-CBT vs G-CBT+P vs WL	0.640	0.5298	0.5963	(0.00,3.01)	0.5442
G-CBT vs G-CBT+P vs PBO	0.617	0.9837	0.3253	(0.00,1.85)	0.2027
BIB-CBT vs G-CBT+P vs I-CBT+P	0.553	1.8262	0.0678	(0.00,1.15)	0.0000
G-CBT vs Int-CBT vs PBO vs TAU	0.551	0.4344	0.6640	(0.00,3.04)	0.3757
I-CBT vs Int-CBT vs TAU	0.525	0.9753	0.3294	(0.00,1.58)	0.0000
BIB-CBT vs I-CBT+P vs WL	0.516	1.2257	0.2203	(0.00,1.34)	0.1045
I-CBT vs PBO vs WL	0.510	0.7950	0.4266	(0.00,1.77)	0.1780
I-CBT vs I-CBT+P vs WL	0.502	2.1248	0.0336	(0.04,0.96)	0.0636
G-CBT+P vs I-CBT+P vs Int-CBT	0.471	1.2294	0.2189	(0.00,1.22)	0.0000
G-CBT vs G-CBT+P vs I-CBT vs I-CBT+P	0.396	1.4594	0.1444	(0.00,0.93)	0.0111
I-CBT+P vs Int-CBT vs TAU	0.370	0.3024	0.7623	(0.00,2.77)	0.3686

© 2018 Zhou X et al. JAMA Psychiatry.

G-CBT vs G-CBT+P vs I-CBT+P vs TAU	0.351	0.7915	0.4287	(0.00,1.22)	0.0127
I-BT+P vs PBO vs WL	0.349	0.5516	0.5813	(0.00,1.59)	0.0000
BIB-CBT vs I-CBT vs WL	0.341	0.4428	0.6579	(0.00,1.85)	0.2463
G-CBT+P vs I-CBT+P vs PBO	0.322	1.1376	0.2553	(0.00,0.88)	0.0000
G-CBT+P vs Int-CBT vs WL	0.319	0.3073	0.7586	(0.00,2.35)	0.4406
I+G-BT vs PBO vs WL	0.300	0.6146	0.5388	(0.00,1.25)	0.0000
I-BT+P vs I-CBT+P vs PBO	0.238	0.5416	0.5881	(0.00,1.10)	0.0000
BIB-CBT vs G-CBT vs G-CBT+P vs I-CBT	0.230	0.3559	0.7219	(0.00,1.50)	0.0999
BIB-CBT vs G-CBT+P vs I-CBT vs PBO	0.219	0.3236	0.7463	(0.00,1.55)	0.0843
I-CBT+P vs Int-CBT vs WL	0.215	0.7397	0.4595	(0.00,0.78)	0.0143
I-CBT vs Int-CBT vs PBO	0.171	0.2286	0.8192	(0.00,1.64)	0.1137
P-CBT vs G-CBT vs G-CBT+P	0.119	0.3222	0.7473	(0.00,0.84)	0.0000
P-CBT vs G-CBT+P vs WL	0.119	0.1465	0.8835	(0.00,1.70)	0.5207
G-CBT vs PBO vs WL	0.105	0.0988	0.9213	(0.00,2.19)	0.7826
I-CBT+P vs Int-CBT vs PBO	0.067	0.1502	0.8806	(0.00,0.95)	0.0000
I-CBT vs I-CBT+P vs Int-CBT	0.041	0.0994	0.9209	(0.00,0.85)	0.0000
I-CBT vs I-CBT+P vs PBO	0.017	0.0675	0.9462	(0.00,0.51)	0.0197
G-CBT+P vs PBO vs WL	0.013	0.0076	0.9940	(0.00,3.33)	0.6613

© 2018 Zhou X et al. JAMA Psychiatry.

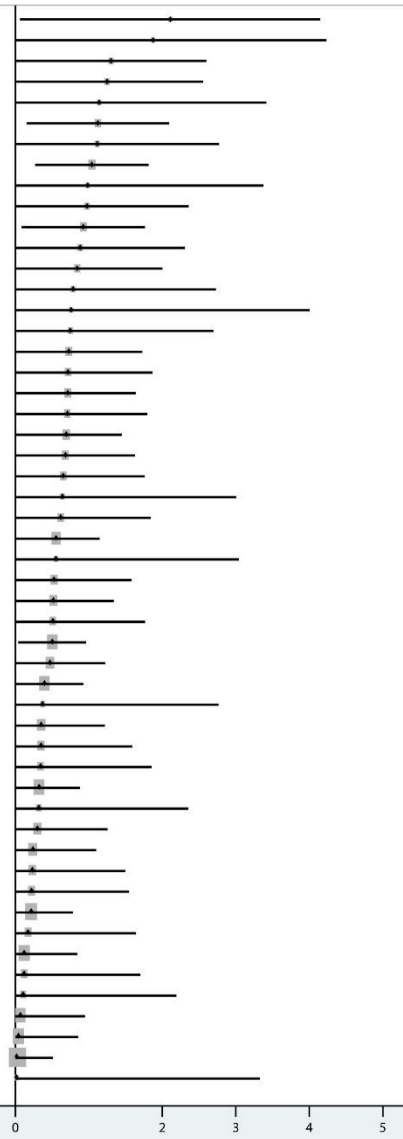

Loop	IF	95%CI (truncated)	Loop−specific Heterogeneity(τ^2)
G-CBT vs I-CBT+P vs TAU vs WL	2.108	(0.06,4.15)	0.5007
G-CBT vs Int-CBT vs TAU vs WL	1.874	(0.00,4.23)	0.4633
G-CBT vs I-CBT vs WL	1.301	(0.00,2.60)	0.6660
I-BT+P vs I-CBT vs I-CBT+P	1.247	(0.00,2.56)	0.0000
P-CBT vs G-CBT vs WL	1.141	(0.00,3.42)	0.9053
Int-CBT vs PBO vs WL	1.123	(0.15,2.09)	0.0095
I-BT+P vs I-CBT vs PBO	1.115	(0.00,2.77)	0.0843
I-CBT+P vs PBO vs WL	1.041	(0.27,1.81)	0.0182
I-BT+P vs I-CBT vs WL	0.983	(0.00,3.37)	0.2565
G-CBT vs G-CBT+P vs WL	0.976	(0.00,2.36)	0.6742
G-CBT vs I-CBT vs PBO	0.924	(0.09,1.76)	0.1812
G-CBT vs G-CBT+P vs I-CBT vs Int-CBT	0.882	(0.00,2.31)	0.0999
I-BT+P vs I-CBT+P vs WL	0.841	(0.00,2.00)	0.0274
G-CBT vs I-CBT+P vs PBO vs TAU	0.785	(0.00,2.73)	0.3064
G-BT vs G-CBT vs WL	0.758	(0.00,4.01)	1.5935
G-CBT vs I-CBT vs TAU	0.747	(0.00,2.70)	0.3324
G-CBT+P vs Int-CBT vs PBO	0.727	(0.00,1.73)	0.0000
I-CBT vs Int-CBT vs WL	0.714	(0.00,1.87)	0.1166
I-CBT vs I-CBT+P vs TAU	0.712	(0.00,1.64)	0.0209
G-CBT+P vs I-CBT+P vs WL	0.707	(0.00,1.80)	0.3534
BIB-CBT vs I-CBT vs I-CBT+P	0.692	(0.00,1.45)	0.0000
G-CBT vs G-CBT+P vs Int-CBT vs TAU	0.681	(0.00,1.63)	0.0000
BIB-CBT vs G-CBT+P vs I-CBT vs Int-CBT	0.652	(0.00,1.76)	0.0000
BIB-CBT vs G-CBT+P vs WL	0.640	(0.00,3.01)	0.5442
G-CBT vs G-CBT+P vs PBO	0.617	(0.00,1.85)	0.2027
BIB-CBT vs G-CBT+P vs I-CBT+P	0.553	(0.00,1.15)	0.0000
G-CBT vs Int-CBT vs PBO vs TAU	0.551	(0.00,3.04)	0.3757
I-CBT vs Int-CBT vs TAU	0.525	(0.00,1.58)	0.0000
BIB-CBT vs I-CBT+P vs WL	0.516	(0.00,1.34)	0.1045
I-CBT vs PBO vs WL	0.510	(0.00,1.77)	0.1780
I-CBT vs I-CBT+P vs WL	0.502	(0.04,0.96)	0.0636
G-CBT+P vs I-CBT+P vs Int-CBT	0.471	(0.00,1.22)	0.0000
G-CBT vs G-CBT+P vs I-CBT vs I-CBT+P	0.396	(0.00,0.93)	0.0111
I-CBT+P vs Int-CBT vs TAU	0.370	(0.00,2.77)	0.3686
G-CBT vs G-CBT+P vs I-CBT+P vs TAU	0.351	(0.00,1.22)	0.0127
I-BT+P vs PBO vs WL	0.349	(0.00,1.59)	0.0000
BIB-CBT vs I-CBT vs WL	0.341	(0.00,1.85)	0.2463
G-CBT+P vs I-CBT+P vs PBO	0.322	(0.00,0.88)	0.0000
G-CBT+P vs Int-CBT vs WL	0.319	(0.00,2.35)	0.4406
I+G-BT vs PBO vs WL	0.300	(0.00,1.25)	0.0000
I-BT+P vs I-CBT+P vs PBO	0.238	(0.00,1.10)	0.0000
BIB-CBT vs G-CBT vs G-CBT+P vs I-CBT	0.230	(0.00,1.50)	0.0999
BIB-CBT vs G-CBT+P vs I-CBT vs PBO	0.219	(0.00,1.55)	0.0843
I-CBT+P vs Int-CBT vs WL	0.215	(0.00,0.78)	0.0143
I-CBT vs Int-CBT vs PBO	0.171	(0.00,1.64)	0.1137
P-CBT vs G-CBT vs G-CBT+P	0.119	(0.00,0.84)	0.0000
P-CBT vs G-CBT+P vs WL	0.119	(0.00,1.70)	0.5207
G-CBT vs PBO vs WL	0.105	(0.00,2.19)	0.7826
I-CBT+P vs Int-CBT vs PBO	0.067	(0.00,0.95)	0.0000
I-CBT vs I-CBT+P vs Int-CBT	0.041	(0.00,0.85)	0.0000
I-CBT vs I-CBT+P vs PBO	0.017	(0.00,0.51)	0.0197
G-CBT+P vs PBO vs WL	0.013	(0.00,3.33)	0.6613

© 2018 Zhou X et al. JAMA Psychiatry.

Mean overall change in symptoms at follow-up

Loop	IF	z-value	P-value	95%CI	τ^2
WL vs G-CBT vs G-CBT+P	4.194	6.953	<0.0001	(3.01,5.38)	0.0149
WL vs G-CBT vs I-CBT	2.846	3.752	0.0002	(1.36,4.33)	0.1119
WL vs P-CBT vs G-CBT+P	2.192	3.594	0.0003	(1.00,3.39)	0.0000
G-CBT vs I-CBT vs PBO	1.631	2.193	0.0283	(0.17,3.09)	0.2551
WL vs G-CBT+P vs PBO	1.603	2.921	0.0035	(0.53,2.68)	0.0000
WL vs G-CBT vs PBO	1.442	2.142	0.0322	(0.12,2.76)	0.0000
G-CBT vs G-CBT+P vs PBO	1.311	1.532	0.1256	(0.00,2.99)	0.3334
WL vs BIB-CBT vs G-CBT+P vs I-CBT	0.997	1.463	0.1433	(0.00,2.33)	0.0000
BIB-CBT vs I-CBT vs I-CBT+P	0.525	1.203	0.2291	(0.00,1.38)	0.0230
G-CBT vs I-CBT vs TAU	0.497	0.585	0.5583	(0.00,2.16)	0.2004
BIB-CBT vs G-CBT vs G-CBT+P vs I-CBT	0.371	0.528	0.5975	(0.00,1.75)	0.1178
BIB-CBT vs G-CBT+P vs I-CBT vs PBO	0.353	0.831	0.4063	(0.00,1.19)	0.0000
I-BT+P vs I-CBT+P vs PBO	0.292	0.640	0.5217	(0.00,1.19)	0.0000
WL vs I-CBT vs PBO	0.254	0.456	0.6482	(0.00,1.34)	0.0000
I-CBT vs I-CBT+P vs PBO	0.141	0.450	0.6525	(0.00,0.75)	0.0205
BIB-CBT vs G-CBT+P vs I-CBT+P vs PBO	0.046	0.115	0.9082	(0.00,0.82)	0.0000

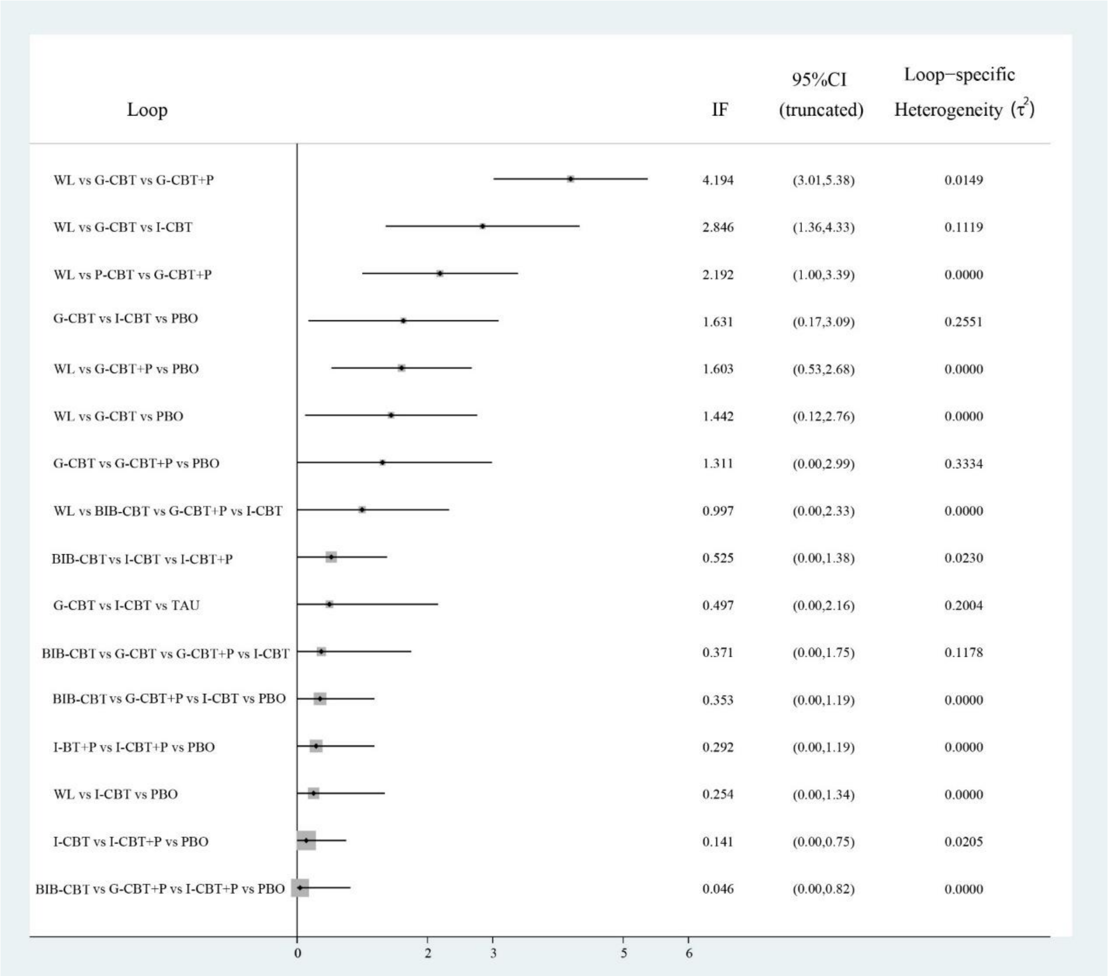

© 2018 Zhou X et al. JAMA Psychiatry.

All-cause discontinuation

Loop	ROR	z-value	P-value	95%CI	τ^2
G-CBT vs I+G-BT vs PBO vs WL	35.443	2.624	0.0087	(2.47,509.39)	0.0000
G-CBT vs Int-CBT vs PBO vs WL	29.153	2.041	0.0413	(1.14,744.01)	0.0000
I+G-BT vs I-CBT vs PBO vs WL	15.627	1.622	0.1048	(1.00,432.94)	0.4906
G-CBT+P vs I+G-BT vs PBO vs WL	14.965	1.681	0.0928	(1.00,350.83)	0.1902
I-CBT vs Int-CBT vs TAU	7.713	0.916	0.3595	(1.00,609.53)	0.0000
G-CBT vs Int-CBT vs PBO vs TAU	7.207	1.032	0.3022	(1.00,306.92)	0.0000
G-CBT+P vs Int-CBT vs PBO	6.931	0.985	0.3245	(1.00,326.01)	0.0000
I+G-BT vs I-CBT+P vs PBO vs WL	6.484	1.354	0.1757	(1.00,97.00)	0.0000
I-CBT vs I-CBT+P vs TAU	6.269	1.064	0.2873	(1.00,184.35)	0.1765
G-CBT vs I-CBT+P vs PBO vs WL	5.467	2.482	0.0131	(1.43,20.90)	0.0000
I-CBT vs Int-CBT vs WL	4.373	0.789	0.4302	(1.00,170.95)	0.3603
G-CBT vs I-CBT+P vs PBO vs TAU	4.236	1.059	0.2894	(1.00,61.21)	0.0000
G-CBT vs Int-CBT vs TAU vs WL	4.045	1.183	0.2368	(1.00,40.98)	0.0000
I-CBT+P vs Int-CBT vs PBO	3.526	0.704	0.4817	(1.00,118.02)	0.0000
G-CBT vs I-CBT vs TAU	3.415	0.585	0.5584	(1.00,208.92)	0.6145
G-CBT vs G-CBT+P vs I-CBT vs Int-CBT	3.179	0.422	0.6733	(1.00,687.42)	0.8379
I-CBT vs Int-CBT vs PBO	3.118	0.443	0.6576	(1.00,475.74)	0.4436
I-CBT vs I-CBT+P vs Int-CBT	2.567	0.475	0.6346	(1.00,125.43)	0.2216
BIB-CBT vs G-CBT vs G-CBT+P vs I-CBT	2.442	0.449	0.6538	(1.00,120.76)	0.8379
BIB-CBT vs I-CBT vs WL	2.418	0.728	0.4665	(1.00,26.02)	0.2769
P-CBT vs G-CBT+P vs WL	2.363	1.194	0.2324	(1.00,9.69)	0.1432
G-CBT+P vs I-CBT+P vs PBO	2.314	1.038	0.2994	(1.00,11.28)	0.0000
I-CBT+P vs Int-CBT vs TAU	2.073	0.494	0.6214	(1.00,37.39)	0.0000
G-CBT vs I-CBT vs WL	2.056	0.911	0.3622	(1.00,9.69)	0.3231
I-BT+P vs I-CBT vs PBO	1.993	0.349	0.727	(1.00,95.91)	0.4091
I-CBT vs I-CBT+P vs PBO	1.963	0.920	0.3575	(1.00,8.26)	0.2858
BIB-CBT vs G-CBT+P vs I-CBT vs PBO	1.931	0.400	0.6889	(1.00,48.44)	0.4091
G-CBT+P vs Int-CBT vs WL	1.800	0.488	0.6253	(1.00,19.05)	0.1500
BIB-CBT vs I-CBT vs I-CBT+P	1.799	0.348	0.7277	(1.00,49.09)	0.2216
G-CBT vs G-CBT+P vs WL	1.736	0.798	0.4251	(1.00,6.74)	0.0912
G-CBT vs G-CBT+P vs I-CBT vs I-CBT+P	1.700	0.546	0.5848	(1.00,11.42)	0.2810
G-CBT vs G-CBT+P vs PBO	1.513	0.504	0.6146	(1.00,7.58)	0.0000
G-CBT vs G-CBT+P vs Int-CBT vs TAU	1.478	0.148	0.8826	(1.00,264.89)	1.1679
I-CBT+P vs Int-CBT vs WL	1.320	0.277	0.7815	(1.00,9.37)	0.0996
BIB-CBT vs G-CBT+P vs I-CBT vs Int-CBT	1.302	0.122	0.9031	(1.00,90.95)	0.0000
G-CBT vs I-CBT+P vs TAU vs WL	1.291	0.187	0.8515	(1.00,18.64)	0.0000
I-BT+P vs I-CBT+P vs PBO	1.269	0.242	0.8091	(1.00,8.78)	0.0000
BIB-CBT vs G-CBT+P vs WL	1.224	0.273	0.7849	(1.00,5.24)	0.1585
I-CBT vs I-CBT+P vs WL	1.223	0.309	0.7574	(1.00,4.40)	0.3504
I+G-BT vs Int-CBT vs PBO vs WL	1.216	0.096	0.9238	(1.00,66.50)	0.0000

© 2018 Zhou X et al. JAMA Psychiatry.

G-CBT vs G-CBT+P vs I-CBT+P vs TAU	1.208	0.123	0.9018	(1.00,24.40)	0.1117
G-CBT+P vs I-CBT+P vs Int-CBT	1.177	0.119	0.9051	(1.00,17.18)	0.0000
G-CBT vs I-CBT vs PBO	1.160	0.179	0.8579	(1.00,5.86)	0.2753
I-BT+P vs I-CBT vs I-CBT+P	1.134	0.071	0.9438	(1.00,37.06)	0.2216
BIB-CBT vs I-CBT+P vs WL	1.092	0.065	0.9485	(1.00,15.57)	0.0000
G-CBT+P vs I-CBT+P vs WL	1.080	0.118	0.906	(1.00,3.85)	0.1473
G-CBT+P vs I-CBT vs PBO vs WL	1.079	0.068	0.9461	(1.00,9.73)	0.3983
BIB-CBT vs G-CBT+P vs I-CBT+P	1.074	0.052	0.959	(1.00,16.38)	0.0000

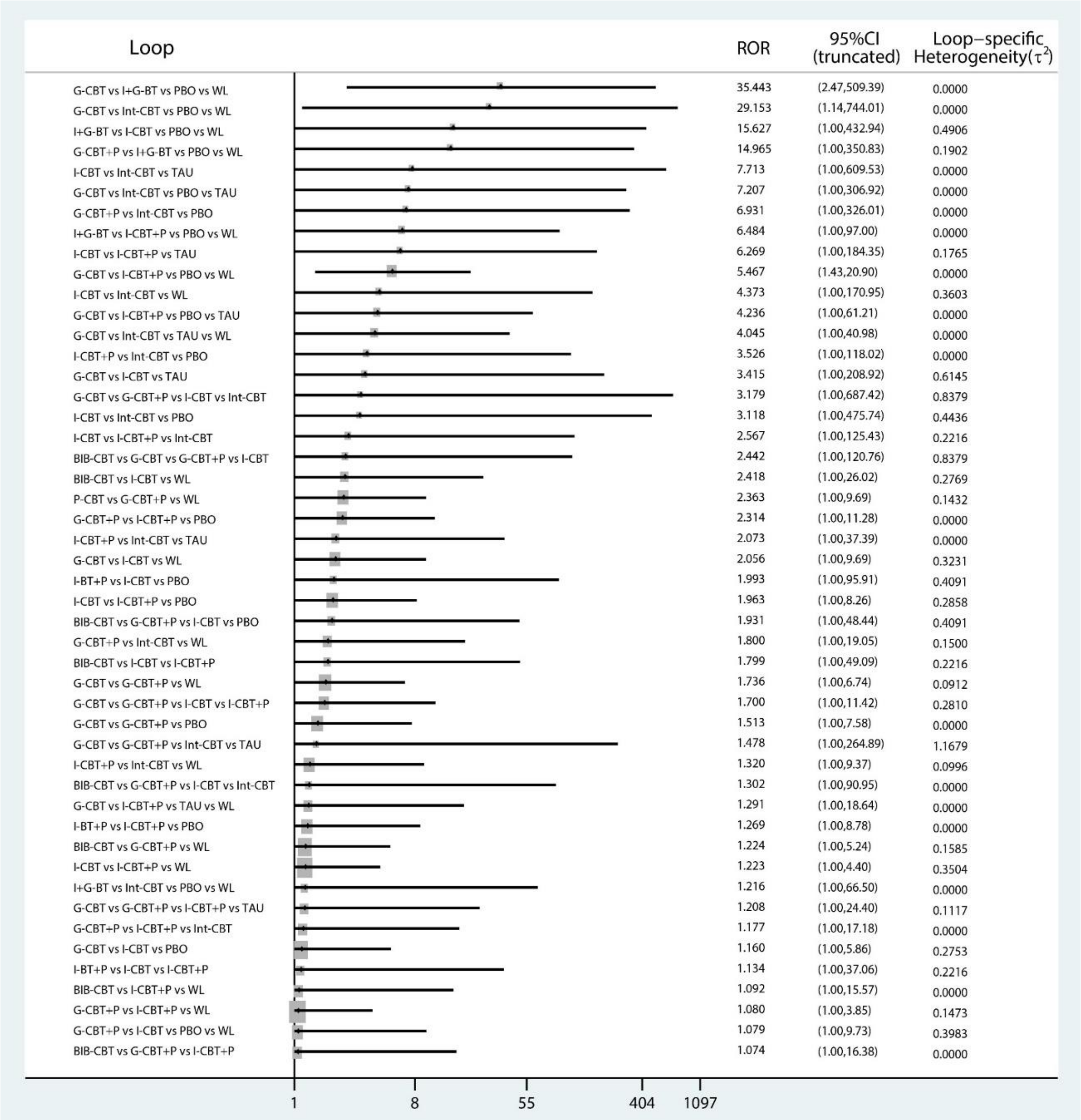

© 2018 Zhou X et al. JAMA Psychiatry.

Mean overall change in quality of life and functional improvement

Loop	IF	z-value	P-value	95%CI	τ^2
G-CBT vs I-CBT vs WL	1.651	1.494	0.1351	(0.00,3.82)	0.4057
G-CBT vs I+G-BT vs PBO vs WL	1.319	0.749	0.4538	(0.00,4.77)	0.7362
G-CBT vs Int-CBT vs PBO vs WL	1.071	0.957	0.3384	(0.00,3.26)	0.4632
G-CBT vs I-CBT vs PBO	0.994	2.157	0.0310	(0.09,1.90)	0.0000
G-CBT vs G-CBT+P vs WL	0.809	1.760	0.0783	(0.00,1.71)	0.0000
G-CBT+P vs I-CBT+P vs WL	0.623	1.190	0.2339	(0.00,1.65)	0.0777
I+G-BT vs I-CBT vs PBO vs WL	0.478	0.886	0.3757	(0.00,1.54)	0.0000
I+G-BT vs Int-CBT vs PBO vs WL	0.246	0.207	0.8360	(0.00,2.58)	0.3452
I-CBT vs Int-CBT vs PBO	0.240	0.384	0.7010	(0.00,1.47)	0.0000
I-CBT+P vs Int-CBT vs WL	0.135	0.162	0.8717	(0.00,1.77)	0.3530
I-CBT+P vs Int-CBT vs TAU	0.118	0.319	0.7498	(0.00,0.84)	0.0000
I-CBT vs I-CBT+P vs Int-CBT	0.047	0.109	0.9132	(0.00,0.89)	0.0000
I-CBT vs Int-CBT vs WL	0.040	0.049	0.9611	(0.00,1.65)	0.2927
I-CBT vs I-CBT+P vs WL	0.039	0.074	0.9414	(0.00,1.07)	0.0922
G-CBT vs G-CBT+P vs I-CBT vs I-CBT+P	0.033	0.060	0.9524	(0.00,1.11)	0.0000

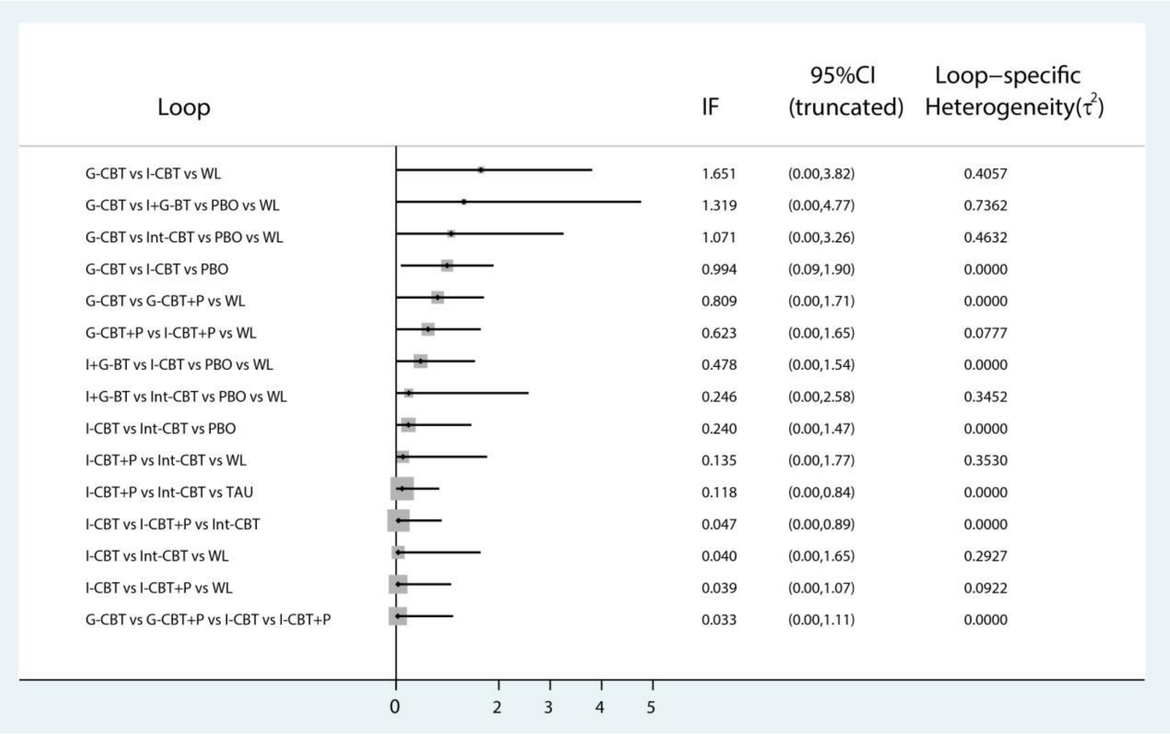

© 2018 Zhou X et al. JAMA Psychiatry.

Mean overall change in symptoms at short-term follow-up

Loop	IF	z-value	P-value	95%CI	τ^2
WL vs G-CBT vs G-CBT+P	3.023	4.392	<0.0001	(1.67,4.37)	0.0737
WL vs P-CBT vs G-CBT+P	2.263	3.734	0.0002	(1.08,3.45)	0.0000
WL vs G-CBT vs I-CBT	2.001	3.586	0.0003	(0.91,3.10)	0.0000
WL vs G-CBT+P vs PBO	1.618	2.946	0.0032	(0.54,2.69)	0.0000
WL vs G-BT vs G-CBT	1.145	2.028	0.0425	(0.04,2.25)	0.0000
WL vs BIB-CBT vs G-CBT+P vs I-CBT	0.997	1.463	0.1433	(0.00,2.33)	0.0000
G-CBT vs I-CBT vs PBO	0.942	1.802	0.0716	(0.00,1.97)	0.0000
WL vs G-CBT vs PBO	0.769	1.592	0.1113	(0.00,1.72)	0.0000
BIB-CBT vs I-CBT vs I-CBT+P	0.535	1.220	0.2224	(0.00,1.39)	0.0236
G-CBT vs G-CBT+P vs PBO	0.459	1.180	0.2379	(0.00,1.22)	0.0000
WL vs I-CBT vs PBO	0.315	0.521	0.6024	(0.00,1.50)	0.0000
BIB-CBT vs G-CBT+P vs I-CBT vs PBO	0.306	0.630	0.5289	(0.00,1.26)	0.0000
I-CBT vs I-CBT+P vs PBO	0.196	0.378	0.7052	(0.00,1.21)	0.0321
G-CBT vs I-CBT vs TAU	0.131	0.254	0.7995	(0.00,1.14)	0.0000
BIB-CBT vs G-CBT+P vs I-CBT+P vs PBO	0.030	0.065	0.9484	(0.00,0.94)	0.0000
BIB-CBT vs G-CBT vs G-CBT+P vs I-CBT	0.016	0.034	0.9730	(0.00,0.94)	0.0000
I-BT+P vs I-CBT+P vs PBO	…	…	…	0	…

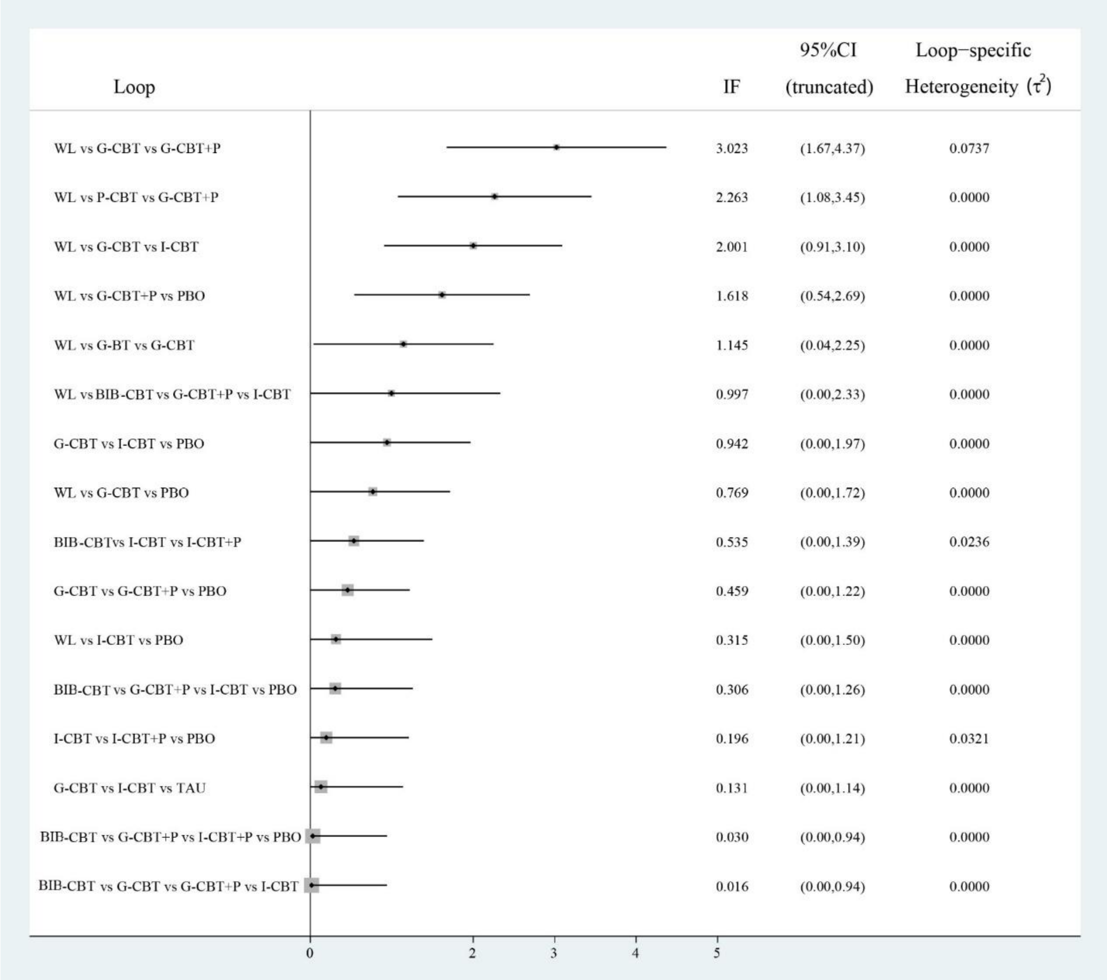

© 2018 Zhou X et al. JAMA Psychiatry.

Mean overall change in symptoms at long-term follow-up

Loop	IF	z-value	P-value	95%CI	τ^2
G-CBT vs I-CBT vs PBO	2.375	4.949	<0.0001	(1.43,3.32)	0.0000
WL vs G-CBT vs PBO	…	…	…	0	…
I-BT+P vs I-CBT+P vs PBO	0.292	0.641	0.5217	(0.00,1.19)	0.0000
I-CBT vs I-CBT+P vs PBO	0.132	0.337	0.7363	(0.00,0.90)	0.0328

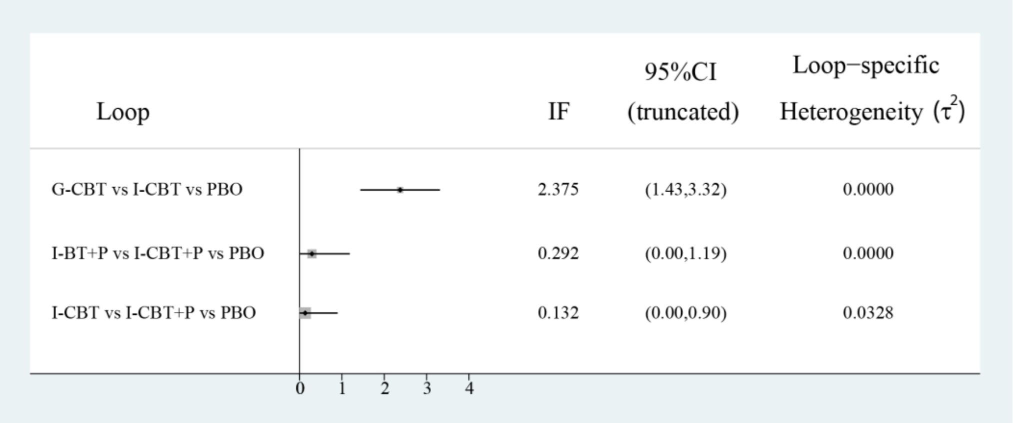

Legend: BIB-CBT=bibliotherapy cognitive-behavioral therapy, G-BT=group behavioral therapy, G-CBT=group cognitive-behavioral therapy, G-CBT+P=group cognitive-behavioral therapy with parental involvement, I-BT+P=individual behavioral therapy with parental involvement, I-CBT=individual cognitive-behavioral therapy, I-CBT+P=individual cognitive-behavioral therapy with parental involvement, I+G-BT=individual and group behavioral therapy, I+G-CBT=individual and group cognitive-behavioral therapy, Int-CBT=internet-assisted cognitive-behavioral therapy, NT=no-treatment, PBO=psychological placebo, P-CBT=parent-only cognitive-behavioral therapy, TAU=treatment as usual, WL=waitlist.

© 2018 Zhou X et al. JAMA Psychiatry.

c. Evaluation of the inconsistency by node-splitting model

Tests of inconsistency by node-splitting method fitted the node-splitting model of Dias et al (Stat Med 2010; 29:932-44). The results reported the estimated direct and indirect treatment effects and their difference; the P-value for the difference is the test of consistency.

Mean overall change in symptoms at post-treatment

Comparisons	Direct		Indirect		Difference			τ^2
	SMD	SE	SMD	SE	SMD	SE	P-value	
BIB-CBT vs WL	0.38	0.31	1.21	0.42	-0.83	0.53	0.1128	0.5680
BIB-CBT vs G-CBT+P	-0.07	0.60	-0.33	0.31	0.27	0.68	0.6929	0.5794
BIB-CBT vs I-CBT	-0.28	0.65	-0.28	0.30	0.00	0.71	0.9951	0.5796
BIB-CBT vs I-CBT+P	0.47	0.47	-0.40	0.32	0.87	0.57	0.1258	0.5695
P-CBT vs WL	0.62	0.30	0.80	0.42	-0.18	0.52	0.7229	0.5794
P-CBT vs G-CBT	-0.24	0.65	-0.77	0.30	0.52	0.72	0.4648	0.5774
P-CBT vs G-CBT+P	-0.22	0.37	-0.31	0.35	0.09	0.51	0.8586	0.5797
G-BT vs WL	1.49	0.48	0.84	1.16	0.65	1.27	0.6105	0.5780
G-BT vs G-CBT	0.25	0.64	-0.17	0.62	0.41	0.89	0.6408	0.5789
G-CBT vs WL	1.93	0.21	0.78	0.21	1.15	0.30	0.0001	0.5177
G-CBT vs G-CBT+P	0.09	0.29	0.63	0.24	-0.54	0.37	0.1488	0.5693
G-CBT vs I-CBT	-0.34	0.32	0.71	0.21	-1.06	0.38	0.0059	0.5491
G-CBT vs NT	0.95	0.35	2.73	31.63	-1.78	31.63	0.9552	0.5750
G-CBT vs PBO	0.67	0.25	0.82	0.30	-0.15	0.38	0.7012	0.5785
G-CBT vs TAU	0.01	0.64	0.88	0.34	-0.87	0.73	0.2322	0.5746
G-CBT+P vs WL	0.95	0.18	0.93	0.24	0.02	0.31	0.9486	0.5797
G-CBT+P vs I-CBT+P	-0.07	0.31	0.24	0.22	-0.32	0.38	0.4053	0.5776
G-CBT+P vs Int-CBT	0.25	0.65	0.36	0.25	-0.11	0.70	0.8794	0.5796
G-CBT+P vs PBO	-0.03	0.61	0.37	0.23	-0.40	0.65	0.5426	0.5785
I+G-BT vs WL	0.78	0.52	0.52	0.68	0.26	0.85	0.7593	0.5790
I+G-BT vs PBO	-0.09	0.65	0.17	0.56	-0.26	0.85	0.7595	0.5790
I+G-CBT vs WL	…	…	…	…	…	…	…	…
I-BT+P vs WL	0.94	0.75	1.05	0.48	-0.11	0.89	0.9000	0.5788
I-BT+P vs I-CBT	0.88	0.85	-0.16	0.46	1.05	0.96	0.2773	0.5745
I-BT+P vs I-CBT+P	-0.23	0.63	0.53	0.53	-0.76	0.82	0.3540	0.5757
I-BT+P vs PBO	0.25	0.65	0.50	0.54	-0.25	0.86	0.7679	0.5788
I-CBT vs WL	0.99	0.23	0.91	0.19	0.08	0.30	0.7900	0.5790
I-CBT vs I-CBT+P	0.00	0.19	0.36	0.23	-0.36	0.29	0.2150	0.5720
I-CBT vs Int-CBT	-0.15	0.67	0.41	0.24	-0.56	0.72	0.4317	0.5770
I-CBT vs PBO	0.41	0.25	0.21	0.28	0.20	0.37	0.5891	0.5782
I-CBT vs TAU	-0.37	0.68	0.43	0.32	-0.80	0.75	0.2845	0.5757
I-CBT+P vs WL	0.41	0.21	1.11	0.19	-0.69	0.29	0.0158	0.5585
I-CBT+P vs Int-CBT	-0.17	0.62	0.26	0.24	-0.43	0.66	0.5175	0.5781

© 2018 Zhou X et al. JAMA Psychiatry.

I-CBT+P vs PBO	0.38	0.45	0.13	0.23	0.25	0.50	0.6177	0.5784
I-CBT+P vs TAU	0.45	0.38	-0.19	0.40	0.63	0.55	0.2531	0.5771
Int-CBT vs WL	0.43	0.21	1.21	0.40	-0.79	0.46	0.0862	0.5665
Int-CBT vs PBO	0.43	0.68	-0.10	0.27	0.53	0.73	0.4719	0.5773
Int-CBT vs TAU	0.31	0.61	-0.19	0.37	0.50	0.71	0.4807	0.5765
PBO vs WL	1.01	0.65	0.59	0.20	0.42	0.68	0.5305	0.5781

Mean overall change in symptoms at follow-up

Comparisons	Direct		Indirect		Difference			τ^2
	SMD	SE	SMD	SE	SMD	SE	P-value	
BIB-CBT vs G-CBT+P	-0.02	0.57	0.00	0.53	-0.02	0.78	0.9795	0.5487
BIB-CBT vs I-CBT	-0.26	0.61	0.16	0.40	-0.42	0.73	0.5630	0.5398
BIB-CBT vs I-CBT+P	0.20	0.45	-0.16	0.48	0.36	0.66	0.5871	0.5421
P-CBT vs WL	1.95	0.64	1.55	0.73	0.40	0.97	0.6792	0.5418
P-CBT vs G-CBT+P	0.06	0.61	0.46	0.75	-0.40	0.97	0.6798	0.5418
G-BT vs G-CBT	-0.32	0.59	-3.01	63.26	2.69	63.26	0.9660	0.5277
G-CBT vs WL	3.05	0.38	0.14	0.33	2.91	0.50	0.0000	0.2556
G-CBT vs G-CBT+P	-0.56	0.32	0.72	0.40	-1.27	0.52	0.0139	0.4696
G-CBT vs I-CBT	-0.45	0.36	0.51	0.39	-0.96	0.53	0.0697	0.5058
G-CBT vs NT	2.69	0.76	3.91	63.26	-1.22	63.26	0.9846	0.5277
G-CBT vs PBO	0.94	0.39	-0.29	0.38	1.23	0.54	0.0241	0.4584
G-CBT vs TAU	-0.04	0.62	-0.08	0.71	0.04	0.94	0.9675	0.5459
G-CBT+P vs WL	-0.26	0.57	2.18	0.35	-2.44	0.67	0.0003	0.3992
G-CBT+P vs PBO	0.17	0.58	0.44	0.40	-0.27	0.71	0.6995	0.5446
I-BT+P vs I-CBT+P	0.23	0.60	-0.09	1.29	0.31	1.42	0.8257	0.5460
I-BT+P vs PBO	0.42	0.63	0.74	1.25	-0.31	1.42	0.8266	0.5460
I-CBT vs WL	0.94	0.69	1.73	0.43	-0.79	0.81	0.3327	0.5355
I-CBT vs I-CBT+P	-0.06	0.18	0.45	0.49	-0.51	0.53	0.3298	0.5299
I-CBT vs Int-CBT	-0.18	0.66	3.00	63.26	-3.18	63.27	0.9599	0.5277
I-CBT vs PBO	0.08	0.42	0.51	0.38	-0.43	0.57	0.4502	0.5386
I-CBT vs TAU	-0.08	0.65	-0.04	0.68	-0.04	0.94	0.9663	0.5459
I-CBT+P vs PBO	0.05	0.42	0.56	0.40	-0.52	0.58	0.3737	0.5319
PBO vs WL	0.99	0.61	1.32	0.48	-0.32	0.78	0.6764	0.5409

All-cause discontinuation

Comparisons	Direct		Indirect		Difference			τ^2
	LogOR	SE	LogOR	SE	LogOR	SE	P-value	
BIB-CBT vs WL	-1.06	0.35	-0.48	0.61	-0.58	0.71	0.4123	0.3097
BIB-CBT vs G-CBT+P	-0.96	0.50	-1.01	0.45	0.05	0.68	0.9411	0.3374
BIB-CBT vs I-CBT	-0.34	0.88	-1.09	0.37	0.75	0.96	0.4309	0.3176
BIB-CBT vs I-CBT+P	-0.58	1.11	-0.82	0.36	0.24	1.17	0.8387	0.3205

© 2018 Zhou X et al. JAMA Psychiatry.

P-CBT vs WL	0.07	0.40	-1.05	0.61	1.12	0.77	0.1447	0.2900
P-CBT vs G-CBT	-0.09	2.05	-0.33	0.40	0.24	2.09	0.9084	0.3194
P-CBT vs G-CBT+P	-0.85	0.44	0.16	0.47	-1.01	0.65	0.1213	0.2990
G-BT vs WL	…	…	…	…	…	…	…	…
G-CBT vs WL	0.54	0.37	-0.36	0.32	0.90	0.49	0.0668	0.2800
G-CBT vs G-CBT+P	0.26	0.50	-0.21	0.34	0.47	0.61	0.4423	0.3056
G-CBT vs I-CBT	-0.37	0.40	0.18	0.33	-0.54	0.51	0.2886	0.2983
G-CBT vs NT	-0.69	0.71	-0.06	108.07	-0.63	108.07	0.9954	0.3169
G-CBT vs PBO	-0.29	0.31	0.81	0.42	-1.10	0.52	0.0358	0.2727
G-CBT vs TAU	-0.51	0.85	-0.02	0.60	-0.49	1.04	0.6369	0.3259
G-CBT+P vs WL	0.06	0.25	0.14	0.36	-0.09	0.44	0.8454	0.3295
G-CBT+P vs I-CBT+P	0.26	0.43	0.17	0.30	0.09	0.53	0.8701	0.3295
G-CBT+P vs Int-CBT	-0.21	1.00	0.24	0.37	-0.46	1.06	0.6663	0.3198
G-CBT+P vs PBO	-0.01	0.68	0.20	0.32	-0.22	0.75	0.7720	0.3296
I+G-BT vs WL	-1.68	1.16	1.04	0.71	-2.72	1.36	0.0455	0.2976
I+G-BT vs PBO	1.01	0.66	-1.71	1.19	2.72	1.36	0.0455	0.2976
I+G-CBT vs WL	…	…	…	…	…	…	…	…
I-BT+P vs WL	0.27	2.07	0.34	0.58	-0.06	2.15	0.9771	0.3193
I-BT+P vs I-CBT	0.00	1.56	0.30	0.58	-0.30	1.67	0.8570	0.3212
I-BT+P vs I-CBT+P	0.27	0.65	0.81	0.93	-0.54	1.12	0.6311	0.3248
I-BT+P vs PBO	0.72	0.69	-0.12	0.92	0.83	1.17	0.4749	0.3223
I-CBT vs WL	0.15	0.33	0.01	0.28	0.14	0.43	0.7528	0.3305
I-CBT vs I-CBT+P	0.16	0.24	0.23	0.34	-0.08	0.42	0.8526	0.3291
I-CBT vs Int-CBT	-0.20	1.59	0.19	0.36	-0.38	1.61	0.8122	0.3208
I-CBT vs PBO	0.12	0.31	0.19	0.40	-0.07	0.51	0.8888	0.3316
I-CBT vs TAU	0.90	1.32	-0.30	0.52	1.20	1.42	0.3963	0.3221
I-CBT+P vs WL	-0.21	0.34	-0.05	0.29	-0.16	0.45	0.7280	0.3213
I-CBT+P vs Int-CBT	-0.28	0.86	0.04	0.39	-0.32	0.94	0.7329	0.3219
I-CBT+P vs PBO	0.58	0.45	-0.33	0.31	0.91	0.54	0.0934	0.2941
I-CBT+P vs TAU	-0.72	0.84	-0.13	0.58	-0.59	1.02	0.5651	0.3164
Int-CBT vs WL	-0.31	0.34	0.70	0.70	-1.00	0.80	0.2072	0.3040
Int-CBT vs PBO	2.03	1.42	-0.16	0.39	2.19	1.46	0.1334	0.3115
Int-CBT vs TAU	-0.04	0.80	-0.49	0.65	0.44	1.03	0.6677	0.3219

Mean overall change in quality of life and functional improvement

Comparisons	Direct		Indirect		Difference			τ^2
	SMD	SE	SMD	SE	SMD	SE	P-value	
BIB-CBT vs I-CBT	0.27	0.53	2.02	63.25	-1.76	63.26	0.9778	0.4431
P-CBT vs WL	…	…	…	…	…	…	…	…
G-CBT vs WL	-1.23	0.40	-0.07	0.39	-1.15	0.56	0.0390	0.4020
G-CBT vs G-CBT+P	0.40	0.57	-0.12	0.47	0.53	0.73	0.4744	0.4544
G-CBT vs I-CBT	1.29	0.53	-0.07	0.37	1.36	0.64	0.0352	0.4063

© 2018 Zhou X et al. JAMA Psychiatry.

G-CBT vs PBO	-0.59	0.31	-1.49	0.57	0.90	0.65	0.1690	0.4316
G-CBT+P vs WL	-0.87	0.31	-0.38	0.52	-0.49	0.61	0.4192	0.4457
G-CBT+P vs I-CBT+P	0.52	0.49	-0.21	0.39	0.74	0.63	0.2407	0.4378
I+G-BT vs WL	-0.42	0.53	-1.01	0.65	0.58	0.83	0.4844	0.4518
I+G-BT vs PBO	-1.05	0.55	-0.47	0.63	-0.58	0.84	0.4842	0.4518
I+G-CBT vs WL	…	…	…	…	…	…	…	…
I-CBT vs WL	-0.87	0.40	-1.10	0.32	0.23	0.51	0.6459	0.4564
I-CBT vs I-CBT+P	-0.17	0.36	-0.25	0.40	0.09	0.54	0.8730	0.4614
I-CBT vs Int-CBT	-0.13	0.57	-0.33	0.32	0.20	0.66	0.7612	0.4573
I-CBT vs PBO	-1.05	0.41	-1.32	0.52	0.27	0.66	0.6872	0.4564
I-CBT+P vs WL	-0.71	0.39	-0.87	0.32	0.16	0.50	0.7566	0.4599
I-CBT+P vs Int-CBT	0.02	0.51	-0.12	0.31	0.14	0.60	0.8082	0.4607
I-CBT+P vs TAU	-0.46	0.36	-0.79	0.58	0.33	0.68	0.6317	0.4573
Int-CBT vs WL	-0.68	0.21	-0.94	0.45	0.26	0.50	0.5960	0.4544
Int-CBT vs PBO	-1.06	0.59	-0.78	0.41	-0.28	0.72	0.6957	0.4560
Int-CBT vs TAU	-0.65	0.50	-0.32	0.46	-0.33	0.68	0.6322	0.4573

Mean overall change in symptoms at short-term follow-up

Comparisons	Direct		Indirect		Difference			τ^2
	SMD	SE	SMD	SE	SMD	SE	P-value	
BIB-CBT vs G-CBT+P	-0.02	0.49	0.38	0.52	-0.39	0.72	0.5834	0.4658
BIB-CBT vs I-CBT	-0.26	0.55	0.03	0.37	-0.30	0.66	0.6529	0.4622
BIB-CBT vs I-CBT+P	0.20	0.40	-0.33	0.43	0.53	0.59	0.3714	0.4507
G-BT vs WL	1.98	0.55	1.25	1.15	0.72	1.31	0.5811	0.4539
G-BT vs G-CBT	-0.33	0.53	0.39	1.19	-0.72	1.31	0.5811	0.4539
G-CBT vs WL	2.72	0.27	0.76	0.35	1.96	0.45	<0.0001	0.2335
G-CBT vs G-CBT+P	0.24	0.51	1.02	0.38	-0.77	0.64	0.2271	0.4474
G-CBT vs I-CBT	-0.03	0.37	1.04	0.36	-1.07	0.52	0.0384	0.3986
G-CBT vs NT	2.69	0.70	4.97	63.25	-2.28	63.25	0.9713	0.4443
G-CBT vs PBO	0.95	0.39	0.63	0.47	0.32	0.62	0.6055	0.4586
G-CBT vs TAU	0.03	0.54	0.50	0.66	-0.47	0.85	0.5775	0.4604
G-CBT+P vs WL	-0.26	0.50	1.84	0.29	-2.10	0.58	0.0003	0.2903
G-CBT+P vs PBO	0.17	0.51	0.00	0.43	0.17	0.67	0.8010	0.4705
I-BT+P vs I-CBT+P	-0.09	0.52	-0.95	1.24	0.86	1.34	0.5230	0.4569
I-BT+P vs PBO	-0.09	0.55	0.76	1.20	-0.86	1.34	0.5236	0.4569
I-CBT vs WL	0.94	0.62	1.73	0.38	-0.78	0.73	0.2822	0.4476
I-CBT vs I-CBT+P	-0.07	0.18	0.52	0.44	-0.59	0.47	0.2116	0.4324
I-CBT vs Int-CBT	-0.18	0.59	3.02	63.26	-3.20	63.26	0.9597	0.4443
I-CBT vs PBO	0.11	0.55	0.38	0.37	-0.27	0.66	0.6819	0.4639
I-CBT vs TAU	-0.08	0.58	-0.55	0.62	0.47	0.85	0.5785	0.4604
I-CBT+P vs PBO	-0.01	0.55	0.42	0.39	-0.43	0.67	0.5233	0.4569
PBO vs WL	1.03	0.54	1.33	0.42	-0.30	0.69	0.6673	0.4593

© 2018 Zhou X et al. JAMA Psychiatry.

P-CBT vs WL	1.95	0.57	1.15	0.63	0.80	0.85	0.3472	0.4496
P-CBT vs G-CBT+P	-0.01	0.53	0.79	0.67	-0.80	0.85	0.3475	0.4496

Mean overall change in symptoms at long-term follow-up

Comparisons	Direct		Indirect		Difference			τ^2
	SMD	SE	SMD	SE	SMD	SE	P-value	
G-BT vs G-CBT	-0.32	0.60	-4.27	63.26	3.95	63.27	0.9502	0.5392
G-CBT vs WL	2.88	0.42	-2.14	0.90	5.03	1.01	<0.0001	0.1496
G-CBT vs G-CBT+P	-0.56	0.36	3.03	23.92	-3.59	23.93	0.8808	0.5392
G-CBT vs I-CBT	-0.77	0.30	1.75	0.41	-2.52	0.51	<0.0001	0.1498
G-CBT vs PBO	1.76	0.35	-0.76	0.37	2.52	0.51	<0.0001	0.1499
G-CBT vs TAU	-0.04	0.61	4.27	63.27	-4.31	63.27	0.9456	0.5392
I-BT+P vs I-CBT+P	0.23	0.62	-0.86	1.48	1.09	1.60	0.4971	0.5666
I-BT+P vs PBO	0.42	0.64	1.51	1.44	-1.08	1.60	0.4982	0.5666
I-CBT vs I-CBT+P	-0.08	0.20	1.99	0.83	-2.08	0.85	0.0150	0.4176
I-CBT vs PBO	0.05	0.57	0.94	0.48	-0.89	0.75	0.2327	0.5299
I-CBT+P vs PBO	0.05	0.33	1.88	0.57	-1.83	0.66	0.0053	0.3830
PBO vs WL	1.12	0.33	6.09	1.01	-4.97	1.01	<0.0001	0.1495
P-CBT vs G-CBT+P	0.06	0.61	-5.38	63.27	5.44	63.27	0.9315	0.5392

Abbreviation: BIB-CBT=bibliotherapy cognitive-behavioral therapy, G-BT=group behavioral therapy, G-CBT=group cognitive-behavioral therapy, G-CBT+P=group cognitive-behavioral therapy with parental involvement, I-BT+P=individual behavioral therapy with parental involvement, I-CBT=individual cognitive-behavioral therapy, I-CBT+P=individual cognitive-behavioral therapy with parental involvement, I+G-BT=individual and group behavioral therapy, I+G-CBT=individual and group cognitive-behavioral therapy, Int-CBT=internet-assisted cognitive-behavioral therapy, NT=no-treatment, PBO=psychological placebo, P-CBT=parent-only cognitive-behavioral therapy, TAU=treatment as usual, WL=waitlist.

© 2018 Zhou X et al. JAMA Psychiatry.

eFigure 5. Comparison-Adjusted Funnel Plot and Egger Test for Each Outcome From the Network Meta-analysis

a. Comparison-adjusted funnel plot for each outcome

Comparison-adjusted funnel plot for mean overall change in symptoms at post-treatment in all comparisons

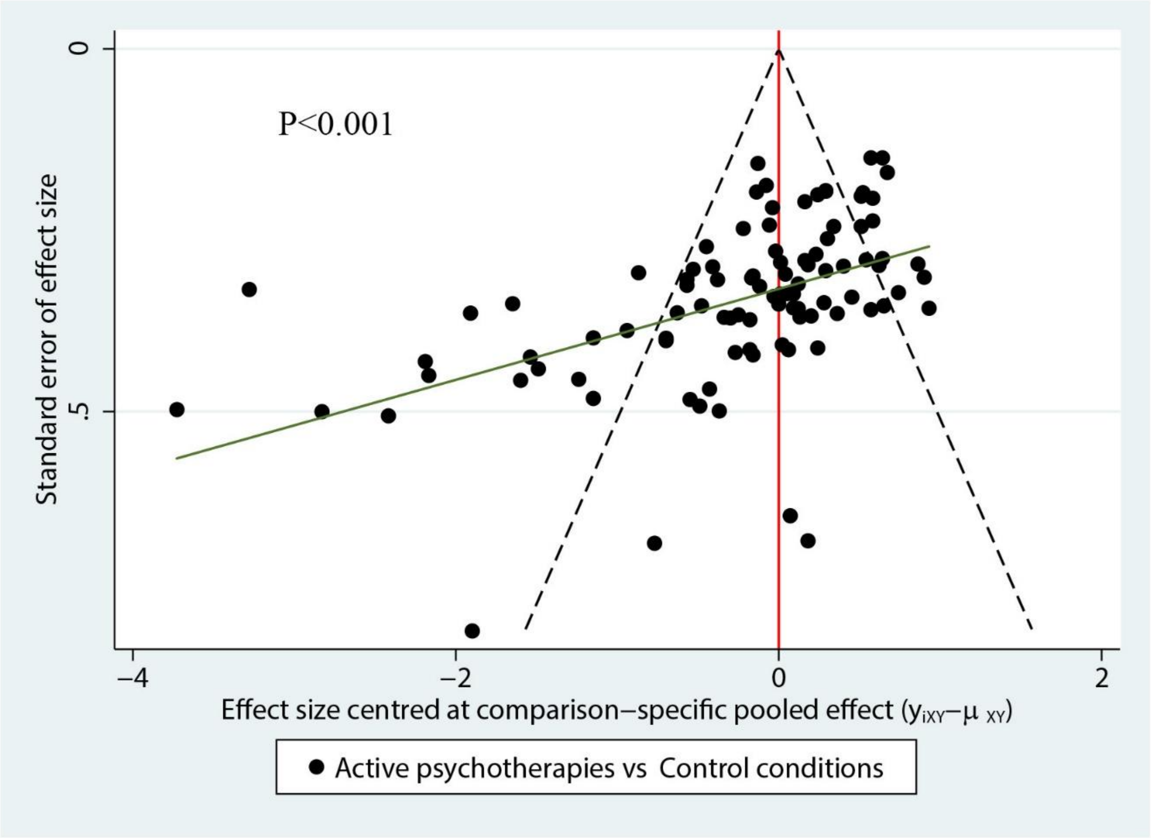

© 2018 Zhou X et al. JAMA Psychiatry.

Comparison-adjusted funnel plot for mean overall change in symptoms at follow-up in all comparisons

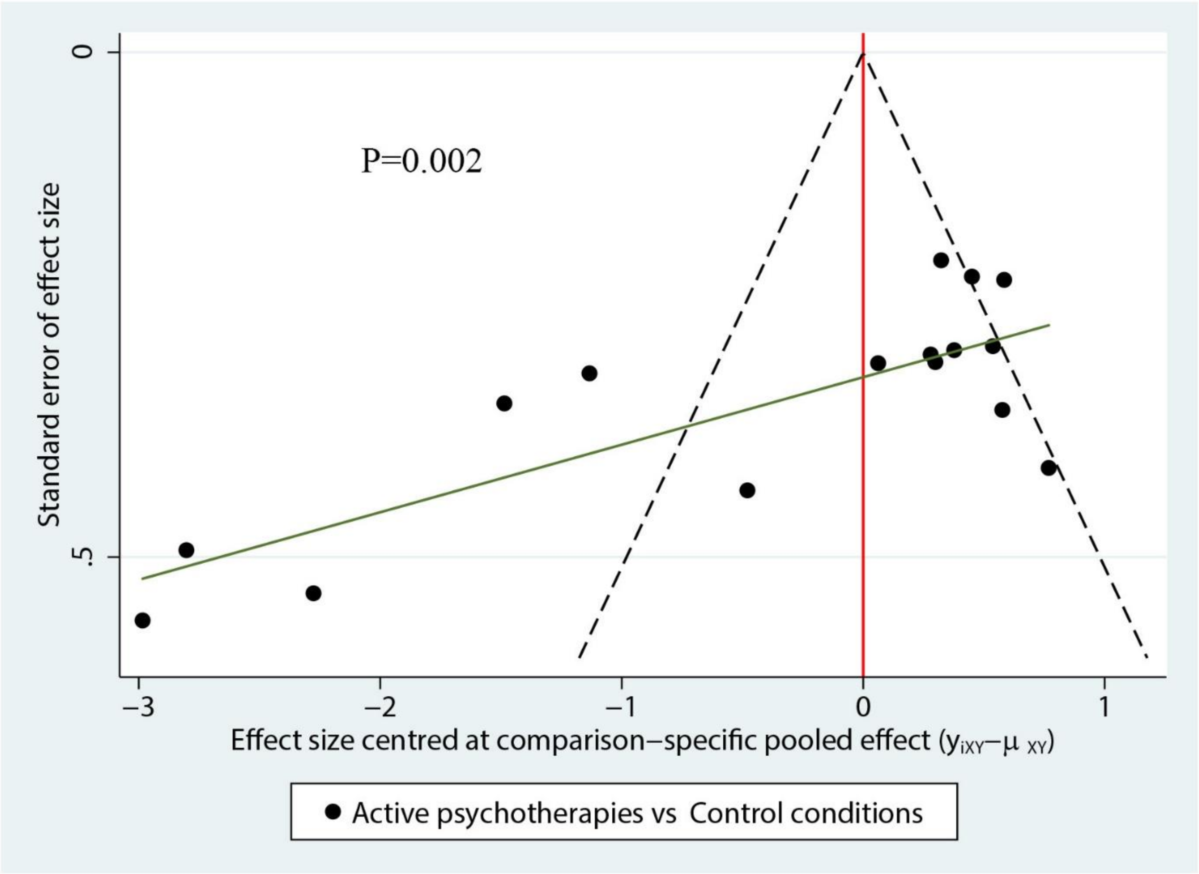

© 2018 Zhou X et al. JAMA Psychiatry.

Comparison-adjusted funnel plot for all-cause discontinuation in all comparisons

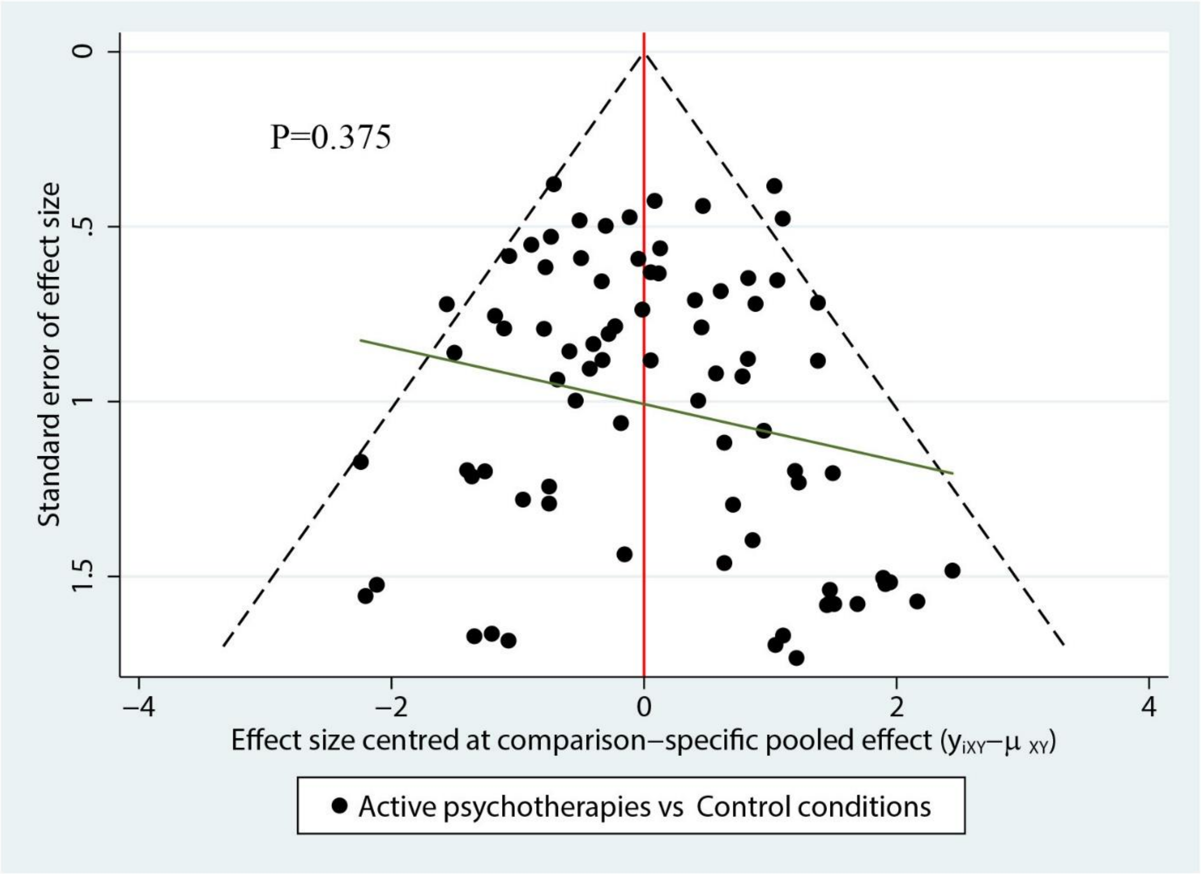

© 2018 Zhou X et al. JAMA Psychiatry.

Comparison-adjusted funnel plot for mean overall change in quality of life and functional improvement in all comparisons

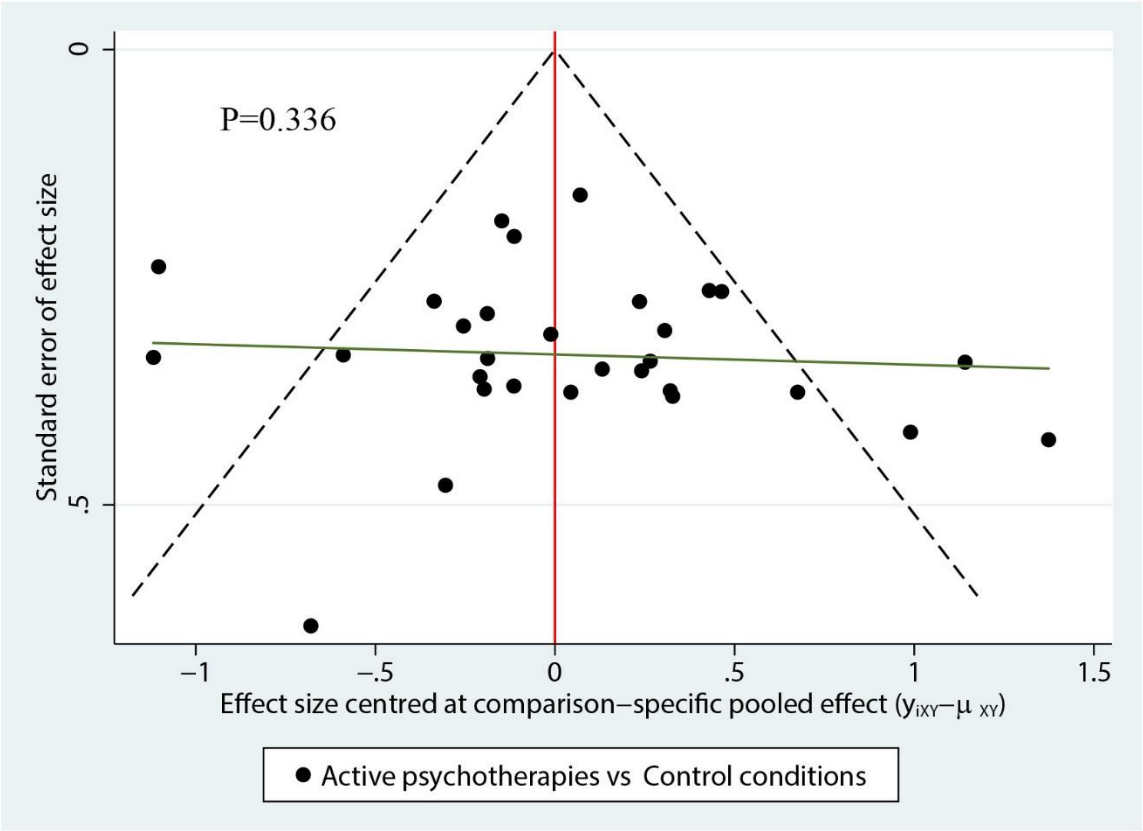

© 2018 Zhou X et al. JAMA Psychiatry.

Comparison-adjusted funnel plot for mean overall change in symptoms at short-term follow-up in all comparisons

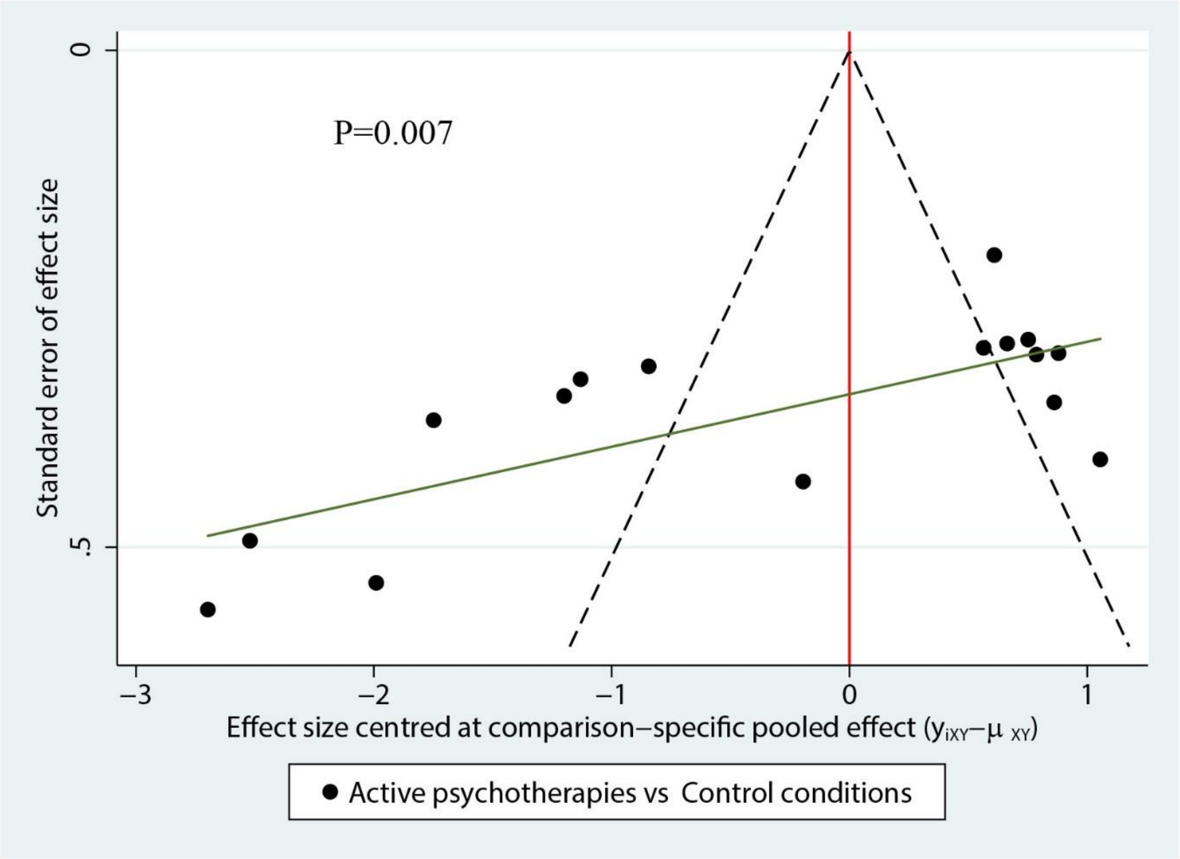

© 2018 Zhou X et al. JAMA Psychiatry.

Comparison-adjusted funnel plot for mean overall change in symptoms at long-term follow-up in all comparisons

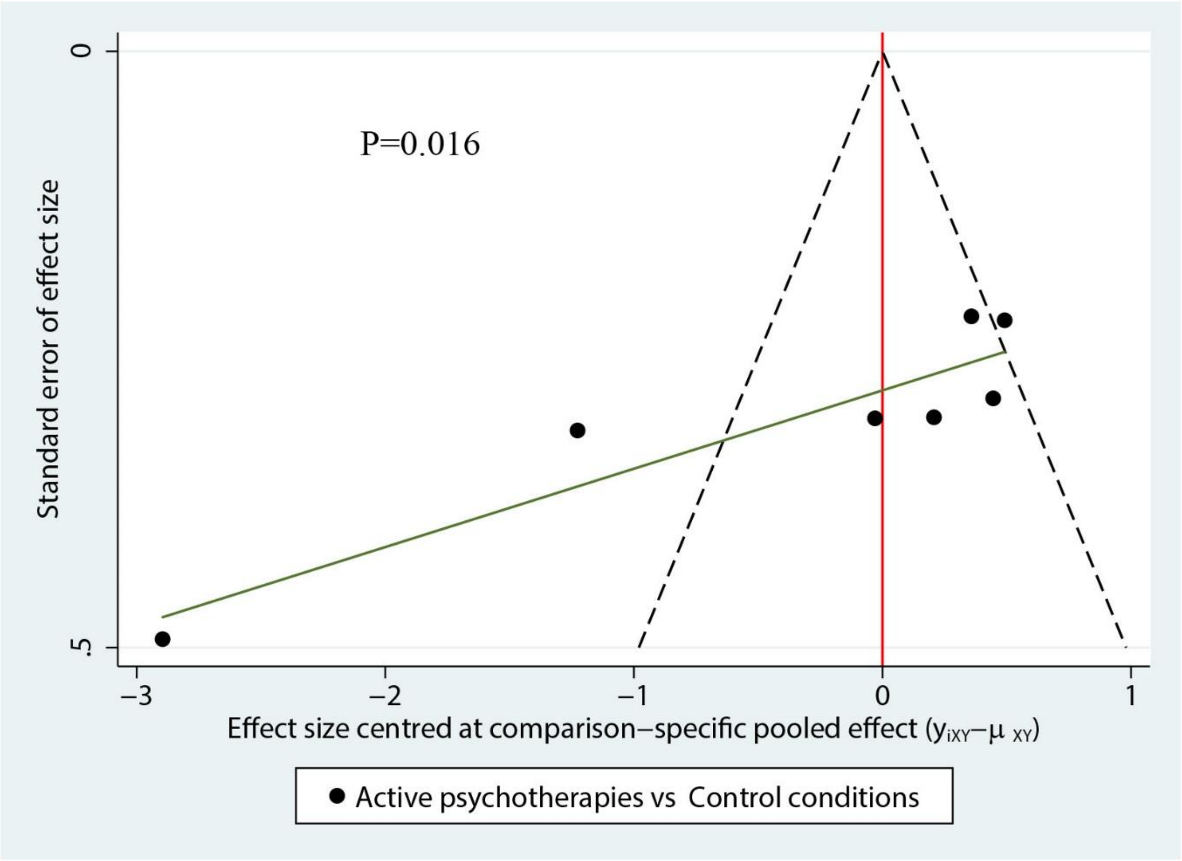

© 2018 Zhou X et al. JAMA Psychiatry.

b. Egger's test for each outcome

Egger's test for mean overall change in symptoms at post-treatment in all comparisons

Comparisons	No. of studies	P-value
BIB-CBT vs WL	4	NA
G-BT vs WL	2	NA
G-CBT vs G-BT	1	NA
G-CBT vs G-CBT+P	5	NA
G-CBT vs NT	4	NA
G-CBT vs PBO	8	NA
G-CBT vs P-CBT	1	NA
G-CBT vs TAU	1	NA
G-CBT vs WL	9	NA
G-CBT+P vs BIB-CBT	1	NA
G-CBT+P vs Int-CBT	1	NA
G-CBT+P vs PBO	1	NA
G-CBT+P vs P-CBT	3	NA
G-CBT+P vs WL	13	0.012*
I-BT+P vs I-CBT	1	NA
I-BT+P vs I-CBT+P	1	NA
I-BT+P vs PBO	1	NA
I-BT+P vs WL	1	NA
I-CBT vs BIB-CBT	1	NA
I-CBT vs G-CBT	4	NA
I-CBT vs I-CBT+P	12	0.229
I-CBT vs PBO	7	NA
I-CBT vs TAU	1	NA
I-CBT vs WL	9	NA
I-CBT+P vs BIB-CBT	2	NA
I-CBT+P vs G-CBT+P	4	NA
I-CBT+P vs PBO	2	NA
I-CBT+P vs TAU	4	NA
I-CBT+P vs WL	9	NA
I+G-BT vs PBO	1	NA
I+G-BT vs WL	2	NA
I+G-CBT vs WL	2	NA
Int-CBT vs I-CBT	1	NA
Int-CBT vs I-CBT+P	1	NA
Int-CBT vs PBO	1	NA
Int-CBT vs TAU	1	NA
Int-CBT vs WL	9	NA
PBO vs WL	1	NA
P-CBT vs WL	5	NA

* The comparisons between G-CBT+P vs WL indicated publication bias.

© 2018 Zhou X et al. JAMA Psychiatry.

Egger's test for mean overall change in symptoms at follow-up in all comparisons

Comparisons	No. of studies	P-value
G-CBT vs G-BT	1	NA
G-CBT vs G-CBT+P	3	NA
G-CBT vs NT	1	NA
G-CBT vs PBO	2	NA
G-CBT vs TAU	1	NA
G-CBT vs WL	2	NA
G-CBT+P vs BIB-CBT	1	NA
G-CBT+P vs PBO	1	NA
G-CBT+P vs P-CBT	1	NA
G-CBT+P vs WL	1	NA
I-BT+P vs I-CBT+P	1	NA
I-BT+P vs PBO	1	NA
I-CBT vs BIB-CBT	1	NA
I-CBT vs G-CBT	3	NA
I-CBT vs I-CBT+P	12	0.961
I-CBT vs PBO	2	NA
I-CBT vs TAU	1	NA
I-CBT vs WL	1	NA
I-CBT+P vs BIB-CBT	2	NA
I-CBT+P vs PBO	2	NA
Int-CBT vs I-CBT	1	NA
PBO vs WL	1	NA
P-CBT vs WL	1	NA

© 2018 Zhou X et al. JAMA Psychiatry.

Egger's test for all-cause discontinuation in all comparisons

Comparisons	No. of studies	P-value
BIB-CBT vs WL	4	NA
P-CBT vs WL	4	NA
G-BT vs WL	1	NA
G-CBT vs WL	7	NA
G-CBT vs P-CBT	1	NA
G-CBT vs G-CBT+P	5	NA
G-CBT vs NT	4	NA
G-CBT vs PBO	7	NA
G-CBT vs TAU	1	NA
G-CBT+P vs WL	13	0.510
G-CBT+P vs BIB-CBT	1	NA
G-CBT+P vs P-CBT	3	NA
G-CBT+P vs Int-CBT	1	NA
G-CBT+P vs PBO	1	NA
I-BT+P vs WL	1	NA
I-BT+P vs I-CBT	1	NA
I-BT+P vs I-CBT+P	1	NA
I-BT+P vs PBO	1	NA
I-CBT vs WL	8	NA
I-CBT vs BIB-CBT	1	NA
I-CBT vs G-CBT	4	NA
I-CBT vs I-CBT+P	11	0.780
I-CBT vs PBO	7	NA
I-CBT vs TAU	1	NA
I-CBT+P vs WL	9	NA
I-CBT+P vs BIB-CBT	2	NA
I-CBT+P vs G-CBT+P	4	NA
I-CBT+P vs PBO	2	NA
I-CBT+P vs TAU	4	NA
I+G-BT vs WL	2	NA
I+G-BT vs PBO	1	NA
I+G-CBT vs WL	1	NA
Int-CBT vs WL	9	NA
Int-CBT vs I-CBT	1	NA
Int-CBT vs I-CBT+P	1	NA
Int-CBT vs PBO	1	NA
Int-CBT vs TAU	1	NA

© 2018 Zhou X et al. JAMA Psychiatry.

Egger's test for mean overall change in quality of life and functional improvement in all comparisons

Comparisons	No. of studies	P-value
G-CBT vs G-CBT+P	1	NA
G-CBT vs PBO	3	NA
G-CBT vs WL	2	NA
G-CBT+P vs WL	3	NA
I-CBT vs BIB-CBT	1	NA
I-CBT vs G-CBT	1	NA
I-CBT vs I-CBT+P	2	NA
I-CBT vs PBO	2	NA
I-CBT vs WL	2	NA
I-CBT+P vs G-CBT+P	1	NA
I-CBT+P vs TAU	3	NA
I-CBT+P vs WL	2	NA
I+G-BT vs PBO	1	NA
I+G-BT vs WL	1	NA
I+G-CBT vs WL	1	NA
Int-CBT vs I-CBT	1	NA
Int-CBT vs I-CBT+P	1	NA
Int-CBT vs PBO	1	NA
Int-CBT vs TAU	1	NA
Int-CBT vs WL	7	NA
P-CBT vs WL	1	NA

© 2018 Zhou X et al. JAMA Psychiatry.

Egger's test for mean overall change in symptoms at short-term follow-up in all comparisons

Comparisons	No. of studies	P-value
G-BT vs WL	1	NA
G-CBT vs G-BT	1	NA
G-CBT vs G-CBT+P	1	NA
G-CBT vs NT	1	NA
G-CBT vs PBO	2	NA
G-CBT vs TAU	1	NA
G-CBT vs WL	3	NA
G-CBT+P vs BIB-CBT	1	NA
G-CBT+P vs PBO	1	NA
G-CBT+P vs P-CBT	1	NA
G-CBT+P vs WL	1	NA
I-BT+P vs I-CBT+P	1	NA
I-BT+P vs PBO	1	NA
I-CBT vs BIB-CBT	1	NA
I-CBT vs G-CBT	2	NA
I-CBT vs I-CBT+P	9	NA
I-CBT vs PBO	1	NA
I-CBT vs TAU	1	NA
I-CBT vs WL	1	NA
I-CBT+P vs BIB-CBT	2	NA
I-CBT+P vs PBO	1	NA
Int-CBT vs I-CBT	1	NA
PBO vs WL	1	NA
P-CBT vs WL	1	NA

© 2018 Zhou X et al. JAMA Psychiatry.

Egger's test for mean overall change in symptoms at long-term follow-up in all comparisons

Comparisons	No. of studies	P-value
G-CBT vs G-BT	1	NA
G-CBT vs G-CBT+P	3	NA
G-CBT vs PBO	1	NA
G-CBT vs TAU	1	NA
G-CBT vs WL	1	NA
G-CBT+P vs P-CBT	1	NA
I-BT+P vs I-CBT+P	1	NA
I-BT+P vs PBO	1	NA
I-CBT vs G-CBT	2	NA
I-CBT vs I-CBT+P	6	NA
I-CBT vs PBO	1	NA
I-CBT+P vs PBO	2	NA
PBO vs WL	1	NA

Legend: BIB-CBT=bibliotherapy cognitive-behavioral therapy, G-BT=group behavioral therapy, G-CBT=group cognitive-behavioral therapy, G-CBT+P=group cognitive-behavioral therapy with parental involvement, I-BT+P=individual behavioral therapy with parental involvement, I-CBT=individual cognitive-behavioral therapy, I-CBT+P=individual cognitive-behavioral therapy with parental involvement, I+G-BT=individual and group behavioral therapy, I+G-CBT=individual and group cognitive-behavioral therapy, Int-CBT=internet-assisted cognitive-behavioral therapy, NT=no-treatment, PBO=psychological placebo, P-CBT=parent-only cognitive-behavioral therapy, TAU=treatment as usual, WL=waitlist

*Egger's test was only used if the number of studies was larger than 10.

© 2018 Zhou X et al. JAMA Psychiatry.

eFigure 6. Treatment Ranking, SUCRA Plot, and Hasse Diagrams

Treatment ranking and SUCRA plot for mean overall change in anxiety symptoms at post-treatment

Treatment ranking:

Treatments	SUCRA (%)
G-CBT	93.4%
G-BT	86.1%
I-BT+P	69.9%
I-CBT	69.5%
G-CBT+P	69.3%
I-CBT+P	54.8%
I+G-BT	45.7%
P-CBT	42.2%
BIB-CBT	42.0%
I+G-CBT	40.8%
PBO	37.9%
TAU	33.5%
Int-CBT	33.4%
NT	29.3%
WL	2.4%

***** Larger SUCRAs denote more effective interventions.

Cumulative probability plots (Random Effects model):

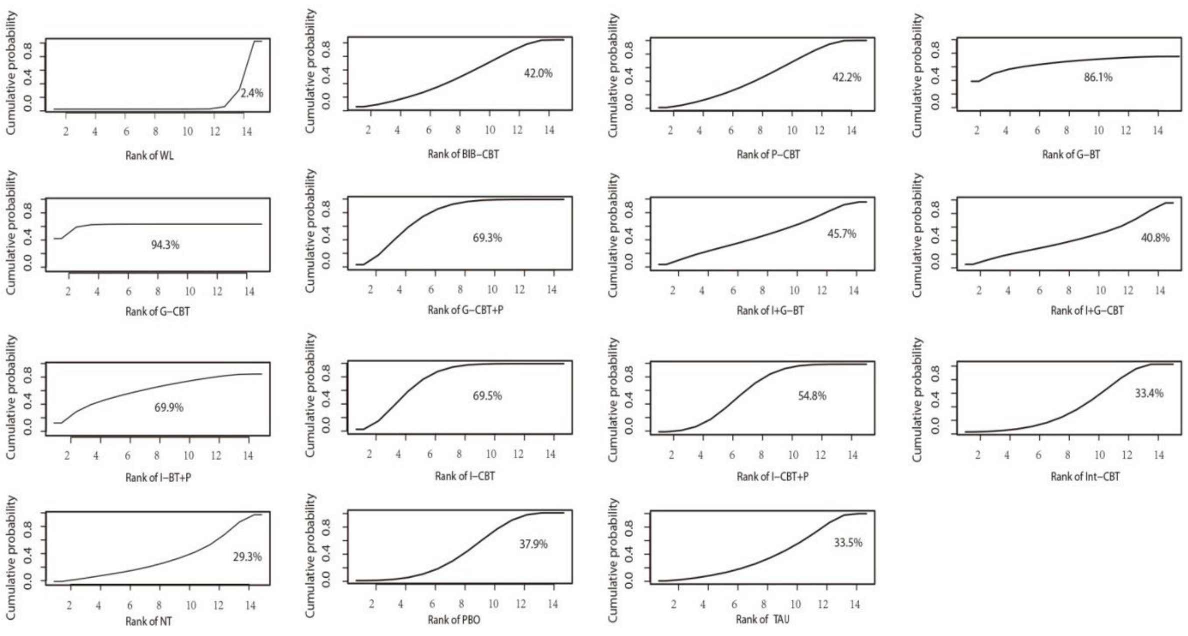

© 2018 Zhou X et al. JAMA Psychiatry.

Treatment ranking and SUCRA plot for mean overall change in symptoms at follow-up

Treatment ranking:

Treatments	SUCRA (%)
P-CBT	67.9%
I-BT+P	66.1%
Int-CBT	65.6%
TAU	62.6%
G-CBT	61.5%
BIB-CBT	60.1%
G-CBT+P	59.7%
I-CBT	58.7%
I-CBT+P	57.6%
G-BT	45.4%
PBO	35.5%
WL	7.8%
NT	1.5%

***** Larger SUCRAs denote more effective interventions.

Cumulative probability plots (Random Effects model):

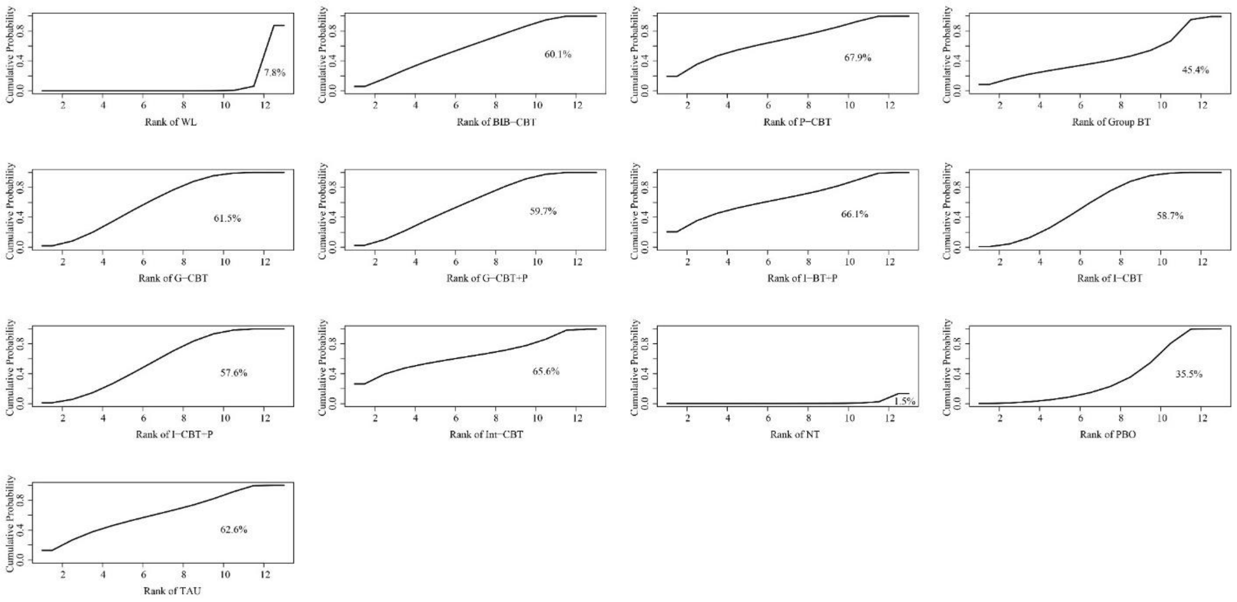

© 2018 Zhou X et al. JAMA Psychiatry.

Treatment ranking and SUCRA plot for all-cause discontinuation

Treatment ranking:

Treatments	SUCRA (%)
NT	85.2%
I+G-CBT	69.3%
TAU	66.4%
I-BT+P	66.0%
G-CBT	59.5%
WL	57.5%
G-CBT+P	56.0%
I-CBT	53.1%
I+G-BT	49.4%
Int-CBT	47.8%
PBO	42.0%
I-CBT+P	35.7%
G-BT	27.6%
P-CBT	27.5%
BIB-CBT	7.1%

***** Larger SUCRAs denote more tolerabale interventions.

Cumulative probability plots (Random Effects model):

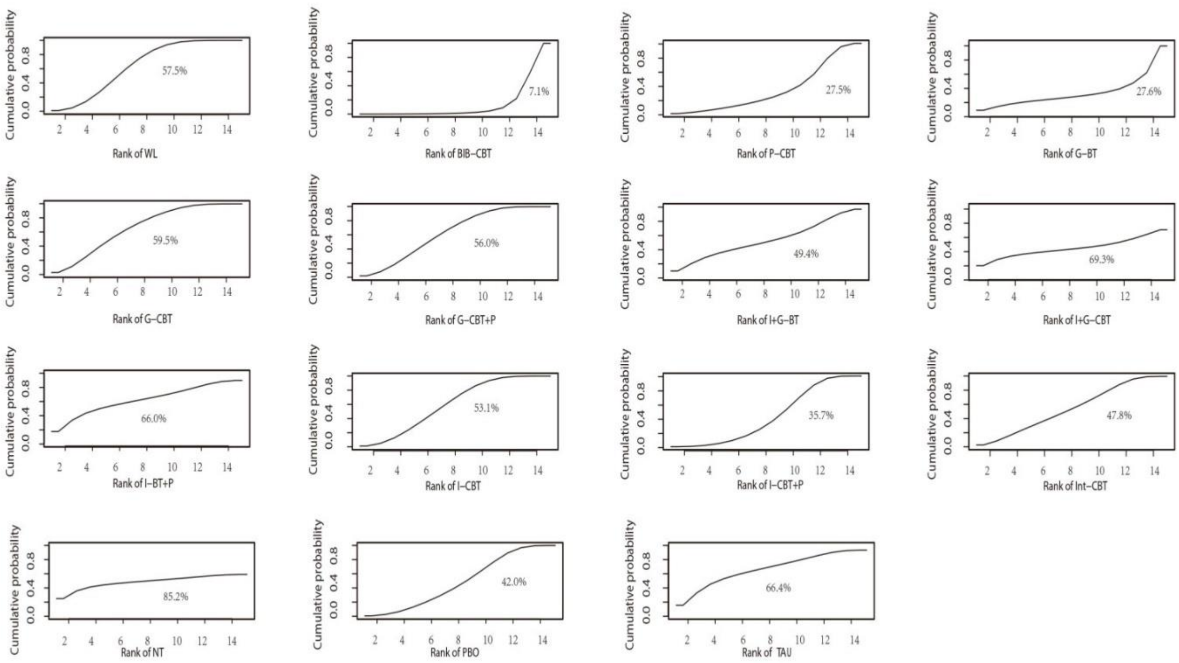

© 2018 Zhou X et al. JAMA Psychiatry.

Treatment ranking and SUCRA plot for mean overall change in quality of life and functional improvement

Treatment ranking:

Treatments	SUCRA (%)
P-CBT	4.4%
I-CBT	23.5%
I-CBT+P	38.6%
G-CBT+P	42.7%
Int-CBT	44.2%
G-CBT	44.8%
BIB-CBT	44.9%
I+G-BT	48.2%
I+G-CBT	55.0%
TAU	73.5%
WL	88.5%
PBO	91.8%

***** Larger SUCRAs denote less effective interventions.

Cumulative probability plots (Random Effects model):

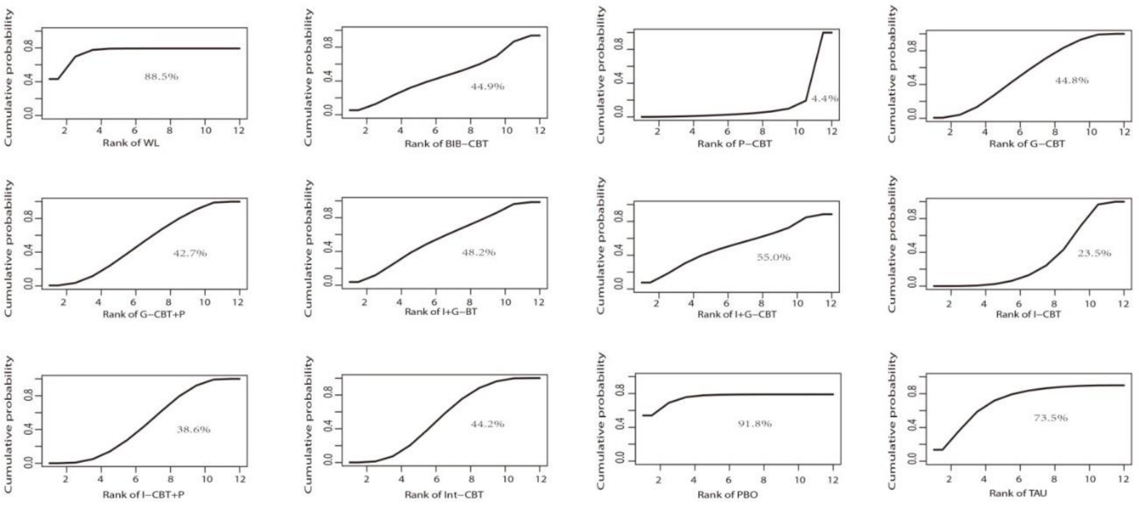

© 2018 Zhou X et al. JAMA Psychiatry.

Treatment ranking and SUCRA plot for mean overall change in anxiety symptoms at short-term follow-up

Treatment ranking:

Treatments	SUCRA (%)
G-CBT	89.3%
TAU	73.7%
G-BT	71.1%
Int-CBT	64.5%
P-CBT	58.8%
I-CBT	56.9%
I-CBT+P	54.1%
BIB-CBT	51.8%
I-BT+P	43.7%
G-CBT+P	39.7%
PBO	36.9%
WL	6.4%
NT	3.1%

***** Larger SUCRAs denote more effective interventions.

Cumulative probability plots (Random Effects model):

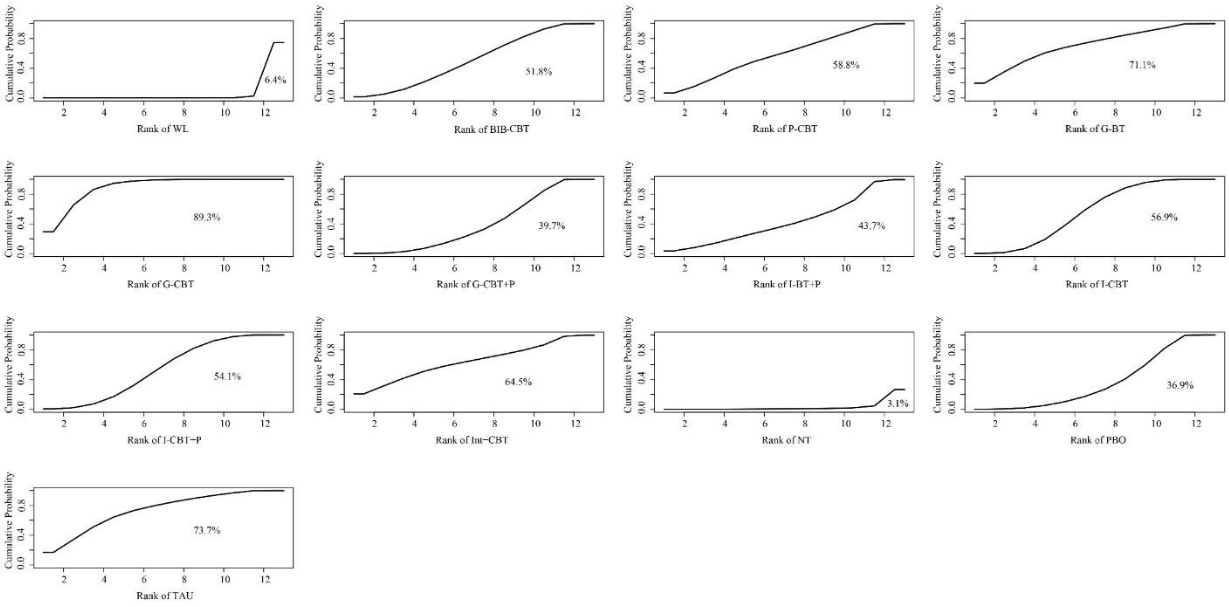

© 2018 Zhou X et al. JAMA Psychiatry.

Treatment ranking and SUCRA plot for mean overall change in anxiety symptoms at long-term follow-up

Treatment ranking:

Treatments	SUCRA (%)
G-CBT+P	81.0%
P-CBT	77.0%
I-BT+P	58.3%
I-CBT	57.8%
I-CBT+P	55.5%
TAU	54.5%
G-CBT	51.6%
G-BT	38.7%
PBO	25.2%
WL	0.6%

***** Larger SUCRAs denote more effective interventions.

Cumulative probability plots (Random Effects model):

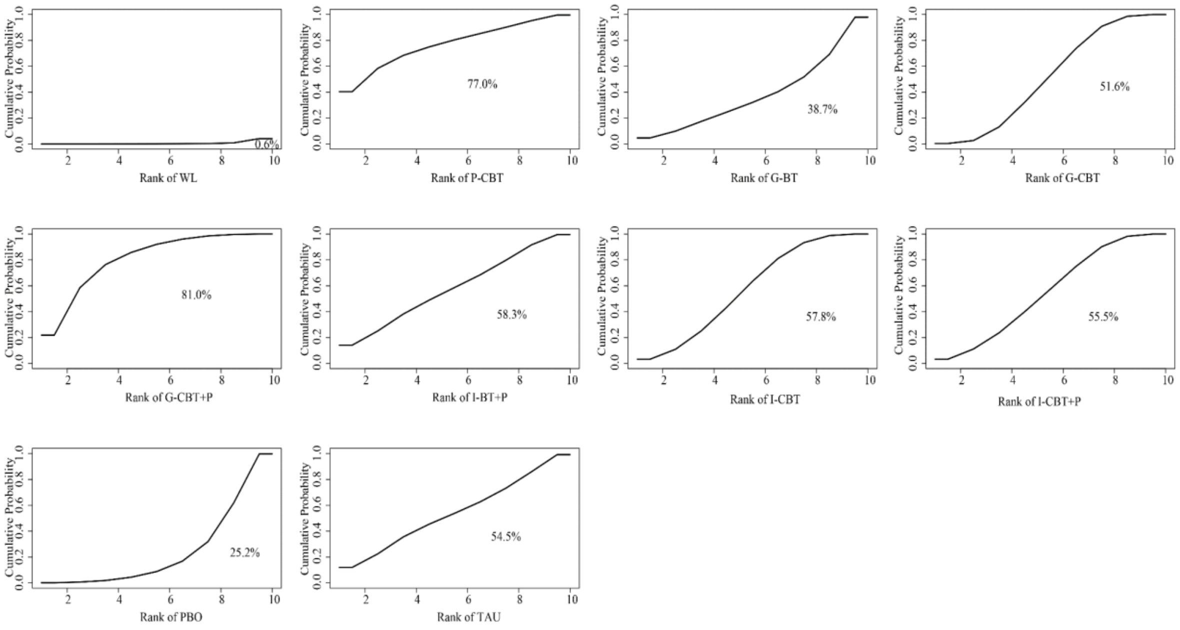

© 2018 Zhou X et al. JAMA Psychiatry.

Hasse diagram for mean overall change in anxiety symptoms at post-treatment and follow-up

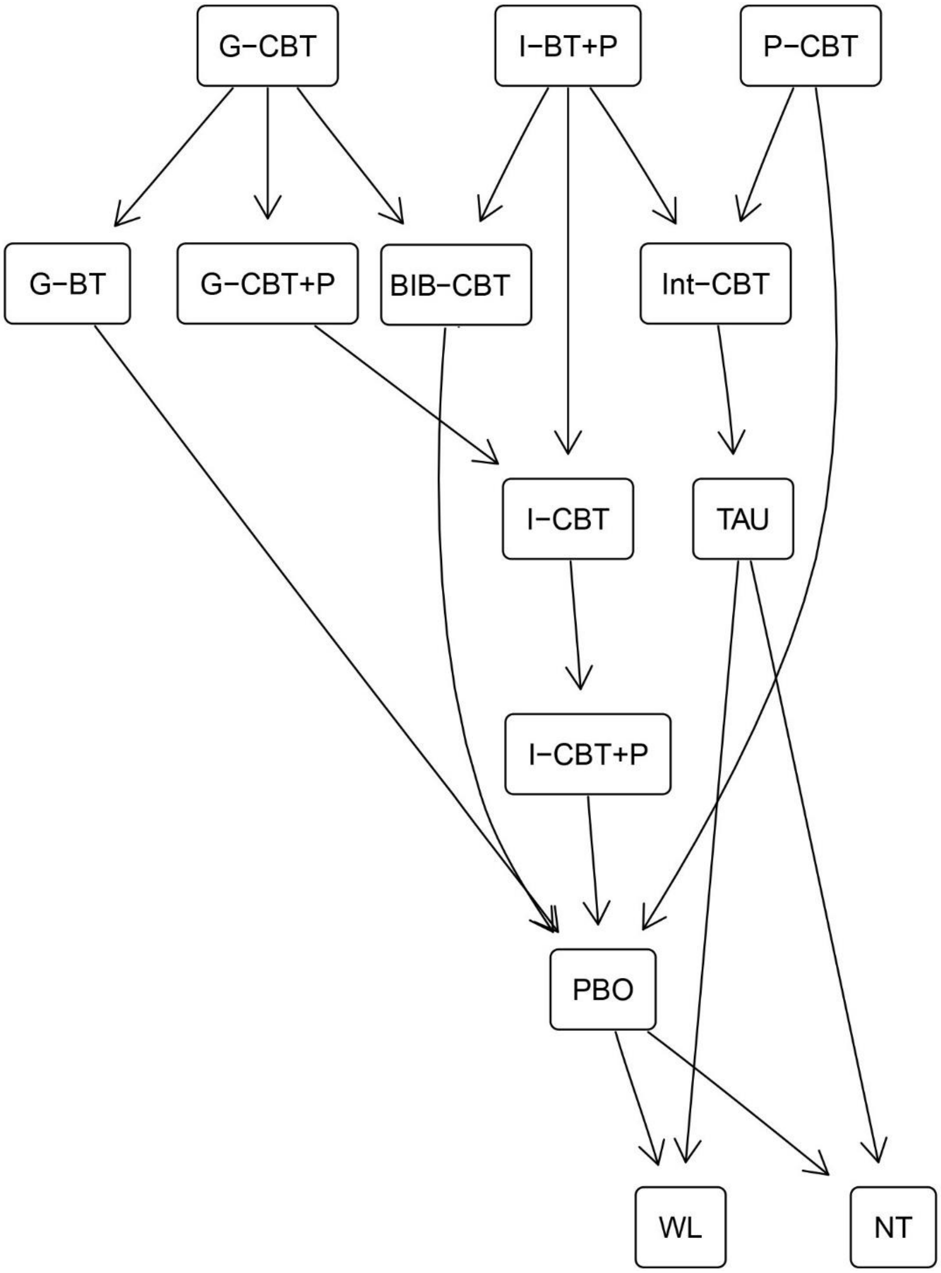

Upper treatments represent higher ranks. BIB-CBT =bibliotherapy cognitive-behavioral therapy, G-BT=group behavioral therapy, G-CBT=group cognitive-behavioral therapy, G-CBT+P=group cognitive-behavioral therapy with parental involvement, I-BT+P=individual behavioral therapy with parental involvement, I-CBT=individual cognitive-behavioral therapy, I-CBT+P=individual cognitive-behavioral therapy with parental involvement, I+G-BT=individual and group behavioral therapy, I+G-CBT=individual and group cognitive-behavioral therapy, Int-CBT=internet-assisted cognitive-behavioral therapy, NT=no-treatment, PBO=psychological placebo, P-CBT=parent-only cognitive-behavioral therapy, TAU=treatment as usual, WL=waitlist.

© 2018 Zhou X et al. JAMA Psychiatry.

eTable 1. Subgroup Network Meta-analyses and Network Metaregression for Efficacy Outcomes

Subgroup network meta-analyses and network meta-regression of each condition for mean improvement in symptoms at post-treatment compared with psychological placebo by standardized mean difference (95% CrI)*

Characteristics	G-CBT	G-BT	I-BT+P	I-CBT	G-CBT+P	I-CBT+P	I+G-BT	P-CBT	BIB-CBT	I+G-CBT	TAU	Int-CBT	NT	WL	SMD-difference[b]
**All trials**	**-0.76 (-1.16 to -0.36)**	-0.77 (-1.76 to 0.22)	-0.42 (-1.29 to 0.44)	-0.32 (-0.72 to 0.07)	-0.33 (-0.78 to 0.13)	-0.18 (-0.61 to 0.25)	-0.06 (-0.94 to 0.82)	-0.04 (-0.67 to 0.60)	-0.03 (-0.68 to 0.61)	0.03 (-1.10 to 1.16)	0.08 (-0.58 to 0.74)	0.06 (-0.48 to 0.60)	0.18 (-0.66 to 1.03)	**0.67 (0.27 to 1.07)**	
**Publication years**															
≤ 2010	-0.81 (-1.27 to -0.37)	-1.38 (-2.62 to -0.14)	-0.09 (-1.29 to 1.11)	-0.22 (-0.67 to 0.24)	-0.54 (-1.06 to -0.04)	-0.17 (-0.67 to 0.33)	-0.14 (-1.21 to 0.92)	-0.39 (-1.32 to 0.54)	-0.13 (-0.98 to 0.72)	-0.28 (-1.85 to 1.29)	-0.80 (-2.24 to 0.63)	-0.01 (-0.81 to 0.78)	0.40 (-1.10 to 1.91)	0.71 (0.24 to 1.17)	-0.12 (-0.40 to 0.16)
> 2010	-0.76 (-1.70 to 0.20)	0.31 (-1.58 to 2.20)	-0.62 (-2.16 to 0.92)	-0.35 (-1.30 to 0.61)	0.25 (-0.87 to 1.37)	0.01 (-1.05 to 1.05)	0.29 (-1.56 to 2.12)	0.36 (-0.86 to 1.58)	0.16 (-1.13 to 1.42)	0.51 (-1.39 to 2.39)	0.40 (-0.80 to 1.60)	0.29 (-0.86 to 1.43)	0.09 (-1.24 to 1.44)	0.84 (-0.19 to 1.87)	
Q test [p value]	0.01 [0.9257]	2.15 [0.1428]	0.28 [0.5947]	0.06 [0.8097]	1.58 [0.2083]	0.09 [0.7616]	0.16 [0.6918]	0.92 [0.3379]	0.14 [0.7107]	0.40 [0.5286]	1.58 [0.2086]	0.18 [0.6732]	0.09 [0.7630]	0.05 [0.8216]	
**Sample size**															
≤ 50	-0.98 (-1.62 to -0.34)	-0.10 (-1.78 to 1.58)	-0.98 (-2.31 to 0.36)	-0.64 (-1.30 to 0.03)	-0.58 (-1.50 to 0.34)	-0.43 (-1.24 to 0.39)	-0.76 (-2.63 to 1.12)	0.25 (-1.42 to 1.93)	-0.64 (-1.90 to 0.61)	-0.20 (-1.50 to 1.09)	-0.06 (-1.08 to 0.97)	-0.31 (-1.23 to 0.61)	-0.04 (-1.04 to 0.97)	0.44 (-0.27 to 1.14)	**-2.64 (-4.07 to -1.21)**
> 50	-0.67 (-1.24 to -0.11)	-1.26 (-2.61 to 0.08)	-0.02 (-1.30 to 1.27)	-0.14 (-0.68 to 0.40)	-0.21 (-0.79 to 0.36)	-0.02 (-0.58 to 0.53)	0.16 (-0.90 to 1.23)	0.01 (-0.75 to 0.77)	0.22 (-0.62 to 1.05)	…	-0.04 (-1.20 to 1.13)	0.23 (-0.51 to 0.98)	…	0.79 (0.25 to 1.34)	
Q test [p value]	0.51 [0.4766]	1.12 [0.2908]	1.03 [0.3099]	1.31 [0.2526]	0.45 [0.5039]	0.66 [0.4151]	0.70 [0.4030]	0.07 [0.7982]	1.25 [0.2635]	….	0.00 [0.9798]	0.80 [0.3713]		0.59 [0.4414]	
**Sex ratio[a]**															
Male-to-female ratio < 1	-0.80 (-1.31 to -0.28)	-0.44 (-1.61 to 0.75)	-1.30 (-3.21 to 0.61)	-0.42 (-0.99 to 0.15)	-0.11 (-0.87 to 0.64)	0.03 (-0.67 to 0.74)	0.23 (-0.80 to 1.27)	0.27 (-0.67 to 1.21)	-0.14 (-1.82 to 1.55)	0.16 (-1.66 to 1.98)	-0.78 (-2.02 to 0.45)	0.61 (-0.22 to 1.45)	-0.09 (-1.17 to 0.99)	1.14 (0.53 to 1.75)	0.27 (-1.69 to 2.22)
Male-to-female	-0.43	…	-0.24	-0.27	-0.30	-0.22	…	-0.34	-0.15	0.09	0.31	-0.12	…	0.43	

© 2018 Zhou X et al. JAMA Psychiatry.

ratio > 1	(-1.10 to 0.25)		(-1.06 to 0.57)	(-0.80 to 0.27)	(-0.85 to 0.24)	(-0.73 to 0.30)		(-1.16 to 0.48)	(-0.82 to 0.51)	(-1.17 to 1.36)	(-0.41 to 1.04)	(-0.78 to 0.55)		(-0.10 to 0.97)	
Q test [p value]	0.73 [0.3930]	…	1.00 [0.3171]	0.14 [0.7069]	0.16 [0.6892]	0.31 [0.5746]	…	0.92 [0.3378]	0.00 [0.9914]	0.00 [0.9506]	2.23 [0.1358]	1.80 [0.1801]	…	2.94 [0.0863]	
**Mean age[b]**															
< 13	-0.50 (-1.03 to 0.02)	-0.79 (-1.70 to 0.12)	-0.43 (-1.22 to 0.36)	-0.26 (-0.70 to 0.18)	-0.31 (-0.80 to 0.16)	-0.22 (-0.68 to 0.23)	0.04 (-0.84 to 0.91)	-0.09 (-0.71 to 0.52)	-0.09 (-0.71 to 0.54)	-0.44 (-1.88 to 0.99)	0.08 (-0.55 to 0.72)	0.00 (-0.57 to 0.57)	1.21 (-0.28 to 2.71)	**0.54 (0.09 to 0.99)**	-1.52 (-3.39 to 0.38)
≥ 13	-0.82 (-1.46 to -0.19)	…	…	-0.80 (-1.63 to 0.04)	-0.48 (-2.19 to 1.23)	0.86 (-0.47 to 2.16)	0.51 (-1.51 to 2.53)	…	…	1.38 (-0.47 to 3.22)	…	1.06 (-0.18 to 2.28)	-0.11 (-1.26 to 1.03)	**1.71 (0.84 to 2.59)**	
Q test [p value]	0.58 [0.4465]	…	…	1.26 [0.2621]	0.04 [0.8512]	2.31 [0.1282]	0.18 [0.6756]	…	…	2.33 [0.1270]	…	2.35 [0.1254]	1.89 [0.1695]	**5.43 [0.0198]**	
**Treatment durations[c]**															
≤ 10 weeks	-0.55 (-1.51 to 0.41)	…	-0.57 (-1.96 to 0.81)	-0.08 (-1.34 to 1.18)	0.27 (-1.01 to 1.55)	-0.66 (-2.26 to 0.95)	…	0.85 (-0.63 to 2.33)	1.60 (-0.47 to 3.70)	…	…	0.97 (-0.48 to 2.44)	-0.09 (-1.63 to 1.47)	1.49 (0.18 to 2.82)	-0.11 (-0.39 to 0.16)
> 10 weeks	-1.01 (-1.51 to -0.51)	-0.83 (-1.83 to 0.18)	-0.24 (-1.81 to 1.32)	-0.22 (-0.70 to 0.26)	-0.59 (-1.19 to 0.00)	-0.17 (-0.68 to 0.33)	-0.04 (-0.91 to 0.83)	-0.50 (-1.52 to 0.52)	-0.20 (-0.91 to 0.52)	0.05 (-1.08 to 1.17)	0.03 (-0.66 to 0.73)	-0.06 (-0.75 to 0.64)	0.44 (-0.71 to 1.58)	0.69 (0.20 to 1.18)	
Q test [p value]	0.69 [0.4049]	…	0.10 [0.7569]	0.04 [0.8387]	1.43 [0.2324]	0.33 [0.5681]	…	2.17 [0.1410]	2.56 [0.1095]	…	…	1.56 [0.2119]	0.29 [0.5899]	1.24 [0.2654]	
**Sessions[d]**															
≤ 10	-0.34 (-1.28 to 0.61)	…	-0.29 (-1.47 to 0.86)	0.00 (-1.11 to 1.12)	-0.21 (-1.10 to 0.67)	0.02 (-0.91 to 0.95)	…	-0.06 (-1.13 to 1.01)	0.37 (-0.77 to 1.51)	…	0.44 (-1.47 to 2.37)	-0.01 (-1.02 to 1.00)	0.50 (-0.78 to 1.79)	0.52 (-0.38 to 1.42)	-0.18 (-0.45 to 0.09)
> 10	-0.95 (-1.44 to -0.47)	-0.74 (-1.77 to 0.29)	-0.15 (-1.77 to 1.46)	-0.30 (-0.78 to 0.17)	-0.63 (-1.28 to 0.02)	-0.22 (-0.74 to 0.31)	0.02 (-0.89 to 0.91)	-0.51 (-1.58 to 0.55)	-0.40 (-1.31 to 0.51)	0.15 (-1.02 to 1.31)	0.10 (-0.70 to 0.89)	-0.14 (-1.01 to 0.73)	0.26 (-1.28 to 1.80)	0.79 (0.29 to 1.28)	
Q test [p value]	1.27 [0.2603]	…	0.02 [0.8904]	0.24 [0.6276]	0.56 [0.4535]	0.19 [0.6596]	…	0.34 [0.5591]	1.07 [0.3008]	…	0.10 [0.7485]	0.04 [0.8484]	0.06 [0.8146]	0.27 [0.6064]	
**Source of outcome information**															
Self-rated	-0.65	-0.72	-0.40	-0.18	-0.28	-0.09	-0.05	0.09	0.02	…	-0.13	0.10	0.19	0.68	**-0.45**

© 2018 Zhou X et al. JAMA Psychiatry.

	(-1.11 to -0.20)	(-1.75 to 0.31)	(-1.43 to 0.62)	(-0.61 to 0.25)	(-0.78 to 0.22)	(-0.56 to 0.38)	(-0.95 to 0.85)	(-0.65 to 0.84)	(-0.66 to 0.69)		(-0.96 to 0.71)	(-0.49 to 0.68)	(-0.80 to 1.18)	(0.23 to 1.13)	**(-0.89 to -0.01)**
Observer-rated	-1.15 (-2.09 to -0.25)	…	-0.06 (-2.04 to 1.91)	-0.87 (-2.02 to 0.28)	-0.64 (-2.00 to 0.72)	0.40 (-1.17 to 1.97)	…	-0.42 (-1.97 to 1.10)	…	0.22 (-1.39 to 1.81)	1.07 (-0.71 to 2.88)	-0.07 (-1.95 to 1.78)	0.06 (-1.73 to 1.84)	0.87 (-0.30 to 2.02)	
Q test [p value]	0.91 [0.3397]	…	0.09 [0.7646]	1.21 [0.2707]	0.24 [0.6263]	0.34 [0.5579]	…	0.34 [0.5580]	…	…	1.41 [0.2348]	0.03 [0.8646]	0.02 [0.9007]	0.09 [0.7647]	

* Negative effect sizes indicate superiority of the specific intervention against PBO control;

[a] We excluded the trials with male-to-female ratio= 1; [b] We excluded the trials with both children and adolescents if mean age were not reported; [c] We excluded the trials with range ≤ 10 weeks and > 10 weeks in different groups; [d] We excluded the trials with range ≤ 10 sessions and > 10 sessions in different groups.

[b] Somer's D (a correlation coefficient for a dichotomous and an ordinal variable) was calculated in meta-regression model, which represent the covariate effect for the whole network meta-analysis, not only for the comparsion of conditions vs. psychological placebo.

BIB-CBT=bibliotherapy cognitive-behavioral therapy, CrI=credibility interval, G-BT=group behavioral therapy, G-CBT=group cognitive-behavioral therapy, G-CBT+P=group cognitive-behavioral therapy with parental involvement, I-BT+P=individual behavioral therapy with parental involvement, I-CBT=individual cognitive-behavioral therapy, I-CBT+P=individual cognitive-behavioral therapy with parental involvement, I+G-BT=individual and group behavioral therapy, I+G-CBT=individual and group cognitive-behavioral therapy, Int-CBT=internet-assisted cognitive-behavioral therapy, NT=no-treatment, P-CBT=parent-only cognitive-behavioral therapy, TAU=treatment as usual, WL=waitlist.

© 2018 Zhou X et al. JAMA Psychiatry.

Subgroup network meta-analyses and network meta-regression of each condition for mean overall change in symptoms at follow-up compared with psychological placebo by standardized mean difference (95% CrI)*

Characteristics	P-CBT	I-BT+P	Int-CBT	TAU	G-CBT	BIB-CBT	G-CBT+P	I-CBT	I-CBT+P	G-BT	WL	NT	SMD-difference[b]
**All trials**	-0.51 (-1.71 to 0.70)	-0.48 (-1.73 to 0.76)	-0.51 (-2.12 to 1.10)	-0.40 (-1.58 to 0.76)	-0.36 (-1.05 to 0.33)	-0.35 (-1.26 to 0.56)	-0.34 (-1.10 to 0.41)	-0.33 (-0.97 to 0.32)	-0.31 (-0.99 to 0.36)	-0.04 (-1.58 to 1.50)	**1.33 (0.50 to 2.15)**	**2.29 (0.58 to 3.99)**	
**Publication years**													
≤ 2010	…	-0.39 (-1.46 to 0.68)	-0.39 (-1.81 to 1.03)	-0.66 (-2.01 to 0.68)	-0.62 (-1.25 to 0.00)	-0.56 (-1.56 to 0.43)	-0.65 (-1.40 to 0.07)	-0.21 (-0.79 to 0.37)	-0.14 (-0.74 to 0.46)	-0.31 (-1.65 to 1.02)	1.71 (0.93 to 2.50)	…	16.07 (-4.88 to 37.15)
> 2010	…	…	…	…	…	…	…	…	…	…	…	…	
Q test [p value]	…	…	…	…	…	…	…	…	…	…	…	…	
**Sample size**													
≤ 50	…	…	…	…	…	…	…	…	…	…	…	…	-7.44 (-28.74 to 13.31)
> 50	-0.46 (-1.44 to 0.52)	-0.50 (-1.48 to 0.47)	…	-0.33 (-1.56 to 0.91)	-0.28 (-0.86 to 0.30)	-0.52 (-1.40 to 0.36)	-0.59 (-1.21 to 0.02)	-0.29 (-0.85 to 0.25)	-0.34 (-0.90 to 0.20)	0.03 (-1.19 to 1.25)	1.66 (0.83 to 2.51)	…	
Q test [p value]	…	…	…	…	…	…	…	…	…	…	…	…	
**Sex ratio[a]**													
Male-to-female ratio < 1	-1.92 (-4.07 to 0.21)	…	…	-1.01 (-2.35 to 0.31)	**-0.91 (-1.81 to -0.01)**	-0.74 (-2.49 to 1.00)	**-1.86 (-3.51 to -0.22)**	-1.00 (-2.08 to 0.07)	-1.16 (-2.37 to 0.04)	-0.60 (-2.22 to 1.04)	**1.88 (0.80 to 2.99)**	…	**21.21 (0.64 to 42.57)**
Male-to-female ratio > 1	-2.34 (-3.46 to -1.23)	-0.32 (-0.85 to 0.22)	-0.28 (-1.14 to 0.58)	…	0.26 (-0.24 to 0.77)	-0.16 (-0.60 to 0.27)	-0.14 (-0.53 to 0.24)	-0.10 (-0.47 to 0.27)	-0.04 (-0.38 to 0.30)	…	-0.40 (-1.34 to 0.54)	…	
Q test [p value]	0.12 [0.7330]	…	…	…	**4.94 [0.0263]**	0.40 [0.5273]	**3.98 [0.0460]**	2.41 [0.1208]	3.07 [0.0796]	…	**9.59 [0.0020]**	…	
**Mean age[b]**													
< 13	-0.74 (-1.47 to -0.01)	-0.36 (-1.02 to 0.29)	-0.23 (-1.19 to 0.75)	-0.03 (-0.78 to 0.72)	0.08 (-0.64 to 0.80)	-0.10 (-0.62 to 0.41)	-0.12 (-0.60 to 0.37)	-0.04 (-0.46 to 0.37)	-0.10 (-0.51 to 0.30)	0.40 (-0.60 to 1.41)	0.68 (-0.02 to 1.34)	2.74 (1.50 to 3.99)	**-51.40 (-81.90 to -21.61)**

© 2018 Zhou X et al. JAMA Psychiatry.

≥ 13	…	…	…	…	-0.90 (-2.00 to 0.18)	…	-1.85 (-3.83 to 0.12)	-1.04 (-2.39 to 0.30)	-0.18 (-2.49 to 2.12)	…	1.90 (0.58 to 3.24)	…	
Q test [p value]	…	…	…	…	2.16 [0.1415]	…	2.78 [0.0955]	1.94 [0.1638]	0.00 [0.9466]	…	2.56 [0.1094]	…	
**Treatment durations[c]**													
≤ 10 weeks	-2.38 (-4.73 to -0.03)	-0.42 (-1.77 to 0.94)	…	…	…	…	-0.17 (-1.48 to 1.14)	-1.37 (-3.76 to 1.05)	-0.19 (-1.56 to 1.18)	…	-0.43 (-2.38 to 1.50)	…	**-49.22 (-77.95 to -21.07)**
> 10 weeks	-1.17 (-2.44 to 0.08)	…	-0.58 (-1.84 to 0.67)	-0.65 (-1.55 to 0.23)	-0.75 (-1.36 to -0.16)	-0.66 (-1.41 to 0.07)	-1.11 (-1.87 to -0.38)	-0.40 (-0.99 to 0.17)	-0.43 (-1.04 to 0.17)	-0.44 (-1.60 to 0.71)	**1.95 (1.18 to 2.73)**	1.89 (0.52 to 3.27)	
Q test [p value]	0.79 [0.3738]	…	…	…	…	…	1.49 [0.2215]	0.59 [0.4422]	0.10 [0.7535]	…	**4.99 [0.0256]**	…	
**Sessions[d]**													
≤ 10	-1.96 (-3.60 to -0.28)	-0.48 (-1.57 to 0.60)	…	…	-4.88 (-110.30 to 122.00)	-0.18 (-1.36 to 0.96)	-0.10 (-0.99 to 0.86)	-0.59 (-1.71 to 0.47)	-0.30 (-1.27 to 0.65)	…	-0.01 (-1.24 to 1.24)	-2.23 (-107.80 to 124.60)	**-51.02 (-81.49 to -21.73)**
> 10	-1.54 (-3.08 to 0.01)	…	-0.59 (-1.98 to 0.78)	-0.79 (-2.09 to 0.51)	-0.75 (-1.42 to -0.07)	-0.66 (-1.74 to 0.41)	-1.48 (-2.53 to -0.42)	-0.41 (-1.08 to 0.22)	-0.45 (-1.16 to 0.22)	-0.43 (-1.71 to 0.86)	**1.96 (1.12 to 2.84)**	…	
Q test [p value]	0.13 [0.7166]	…	…	…	0.00 [0.9444]	0.35 [0.5519]	3.72 [0.0539]	0.08 [0.7810]	0.06 [0.8036]	…	**6.55 [0.0105]**	…	

* Negative effect sizes indicate superiority of the specific intervention against PBO control;

[a] We excluded the trials with male-to-female ratio= 1; [b] We excluded the trials with both children and adolescents if mean age were not reported; [c] We excluded the trials with range ≤ 10 weeks and > 10 weeks in different groups; [d] We excluded the trials with range ≤ 10 sessions and > 10 sessions in different groups.

[b] Somer's D (a correlation coefficient for a dichotomous and an ordinal variable) was calculated in meta-regression model, which represent the covariate effect for the whole network meta-analysis, not only for the comparsion of conditions vs. psychological placebo.

BIB-CBT=bibliotherapy cognitive-behavioral therapy, CrI=credibility interval, G-BT=group behavioral therapy, G-CBT=group cognitive-behavioral therapy, G-CBT+P=group cognitive-behavioral therapy with parental involvement, I-BT+P=individual behavioral therapy with parental involvement, I-CBT=individual cognitive-behavioral therapy, I-CBT+P=individual cognitive-behavioral therapy with parental involvement, Int-CBT=internet-assisted cognitive-behavioral therapy, NT=no-treatment, PBO=psychological placebo, P-CBT=parent-only cognitive-behavioral therapy, TAU=treatment as usual, WL=waitlist.

© 2018 Zhou X et al. JAMA Psychiatry.

**G-CBT**	-0.32<br>(-1.78 to 1.16)	-0.24<br>(-1.78 to 1.30)	-0.12<br>(-0.87 to 0.61)	-0.46<br>(-1.45 to 0.52)	-0.45<br>(-1.46 to 0.55)	…	-0.06<br>(-1.73 to 1.60)	-0.33<br>(-1.47 to 0.79)	…	-0.68<br>(-1.59 to 0.22)	0.04<br>(-1.45 to 1.53)	-0.28<br>(-2.12 to 1.56)	**-2.66**<br>**(-4.30 to -1.01)**	**-1.95**<br>**(-2.84 to -1.06)**
0.01<br>(-0.95 to 0.96)	**G-BT**	0.07<br>(-2.06 to 2.20)	0.19<br>(-1.46 to 1.83)	-0.15<br>(-1.93 to 1.63)	-0.14<br>(-1.93 to 1.64)	…	0.25<br>(-1.97 to 2.47)	-0.02<br>(-1.88 to 1.83)	…	-0.37<br>(-2.10 to 1.36)	0.36<br>(-1.74 to 2.44)	0.03<br>(-2.32 to 2.39)	**-2.34**<br>**(-4.55 to -0.14)**	-1.64<br>(-3.35 to 0.09)
-0.34<br>(-1.22 to 0.54)	-0.35<br>(-1.60 to 0.90)	**I-BT+P**	0.12<br>(-1.50 to 1.73)	-0.22<br>(-1.59 to 1.14)	-0.21<br>(-1.54 to 1.11)	…	0.18<br>(-2.02 to 2.37)	-0.09<br>(-1.68 to 1.51)	…	-0.44<br>(-1.78 to 0.89)	0.28<br>(-1.86 to 2.42)	-0.04<br>(-2.11 to 2.02)	**-2.41**<br>**(-4.67 to -0.15)**	**-1.71**<br>**(-3.37 to -0.05)**
**-0.43**<br>**(-0.82 to -0.04)**	-0.44<br>(-1.41 to 0.54)	-0.09<br>(-0.97 to 0.79)	**G-CBT+P**	-0.34<br>(-1.41 to 0.74)	-0.33<br>(-1.42 to 0.77)	…	0.06<br>(-1.43 to 1.55)	-0.21<br>(-1.30 to 0.88)	…	-0.56<br>(-1.59 to 0.49)	0.16<br>(-1.50 to 1.83)	-0.16<br>(-2.04 to 1.74)	**-2.53**<br>**(-4.33 to -0.73)**	**-1.83**<br>**(-2.81 to -0.83)**
**-0.44**<br>**(-0.83 to -0.06)**	-0.45<br>(-1.43 to 0.52)	-0.10<br>(-0.95 to 0.74)	-0.01<br>(-0.41 to 0.38)	**I-CBT**	0.01<br>(-0.41 to 0.42)	…	0.40<br>(-1.45 to 2.23)	0.13<br>(-0.83 to 1.09)	…	-0.22<br>(-1.00 to 0.57)	0.51<br>(-1.28 to 2.28)	0.18<br>(-1.37 to 1.74)	**-2.19**<br>**(-4.11 to -0.28)**	**-1.49**<br>**(-2.66 to -0.31)**
**-0.58**<br>**(-0.98 to -0.17)**	-0.58<br>(-1.56 to 0.39)	-0.23<br>(-1.09 to 0.61)	-0.15<br>(-0.53 to 0.24)	-0.13<br>(-0.45 to 0.19)	**I-CBT+P**	…	0.39<br>(-1.46 to 2.23)	0.12<br>(-0.84 to 1.09)	…	-0.23<br>(-1.01 to 0.56)	0.50<br>(-1.30 to 2.29)	0.17<br>(-1.43 to 1.79)	**-2.20**<br>**(-4.13 to -0.28)**	**-1.50**<br>**(-2.69 to -0.31)**
-0.70<br>(-1.60 to 0.21)	-0.71<br>(-1.97 to 0.56)	-0.36<br>(-1.55 to 0.83)	-0.27<br>(-1.18 to 0.64)	-0.26<br>(-1.15 to 0.64)	-0.12<br>(-1.03 to 0.78)	**I+G-BT**	…	…	…	…	…	…	…	…
**-0.72**<br>**(-1.31 to -0.14)**	-0.73<br>(-1.79 to 0.33)	-0.38<br>(-1.36 to 0.60)	-0.29<br>(-0.84 to 0.25)	-0.28<br>(-0.87 to 0.31)	-0.15<br>(-0.73 to 0.44)	-0.02<br>(-1.03 to 0.98)	**P-CBT**	-0.27<br>(-2.11 to 1.57)	…	-0.62<br>(-2.43 to 1.20)	0.10<br>(-2.12 to 2.34)	-0.22<br>(-2.61 to 2.21)	**-2.59**<br>**(-4.92 to -0.25)**	**-1.89**<br>**(-3.67 to -0.10)**
**-0.72**<br>**(-1.34 to -0.10)**	-0.73<br>(-1.81 to 0.35)	-0.38<br>(-1.37 to 0.61)	-0.29<br>(-0.89 to 0.30)	-0.28<br>(-0.87 to 0.31)	-0.15<br>(-0.72 to 0.43)	-0.02<br>(-1.04 to 0.99)	0.00<br>(-0.74 to 0.74)	**BIB-CBT**	…	-0.35<br>(-1.46 to 0.76)	0.37<br>(-1.49 to 2.24)	0.05<br>(-1.77 to 1.88)	**-2.32**<br>**(-4.32 to -0.32)**	**-1.62**<br>**(-2.92 to -0.32)**
-0.78<br>(-1.89 to 0.33)	-0.79<br>(-2.20 to 0.62)	-0.44<br>(-1.80 to 0.91)	-0.35<br>(-1.45 to 0.76)	-0.34<br>(-1.44 to 0.77)	-0.21<br>(-1.31 to 0.90)	-0.08<br>(-1.45 to 1.28)	-0.06<br>(-1.23 to 1.12)	-0.06<br>(-1.25 to 1.14)	**I+G-CBT**	…	…	…	…	…
**-0.76**<br>**(-1.16 to -0.36)**	-0.77<br>(-1.77 to 0.23)	-0.42<br>(-1.30 to 0.45)	-0.33<br>(-0.79 to 0.12)	-0.32<br>(-0.72 to 0.08)	-0.19<br>(-0.62 to 0.25)	-0.06<br>(-0.95 to 0.83)	-0.04<br>(-0.67 to 0.60)	-0.04<br>(-0.69 to 0.61)	0.02<br>(-1.11 to 1.15)	**PBO**	0.72<br>(-1.02 to 2.46)	0.40<br>(-1.34 to 2.15)	**-1.97**<br>**(-3.85 to -0.09)**	**-1.27**<br>**(-2.35 to -0.19)**
**-0.84**<br>**(-1.47 to -0.20)**	-0.84<br>(-1.95 to 0.25)	-0.49<br>(-1.50 to 0.50)	-0.41<br>(-1.05 to 0.24)	-0.39<br>(-1.00 to 0.21)	-0.26<br>(-0.84 to 0.31)	-0.14<br>(-1.18 to 0.90)	-0.11<br>(-0.90 to 0.67)	-0.11<br>(-0.90 to 0.67)	-0.05<br>(-1.27 to 1.16)	-0.07<br>(-0.74 to 0.60)	**TAU**	-0.32<br>(-2.69 to 2.03)	**-2.70**<br>**(-4.92 to -0.48)**	**-1.99**<br>**(-3.72 to -0.26)**
**-0.82**<br>**(-1.33 to -0.30)**	-0.83<br>(-1.84 to 0.19)	-0.48<br>(-1.40 to 0.45)	-0.39<br>(-0.88 to 0.11)	-0.38<br>(-0.86 to 0.11)	-0.24<br>(-0.73 to 0.24)	-0.12<br>(-1.07 to 0.83)	-0.10<br>(-0.75 to 0.56)	-0.09<br>(-0.77 to 0.58)	-0.04<br>(-1.18 to 1.10)	-0.06<br>(-0.60 to 0.49)	0.02<br>(-0.65 to 0.69)	**Int-CBT**	-2.37<br>(-4.84 to 0.09)	-1.67<br>(-3.62 to 0.28)
**-0.94**<br>**(-1.69 to -0.19)**	-0.95<br>(-2.17 to 0.26)	-0.60<br>(-1.76 to 0.55)	-0.51<br>(-1.36 to 0.33)	-0.50<br>(-1.34 to 0.34)	-0.36<br>(-1.22 to 0.49)	-0.24<br>(-1.42 to 0.93)	-0.22<br>(-1.17 to 0.73)	-0.22<br>(-1.19 to 0.75)	-0.16<br>(-1.51 to 1.18)	-0.18<br>(-1.03 to 0.67)	-0.10<br>(-1.09 to 0.87)	-0.12<br>(-1.04 to 0.78)	**NT**	0.70<br>(-1.16 to 2.57)
**-1.43**<br>**(-1.76 to -1.08)**	**-1.43**<br>**(-2.36 to -0.50)**	**-1.08**<br>**(-1.93 to -0.24)**	**-1.00**<br>**(-1.31 to -0.69)**	**-0.98**<br>**(-1.30 to -0.67)**	**-0.85**<br>**(-1.16 to -0.54)**	-0.72<br>(-1.59 to 0.14)	**-0.70**<br>**(-1.22 to -0.19)**	**-0.70**<br>**(-1.25 to -0.16)**	-0.64<br>(-1.70 to 0.41)	**-0.66**<br>**(-1.07 to -0.26)**	-0.59<br>(-1.19 to 0.01)	**-0.61**<br>**(-1.02 to -0.19)**	-0.49<br>(-1.31 to 0.34)	**WL**

eFigure 7. Sensitivity Network Meta-analyses

Sensitivity network meta-analysis for primary outcomes by omitting the trial with maternal anxiety disorder

Treatment Efficacy (mean overall change in symptoms at post-treatment, SMD [95% CrI]) Efficacy at follow-up (mean overall change in symptoms at follow-up, SMD [95% CrI])

Comparisons between treatments should be read from left to right, and the estimate is in the cell in common between the column-defining treatment and the row-defining treatment. For efficacy in post-treatment, standardized mean differences (SMDs) less than 0 favor the column-defining treatment. For efficacy at follow-up, standardized mean differences (SMDs) less than 0 favors the row-defining treatment. To obtain SMDs for comparisons in the opposite direction, negative values should be converted into positive values, and vice versa. To obtain ORs for comparisons in the opposing direction, reciprocals should be taken, and vice versa. Significant results are in bold and underlined. BIB-CBT=bibliotherapy cognitive-behavioral therapy, CI=confidence interval, CrI=credibility interval, G-BT=group behavioral therapy, G-CBT=group cognitive-behavioral therapy, G-CBT+P=group cognitive-behavioral therapy with parental involvement, I-BT+P=individual behavioral therapy with parental involvement, I-CBT=individual cognitive-behavioral therapy, I-CBT+P=individual cognitive-behavioral therapy with parental involvement, I+G-BT=individual and group behavioral therapy, I+G-CBT=individual and group cognitive-behavioral therapy, Int-CBT=internet-assisted cognitive-behavioral therapy, NT=no-treatment, OR=odds ratio, PBO=psychological placebo, P-CBT=parent-only cognitive-behavioral therapy, SMD=standardized mean difference, TAU=treatment as usual, WL=waitlist.

© 2018 Zhou X et al. JAMA Psychiatry.

**G-CBT**	-0.32<br>(-1.78 to 1.16)	-0.12<br>(-0.87 to 0.61)	-0.24<br>(-1.78 to 1.30)	-0.46<br>(-1.45 to 0.52)	-0.45<br>(-1.46 to 0.55)	0.04<br>(-1.45 to 1.53)	-0.06<br>(-1.73 to 1.60)		-0.68<br>(-1.59 to 0.22)	**-2.66**<br>**(-4.30 to -1.01)**	-0.28<br>(-2.12 to 1.56)	-0.33<br>(-1.47 to 0.79)		**-1.95**<br>**(-2.84 to -1.06)**
-0.18<br>(-1.16 to 0.80)	**G-BT**	0.19<br>(-1.46 to 1.83)	0.07<br>(-2.06 to 2.20)	-0.15<br>(-1.93 to 1.63)	-0.14<br>(-1.93 to 1.64)	0.36<br>(-1.74 to 2.44)	0.25<br>(-1.97 to 2.47)		-0.37<br>(-2.10 to 1.36)	**-2.34**<br>**(-4.55 to -0.14)**	0.03<br>(-2.32 to 2.39)	-0.02<br>(-1.88 to 1.83)		-1.64<br>(-3.35 to 0.09)
**-0.54**<br>**(-0.97 to -0.11)**	-0.37<br>(-1.36 to 0.63)	**G-CBT+P**	-0.12<br>(-1.73 to 1.50)	-0.34<br>(-1.41 to 0.74)	-0.33<br>(-1.42 to 0.77)	0.16<br>(-1.50 to 1.83)	0.06<br>(-1.43 to 1.55)		-0.56<br>(-1.59 to 0.49)	**-2.53**<br>**(-4.33 to -0.73)**	-0.16<br>(-2.04 to 1.74)	-0.21<br>(-1.30 to 0.88)		**-1.83**<br>**(-2.81 to -0.83)**
-0.52<br>(-1.42 to 0.38)	-0.35<br>(-1.62 to 0.93)	0.02<br>(-0.88 to 0.92)	**I-BT+P**	-0.22<br>(-1.59 to 1.14)	-0.21<br>(-1.54 to 1.11)	0.28<br>(-1.86 to 2.42)	0.18<br>(-2.02 to 2.37)		-0.44<br>(-1.78 to 0.89)	**-2.41**<br>**(-4.67 to -0.15)**	-0.04<br>(-2.11 to 2.02)	-0.09<br>(-1.68 to 1.51)		**-1.71**<br>**(-3.37 to -0.05)**
**-0.62**<br>**(-1.07 to -0.17)**	-0.44<br>(-1.45 to 0.57)	-0.08<br>(-0.54 to 0.38)	-0.10<br>(-0.96 to 0.76)	**I-CBT**	0.01<br>(-0.41 to 0.42)	0.51<br>(-1.28 to 2.28)	0.40<br>(-1.45 to 2.23)		-0.22<br>(-1.00 to 0.57)	**-2.19**<br>**(-4.11 to -0.28)**	0.18<br>(-1.37 to 1.74)	0.13<br>(-0.83 to 1.09)		**-1.49**<br>**(-2.66 to -0.31)**
**-0.74**<br>**(-1.19 to -0.28)**	-0.56<br>(-1.56 to 0.45)	-0.19<br>(-0.62 to 0.23)	-0.21<br>(-1.08 to 0.65)	-0.12<br>(-0.46 to 0.23)	**I-CBT+P**	0.50<br>(-1.30 to 2.29)	0.39<br>(-1.46 to 2.23)		-0.23<br>(-1.01 to 0.56)	**-2.20**<br>**(-4.13 to -0.28)**	0.17<br>(-1.43 to 1.79)	0.12<br>(-0.84 to 1.09)		**-1.50**<br>**(-2.69 to -0.31)**
-0.75<br>(-1.53 to 0.03)	-0.57<br>(-1.77 to 0.62)	-0.21<br>(-1.00 to 0.58)	-0.23<br>(-1.34 to 0.88)	-0.13<br>(-0.91 to 0.65)	-0.01<br>(-0.75 to 0.73)	**TAU**	-0.10<br>(-2.34 to 2.12)		-0.72<br>(-2.46 to 1.02)	**-2.70**<br>**(-4.92 to -0.48)**	-0.32<br>(-2.69 to 2.03)	-0.37<br>(-2.24 to 1.49)		**-1.99**<br>**(-3.72 to -0.26)**
**-0.87**<br>**(-1.54 to -0.20)**	-0.70<br>(-1.81 to 0.42)	-0.33<br>(-0.96 to 0.29)	-0.35<br>(-1.40 to 0.69)	-0.25<br>(-0.95 to 0.45)	-0.14<br>(-0.82 to 0.55)	-0.12<br>(-1.08 to 0.83)	**P-CBT**		-0.62<br>(-2.43 to 1.20)	**-2.59**<br>**(-4.92 to -0.25)**	-0.22<br>(-2.61 to 2.21)	-0.27<br>(-2.11 to 1.57)		**-1.89**<br>**(-3.67 to -0.10)**
-0.91<br>(-1.83 to 0.02)	-0.73<br>(-2.01 to 0.55)	-0.37<br>(-1.30 to 0.56)	-0.39<br>(-1.59 to 0.82)	-0.29<br>(-1.22 to 0.64)	-0.17<br>(-1.10 to 0.75)	-0.16<br>(-1.30 to 0.98)	-0.04<br>(-1.10 to 1.02)	**I+G-BT**						
**-0.88**<br>**(-1.33 to -0.44)**	-0.71<br>(-1.74 to 0.33)	-0.34<br>(-0.86 to 0.18)	-0.36<br>(-1.26 to 0.53)	-0.26<br>(-0.72 to 0.20)	-0.15<br>(-0.63 to 0.34)	-0.13<br>(-0.95 to 0.69)	-0.01<br>(-0.75 to 0.73)	0.03<br>(-0.88 to 0.93)	**PBO**	**-1.97**<br>**(-3.85 to -0.09)**	0.40<br>(-1.34 to 2.15)	0.35<br>(-0.76 to 1.46)		**-1.27**<br>**(-2.35 to -0.19)**
**-1.09**<br>**(-1.96 to -0.22)**	-0.91<br>(-2.22 to 0.40)	-0.54<br>(-1.52 to 0.43)	-0.56<br>(-1.82 to 0.68)	-0.47<br>(-1.45 to 0.51)	-0.35<br>(-1.34 to 0.63)	-0.34<br>(-1.51 to 0.83)	-0.21<br>(-1.32 to 0.88)	-0.18<br>(-1.45 to 1.09)	-0.20<br>(-1.19 to 0.77)	**NT**	2.37<br>(-0.09 to 4.84)	**2.32**<br>**(0.32 to 4.32)**		0.70<br>(-1.16 to 2.57)
**-1.02**<br>**(-1.58 to -0.46)**	-0.85<br>(-1.89 to 0.20)	-0.48<br>(-1.03 to 0.05)	-0.50<br>(-1.46 to 0.46)	-0.40<br>(-0.96 to 0.15)	-0.29<br>(-0.84 to 0.25)	-0.27<br>(-1.07 to 0.53)	-0.15<br>(-0.90 to 0.60)	-0.11<br>(-1.09 to 0.86)	-0.14<br>(-0.75 to 0.46)	0.06<br>(-0.97 to 1.10)	**Int-CBT**	-0.05<br>(-1.88 to 1.77)		-1.67<br>(-3.62 to 0.28)
**-1.05**<br>**(-1.75 to -0.36)**	-0.88<br>(-2.00 to 0.25)	-0.51<br>(-1.18 to 0.16)	-0.53<br>(-1.56 to 0.51)	-0.43'<br>(-1.10 to 0.23)	-0.32<br>(-0.97 to 0.34)	-0.30<br>(-1.25 to 0.65)	-0.18<br>(-1.03 to 0.68)	-0.14<br>(-1.21 to 0.92)	-0.17<br>(-0.91 to 0.56)	0.03<br>(-1.08 to 1.15)	-0.03<br>(-0.78 to 0.72)	**BIB-CBT**		**-1.62**<br>**(-2.92 to -0.32)**
-1.35<br>(-2.87 to 0.16)	-1.18<br>(-2.93 to 0.57)	-0.81<br>(-2.32 to 0.70)	-0.83<br>(-2.54 to 0.87)	-0.73<br>(-2.25 to 0.78)	-0.62<br>(-2.13 to 0.89)	-0.60<br>(-2.26 to 1.04)	-0.48<br>(-2.08 to 1.11)	-0.44<br>(-2.16 to 1.27)	-0.47<br>(-2.01 to 1.06)	-0.27<br>(-2.02 to 1.49)	-0.33<br>(-1.86 to 1.20)	-0.30<br>(-1.90 to 1.29)	**I+G-CBT**	
**-1.69**<br>**(-2.07 to -1.30)**	**-1.51**<br>**(-2.45 to -0.56)**	**-1.15**<br>**(-1.49 to -0.80)**	**-1.16**<br>**(-2.03 to -0.30)**	**-1.07**<br>**(-1.46 to -0.68)**	**-0.95**<br>**(-1.32 to -0.58)**	**-0.94**<br>**(-1.70 to -0.18)**	**-0.82**<br>**(-1.43 to -0.20)**	-0.78<br>(-1.66 to 0.10)	**-0.81**<br>**(-1.27 to -0.34)**	-0.60<br>(-1.55 to 0.36)	**-0.66**<br>**(-1.11 to -0.22)**	**-0.63**<br>**(-1.25 to -0.02)**	-0.33<br>(-1.80 to 1.14)	**WL**

Sensitivity network meta-analysis for primary outcomes by omitting trials rated as high risk of bias

Treatment Efficacy (mean overall change in symptoms at post-treatment, SMD [95% CrI]) Efficacy at follow-up (mean overall change in symptoms at follow-up, SMD [95% CrI])

Comparisons between treatments should be read from left to right, and the estimate is in the cell in common between the column-defining treatment and the row-defining treatment. For efficacy in post-treatment, standardized mean differences (SMDs) less than 0 favor the column-defining treatment. For efficacy at follow-up, standardized mean differences (SMDs) less than 0 favors the row-defining treatment. To obtain SMDs for comparisons in the opposite direction, negative values should be converted into positive values, and vice versa. To obtain ORs for comparisons in the opposing direction, reciprocals should be taken, and vice versa. Significant results are in bold and underlined. BIB-CBT=bibliotherapy cognitive-behavioral therapy, CI=confidence interval, CrI=credibility interval, G-BT=group behavioral therapy, G-CBT=group cognitive-behavioral therapy, G-CBT+P=group cognitive-behavioral therapy with parental involvement, I-BT+P=individual behavioral therapy with parental involvement, I-CBT=individual cognitive-behavioral therapy, I-CBT+P=individual cognitive-behavioral therapy with parental involvement, I+G-BT=individual and group behavioral therapy, I+G-CBT=individual and group cognitive-behavioral therapy, Int-CBT=internet-assisted cognitive-behavioral therapy, NT=no-treatment, OR=odds ratio, PBO=psychological placebo, P-CBT=parent-only cognitive-behavioral therapy, SMD=standardized mean difference, TAU=treatment as usual, WL=waitlist.

© 2018 Zhou X et al. JAMA Psychiatry.

eFigure 8. Contribution Matrix and Contributions Summary of Risk of Bias Assessments for Each Outcome

a. The contribution matrix for each outcome

The contributions (expressed as percentage) of direct comparisons (column) to the effect estimates of mixed or indirect comparisons (row) were calculated with the methods of Chaimani et al (PLoS ONE 8(10): e76654).

Abbreviation: BIB-CBT=bibliotherapy cognitive-behavioral therapy, G-BT=group behavioral therapy, G-CBT=group cognitive-behavioral therapy, G-CBT+P=group cognitive-behavioral therapy with parental involvement, I-BT+P=individual behavioral therapy with parental involvement, I-CBT=individual cognitive-behavioral therapy, I-CBT+P=individual cognitive-behavioral therapy with parental involvement, I+G-BT=individual and group behavioral therapy, I+G-CBT=individual and group cognitive-behavioral therapy, Int-CBT=internet-assisted cognitive-behavioral therapy, NT=no-treatment, PBO=psychological placebo, P-CBT=parent-only cognitive-behavioral therapy, TAU=treatment as usual, WL=waitlist.

© 2018 Zhou X et al. JAMA Psychiatry.

Mean overall change in symptoms at post-treatment

	Comparison	N of trials	WL vs BIB-CBT	WL vs P-CBT	WL vs G-BT	WL vs G-CBT	WL vs G-CBT+P	WL vs I+G-BT	WL vs I+G-CBT	WL vs I-BT+P	WL vs I-CBT	WL vs I-CBT+P	WL vs Int-CBT	WL vs PBO	BIB-CBT vs G-CBT+P	BIB-CBT vs I-CBT	BIB-CBT vs I-CBT+P	P-CBT vs G-CBT	P-CBT vs G-CBT+P	G-BT vs G-CBT	G-CBT vs G-CBT+P	G-CBT vs I-CBT	G-CBT vs NT	G-CBT vs PBO	G-CBT vs TAU	G-CBT+P vs I-CBT+P	G-CBT+P vs Int-CBT	G-CBT+P vs PBO	I+G-BT vs PBO	I-BT+P vs I-CBT	I-BT+P vs I-CBT+P	I-BT+P vs PBO	I-CBT vs I-CBT+P	I-CBT vs Int-CBT	I-CBT vs PBO	I-CBT vs TAU	I-CBT+P vs Int-CBT	I-CBT+P vs PBO	I-CBT+P vs TAU	Int-CBT vs PBO	Int-CBT vs TAU
Direct comparison	WL vs BIB-CBT	4	8.9	2	0.2	1.1	3.9	0.9	0	0.6	3.9	12.1	6.7	1.7	18.9	5.8	8.3	0	2.1	0.2	2.7	0.2	0	0.5	0.7	6.6	1.8	1.9	0.9	0.1	0.4	0.1	0.4	0.8	0.5	0.3	2.1	0.4	0.3	0.6	1.3
	WL vs P-CBT	5	2.3	8.7	0.2	1.5	4.1	0.8	0	0.5	2.9	9.4	6.1	1.6	3.5	0.6	0.7	5.9	23.5	0.2	0	1.1	0	1.6	1.5	11.2	2	2.6	0.8	0	0.2	0.3	1.1	0.5	0.6	0	1.4	0.4	0	0.6	1.5
	WL vs G-BT	2	1.6	1.9	2.5	2.3	2.8	0.8	0	0.4	2.5	7.5	5.4	1.4	2.1	0.2	0.3	2.3	0.4	26.6	12.6	2.5	0	4	2.9	6.1	1.2	0.9	0.8	0	0.1	0.3	0.9	0.4	0.8	0.3	1	1	0.3	0.6	2.3
	WL vs G-CBT	9	2.2	2.7	0.6	3.3	3.8	1.1	0	0.6	3.5	10.4	7.6	2	2.9	0.2	0.4	3.3	0.6	0.6	17.6	3.5	0	5.6	4	8.5	1.7	1.3	1.1	0	0.1	0.5	1.3	0.6	1.2	0.4	1.4	1.3	0.4	0.8	3.1
	WL vs G-CBT+P	13	3.3	3.2	0.3	1.6	6.1	1.2	0	0.7	4	13.6	8.4	2.1	5.3	0.9	1.1	0.2	3.4	0.3	5.3	0.9	0	1.3	1.4	17.3	3.1	4.2	1.2	0	0.4	0.3	2.4	0.7	0.7	0.1	2.1	0.3	0.2	0.8	1.7
	WL vs I+G-BT	2	1.1	0.9	0.1	0.7	1.7	26.9	0	0.6	2.5	7.1	4.5	2.8	0.7	0.3	0.2	0.3	0.7	0.1	0.6	0	0	2.1	0.4	0.4	0.6	3.5	22.1	0.1	0.6	1.2	2	0.5	5.4	0.2	1.1	5.5	0.1	1.6	0.8
	WL vs I+G-CBT	2	0	0	0	0	0	0	100	0	0	0	0	0	0	0	0	0	0	0	0	0	0	0	0	0	0	0	0	0	0	0	0	0	0	0	0	0	0	0	0
	WL vs I-BT+P	1	1.8	1.3	0.1	0.9	2.4	1.4	0	6.3	4.1	13.4	6.7	2.6	0.6	0.5	0.8	0.3	1.1	0.1	0.1	0.3	0	1.3	0.3	3.1	0.9	1.9	1.4	3.2	19.4	12.3	1	0.9	2.1	0.5	2.5	1.8	0.4	1.3	1.2
	WL vs I-CBT	9	2.4	1.6	0.2	1	2.9	1.3	0	0.9	8	16.2	8.7	2.3	0.2	1.9	0.7	0.3	1.3	0.2	0.1	1.4	0	0.4	0.2	4.1	1	0.7	1.3	0.4	0.4	0.1	24.9	2.1	5.2	1.4	2.8	0.5	0.3	0.9	1.9
	WL vs I-CBT+P	9	2.8	2	0.2	1.2	3.7	1.4	0	1.1	6.1	23.3	10	2.5	0.5	0.6	1.7	0.2	1.8	0.2	1.1	0.5	0	0.3	0.3	8.5	1.3	0	1.4	0.1	1.2	0.2	10.7	1.3	1.5	0.7	4.6	3.4	0.9	0.9	1.8
	WL vs Int-CBT	9	1.5	1.2	0.1	0.8	2.2	0.8	0	0.5	3.1	9.4	47.6	1.5	0.9	0.3	0.3	0.3	0.9	0.1	0.5	0.1	0	0.1	1.9	0.3	3.8	0	0.8	0	0.2	0.2	0.8	2.8	0	1.3	7.5	0.2	1.1	2.6	4.3
	WL vs PBO	1	2.1	1.7	0.2	1.2	3.1	2.8	0	1.1	4.6	13.2	8.4	5.2	1.3	0.5	0.3	0.5	1.2	0.2	1.1	0.1	0	3.8	0.7	0.8	1.1	6.4	2.8	0.1	1.1	2.3	3.8	0.9	10	0.4	2	10.2	0.2	2.9	1.4
	BIB-CBT vs G-CBT+P	1	8	1.3	0.1	0.6	2.6	0.2	0	0.1	0.1	0.9	1.8	0.4	33.3	6.9	10.1	0.2	1.6	0.1	3.1	0.9	0	0.9	0.8	13.4	1.5	2.8	0.2	0.1	0.1	0.2	3.6	0.2	1.7	0.3	0.1	0.9	0.1	0.2	0.4
	BIB-CBT vs I-CBT	1	6.4	0.5	0	0.1	1.2	0.2	0	0.2	3.4	2.7	1.3	0.4	18	7.3	8.6	0.3	0.9	0	2.7	1.5	0	0.9	0.6	9.9	0.9	2.5	0.2	0.3	0.1	0	21.7	1.1	4.1	0.9	0.5	0	0.1	0.2	0.4
	BIB-CBT vs I-CBT+P	2	7.7	0.6	0	0.2	1.2	0.1	0	0.2	1	6.8	1.2	0.3	22.2	7.2	11.1	0.2	0.8	0	2.1	0.7	0	0.8	0.6	15	0.9	2.2	0.1	0	0.6	0.3	9.9	0.2	0.7	0.2	1.7	2.5	0.5	0.1	0.1
	P-CBT vs G-CBT	1	0	7.1	0.4	2.2	0.3	0.3	0	0.1	0.7	1.3	1.9	0.6	0.7	0.4	0.3	10.8	27.1	0.4	21	2.9	0	4.7	3.1	3.2	0.4	1.5	0.3	0	0.1	0.2	2.9	0.1	0.7	0.5	0	1.2	0.5	0.2	2
	P-CBT vs G-CBT+P	3	1	10.2	0	0.1	1.9	0.3	0	0.2	1	3.7	2	0.5	1.8	0.4	0.4	9.7	44.9	0	7.6	0.6	0	1	0.5	6	1	1.7	0.3	0	0.1	0	1.5	0.2	0	0.2	0.6	0.3	0.2	0.2	0.1
	G-BT vs G-CBT	1	0.4	0.4	6	0.5	0.6	0.2	0	0.1	0.6	1.7	1.2	0.3	0.5	0	0.1	0.5	0.1	78.1	2.8	0.6	0	0.9	0.6	1.4	0.3	0.2	0.2	0	0	0.1	0.2	0.1	0.2	0.1	0.2	0.2	0.1	0.1	0.5
	G-CBT vs G-CBT+P	5	0.9	0	0.5	2.9	2	0.2	0	0	0.1	1.6	0.7	0.3	2.5	0.8	0.7	5.2	5.2	0.5	33.3	4.2	0	6.8	4.3	9.3	1.4	3.4	0.2	0	0.3	0.3	5	0	0.8	0.8	0.6	1.7	0.9	0.1	2.6
	G-CBT vs I-CBT	4	0.2	1.3	0.4	2.3	1.4	0	0	0.1	3.1	2.9	0.4	0.1	2.9	1.8	0.9	2.9	1.6	0.4	16.8	4.5	0	5.7	3.7	11.4	0.9	1.8	0	0.3	0.2	0.4	18.7	1.1	5.3	1.5	0.9	1.7	0.7	0	1.5
	G-CBT vs NT	4	0	0	0	0	0	0	0	0	0	0	0	0	0	0	0	0	0	0	0	0	100	0	0	0	0	0	0	0	0	0	0	0	0	0	0	0	0	0	0
	G-CBT vs PBO	8	0.4	1.3	0.4	2.4	1.3	1.4	0	0.4	0.6	1	0.4	2.7	1.9	0.7	0.8	3	1.7	0.4	17.8	3.7	0	9.5	3.6	8.3	0.8	7.3	1.4	0.2	1.1	1.6	2.1	0.2	8	0.9	0.4	8	0.7	1.8	2.1
	G-CBT vs TAU	1	0.7	1.3	0.3	2	1.6	0.3	0	0.1	0.2	1.3	8.6	0.6	1.9	0.5	0.6	2.3	1	0.3	13	2.8	0	4.2	11.9	7.4	2	1	0.3	0	0.1	0.3	1.4	0.8	1.6	5.4	2.2	1.3	4.5	1.1	14.7
	G-CBT+P vs I-CBT+P	4	1.3	2	0.1	0.8	3.9	0.1	0	0.2	1.3	7.1	0.3	0.1	6.2	1.7	3.2	0.5	2.4	0.1	5.5	1.7	0	1.9	1.4	30.6	2.5	5.2	0.1	0	0.8	0.6	8.1	0.5	2.5	0.5	2.1	3.8	0.7	0.1	0.2
	G-CBT+P vs Int-CBT	1	2.1	2.1	0.2	0.9	4.1	0.6	0	0.3	1.8	6.5	19.5	1	4.1	0.9	1.1	0.3	2.4	0.2	4.8	0.7	0	1	2.3	14.8	4.8	3.6	0.6	0	0.2	0.2	2.5	2.2	0.6	0.8	6.1	0.1	0.8	2.2	3.9
	G-CBT+P vs PBO	1	1.2	1.4	0.1	0.4	3	1.7	0	0.4	0.7	0.1	0.1	3.2	4.1	1.4	1.5	0.7	2.2	0.1	6.5	0.8	0	5.2	0.6	16.7	2	10.9	1.8	0.2	1.5	2	6.3	0.2	9.7	0.3	0.1	10.3	0.1	2.2	0.3
	I+G-BT vs PBO	1	1.2	1	0.1	0.7	1.7	22.9	0	0.6	2.6	7.4	4.7	2.9	0.7	0.3	0.2	0.3	0.7	0.1	0.6	0	0	2.1	0.4	0.4	0.6	3.6	24.3	0.1	0.6	1.3	2.1	0.5	5.6	0.2	1.1	5.7	0.1	1.6	0.8
	I-BT+P vs I-CBT	1	0.4	0.1	0	0.1	0.2	0.3	0	6.1	3.5	1.4	1.3	0.6	0.8	1.4	0.2	0.1	0.1	0	0.2	1.2	0	1	0.2	0.6	0	1.4	0.3	3.9	21.8	13.7	26.1	1.1	7.5	0.9	0.1	2.5	0.1	0.5	0.5
	I-BT+P vs I-CBT+P	1	0.4	0.2	0	0.1	0.5	0.5	0	7.3	0.7	5.7	1.2	1	0.2	0.1	0.7	0.1	0.3	0	1	0.2	0	1.4	0.1	4.5	0.2	2.6	0.5	4.3	27.2	16.3	9.6	0.1	4.3	0.1	1.4	5.8	0.3	0.7	0.2
	I-BT+P vs PBO	1	0.2	0.3	0.1	0.3	0.5	1.5	0	6.1	0.3	1.2	1.4	2.7	0.8	0.1	0.5	0.3	0.1	0.1	1.3	0.4	0	2.7	0.4	4.5	0.2	4.8	1.5	3.6	21.5	16.9	5.3	0	8.6	0.1	0.6	9.2	0.2	1.7	0.1
	I-CBT vs I-CBT+P	12	0	0.1	0	0.1	0.3	0.2	0	0	4.7	5.4	0.4	0.4	1	2.3	1.3	0.2	0.4	0	1.8	1.6	0	0.3	0.2	4.9	0.3	1.2	0.2	0.5	1	0.4	54.7	1.6	6.4	1.3	1.7	3.7	0.6	0.2	0.5
	I-CBT vs Int-CBT	1	1.2	0.7	0.1	0.4	1.3	0.6	0	0.5	5.2	8.6	19.8	1.1	0.7	1.5	0.4	0.1	0.6	0.1	0.1	1.3	0	0.4	1.2	3.7	3	0.6	0.6	0.3	0.2	0	21.1	3.4	4.5	1.9	6.8	0.5	0.9	2.3	4.1
	I-CBT vs PBO	7	0.2	0.2	0	0.2	0.3	1.8	0	0.3	3.5	2.7	0.1	3.4	1.7	1.6	0.3	0.2	0	0	1.1	1.7	0	3.9	0.7	5.3	0.2	6.5	1.8	0.6	1.6	2.5	22.8	1.2	16.9	1	0.8	12.1	0.1	2.3	0.5
	I-CBT vs TAU	1	0.5	0	0.1	0.3	0.2	0.3	0	0.2	3.4	4.2	9	0.5	1.1	1.2	0.3	0.6	0.6	0.1	3.9	1.7	0	1.5	8.1	4	1.1	0.8	0.3	0.2	0.1	0.1	17.3	1.9	3.7	6.9	3.2	0.4	5.2	1	16.2
	I-CBT+P vs Int-CBT	1	1.4	0.9	0.1	0.5	1.7	0.6	0	0.6	3.1	13.2	23	1.1	0.2	0.3	1.2	0	0.9	0.1	1.2	0.5	0	0.3	1.5	7.3	3.6	0.1	0.6	0	0.8	0.3	9.5	2.9	1.3	1.4	8.8	2.9	1.5	2.5	4.4
	I-CBT+P vs PBO	2	0.2	0.2	0.1	0.3	0.1	2.1	0	0.3	0.4	6.7	0.4	3.9	1.1	0	1.3	0.4	0.2	0.1	2.4	0.6	0	4.4	0.6	9.2	0.1	7.8	2.1	0.2	2.5	2.9	15	0.2	13.5	0.1	2	15.6	0.6	2.6	0.1
	I-CBT+P vs TAU	4	0.5	0.1	0.1	0.4	0.4	0.2	0	0.3	1.1	7.4	9.8	0.4	0.6	0.1	1	0.8	0.8	0.1	5.1	1	0	1.5	8.8	6.9	1.3	0.2	0.2	0	0.6	0.3	10.5	1.2	0.5	6.7	4.3	2.4	5.9	1	17.6
	Int-CBT vs PBO	1	1	0.9	0.1	0.6	1.5	2	0	0.7	2.4	6.4	19.4	3.8	0.6	0.3	0.1	0.3	0.6	0.1	0.7	0	0	3.4	1.7	0.5	3.1	5.7	2	0.1	1.1	1.9	2.9	2.4	8.8	1.1	6.1	9	0.8	4.1	3.7
	Int-CBT vs TAU	1	0.9	0.9	0.2	1	1.3	0.4	0	0.3	2	5.1	12.8	0.7	0.6	0.2	0.1	1	0.1	0.2	5.1	0.7	0	1.6	9.6	0.8	2.2	0.3	0.4	0.1	0.1	0.1	3	1.7	0.7	7	4.2	0.1	5.9	1.5	27.2
Indirect comparison	WL vs NT		1.6	2	0.4	2.4	2.8	0.8	0	0.4	2.5	7.6	5.5	1.5	2.1	0.2	0.3	2.4	0.4	0.4	12.8	2.6	27.4	4	2.9	6.1	1.2	0.9	0.8	0	0.1	0.3	0.9	0.4	0.8	0.3	1	1	0.3	0.6	2.3
	WL vs TAU		1.6	1.4	0.2	1.3	2.4	0.8	0	0.5	3.5	9.9	18	1.5	1	0.3	0.2	1	0.5	0.2	4.5	0.7	0	1.4	9	0.8	0.4	0.2	0.8	0.1	0.3	0.2	3	0.3	0.6	6.6	1	0	5.5	0.4	20
	BIB-CBT vs P-CBT		6	7.1	0.1	0.5	0.6	0	0	0.1	0.7	1.6	0	0	21.5	4.9	7.1	6.1	26.3	0.1	2.5	1	0	1.2	0.8	5.4	0.4	0.8	0	0	0.1	0.1	1.5	0.2	1.1	0.3	0.5	0.8	0.2	0	0.3
	BIB-CBT vs G-BT		4.6	0.5	2.4	1.6	0.1	0.1	0	0	0.2	0.9	0.8	0.3	15.5	3.9	5.6	2.4	1.9	27.2	14.7	2.4	0	3.7	2.4	1.5	0	0.4	0.1	0	0.2	0.2	0.7	0.1	1.2	0.6	0.5	1.3	0.5	0.1	1.3
	BIB-CBT vs G-CBT		6.1	0.9	0.4	2.4	0.4	0.3	0	0	0	0.5	1.7	0.5	21	5.3	7.5	3.5	2.6	0.4	21.1	3.5	0	5.4	3.5	2.7	0.1	0.5	0.3	0	0.3	0.4	1	0.1	1.7	0.8	0.5	1.8	0.7	0.2	2.1
	BIB-CBT vs I+G-BT		5.8	0.9	0.1	0.4	1.8	16.2	0	0.1	1.3	4.4	2	0.5	14.3	4.4	6.2	0.2	1.1	0.1	2.3	0.1	0	1.7	0.3	4.5	1	3.6	14.5	0.1	0.7	0.7	1	0.3	3.8	0.1	0.8	3.8	0.1	0.5	0.5
	BIB-CBT vs I+G-CBT		6.2	1.4	0.1	0.8	2.7	0.6	29.5	0.4	2.7	8.5	4.7	1.2	13.3	4.1	5.9	0	1.4	0.1	1.9	0.1	0	0.4	0.5	4.6	1.3	1.4	0.6	0	0.3	0.1	0.3	0.6	0.4	0.2	1.5	0.3	0.2	0.5	0.9
	BIB-CBT vs I-BT+P		5.9	0.6	0	0.2	1.2	0.4	0	4.9	0.3	1.3	0.1	0.8	16.4	5.3	7.6	0.2	0.8	0	2.2	0.4	0	1.5	0.3	8.2	0.8	3.3	0.4	2.8	16.9	10.7	1.2	0.1	2.2	0.1	0.4	1.9	0.1	0.5	0.1
	BIB-CBT vs Int-CBT		6.8	1.1	0.1	0.5	2.2	0.4	0	0.3	1.7	5.4	19	0.7	16.5	5.1	7.2	0.2	1.3	0.1	2.5	0.1	0	0.4	1.6	5.4	3.5	1.7	0.4	0	0.2	0	0.1	2.1	0.5	1	5.7	0.5	0.8	1.9	3.4

© 2018 Zhou X et al. JAMA Psychiatry.

BIB-CBT vs NT	4.3	0.6	0.3	1.7	0.3	0.2	0	0	0	0.3	1.2	0.4	15	3.8	5.3	2.5	1.8	0.3	15.1	2.5	28.5	3.9	2.5	1.9	0	0.4	0.2	0	0.2	0.3	0.7	0.1	1.2	0.6	0.4	1.3	0.5	0.2	1.5
BIB-CBT vs PBO	6.4	0.4	0	0.1	0.9	1.7	0	0.4	0.5	0.5	1.3	3.1	18.9	5.8	8.1	0.5	0.8	0	3.5	0.1	0	3.9	0	5.4	0.7	7.6	1.7	0.2	1.3	1.9	3	0.1	9.5	0.1	0.1	9.6	0	2	0
BIB-CBT vs TAU	5.6	0.4	0	0.2	1.1	0.1	0	0.1	0.2	1.5	9	0.1	15.4	4.7	6.6	0.8	1.2	0	5.6	0.4	0	0.7	7.7	4.4	1.7	1.3	0.1	0	0.1	0	2.1	0.8	0	5.4	2.4	0.3	4.5	0.8	14.6
P-CBT vs G-BT	0.1	5.2	2.5	1.3	0.4	0.1	0	0	0.3	0.3	0.9	0.3	0.7	0.3	0.2	7.5	19.3	29.5	13.9	1.8	0	3	1.9	2.8	0.4	1.2	0.1	0	0.1	0.1	2	0	0.4	0.4	0.1	0.7	0.4	0.1	1.2
P-CBT vs I+G-BT	1	6.1	0.1	0.7	2.1	15.2	0	0	0.7	2.9	1.9	0.5	2.2	0.6	0.6	4.6	18.2	0.1	0.4	0.8	0	2.5	0.9	8.2	1.2	4	13.6	0.1	0.5	0.5	2.1	0.1	2.7	0.1	0.4	3	0	0.5	0.7
P-CBT vs I+G-CBT	1.6	6.3	0.2	1.1	3	0.6	27.6	0.4	2.1	6.8	4.4	1.1	2.5	0.4	0.5	4.2	17	0.2	0	0.8	0	1.2	1.1	8.1	1.5	1.9	0.6	0	0.2	0.2	0.8	0.4	0.4	0	1	0.3	0	0.4	1.1
P-CBT vs I-BT+P	0.5	6.2	0.1	0.5	1.5	0.4	0	4.4	0.8	2.5	0.1	0.7	2.5	0.8	1.2	5	20.2	0.1	0	1.1	0	2.3	0.9	11.6	1	3.6	0.4	2.4	15	9.6	0.2	0.3	1.1	0.4	0.7	1.1	0.3	0.5	0.3
P-CBT vs I-CBT	0.2	6.4	0.1	0.5	1.5	0.2	0	0.2	3.4	3.6	1	0.3	3.2	1.9	1.1	5.3	21.3	0.1	0.1	2	0	1.7	1.2	12.7	1.1	2.7	0.2	0.3	0.1	0.1	19.1	1.1	4.3	1	0.8	0.7	0.2	0.1	0.1
P-CBT vs I-CBT+P	0.3	7.5	0.1	0.6	1.6	0.1	0	0.2	1.5	7	1	0.2	3.2	1	1.9	6.1	25.2	0.1	0.8	1.5	0	1.9	1.3	17.5	1.1	2.6	0.1	0	0.6	0.4	6.5	0.4	1.7	0.5	1.8	2.8	0.6	0.1	0.2
P-CBT vs Int-CBT	1.2	6.9	0.1	0.9	2.5	0.3	0	0.2	1	3.6	17.5	0.6	2.6	0.6	0.7	5.2	20.5	0.1	0.3	0.9	0	1.4	2.1	9.5	3.5	2.2	0.3	0	0.1	0.1	1.4	1.8	0.5	0.6	4.8	0.2	0.6	1.8	3.3
P-CBT vs NT	0	4.9	0.3	1.5	0.2	0.2	0	0.1	0.5	0.9	1.3	0.4	0.5	0.3	0.2	7.5	18.7	0.3	14.5	2	31	3.2	2.1	2.2	0.3	1.1	0.2	0	0.1	0.2	2	0.1	0.5	0.4	0	0.8	0.4	0.2	1.4
P-CBT vs PBO	0.4	6.5	0.1	0.4	1.3	1.5	0	0.4	1.1	1.9	1.1	2.7	2.1	0.9	0.9	5.7	22.1	0.1	0.9	0.9	0	4.5	0.7	9.5	1	7.4	1.5	0.1	1	1.6	4	0.3	7.3	0.3	0.3	7.7	0.2	1.7	0.2
P-CBT vs TAU	0.6	5.9	0	0.2	1.5	0.1	0	0	0.3	0.2	8.5	0.1	2	0.7	0.7	5.4	19.1	0	3.4	0.4	0	0.3	7.9	8.3	1.9	1.9	0.1	0	0	0.1	3.1	0.6	0.9	4.9	1.9	0.3	4.1	0.7	13.6
G-BT vs G-CBT+P	0.7	0.2	3	1.6	1.6	0	0	0	0.2	1.8	0.1	0	1.8	0.5	0.5	3.1	3.3	34.1	20.3	2.5	0	4	2.5	6.6	1	2.3	0	0	0.2	0.2	3.1	0.1	0.5	0.5	0.5	1	0.5	0	1.5
G-BT vs I+G-BT	0.8	1.2	2.1	1.6	1.5	12.9	0	0.1	0.8	2.7	2.3	0.2	1.4	0.3	0.4	1.8	0.7	22.5	10.3	2.1	0	4.4	2.2	4.9	0.7	2.5	11.7	0.1	0.4	0.3	0.2	0.1	2	0.4	0.3	2	0.3	0.3	1.5
G-BT vs I+G-CBT	1.2	1.5	1.9	1.8	2.1	0.6	22.6	0.3	1.9	5.8	4.2	1.1	1.6	0.1	0.2	1.8	0.3	20.6	9.7	2	0	3.1	2.2	4.7	0.9	0.7	0.6	0	0.1	0.3	0.7	0.3	0.6	0.2	0.8	0.7	0.2	0.4	1.7
G-BT vs I-BT+P	0.3	0.9	2.1	1.5	0.9	0.2	0	3.5	0.4	1.8	0.6	0.4	1.5	0.4	0.8	1.9	1	23.6	11.2	2.4	0	4.3	2.3	7.3	0.5	2	0.2	2	12.2	8	1.4	0.2	0.6	0.6	0.7	0.3	0.5	0.3	1.2
G-BT vs I-CBT	0.1	0.9	2.3	1.6	0.9	0	0	0.1	2.6	2.8	0.1	0	2.1	1.3	0.7	2	1.2	25.3	11.8	3.3	0	4	2.6	8.2	0.6	1.3	0	0.2	0.2	0.2	14.3	0.9	4	1.1	0.8	1.2	0.5	0	1
G-BT vs I-CBT+P	0.1	0.9	2.5	1.7	0.8	0.1	0	0.1	0.8	5.2	0.1	0.1	1.8	0.5	1.3	2.3	1.4	27.5	13.6	2.9	0	4.3	2.8	10.9	0.5	0.9	0.1	0	0.6	0.4	6.9	0.3	1.7	0.7	1.5	2.8	0.8	0.1	1.3
G-BT vs Int-CBT	0.9	1.3	2.2	1.8	1.7	0.4	0	0.2	1.1	3.2	13.6	0.7	1.5	0.2	0.4	2	0.7	24.6	11.4	2.3	0	3.6	3.4	5.5	2.6	0.8	0.4	0	0	0.2	0.5	1.5	0.8	0.2	3.9	0.8	0.2	1.5	3.7
G-BT vs NT	0.2	0.2	3.3	0.3	0.3	0.1	0	0.1	0.3	0.9	0.7	0.2	0.3	0	0	0.3	0.1	42.4	1.5	0.3	45.7	0.5	0.4	0.7	0.1	0.1	0.1	0	0	0	0.1	0.1	0.1	0	0.1	0.1	0	0.1	0.3
G-BT vs PBO	0.2	0.8	2.4	1.5	0.7	1.1	0	0.3	0.6	1.4	0.2	2.1	1.2	0.5	0.6	2	1.2	27.2	12	2.5	0	6.6	2.4	5.6	0.5	5.3	1.1	0.1	0.8	1.2	1.6	0.2	5.9	0.6	0.4	5.9	0.5	1.4	1.3
G-BT vs TAU	0.4	0.9	2.2	1.3	1	0.2	0	0.1	0	0.4	7	0.4	1.3	0.4	0.5	1.6	0.7	25.7	9	2	0	2.9	8.9	5.2	1.4	0.7	0.2	0	0.1	0.2	1.2	0.6	1.2	4.2	1.6	0.9	3.5	0.8	11.4
G-CBT vs I+G-BT	1.1	1.6	0.4	2.2	2.1	16	0	0.1	1.2	4	3.3	0.1	1.9	0.3	0.5	2.5	0.9	0.4	14	2.8	0	5.8	3	6.6	1	3.2	14.8	0.1	0.4	0.4	0.2	0.2	2.5	0.5	0.4	2.4	0.4	0.3	2.1
G-CBT vs I+G-CBT	1.6	2	0.4	2.4	2.8	0.8	27.4	0.4	2.5	7.6	5.5	1.5	2.1	0.2	0.3	2.4	0.4	0.4	12.8	2.6	0	4	2.9	6.1	1.2	0.9	0.8	0	0.1	0.3	0.9	0.4	0.8	0.3	1	1	0.3	0.6	2.3
G-CBT vs I-BT+P	0.5	1.3	0.4	2.2	1.4	0.2	0	4.5	0.3	1.7	1.3	0.3	2.1	0.6	1	2.7	1.4	0.4	15.6	3.3	0	5.9	3.3	10	0.8	2.7	0.2	2.6	15.8	10.4	1.9	0.2	0.6	0.8	0.8	0.3	0.7	0.3	1.8
G-CBT vs I-CBT+P	0.3	1.4	0.4	2.6	1.4	0.2	0	0.2	0.9	6.2	0.7	0.3	2.7	0.7	1.7	3.3	1.9	0.4	19.6	4.1	0	6.1	4	15.3	0.8	1.3	0.2	0	0.8	0.6	9.3	0.3	2.3	1	1.9	3.9	1.1	0.1	2
G-CBT vs Int-CBT	1.3	1.9	0.4	2.6	2.4	0.6	0	0.3	1.6	4.8	16.9	1.1	2.2	0.3	0.5	2.8	1	0.4	15.8	3.2	0	5	4.6	7.5	3.4	1.2	0.6	0	0	0.3	0.8	1.9	1	0.2	5	1.1	0.2	2	5
G-CBT+P vs I+G-BT	1.7	1.7	0.1	0.8	3.4	17.3	0	0.1	1.3	5.2	3.2	0.3	3.5	0.8	0.9	0.3	2.1	0.1	4.3	0.6	0	2.3	0.8	12.5	1.9	5.4	15.8	0.1	0.7	0.6	3.1	0.2	3.1	0.1	0.8	3.5	0.1	0.5	0.7
G-CBT+P vs I+G-CBT	2.3	2.2	0.2	1.1	4.2	0.8	30.9	0.5	2.8	9.4	5.8	1.5	3.7	0.6	0.8	0.1	2.3	0.2	3.6	0.6	0	0.9	1	11.9	2.1	2.9	0.8	0	0.3	0.2	1.6	0.5	0.5	0.1	1.5	0.2	0.1	0.6	1.2
G-CBT+P vs I-BT+P	1.2	1.5	0.1	0.6	3.1	0.3	0	5.4	0.4	0.9	1.1	0.6	4.2	1.3	1.7	0.4	1.9	0.1	4.6	1	0	2.3	0.9	18.3	1.9	5.6	0.3	3.1	19	12.1	1.1	0.2	1.3	0.3	0.5	1.5	0.2	0.5	0.3
G-CBT+P vs I-CBT	1	1.6	0.1	0.6	3.2	0.1	0	0.1	3.8	2.3	0.1	0.1	5.4	2.7	1.7	0.5	2.1	0.1	5.3	2.3	0	1.6	1.2	20.7	2.1	4.7	0.1	0.3	0	0.2	26.2	1.3	5.7	1.2	0.6	0.7	0.2	0.1	0.1
G-CBT+P vs NT	0.6	0	0.3	1.8	1.3	0.1	0	0	0	1	0.4	0.2	1.6	0.5	0.5	3.3	3.3	0.3	21.2	2.7	36.3	4.3	2.7	5.9	0.9	2.2	0.1	0	0.2	0.2	3.2	0	0.5	0.5	0.4	1.1	0.5	0.1	1.6
G-CBT+P vs TAU	1.3	1.3	0	0.1	2.8	0.2	0	0.1	0.2	2.3	8.8	0.4	3.4	1	1.1	1	2.3	0	8.1	0.1	0	0.2	8.9	13.1	2.8	3.1	0.2	0	0.1	0.1	4.5	0.8	1.1	5.8	2.6	0.2	4.9	1	15.9
I+G-BT vs I+G-CBT	0.8	0.6	0.1	0.4	1.1	18.1	32.9	0.4	1.7	4.8	3	1.9	0.5	0.2	0.1	0.2	0.4	0.1	0.4	0	0	1.4	0.3	0.3	0.4	2.3	14.8	0	0.4	0.8	1.4	0.3	3.6	0.2	0.7	3.7	0.1	1.1	0.5
I+G-BT vs I-BT+P	0.7	0.5	0	0.3	0.9	18.2	0	5	1.7	6.4	2.5	0.2	0	0.2	0.5	0	0.5	0	0.5	0.3	0	0.4	0	3	0.3	0.8	17.1	2.7	16.2	11.5	2.3	0.4	2.1	0.2	1.3	2.4	0.3	0.1	0.5
I+G-BT vs I-CBT	1	0.5	0.1	0.3	1	17.9	0	0.2	4.3	7.2	3.4	0.2	0.6	1.3	0.4	0.1	0.5	0.1	0.3	1.1	0	1.1	0.2	3.4	0.3	1.9	16.4	0.3	0.7	0.8	17.4	1.2	7.7	0.9	1.4	4.2	0.2	0.4	0.9
I+G-BT vs I-CBT+P	1.1	0.7	0.1	0.3	1.3	19.5	0	0.3	2.3	10.6	3.4	0.4	0.2	0.2	1	0.1	0.7	0.1	1.2	0.4	0	1.4	0.1	6.1	0.5	2.7	17.7	0.1	1.3	1.1	8.9	0.5	5.1	0.3	2.3	6.5	0.5	0.6	0.7
I+G-BT vs Int-CBT	0.1	0.1	0	0.1	0.1	21	0	0.2	0.2	0.3	23.5	1.4	0	0.1	0	0	0	0	0.2	0	0	1.7	1.4	0.2	2.6	2.7	18.1	0.1	0.6	0.9	1.2	2	4.3	0.9	5.2	4.3	0.7	2.8	3
I+G-BT vs NT	0.8	1.2	0.3	1.7	1.6	12.2	0	0.1	1	3.1	2.6	0.1	1.5	0.3	0.4	1.9	0.7	0.3	10.7	2.2	23.5	4.5	2.3	5.1	0.8	2.4	11.3	0.1	0.3	0.3	0.2	0.1	1.9	0.4	0.3	1.8	0.3	0.3	1.6
I+G-BT vs TAU	0.5	0.5	0.1	0.6	0.7	15.9	0	0	1	2.8	10.4	0.6	0.3	0.1	0.1	0.6	0.1	0.1	2.9	0.5	0	2.3	6.9	0.4	0.7	2.3	14.2	0.1	0.5	0.6	1	0.5	3.7	4.9	1.4	3.4	4.1	1.2	14.2
I+G-CBT vs I-BT+P	1.3	1	0.1	0.6	1.7	1	29.1	4.4	2.9	9.5	4.7	1.8	0.4	0.3	0.5	0.2	0.8	0.1	0.1	0.2	0	0.9	0.2	2.2	0.6	1.4	1	2.2	13.7	8.7	0.7	0.6	1.5	0.3	1.7	1.3	0.3	0.9	0.9
I+G-CBT vs I-CBT	1.6	1.1	0.1	0.7	2	0.9	31.2	0.6	5.5	11.1	6	1.6	0.1	1.3	0.5	0.2	0.9	0.1	0.1	1	0	0.3	0.1	2.8	0.7	0.5	0.9	0.3	0.3	0.1	17.2	1.4	3.6	0.9	2	0.3	0.2	0.6	1.3
I+G-CBT vs I-CBT+P	1.8	1.3	0.1	0.8	2.4	0.9	35.2	0.7	4	15.1	6.5	1.6	0.3	0.4	1.1	0.1	1.1	0.1	0.7	0.3	0	0.2	0.2	5.5	0.9	0	0.9	0	0.8	0.1	6.9	0.8	1	0.4	3	2.2	0.6	0.6	1.2
I+G-CBT vs Int-CBT	0.9	0.7	0.1	0.5	1.3	0.5	40.7	0.3	1.8	5.6	28.2	0.9	0.5	0.1	0.2	0.2	0.5	0.1	0.3	0	0	0.1	1.1	0.2	2.2	0	0.5	0	0.1	0.1	0.5	1.7	0	0.8	4.5	0.1	0.7	1.6	2.5
I+G-CBT vs NT	1.3	1.5	0.3	1.9	2.2	0.6	21.5	0.3	2	5.9	4.3	1.1	1.6	0.1	0.2	1.9	0.3	0.3	10	2	21.5	3.2	2.3	4.8	1	0.7	0.6	0	0.1	0.3	0.7	0.3	0.7	0.3	0.8	0.8	0.2	0.4	1.8
I+G-CBT vs PBO	1.5	1.2	0.1	0.8	2.2	2	30.4	0.8	3.2	9.2	5.8	3.6	0.9	0.3	0.2	0.4	0.9	0.1	0.7	0.1	0	2.7	0.5	0.5	0.8	4.5	2	0.1	0.7	1.6	2.6	0.6	7	0.3	1.4	7.1	0.2	2	1
I+G-CBT vs TAU	1.1	1	0.2	0.9	1.7	0.6	29.1	0.4	2.5	7	12.7	1.1	0.7	0.2	0.2	0.7	0.3	0.2	3.2	0.5	0	1	6.4	0.6	0.3	0.2	0.6	0.1	0.2	0.1	2.1	0.2	0.4	4.7	0.7	0	3.9	0.3	14.2
I-BT+P vs Int-CBT	0.8	0.5	0.1	0.3	1	0.8	0	5.1	2	6.7	18.7	1.5	0	0.3	0.5	0.1	0.5	0.1	0.3	0.3	0	1.1	1.3	2.8	2.7	1.6	0.8	2.7	16.7	10.6	1.3	2.2	1.7	1.1	6	1.4	0.9	2.4	3.2
I-BT+P vs NT	0.4	1	0.3	1.6	1.1	0.1	0	3.4	0.2	1.3	1	0.3	1.6	0.4	0.8	2	1	0.3	11.7	2.5	25	4.4	2.4	7.5	0.6	2	0.1	1.9	11.8	7.8	1.5	0.2	0.5	0.6	0.6	0.2	0.5	0.2	1.3
I-BT+P vs TAU	0.2	0.1	0.1	0.3	0.1	0.4	0	4.3	0.5	2.6	8.4	0.8	0.4	0.1	0.4	0.5	0.5	0.1	3.4	0.7	0	2	7	3	1	1.6	0.4	2.4	14.7	9.3	3	0.9	2	5.3	2.6	1.3	4.4	1.2	14
I-CBT vs NT	0.2	1	0.3	1.7	1	0	0	0.1	2.3	2.1	0.3	0.1	2.2	1.3	0.7	2.1	1.1	0.3	12.3	3.3	26.7	4.2	2.7	8.4	0.6	1.3	0	0.2	0.1	0.3	13.7	0.8	3.9	1.1	0.7	1.2	0.5	0	1.1
I-CBT+P vs NT	0.2	1	0.3	1.8	1	0.1	0	0.1	0.6	4.4	0.5	0.2	1.9	0.5	1.2	2.4	1.4	0.3	13.9	2.9	28.7	4.4	2.9	10.9	0.6	0.9	0.1	0	0.6	0.4	6.6	0.2	1.7	0.7	1.4	2.8	0.8	0.1	1.4
Int-CBT vs NT	1	1.4	0.3	1.9	1.8	0.4	0	0.2	1.2	3.6	12.6	0.8	1.6	0.2	0.4	2.1	0.7	0.3	11.7	2.4	25.6	3.7	3.4	5.6	2.5	0.9	0.4	0	0	0.2	0.6	1.4	0.8	0.2	3.8	0.8	0.1	1.5	3.7
NT vs PBO	0.3	0.9	0.3	1.7	0.9	1	0	0.3	0.4	0.7	0.3	1.9	1.4	0.5	0.6	2.1	1.2	0.3	12.7	2.6	28.7	6.8	2.6	5.9	0.6	5.2	1	0.1	0.8	1.2	1.5	0.2	5.7	0.6	0.3	5.7	0.5	1.3	1.5
NT vs TAU	0.5	1	0.2	1.4	1.2	0.2	0	0.1	0.2	1	6.3	0.4	1.4	0.4	0.5	1.7	0.7	0.2	9.5	2.1	26.7	3.1	8.7	5.4	1.5	0.8	0.2	0	0.1	0.2	1	0.6	1.2	4	1.6	0.9	3.3	0.8	10.8
PBO vs TAU	0.3	0.2	0	0.2	0.5	1.6	0	0.5	0.8	2.3	8.9	3	0.2	0.1	0.1	0.4	0.6	0	3.1	0.5	0	4.4	8.6	0.1	1.3	5.5	1.6	0.2	1.1	1.8	0.5	1	8.9	6.2	2.6	8.5	5.1	2.7	16.6

© 2018 Zhou X et al. JAMA Psychiatry.

Total		1.6	1.8	0.5	1.1	1.6	3	4.8	0.9	1.7	4.8	5.7	1	3.7	1.2	1.5	1.9	3.9	4.5	6.9	1.5	4.8	2.7	2.6	6.4	1.2	2.3	2.8	0.4	2.6	1.9	4.8	0.7	2.6	1.2	1.7	2.3	1	0.8	3.4

© 2018 Zhou X et al. JAMA Psychiatry.

Mean overall change in symptoms at follow-up

	Comparison	N of trials	WL vs G-CBT	WL vs G-CBT+P	WL vs I-CBT	WL vs PBO	WL vs P-CBT	BIB-CBT vs G-CBT+P	BIB-CBT vs I-CBT	BIB-CBT vs I-CBT+P	G-BT vs G-CBT	G-CBT vs G-CBT+P	G-CBT vs I-CBT	G-CBT vs NT	G-CBT vs PBO	G-CBT vs TAU	G-CBT+P vs PBO	I-BT+P vs I-CBT+P	I-BT+P vs PBO	I-CBT vs I-CBT+P	I-CBT vs Int-CBT	I-CBT vs PBO	I-CBT vs TAU	I-CBT+P vs PBO	P-CBT vs G-CBT+P
Direct comparison	WL vs G-CBT	2	14.7	6.9	6.1	11.7	5.8	0.7	0.1	0.6	0	16.7	7.8	0	2	4.1	3.2	0.5	0.5	2.6	0	3.4	4.1	2.6	5.8
	WL vs G-CBT+P	1	9.1	10.2	6.6	13.7	8.5	8.3	3.6	4.7	0	9.3	0.1	0	0.1	0.1	11.7	0.3	0.3	3.2	0	0.4	0.1	1.2	8.5
	WL vs I-CBT	1	7.1	5.8	7.7	12.9	4.9	7.2	3.6	3.6	0	0.2	4.2	0	0.3	2.2	3.8	1.3	1.3	11.1	0	9.5	2.2	6.2	4.9
	WL vs PBO	1	7.5	6.8	7.2	17.9	5.7	4	1.3	2.7	0	2.2	2.8	0	1.1	1.5	10.6	1.3	1.3	4.9	0	7.9	1.5	6.3	5.7
	WL vs P-CBT	1	5.6	6.2	4	8.4	25	5.1	2.2	2.9	0	5.7	0.1	0	0	0	7.2	0.2	0.2	2	0	0.3	0	0.8	24.2
	BIB-CBT vs G-CBT+P	1	0.2	1.4	1.4	1.4	1.2	46.1	6.4	8.8	0	4.2	2.5	0	0.3	1.3	8.4	1	1	3	0	4.2	1.3	4.8	1.2
	BIB-CBT vs I-CBT	1	0.1	1.6	1.8	1.2	1.3	17	11.7	14.6	0	5.1	3.2	0	0.2	1.7	8.9	0.5	0.5	17.6	0	7.3	1.7	2.5	1.3
	BIB-CBT vs I-CBT+P	2	0.2	1.5	1.2	1.7	1.2	16.3	10	17.2	0	4.3	2.4	0	0.4	1.3	9.3	1.5	1.5	17.7	0	2.7	1.3	7.1	1.2
	G-BT vs G-CBT	1	0	0	0	0	0	0	0	0	100	0	0	0	0	0	0	0	0	0	0	0	0	0	0
	G-CBT vs G-CBT+P	3	6.8	2.9	0.1	1.4	2.4	7.9	3.6	4.3	0	28.2	8.5	0	2.1	4.5	8.7	0.3	0.3	6.2	0	3.3	4.5	1.6	2.4
	G-CBT vs I-CBT	3	5.8	0.1	2.7	3.2	0.1	8.4	4	4.4	0	15.5	11.7	0	2.2	6.2	7	0.9	0.9	9.6	0	7.1	6.2	4.3	0.1
	G-CBT vs NT	1	0	0	0	0	0	0	0	0	0	0	0	99.9	0	0	0	0	0	0	0	0	0	0	0
	G-CBT vs PBO	2	6.3	0.1	1	5.5	0.1	4.2	1.3	2.9	0	16.6	9.4	0	2.7	4.9	12.2	1.7	1.7	6.6	0	10	4.9	7.8	0.1
	G-CBT vs TAU	1	3	0	1.4	1.7	0	4.4	2.1	2.3	0	8.1	6.2	0	1.1	35.4	3.7	0.5	0.5	5.1	0	3.7	18.4	2.3	0
	G-CBT+P vs PBO	1	1	2.9	1.1	5.4	2.5	12.5	5	7.6	0	7	3.1	0	1.2	1.6	23	1.7	1.7	2	0	8.8	1.6	7.9	2.5
	I-BT+P vs I-CBT+P	1	0.3	0.1	0.7	1.2	0.1	2.8	0.5	2.2	0	0.5	0.7	0	0.3	0.4	3	38.2	21.6	9.8	0	7.5	0.4	9.6	0.1
	I-BT+P vs PBO	1	0.4	0.1	0.8	1.5	0.1	3.5	0.7	2.8	0	0.6	0.9	0	0.4	0.5	3.8	27.2	22	12.3	0	9.4	0.5	12.1	0.1
	I-CBT vs I-CBT+P	12	0.3	0.3	1	0.8	0.2	1.4	3.1	4.6	0	1.6	1.4	0	0.2	0.7	0.6	1.7	1.7	63	0	8.2	0.7	8.2	0.2
	I-CBT vs Int-CBT	1	0	0	0	0	0	0	0	0	0	0	0	0	0	0	0	0	0	0	100	0	0	0	0
	I-CBT vs PBO	2	0.9	0.1	2.2	3.3	0.1	5.2	3.4	1.8	0	2.2	2.6	0	0.8	1.4	7.3	3.4	3.4	21.4	0	22.6	1.4	16.2	0.1
	I-CBT vs TAU	1	3.7	0	1.7	2	0	5.3	2.5	2.8	0	9.9	7.5	0	1.4	22.4	4.4	0.6	0.6	6.1	0	4.5	21.7	2.8	0
	I-CBT+P vs PBO	2	0.7	0.3	1.5	2.7	0.2	6.2	1.2	5	0	1.1	1.7	0	0.7	0.9	6.8	4.6	4.6	22.1	0	16.8	0.9	21.7	0.2
	P-CBT vs G-CBT+P	1	4.5	5	3.2	6.8	19.5	4.1	1.8	2.3	0	4.6	0.1	0	0	0	5.8	0.1	0.1	1.6	0	0.2	0	0.6	39.5
Indirect comparison	WL vs BIB-CBT		6.6	6.7	5.6	10.9	5.6	20.6	6.3	8.6	0	4.5	1.3	0	0.1	0.7	3.8	0.8	0.8	4.1	0	2.8	0.7	3.7	5.6
	WL vs G-BT		10.1	4.8	4.2	8	4	0.5	0.1	0.4	31.1	11.5	5.4	0	1.3	2.8	2.2	0.4	0.4	1.8	0	2.3	2.8	1.8	4
	WL vs I-BT+P		5.3	4.6	5.3	11.1	3.9	4.8	1.3	3.5	0	1.1	2.4	0	0.5	1.3	4.8	17.6	12.6	10.9	0	0.5	1.3	3.2	3.9
	WL vs I-CBT+P		6.2	5.3	6.4	11.8	4.5	7	1.8	5.2	0	0.9	3.2	0	0.4	1.7	3.7	1.9	1.9	17.9	0	4.8	1.7	9.2	4.5
	WL vs Int-CBT		5.1	4.2	5.6	9.3	3.5	5.2	2.6	2.6	0	0.2	3.1	0	0.3	1.6	2.7	0.9	0.9	8	27.7	6.8	1.6	4.5	3.5
	WL vs NT		10.1	4.8	4.2	8	4	0.5	0.1	0.4	0	11.5	5.4	31.1	1.3	2.8	2.2	0.4	0.4	1.8	0	2.3	2.8	1.8	4
	WL vs TAU		9	5.1	5.4	9.7	4.3	2.2	1.2	1	0	7.3	2	0	0.7	18.9	0.1	0.7	0.7	5	0	4.8	14.5	3.3	4.3
	BIB-CBT vs P-CBT		2.7	2.3	2.8	5	12.7	24.8	4.8	6.7	0	0.3	1.4	0	0.2	0.8	1.4	0.7	0.7	2.8	0	2.6	0.8	3.2	23.6
	BIB-CBT vs G-BT		3.9	1	0.7	1.4	0.8	16.7	4.9	6.5	28.1	13.9	5.9	0	1.3	3.1	1	0.3	0.3	4.9	0	0.1	3.1	1.3	0.8
	BIB-CBT vs G-CBT		5.4	1.4	0.9	1.9	1.2	23.3	6.8	9	0	19.3	8.2	0	1.8	4.3	1.4	0.4	0.4	6.8	0	0.1	4.3	1.8	1.2
	BIB-CBT vs I-BT+P		0.4	1.2	0.6	2	1	14.3	7.5	12.2	0	3.1	1.5	0	0.5	0.8	9	20.5	13.4	8.2	0	2.1	0.8	0.1	1
	BIB-CBT vs Int-CBT		0.1	1.1	1.3	0.8	0.9	11.9	8.2	10.2	0	3.6	2.2	0	0.2	1.2	6.2	0.4	0.4	12.3	30.2	5.1	1.2	1.8	0.9
	BIB-CBT vs NT		3.9	1	0.7	1.4	0.8	16.7	4.9	6.5	0	13.9	5.9	28.1	1.3	3.1	1	0.3	0.3	4.9	0	0.1	3.1	1.3	0.8
	BIB-CBT vs PBO		0.8	1.7	0	3.9	1.4	21.2	9	13.1	0	3.4	1	0	0.9	0.6	14.8	2.2	2.2	0.3	0	10.9	0.6	10.5	1.4
	BIB-CBT vs TAU		2.6	1.2	0.1	0.6	1	16.5	6.9	8.8	0	11	3.1	0	0.8	17.6	3.4	0	0	8.6	0	2.1	14.6	0.1	1
	P-CBT vs G-BT		5.7	0.7	1.4	2.3	10.1	2.3	1.1	1.2	26.8	13	4.6	0	1.1	2.4	2	0.2	0.2	2.6	0	1.9	2.4	1.1	16.6
	P-CBT vs G-CBT		7.7	1	1.9	3.2	13.9	3.2	1.5	1.7	0	17.7	6.3	0	1.6	3.3	2.8	0.3	0.3	3.5	0	2.6	3.3	1.5	22.7
	P-CBT vs I-BT+P		1.6	0.7	2.4	5	9.7	6.7	2.3	4.5	0	2.1	2.1	0	0.5	1.1	7.8	15	10.6	8.2	0	0.3	1.1	2.3	15.9
	P-CBT vs I-CBT		2.1	0.8	3.6	4.9	11.4	8.7	4.1	4.6	0	3.3	3.4	0	0.3	1.8	7.3	0.9	0.9	9.9	0	7.2	1.8	4.4	18.6
	P-CBT vs I-CBT+P		1.9	0.8	3	5	10.7	8.8	2.8	6	0	2.5	2.7	0	0.4	1.4	7.2	1.5	1.5	13.7	0	3.8	1.4	7.2	17.7
	P-CBT vs Int-CBT		1.7	0.6	2.7	3.8	8.8	6.7	3.2	3.5	0	2.5	2.6	0	0.2	1.4	5.6	0.7	0.7	7.6	23.1	5.6	1.4	3.4	14.3
	P-CBT vs NT		5.6	0.7	1.4	2.3	10.1	2.3	1.1	1.2	0	13	4.6	26.8	1.1	2.4	2	0.2	0.2	2.6	0	1.9	2.4	1.1	16.6
	P-CBT vs PBO		1.9	0.8	2.7	8	13.4	6.8	2.6	4.2	0	2.4	2.2	0	0.9	1.2	13.4	1.1	1.1	2.4	0	6.3	1.2	5.4	21.8
	P-CBT vs TAU		4.2	0.7	2.1	3.2	10.2	4.5	2.2	2.4	0	9	1.5	0	0.6	15.2	3.9	0.5	0.5	5.1	0	3.7	11.6	2.3	16.7
	G-BT vs G-CBT+P		4.5	1.9	0.1	0.9	1.6	5.2	2.4	2.9	33.4	18.8	5.7	0	1.4	3	5.8	0.2	0.2	4.1	0	2.2	3	1.1	1.6

© 2018 Zhou X et al. JAMA Psychiatry.

	G-BT vs I-BT+P		3.4	0	0.9	2.5	0	4	1.1	2.9	22.8	9.2	5.8	0	1.4	3.1	5.2	13.5	9.2	9.5	0	1.3	3.1	1.1	0
	G-BT vs I-CBT		4.1	0.1	1.9	2.3	0	5.9	2.8	3.1	29.2	10.9	8.3	0	1.5	4.4	4.9	0.6	0.6	6.8	0	5	4.4	3.1	0
	G-BT vs I-CBT+P		3.7	0	1.4	2.3	0	5.7	1.4	4.3	26	10.3	6.9	0	1.4	3.6	4.6	1.2	1.2	15.1	0	1.7	3.6	5.5	0
	G-BT vs Int-CBT		3.2	0	1.5	1.7	0	4.6	2.2	2.4	22.6	8.5	6.4	0	1.2	3.4	3.8	0.5	0.5	5.3	22.6	3.9	3.4	2.4	0
	G-BT vs NT		0	0	0	0	0	0	0	0	50	0	0	50	0	0	0	0	0	0	0	0	0	0	0
	G-BT vs PBO		4.5	0.1	0.7	4	0.1	3	0.9	2.1	28.5	11.9	6.7	0	1.9	3.5	8.7	1.2	1.2	4.7	0	7.1	3.5	5.6	0.1
	G-BT vs TAU		2	0	0.9	1.1	0	2.9	1.4	1.5	35	5.3	4	0	0.7	23	2.4	0.3	0.3	3.3	0	2.4	12	1.5	0
	G-CBT vs I-BT+P		4.4	0	1.2	3.2	0	5.2	1.4	3.8	0	11.9	7.5	0	1.8	4	6.7	17.5	12	12.3	0	1.7	4	1.5	0
	G-CBT vs I-CBT+P		5	0.1	1.8	3.1	0	7.8	2	5.8	0	13.9	9.3	0	1.9	4.9	6.2	1.6	1.6	20.4	0	2.3	4.9	7.4	0
	G-CBT vs Int-CBT		4.1	0.1	1.9	2.3	0	5.9	2.8	3.1	0	10.9	8.3	0	1.5	4.4	4.9	0.6	0.6	6.8	29.2	5	4.4	3.1	0
	G-CBT+P vs I-BT+P		0.4	1.9	1.3	2.6	1.6	10.8	3.8	6.9	0	5.2	2.7	0	0.6	1.4	13	19	13.4	9.5	0	0.2	1.4	2.6	1.6
	G-CBT+P vs I-CBT		0.2	2.3	2.5	2	1.9	14	6.5	7.5	0	7.3	4.4	0	0.4	2.3	13.4	1.1	1.1	14	0	9.3	2.3	5.3	1.9
	G-CBT+P vs I-CBT+P		0.3	2.1	1.9	2.3	1.8	14.1	4.7	9.4	0	6.2	3.6	0	0.5	1.9	13.1	1.9	1.9	17	0	4.9	1.9	9	1.8
	G-CBT+P vs Int-CBT		0.1	1.7	1.8	1.4	1.4	10.1	4.7	5.4	0	5.3	3.2	0	0.3	1.7	9.6	0.8	0.8	10	28.1	6.7	1.7	3.8	1.4
	G-CBT+P vs NT		4.5	1.9	0.1	0.9	1.6	5.2	2.4	2.9	0	18.8	5.7	33.4	1.4	3	5.8	0.2	0.2	4.1	0	2.2	3	1.1	1.6
	G-CBT+P vs TAU		2.7	2	0.8	0.1	1.7	8.1	3.8	4.4	0	14.1	1.9	0	0.7	19.4	8.3	0.5	0.5	7.4	0	4.6	14.7	2.5	1.7
	I-BT+P vs I-CBT		0.4	0.1	1.1	1.4	0.1	1.2	2.2	1	0	1.3	1.3	0	0.4	0.7	2.6	27.3	17	28.7	0	10.2	0.7	2.4	0.1
	I-BT+P vs Int-CBT		0.3	0	0.7	0.9	0	0.8	1.5	0.7	0	0.9	0.9	0	0.2	0.5	1.8	18.9	11.8	19.9	30.7	7.1	0.5	1.7	0
	I-BT+P vs NT		3.4	0	0.9	2.5	0	4	1.1	2.9	0	9.2	5.8	22.8	1.4	3.1	5.2	13.5	9.2	9.5	0	1.3	3.1	1.1	0
	I-BT+P vs TAU		2.6	0	0.4	2.1	0	2.6	0.2	2.4	0	7	3.9	0	1.1	14.5	4.4	16.7	11	14.1	0	3.6	13.2	0.2	0
	I-CBT vs NT		4.1	0.1	1.9	2.3	0	5.9	2.8	3.1	0	10.9	8.3	29.2	1.5	4.4	4.9	0.6	0.6	6.8	0	5	4.4	3.1	0
	I-CBT+P vs Int-CBT		0.2	0.1	0.6	0.4	0.1	0.8	1.8	2.6	0	0.9	0.8	0	0.1	0.4	0.4	1	1	35.5	43.6	4.6	0.4	4.6	0.1
	I-CBT+P vs NT		3.7	0	1.4	2.3	0	5.7	1.4	4.3	0	10.3	6.9	26	1.4	3.6	4.6	1.2	1.1	15.1	0	1.7	3.6	5.5	0
	I-CBT+P vs TAU		3	0.1	0.9	1.9	0.1	4.8	0.6	4.2	0	8.4	5.2	0	1.2	17.8	3.8	1.2	1.2	23.2	0	0.1	16.6	5.8	0.1
	Int-CBT vs NT		3.2	0	1.5	1.7	0	4.6	2.2	2.4	0	8.5	6.4	22.6	1.2	3.4	3.8	0.5	0.5	5.3	22.6	3.9	3.4	2.4	0
	Int-CBT vs PBO		0.6	0.1	1.4	2.2	0	3.4	2.2	1.2	0	1.5	1.7	0	0.6	0.9	4.8	2.2	2.2	13.9	34.9	14.7	0.9	10.5	0
	Int-CBT vs TAU		2.5	0	1.2	1.4	0	3.7	1.8	1.9	0	6.8	5.2	0	1	15.5	3.1	0.4	0.4	4.3	30.6	3.1	15.1	1.9	0
	NT vs PBO		4.5	0.1	0.7	4	0.1	3	0.9	2.1	0	11.9	6.7	28.5	1.9	3.5	8.7	1.2	1.2	4.7	0	7.1	3.5	5.6	0.1
	NT vs TAU		2	0	0.9	1.1	0	2.9	1.4	1.5	0	5.3	4	35	0.7	23	2.4	0.3	0.3	3.3	0	2.4	12	1.5	0
	PBO vs TAU		3.5	0.1	0.1	3.8	0.1	0.8	0.2	1	0	9.3	4.2	0	1.6	18.6	8.3	1.8	1.8	9.1	0	11.1	16.3	8.3	0.1
**Total**			3.5	1.4	2	3.7	3.1	7.1	2.8	4.1	5.4	7.8	4.3	5.4	0.9	5	5.5	3.9	2.9	9	5.4	4.3	4.3	3.8	4.3

© 2018 Zhou X et al. JAMA Psychiatry.

All-cause discontinuation

	Comparison	N of trials	WL vs BIB-CBT	WL vs P-CBT	WL vs G-BT	WL vs G-CBT	WL vs G-CBT+P	WL vs I+G-BT	WL vs I+G-CBT	WL vs I-CBT	WL vs I-CBT+P	WL vs Int-CBT	BIB-CBT vs G-CBT+P	BIB-CBT vs I-CBT	BIB-CBT vs I-CBT+P	P-CBT vs G-CBT+P	G-CBT vs G-CBT+P	G-CBT vs I-CBT	G-CBT vs NT	G-CBT vs PBO	G-CBT vs TAU	G-CBT+P vs I-CBT+P	G-CBT+P vs Int-CBT	G-CBT+P vs PBO	I+G-BT vs PBO	I-BT+P vs I-CBT	I-BT+P vs I-CBT+P	I-BT+P vs PBO	I-CBT vs I-CBT+P	I-CBT vs Int-CBT	I-CBT vs PBO	I-CBT vs TAU	I-CBT+P vs Int-CBT	I-CBT+P vs PBO	I-CBT+P vs TAU	Int-CBT vs PBO	Int-CBT vs TAU
Direct comparison	WL vs BIB-CBT	3	39.7	2.6	0	2.7	10.6	0.3	0	2.4	3.8	1.6	18.2	4.1	1.7	2.6	0.9	0.5	0	1.4	0	2.4	0.6	1.1	0.3	0	0	0.1	0.5	0.1	0.4	0.1	0.5	0.2	0.2	0.1	0.3
	WL vs P-CBT	4	4.5	32.9	0	2.3	12.6	0.2	0	1.4	2.9	1.5	4.6	0.1	0	25.4	1.3	0	0	1	0.1	4.3	0.8	1.8	0.2	0	0	0	0.9	0.1	0.4	0	0.3	0	0.1	0.1	0.2
	WL vs G-BT	1	0	0	99.8	0	0	0	0	0	0	0	0	0	0	0	0	0	0	0	0	0	0	0	0	0	0	0	0	0	0	0	0	0	0	0	0
	WL vs G-CBT	7	3	1.5	0	21.3	6	0.9	0	4.5	6.2	3.2	1.6	1.1	0.3	1.5	4.1	4.7	0	14.3	2.3	2.1	0.1	3.1	0.9	0.1	1.1	1.1	2.7	0.2	3.6	0.2	0.7	5	0.5	0.5	1.7
	WL vs G-CBT+P	13	9.4	6.4	0	4.8	26.3	0.5	0	2.9	6	3	9.6	0.2	0	6.4	2.7	0.1	0	2.1	0.1	8.9	1.6	3.7	0.5	0.1	0	0	1.9	0.1	0.9	0.1	0.7	0	0.2	0.2	0.4
	WL vs I+G-BT	2	2.4	1.1	0	7.3	4.5	8.2	0	3.6	5.4	2.3	1.2	0.9	0.3	1.1	1	0.4	0	9.2	0.5	2.2	0.1	3.7	26.5	0.1	1.3	1.4	1.3	0.2	5.5	0.1	0.7	6.2	0.1	0.6	0.7
	WL vs I+G-CBT	1	0	0	0	0	0	0	99.8	0	0	0	0	0	0	0	0	0	0	0	0	0	0	0	0	0	0	0	0	0	0	0	0	0	0	0	0
	WL vs I-CBT	7	4.5	1.5	0	7.6	6.1	0.8	0	11.5	10	3.7	0.8	3.1	0.5	1.5	0.9	4.2	0	4.2	0.1	5.3	0.1	2.3	0.8	0.5	0.3	0.2	16.9	0.8	7.7	0.9	1.4	0.2	0.3	0.4	1.1
	WL vs I-CBT+P	8	4.4	1.9	0	6.5	8	0.7	0	6.2	16.6	4.4	1.9	1.4	1.1	1.9	0.3	1.5	0	5.1	0.3	10	0.2	1.7	0.7	0.1	1.4	1.2	10.3	0.3	0.8	0.2	2.6	5.7	1.1	0.3	1
	WL vs Int-CBT	8	2.2	1.1	0	3.8	4.5	0.4	0	2.6	4.9	46.8	1.3	0.6	0.3	1.1	0.2	0.2	0	1.2	2.7	1.8	4.2	0.6	0.4	0	0.1	0.1	0.8	1.6	0.1	1	6.8	0.3	1.5	1.8	5.1
	BIB-CBT vs G-CBT+P	1	25.2	3.7	0	2	15	0.2	0	0.6	2.3	1.4	24.3	3.3	1.4	3.7	1.7	0.3	0	0.8	0.1	6.1	1	2.5	0.2	0.1	0	0.1	2.2	0	1.2	0	0.2	0.2	0.1	0.1	0.1
	BIB-CBT vs I-CBT	1	19.6	0.3	0	4.6	1.2	0.5	0	8	5.9	2.1	11.3	4.9	1.4	0.3	1.2	3.2	0	2.6	0.1	5.7	0.3	2.5	0.5	0.4	0.3	0.1	13.5	0.5	6	0.7	0.8	0.3	0.1	0.2	0.7
	BIB-CBT vs I-CBT+P	1	21.5	0	0	3.6	0.1	0.4	0	3.6	11.3	2.6	13.1	3.8	2	0	0.9	0.9	0	3.3	0.3	9.8	0.3	2.1	0.4	0.1	1.1	1	8.9	0.2	0.4	0.1	1.8	4.6	0.8	0.2	0.6
	P-CBT vs G-CBT+P	2	4.6	26	0	2.4	13	0.2	0	1.4	3	1.5	4.7	0.1	0	31	1.3	0	0	1	0.1	4.4	0.8	1.8	0.2	0	0	0	0.9	0.1	0.4	0	0.3	0	0.1	0.1	0.2
	G-CBT vs G-CBT+P	4	3.6	3	0	14.7	12.3	0.5	0	1.9	1.2	0.7	5	1	0.3	3	5.2	3.9	0	13.4	1.9	7.6	1	5.1	0.5	0	0.9	0.9	3.5	0.1	2.4	0.2	0.1	4.2	0.5	0.3	1.1
	G-CBT vs I-CBT	3	1.5	0.1	0	12.9	0.2	0.1	0	6.9	3.8	0.5	0.8	2	0.2	0.1	3	8.5	0	17.7	2.3	3.2	0	0.7	0.1	0.5	0.7	1.3	13.9	0.5	10.9	1.1	0.7	4.6	0.7	0.1	0.5
	G-CBT vs NT	3	0	0	0	0	0	0	0	0	0	0	0	0	0	0	0	0	99.9	0	0	0	0	0	0	0	0	0	0	0	0	0	0	0	0	0	0
	G-CBT vs PBO	6	1.2	0.5	0	11.3	2.1	0.9	0	2	3.8	0.8	0.5	0.5	0.2	0.5	2.9	5.1	0	37.5	1.9	2.1	0.1	4	0.9	0.2	1.4	1.6	0.7	0.1	7.1	0.4	0.6	6.9	0.8	0.6	0.7
	G-CBT vs TAU	1	0.1	0.2	0	10.4	0.6	0.3	0	0.2	1.3	10.1	0.3	0.1	0.1	0.2	2.3	3.7	0	10.5	15.6	1.4	1	1.2	0.3	0.1	0.9	1	1.8	0.4	3.2	5.1	1.4	4.1	8.2	0.6	13.5
	G-CBT+P vs I-CBT+P	3	3.1	3.1	0	2.4	12.8	0.3	0	3.6	10.9	1.8	5.6	1.5	1	3.1	2.4	1.3	0	3.1	0.4	16.2	1.1	4.5	0.3	0.1	1.3	1.2	8.2	0.2	1.5	0.2	1.9	5.4	0.9	0.1	0.6
	G-CBT+P vs Int-CBT	1	5.3	3.9	0	1	16	0.1	0	0.4	1.2	27.8	6	0.5	0.2	3.9	2.1	0.1	0	0.8	1.8	7.5	3.8	3.1	0.1	0	0	0.1	0.8	1.1	0.6	0.7	4.9	0.2	1.1	1.3	3.6
	G-CBT+P vs PBO	1	2.8	2.6	0	7	10.7	1.1	0	3.1	3.6	1.2	4.5	1.3	0.4	2.6	3.2	0.6	0	11.4	0.6	8.8	0.9	7.5	1.1	0.2	1.8	2	3	0.2	7	0.1	0.5	8.6	0	0.7	0.7
	I+G-BT vs PBO	1	1.2	0.6	0	3.7	2.3	13.4	0	1.8	2.7	1.1	0.6	0.4	0.2	0.6	0.5	0.2	0	4.6	0.3	1.1	0.1	1.9	53.6	0.1	0.7	0.7	0.6	0.1	2.8	0.1	0.4	3.1	0	0.3	0.3
	I-BT+P vs I-CBT	1	0.4	0.3	0	1	1.2	0.3	0	4.9	2.3	0.2	0.9	1.5	0.2	0.3	0.1	3.3	0	4.6	0.2	1.3	0.1	1.2	0.3	3.5	21.9	17.7	18.7	0.4	10	0.6	0.4	1.4	0.3	0.2	0.1
	I-BT+P vs I-CBT+P	1	0.1	0	0	3.2	0.1	0.5	0	0.5	3.8	0.2	0	0.2	0.3	0	0.7	0.8	0	5.3	0.6	3.2	0	2.4	0.5	3.8	29.7	21.3	8.2	0	4.5	0	0.7	8.4	0.5	0.3	0.1
	I-BT+P vs PBO	1	0.2	0	0	3.7	0.1	0.6	0	0.3	3.7	0.2	0.2	0.1	0.3	0	0.8	1.5	0	6.7	0.7	3.3	0	2.8	0.6	3.4	23.5	23.5	5.5	0	6.8	0.1	0.7	9.5	0.5	0.4	0.1
	I-CBT vs I-CBT+P	10	0.5	0.5	0	2.1	1.9	0.1	0	7.7	7.6	0.5	1.4	2.4	0.6	0.5	0.8	3.9	0	0.7	0.3	5.5	0.1	1	0.1	0.8	2.2	1.3	36.3	0.6	9.4	1	1.4	7.6	1	0.1	0.2
	I-CBT vs Int-CBT	1	2.4	0.6	0	4	2.4	0.4	0	7.8	5.3	23	0.1	2.2	0.3	0.6	0.6	3.3	0	2.7	1.4	3.2	2.4	1.5	0.4	0.4	0.3	0.1	14.1	1.5	6.2	1.3	4.9	0.3	1.1	1.3	3.8
	I-CBT vs PBO	6	0.6	0.4	0	4.9	1.6	1	0	6.1	1.1	0.1	1.3	1.9	0.1	0.4	0.9	5.4	0	12.2	1	1.8	0.1	4.2	1	0.8	2.1	2.8	16.5	0.5	18.6	0.8	0.2	11.1	0.1	0.7	0
	I-CBT vs TAU	1	1.4	0.2	0	1.8	0.8	0.1	0	5.8	2.2	8.7	0.4	1.7	0.1	0.2	0.5	4.1	0	5.9	12.3	1.4	0.9	0.5	0.1	0.4	0.2	0.2	13.7	0.8	6.6	5.6	1.9	0.2	8.1	0.5	12.7
	I-CBT+P vs Int-CBT	1	2.3	0.9	0	3	3.8	0.4	0	3.5	10.6	24.6	0.8	0.8	0.7	0.9	0.1	1.1	0	3.4	1.4	7.1	2.7	1	0.4	0.1	1.1	1	8	1.2	0.7	0.7	6.2	4.5	1.8	1.3	3.9
	I-CBT+P vs PBO	2	0.3	0	0	6.8	0	1.1	0	0.1	7.4	0.4	0.2	0.1	0.6	0	1.6	2.2	0	11.8	1.3	6.4	0	5.1	1.1	0.1	3.9	4	13.3	0	11.2	0	1.4	17.6	1	0.8	0.2
	I-CBT+P vs TAU	2	1.1	0.5	0	3.1	1.9	0.1	0	1	7	8.5	0.4	0.2	0.5	0.5	1	1.7	0	6.4	12.3	4.9	1	0.1	0.1	0.1	1.2	1.1	9	0.4	0.7	5	2.8	5	8.9	0.4	13.1
	Int-CBT vs PBO	1	1.8	0.8	0	6.9	3.3	1	0	3	4.1	21	0.8	0.8	0.2	0.8	1.1	0.6	0	10.6	2	1.8	2.3	4.2	1	0.2	1.6	1.8	2	1	6.7	0.6	4.3	7.7	0.9	1.6	3.4
	Int-CBT vs TAU	1	1.3	0.5	0	6.8	2	0.4	0	2.7	3.9	17.6	0.4	0.7	0.2	0.5	1.3	0.8	0	3.4	12.3	2.2	1.8	1.2	0.4	0	0.2	0.1	1.3	0.9	0	4.8	3.7	0.6	8	1	19
Indirect comparison	WL vs I-BT+P		3.1	1.4	0	6.6	5.6	0.8	0	4.7	9.4	3	1.4	1.1	0.6	1.4	0.7	0.5	0	6.9	0.2	5.1	0.1	2.7	0.8	2.5	17.8	14.4	2.2	0.3	2.2	0.2	1.4	1.2	0.5	0.4	0.8
	WL vs NT		2.1	1	0	14.5	4.1	0.6	0	3.1	4.2	2.2	1.1	0.7	0.2	1	2.8	3.2	31.8	9.7	1.6	1.4	0.1	2.1	0.6	0	0.7	0.8	1.9	0.2	2.5	0.1	0.5	3.4	0.3	0.3	1.1
	WL vs PBO		3.5	1.6	0	10.8	6.7	1.5	0	5.3	8.1	3.4	1.8	1.3	0.4	1.6	1.5	0.6	0	13.6	0.7	3.3	0.2	5.5	1.5	0.2	1.9	2.1	1.9	0.3	8.2	0.2	1.1	9.2	0.1	0.9	1
	WL vs TAU		2.6	1.2	0	8.8	4.7	0.6	0	4.2	6.8	12.7	1.2	1	0.4	1.2	1.4	0.6	0	2.5	13.3	3.3	0.9	1.6	0.6	0	0.1	0.1	0.7	0.2	0.1	5.1	0.8	0.4	8.5	0.2	14.7
	BIB-CBT vs P-CBT		24.8	23.8	0	0.2	2.3	0	0	0.6	0.4	0	16.6	2.8	1.2	21.6	0.4	0.3	0	0.2	0	1.6	0.2	0.6	0	0	0	0	1.1	0	0.6	0.1	0.1	0.1	0	0	0.1
	BIB-CBT vs G-BT		24.3	1.6	38.9	1.7	6.5	0.2	0	1.5	2.3	1	11.1	2.5	1	1.6	0.6	0.3	0	0.8	0	1.5	0.4	0.7	0.2	0	0	0	0.3	0.1	0.3	0.1	0.3	0.1	0.1	0.1	0.2
	BIB-CBT vs G-CBT		19.9	0.3	0	14.8	1.3	0.6	0	2.1	2.6	1.6	11.5	3.1	1.2	0.3	3.6	3.8	0	11.7	1.8	2.9	0.2	3	0.6	0.1	0.8	0.9	1.8	0.1	3	0.2	0.3	3.9	0.4	0.3	1.1
	BIB-CBT vs I+G-BT		16	0.3	0	4.8	1	6.7	0	1.9	2.8	1.1	9.2	2.6	1	0.3	1.3	0.5	0	7	0.4	2.9	0.2	3.5	22.1	0.1	1.1	1.2	0.8	0.1	4.7	0	0.4	5.2	0	0.4	0.4
	BIB-CBT vs I+G-CBT		24.3	1.6	0	1.7	6.5	0.2	38.9	1.5	2.3	1	11.1	2.5	1	1.6	0.6	0.3	0	0.8	0	1.5	0.4	0.7	0.2	0	0	0	0.3	0.1	0.3	0.1	0.3	0.1	0.1	0.1	0.2
	BIB-CBT vs I-BT+P		16	0	0	4.4	0.1	0.6	0	3	6.3	1.8	9.7	2.9	1.3	0	1	0.2	0	5.3	0.1	5.5	0.2	2.8	0.6	2.2	15.4	12.4	2.1	0.2	2.1	0.1	1	1.2	0.3	0.3	0.5
	BIB-CBT vs Int-CBT		24.5	1	0	0.6	4.1	0	0	0.1	0.6	28.2	12.7	3	1.3	1	0.7	0.2	0	0.2	1.7	2.7	3	1.1	0	0	0	0	0.9	1.1	0.3	0.7	4.6	0.1	1	1.2	3.4
	BIB-CBT vs NT		14.7	0.2	0	10.9	1	0.4	0	1.6	1.9	1.2	8.4	2.3	0.9	0.2	2.7	2.8	26.3	8.6	1.3	2.2	0.2	2.2	0.4	0	0.6	0.7	1.3	0.1	2.2	0.1	0.2	2.9	0.3	0.2	0.8

© 2018 Zhou X et al. JAMA Psychiatry.

BIB-CBT vs PBO	18.6	0	0	7.6	0.1	1.1	0	3.2	4.7	2	11.4	3.3	1.3	0	1.8	0.7	0	10.8	0.6	4.1	0.2	5.2	1.1	0.2	1.7	1.8	1.3	0.2	7.1	0.1	0.6	7.9	0	0.7	0.7
BIB-CBT vs TAU	17.9	0.4	0	5.4	1.7	0.3	0	2	3.3	8.9	10	2.8	1.2	0.4	1.5	0.7	0	2.6	10.2	3.7	1	1.7	0.3	0	0.1	0.1	0.3	0.2	0.3	4	0.8	0.4	6.6	0.2	11.1
P-CBT vs G-BT	2.8	20.8	36.8	1.4	8	0.1	0	0.9	1.8	0.9	2.9	0.1	0	16	0.8	0	0	0.6	0	2.7	0.5	1.1	0.1	0	0	0	0.6	0	0.3	0	0.2	0	0.1	0.1	0.1
P-CBT vs G-CBT	0.4	17.7	0	13.9	2.9	0.5	0	2.4	2.7	1.5	1.5	0.8	0.2	15.5	3.6	3.3	0	10.8	1.6	3.9	0.4	3.2	0.5	0	0.8	0.8	2.5	0.1	2.3	0.1	0.3	3.5	0.4	0.3	1.1
P-CBT vs I+G-BT	0.3	14.4	0	4.6	2.3	6.3	0	2.1	2.9	1.1	1.2	0.7	0.2	12.6	1.4	0.3	0	6.7	0.4	3.7	0.3	3.7	20.8	0.1	1	1.1	1.4	0.1	4.1	0.1	0.4	4.8	0	0.4	0.4
P-CBT vs I+G-CBT	2.8	20.8	0	1.4	8	0.1	36.8	0.9	1.8	0.9	2.9	0.1	0	16	0.8	0	0	0.6	0	2.7	0.5	1.1	0.1	0	0	0	0.6	0	0.3	0	0.2	0	0.1	0.1	0.1
P-CBT vs I-BT+P	0.3	14.8	0	4.3	1.5	0.6	0	3.2	6.2	1.7	1.1	1	0.5	13.4	1.2	0.4	0	5.1	0.1	6.2	0.3	3.1	0.6	2	14.5	11.7	1.3	0.2	1.6	0.1	1	1	0.3	0.3	0.5
P-CBT vs I-CBT	0.7	17.6	0	4.2	2.7	0.4	0	7.5	5.6	1.9	2	2.3	0.4	15.5	1.4	3	0	2.5	0.1	6.2	0.4	2.7	0.4	0.4	0.2	0.1	12.8	0.5	5.8	0.7	0.8	0.1	0.1	0.2	0.7
P-CBT vs I-CBT+P	0.5	19.2	0	3.4	1.9	0.4	0	3.8	10.6	2.4	1.4	1.2	0.8	17.4	1.1	1.1	0	3.2	0.3	10.2	0.4	2.4	0.4	0.1	1	0.9	7.2	0.2	0.9	0.1	1.7	4.3	0.8	0.2	0.6
P-CBT vs Int-CBT	1.7	21.1	0	0.7	5.7	0.1	0	0.6	1	26.2	2.3	0.4	0.2	17.4	1	0.1	0	0	1.6	3.9	3	1.6	0.1	0	0	0	0.1	0.9	0.3	0.6	4.2	0.2	0.9	1.1	3.1
P-CBT vs NT	0.3	13.3	0	10.4	2.2	0.4	0	1.8	2.1	1.1	1.1	0.6	0.2	11.7	2.7	2.5	25	8.1	1.2	2.9	0.3	2.4	0.4	0	0.6	0.6	1.9	0.1	1.8	0.1	0.2	2.7	0.3	0.2	0.8
P-CBT vs PBO	0.3	16.8	0	7.2	1.7	1	0	3.4	4.7	1.8	1.1	1.1	0.3	15.2	1.9	0.4	0	10.1	0.5	4.9	0.3	5.3	1	0.1	1.5	1.7	2	0.2	6.1	0.1	0.6	7.2	0	0.6	0.7
P-CBT vs TAU	0.4	16	0	5.2	3.1	0.3	0	2.3	3.4	8.4	1.5	0.8	0.3	13.8	1.6	0.4	0	2.3	9.5	4.5	1	2	0.3	0	0.1	0.1	1	0.1	0.2	3.7	0.7	0.3	6.1	0.2	10.4
G-BT vs G-CBT	2.1	1	31.8	14.5	4.1	0.6	0	3.1	4.2	2.2	1.1	0.7	0.2	1	2.8	3.2	0	9.7	1.6	1.4	0.1	2.1	0.6	0	0.7	0.8	1.9	0.2	2.5	0.1	0.5	3.4	0.3	0.3	1.1
G-BT vs G-CBT+P	5.9	4	37.2	3	16.5	0.3	0	1.8	3.8	1.9	6	0.1	0	4	1.7	0	0	1.3	0.1	5.6	1	2.4	0.3	0	0	0	1.2	0.1	0.6	0	0.4	0	0.1	0.1	0.2
G-BT vs I+G-BT	1.7	0.8	25.8	5.4	3.4	6.1	0	2.7	4	1.7	0.9	0.6	0.2	0.8	0.8	0.3	0	6.8	0.4	1.7	0.1	2.7	19.7	0.1	1	1.1	0.9	0.1	4.1	0.1	0.5	4.6	0.1	0.4	0.5
G-BT vs I+G-CBT	0	0	50	0	0	0	50	0	0	0	0	0	0	0	0	0	0	0	0	0	0	0	0	0	0	0	0	0	0	0	0	0	0	0	0
G-BT vs I-BT+P	2.3	1	25.7	4.9	4.2	0.6	0	3.5	7	2.2	1	0.9	0.4	1	0.5	0.4	0	5.1	0.1	3.8	0.1	2	0.6	1.8	13.2	10.7	1.6	0.2	1.6	0.1	1	0.9	0.3	0.3	0.6
G-BT vs I-CBT	3.1	1	31.3	5.2	4.2	0.5	0	7.9	6.8	2.6	0.6	2.2	0.4	1	0.6	2.9	0	2.9	0	3.6	0.1	1.6	0.5	0.3	0.2	0.1	11.6	0.5	5.3	0.6	1	0.1	0.2	0.3	0.8
G-BT vs I-CBT+P	3	1.3	32.8	4.4	5.4	0.5	0	4.2	11.1	3	1.3	1	0.7	1.3	0.2	1	0	3.4	0.2	6.7	0.1	1.1	0.5	0.1	0.9	0.8	6.9	0.2	0.6	0.1	1.8	3.9	0.7	0.2	0.7
G-BT vs Int-CBT	1.3	0.7	39.8	2.3	2.7	0.2	0	1.6	3	28.2	0.8	0.3	0.2	0.7	0.1	0.1	0	0.7	1.6	1.1	2.5	0.4	0.2	0	0.1	0	0.5	0.9	0	0.6	4.1	0.2	0.9	1.1	3.1
G-BT vs NT	1.6	0.8	24.1	11	3.1	0.5	0	2.3	3.2	1.7	0.8	0.6	0.2	0.8	2.1	2.4	24.1	7.4	1.2	1.1	0.1	1.6	0.5	0	0.6	0.6	1.4	0.1	1.9	0.1	0.4	2.6	0.2	0.3	0.9
G-BT vs PBO	2.5	1.2	29	7.7	4.8	1.1	0	3.8	5.7	2.4	1.3	0.9	0.3	1.2	1.1	0.4	0	9.7	0.5	2.4	0.1	3.9	1.1	0.1	1.4	1.5	1.3	0.2	5.8	0.1	0.8	6.5	0.1	0.6	0.7
G-BT vs TAU	1.8	0.8	29.4	6.2	3.3	0.4	0	2.9	4.8	9	0.8	0.7	0.3	0.8	1	0.4	0	1.8	9.4	2.3	0.6	1.1	0.4	0	0.1	0.1	0.5	0.1	0	3.6	0.5	0.3	6	0.1	10.4
G-CBT vs I+G-BT	0.1	0	0	10.5	0.1	9.2	0	0.3	1	0.2	0	0.1	0.1	0	2.4	3.8	0	24.2	1.5	0.8	0	1.7	33.2	0.1	0.6	0.7	0.9	0	3.4	0.3	0.2	3	0.5	0.3	0.7
G-CBT vs I+G-CBT	2.1	1	0	14.5	4.1	0.6	31.8	3.1	4.2	2.2	1.1	0.7	0.2	1	2.8	3.2	0	9.8	1.6	1.4	0.1	2.1	0.6	0	0.7	0.8	1.9	0.2	2.5	0.1	0.5	3.4	0.3	0.3	1.1
G-CBT vs I-BT+P	0.9	0.3	0	9.9	1.2	0.1	0	1.5	5.2	0.6	0.2	0.4	0.4	0.3	2.5	4.3	0	18.8	1.7	3.8	0	0.4	0.1	2.6	18.2	16.3	4.5	0.1	0.5	0.3	0.9	2.6	0.9	0.1	0.5
G-CBT vs I-CBT+P	1.1	0.4	0	13.7	1.5	0.2	0	1.3	8.8	0.9	0.2	0.3	0.6	0.4	3.4	5.5	0	17.4	2.4	6.8	0	1.3	0.2	0.1	2.2	2.1	11.5	0.1	4	0.3	1.6	9.5	1.4	0.2	0.7
G-CBT vs Int-CBT	1.2	0.5	0	14.7	2.2	0.6	0	2.1	2.1	23.5	0.6	0.5	0.1	0.5	3.1	3.8	0	12	3.3	0.6	2.4	2.1	0.6	0	0.9	0.9	2.6	1.1	2.8	0.4	4.3	4.1	0.5	1.4	4.2
G-CBT+P vs I+G-BT	2.8	2.4	0	4	9.7	7.1	0	1.7	1.7	0.5	4	0.9	0.3	2.4	2.3	0.4	0	7.1	0.4	6.6	0.8	5.3	24	0.1	1.2	1.3	2.1	0.1	4.4	0.1	0.3	5.5	0.1	0.4	0.4
G-CBT+P vs I+G-CBT	5.9	4	0	3	16.5	0.3	37.2	1.8	3.8	1.9	6	0.1	0	4	1.7	0	0	1.3	0.1	5.6	1	2.4	0.3	0	0	0	1.2	0.1	0.6	0	0.4	0	0.1	0.1	0.2
G-CBT+P vs I-BT+P	2.3	2.3	0	3.7	9.4	0.5	0	2.9	5.7	1.2	4.1	1.2	0.6	2.3	2.2	0.5	0	5.5	0.1	9.9	0.8	4.7	0.5	2.4	17.2	13.8	1	0.2	1.6	0.1	0.9	1.2	0.3	0.3	0.5
G-CBT+P vs I-CBT	2.4	3	0	3.4	12.3	0.4	0	8.1	4.6	1.2	5.7	2.9	0.5	3	2.6	3.6	0	2.2	0.1	10.6	1	4.5	0.4	0.5	0.3	0.2	16	0.6	7.3	0.8	0.7	0.1	0.1	0.2	0.7
G-CBT+P vs NT	2.6	2.2	0	10.6	8.9	0.3	0	1.3	0.8	0.5	3.6	0.8	0.2	2.2	3.7	2.8	28.1	9.6	1.3	5.5	0.7	3.6	0.3	0	0.7	0.7	2.5	0.1	1.8	0.1	0.1	3	0.4	0.2	0.8
G-CBT+P vs TAU	3.2	2.7	0	4.5	11.2	0.2	0	1.8	2.1	8.6	4.5	1	0.3	2.7	2.6	0.5	0	3.2	10.8	7.7	1.6	3.4	0.2	0	0.1	0	1.7	0.2	0.5	4.2	1	0.3	7	0.2	11.7
I+G-BT vs I+G-CBT	1.7	0.8	0	5.4	3.4	6.1	25.8	2.7	4	1.7	0.9	0.6	0.2	0.8	0.8	0.3	0	6.8	0.4	1.7	0.1	2.7	19.7	0.1	1	1.1	0.9	0.1	4.1	0.1	0.5	4.6	0.1	0.4	0.5
I+G-BT vs I-BT+P	0.7	0.3	0	0.6	1.1	7.4	0	1.2	4	0.7	0.2	0.3	0.3	0.3	0.3	0.9	0	2.2	0.3	2.9	0	1	27.4	2.4	16.6	15.9	3.4	0.1	3.3	0.1	0.7	5	0.4	0.1	0.1
I+G-BT vs I-CBT	1.1	0	0	1.5	0.1	7.9	0	5.4	2.3	0.6	0.6	1.6	0.1	0	0.4	3.7	0	6.2	0.6	1.9	0	2	28.3	0.5	1.1	1.6	12.2	0.4	11.8	0.6	0.4	6.3	0.1	0.3	0.2
I+G-BT vs I-CBT+P	0.8	0.3	0	2.7	1.2	8	0	0.9	6.6	0.9	0.2	0.2	0.5	0.3	0.8	1.5	0	5.8	0.7	5.1	0	2.6	28	0	2.4	2.4	8.9	0	6.3	0	1.2	10.6	0.7	0.4	0
I+G-BT vs Int-CBT	1	0.4	0	4.4	1.8	6.7	0	1.8	2.4	18.6	0.4	0.5	0.1	0.4	0.8	0.4	0	7.1	1.6	1.1	1.9	2.8	22.3	0.1	1.1	1.2	1.4	0.8	4.6	0.5	3.6	5.3	0.7	1.3	2.8
I+G-BT vs NT	0.1	0	0	7.4	0.1	6.4	0	0.2	0.7	0.1	0	0.1	0.1	0	1.7	2.7	29.8	17	1.1	0.6	0	1.2	23.3	0.1	0.4	0.5	0.7	0	2.4	0.2	0.2	2.1	0.4	0.2	0.5
I+G-BT vs TAU	0.2	0.1	0	0.1	0.5	6.9	0	0.1	0.2	7.5	0.2	0	0	0.1	0.1	0.1	0	10	10.3	0.4	0.8	2.1	24.1	0.1	1.1	1.2	0.6	0.3	4.9	3.9	1.2	5.2	6.4	0.7	10.4
I+G-CBT vs I-BT+P	2.3	1	0	4.9	4.2	0.6	25.7	3.5	7	2.2	1	0.9	0.4	1	0.5	0.4	0	5.1	0.1	3.8	0.1	2	0.6	1.8	13.2	10.7	1.6	0.2	1.6	0.1	1	0.9	0.3	0.3	0.6
I+G-CBT vs I-CBT	3.1	1	0	5.2	4.2	0.5	31.3	7.9	6.8	2.6	0.6	2.2	0.4	1	0.6	2.9	0	2.9	0	3.6	0.1	1.6	0.5	0.3	0.2	0.1	11.6	0.5	5.3	0.6	1	0.1	0.2	0.3	0.8
I+G-CBT vs I-CBT+P	3	1.3	0	4.3	5.4	0.5	32.7	4.2	11.1	3	1.3	1	0.7	1.3	0.2	1	0	3.4	0.2	6.7	0.1	1.1	0.5	0.1	0.9	0.8	6.9	0.2	0.6	0.1	1.8	3.9	0.7	0.2	0.7
I+G-CBT vs Int-CBT	1.3	0.7	0	2.3	2.7	0.2	39.8	1.6	3	28.2	0.8	0.3	0.2	0.7	0.1	0.1	0	0.7	1.6	1.1	2.5	0.4	0.2	0	0.1	0	0.5	0.9	0	0.6	4.1	0.2	0.9	1.1	3.1
I+G-CBT vs NT	1.6	0.8	0	11	3.1	0.5	24.1	2.3	3.2	1.7	0.8	0.6	0.2	0.8	2.1	2.4	24.1	7.4	1.2	1.1	0.1	1.6	0.5	0	0.6	0.6	1.4	0.1	1.9	0.1	0.4	2.6	0.2	0.3	0.9
I+G-CBT vs PBO	2.5	1.2	0	7.7	4.8	1.1	29	3.8	5.7	2.4	1.3	0.9	0.3	1.2	1.1	0.4	0	9.7	0.5	2.4	0.1	3.9	1.1	0.1	1.4	1.5	1.3	0.2	5.8	0.1	0.8	6.5	0.1	0.6	0.7
I+G-CBT vs TAU	1.8	0.8	0	6.2	3.3	0.4	29.3	2.9	4.8	9	0.8	0.7	0.3	0.8	1	0.4	0	1.8	9.4	2.3	0.6	1.1	0.4	0	0.1	0.1	0.5	0.1	0	3.6	0.5	0.3	6	0.1	10.4
I-BT+P vs Int-CBT	1.7	0.7	0	3.9	2.8	0.6	0	2.9	5.8	18.4	0.6	0.7	0.4	0.7	0.5	0.4	0	5.4	1.3	3.5	2	2	0.6	2.1	15.3	12.3	1.5	0.9	1.9	0.6	4.2	1.2	1.1	1.2	3
I-BT+P vs NT	0.7	0.2	0	7.2	0.9	0.1	0	1.1	3.8	0.5	0.1	0.3	0.3	0.2	1.8	3.1	27	13.7	1.2	2.8	0	0.3	0.1	1.9	13.3	11.9	3.3	0.1	0.4	0.2	0.7	1.9	0.6	0.1	0.4
I-BT+P vs TAU	0.8	0.4	0	0.7	1.5	0.3	0	1.1	3.4	6.8	0.4	0.3	0.2	0.4	0.4	0.9	0	8.1	10	2.1	0.8	1.2	0.3	2.2	15.9	12.9	2.5	0.3	1.9	4	1.8	0.8	6.7	0.5	10.3
I-CBT vs NT	1	0	0	8.9	0.2	0.1	0	4.8	2.7	0.4	0.5	1.4	0.2	0	2.1	5.9	30.7	12.2	1.6	2.2	0	0.5	0.1	0.4	0.5	0.9	9.6	0.4	7.6	0.7	0.5	3.2	0.5	0.1	0.4
I-CBT+P vs NT	0.8	0.3	0	9.6	1	0.2	0	0.9	6.2	0.6	0.1	0.2	0.4	0.3	2.4	3.9	29.8	12.2	1.7	4.8	0	0.9	0.1	0	1.5	1.5	8.1	0	2.8	0.2	1.1	6.7	1	0.1	0.5
Int-CBT vs NT	0.9	0.4	0	10.8	1.6	0.4	0	1.5	1.5	17.2	0.4	0.4	0.1	0.4	2.2	2.8	27	8.7	2.4	0.5	1.8	1.5	0.4	0	0.7	0.7	1.9	0.8	2.1	0.3	3.2	3	0.3	1	3.1
NT vs PBO	0.8	0.3	0	7.1	1.3	0.6	0	1.2	2.4	0.5	0.3	0.3	0.1	0.3	1.8	3.2	36.9	23.6	1.2	1.4	0	2.5	0.6	0.1	0.9	1	0.5	0.1	4.5	0.3	0.4	4.3	0.5	0.4	0.4
NT vs TAU	0.1	0.1	0	7.3	0.4	0.2	0	0.1	0.9	7.1	0.2	0	0.1	0.1	1.6	2.6	29.8	7.4	11	1	0.7	0.9	0.2	0	0.6	0.7	1.2	0.3	2.3	3.6	1	2.9	5.8	0.4	9.5

© 2018 Zhou X et al. JAMA Psychiatry.

	PBO vs TAU		0.9	0.5	0	1.9	1.9	0.9	0	1.1	1.3	8.4	0.6	0.3	0.1	0.5	0.2	0	0	14.8	12.7	0.1	1	3.6	0.9	0.2	1.7	1.9	1.1	0.4	7.5	4.8	1.7	8.1	7.8	1	12.4
	**Total**		4.3	3.4	4.8	6.1	4	1.5	4.8	2.8	4.3	5.3	2.8	1.1	0.4	3.1	1.5	1.7	4.8	6.9	2.3	3.7	0.6	2.3	4.2	0.4	3.1	2.7	3.9	0.3	3.2	0.8	1.2	3.2	1.4	0.4	2.5

Mean overall change in quality of life and functional improvement

	**Comparison**	N of trials	WL vs P-CBT	WL vs G-CBT	WL vs G-CBT+P	WL vs I+G-BT	WL vs I+G-CBT	WL vs I-CBT	WL vs I-CBT+P	WL vs Int-CBT	BIB-CBT vs I-CBT	G-CBT vs G-CBT+P	G-CBT vs I-CBT	G-CBT vs PBO	G-CBT+P vs I-CBT+P	I+G-BT vs PBO	I-CBT vs I-CBT+P	I-CBT vs Int-CBT	I-CBT vs PBO	I-CBT+P vs Int-CBT	I-CBT+P vs TAU	Int-CBT vs PBO	Int-CBT vs TAU
**Direct comparison**	WL vs P-CBT	1	100	0	0	0	0	0	0	0	0	0	0	0	0	0	0	0	0	0	0	0	0
	WL vs G-CBT	2	0	3.7	18.6	3.6	0	9.7	1.6	5.5	0	17	15.1	6.9	1.5	3.6	5.5	1.4	1.5	1.6	0.8	1.8	0.8
	WL vs G-CBT+P	3	0	0.9	54.1	1.7	0	6.1	1.6	4.5	0	3.9	1.9	1.1	10.9	1.7	5.1	0.3	0.6	2.8	1.4	0	1.4
	WL vs I+G-BT	1	0	0.5	5.4	35.7	0	6.3	1.1	4.6	0	2.1	0.1	2.7	3.3	18	3.6	0.3	10.3	0.5	0.2	5	0.2
	WL vs I+G-CBT	1	0	0	0	0	100	0	0	0	0	0	0	0	0	0	0	0	0	0	0	0	0
	WL vs I-CBT	2	0	0.9	12.3	3.9	0	18.8	2.8	8.9	0	3.1	3.6	0.4	9.2	3.9	16.7	3.5	5	3.1	1.6	0.7	1.6
	WL vs I-CBT+P	2	0	0.7	14.7	3	0	12.7	3.8	10.1	0	1.5	2	0.3	13.2	3	17.3	0.2	2.4	7.1	3.6	0.9	3.6
	WL vs Int-CBT	7	0	0.6	10.3	3.3	0	10.2	2.5	18.3	0	1.6	1.5	0.8	8.7	3.3	6.2	5.2	0.2	11.6	5.8	4.3	5.8
	BIB-CBT vs I-CBT	1	0	0	0	0	0	0	0	0	100	0	0	0	0	0	0	0	0	0	0	0	0
	G-CBT vs G-CBT+P	1	0	3.3	15.9	2.7	0	6.3	0.7	2.9	0	20.7	14.8	6.6	8.8	2.7	9.2	1.2	1.9	0.2	0.1	1.9	0.1
	G-CBT vs I-CBT	1	0	3.2	8.6	0.1	0	8.1	1	2.8	0	16.1	20.8	8.1	7.6	0.1	10.7	2	6.7	1.4	0.7	1.3	0.7
	G-CBT vs PBO	3	0	2.5	8.5	6.7	0	1.5	0.2	2.6	0	12.6	14.3	11.2	4.1	6.7	1	0.7	16.1	2.3	1.1	6.7	1.1
	G-CBT+P vs I-CBT+P	1	0	0.1	20.7	1.9	0	8.7	2.7	7.2	0	4.1	3.2	1	20.5	1.9	14	0	2	5.4	2.7	0.9	2.7
	I+G-BT vs PBO	1	0	0.6	6.1	20.1	0	7.1	1.2	5.2	0	2.4	0.1	3.1	3.7	28.1	4.1	0.4	11.5	0.5	0.3	5.6	0.3
	I-CBT vs I-CBT+P	2	0	0.2	4.6	1	0	7.4	1.7	2.4	0	2	2.1	0.1	6.6	1	48.3	4.5	3.4	6.1	3.1	2.3	3.1
	I-CBT vs Int-CBT	1	0	0.3	1.5	0.5	0	8.2	0.1	10.5	0	1.4	2.1	0.4	0.1	0.5	23.5	9.1	5.3	15.6	7.8	5.3	7.8
	I-CBT vs PBO	2	0	0.2	1.6	8.5	0	6.7	0.7	0.3	0	1.3	4.1	5.5	2.9	8.5	10.2	3.1	27.6	4.4	2.2	10	2.2
	I-CBT+P vs Int-CBT	1	0	0.1	5.4	0.3	0	3.1	1.5	9.9	0	0.1	0.6	0.6	5.5	0.3	13.4	6.6	3.2	22.6	11.4	4	11.4
	I-CBT+P vs TAU	3	0	0.1	3	0.2	0	1.7	0.8	5.5	0	0	0.3	0.3	3.1	0.2	7.5	3.7	1.8	12.6	32.8	2.3	24.1
	Int-CBT vs PBO	1	0	0.4	0	7.1	0	1.6	0.5	8.6	0	2.2	1.4	3.9	2.2	7.1	11.7	5.2	17.1	9.5	4.8	12.2	4.8
	Int-CBT vs TAU	1	0	0.1	2.8	0.1	0	1.6	0.8	5.1	0	0	0.3	0.3	2.8	0.1	6.9	3.4	1.6	11.7	22.2	2.1	38
**Indirect comparison**	WL vs BIB-CBT		0	0.6	8.3	2.7	0	12.7	1.9	6	32.3	2.1	2.5	0.3	6.2	2.7	11.3	2.4	3.4	2.1	1.1	0.5	1.1
	WL vs PBO		0	0.9	9.3	10.2	0	10.9	1.8	7.9	0	3.7	0.1	4.7	5.6	10.2	6.3	0.6	17.6	0.8	0.4	8.5	0.4
	WL vs TAU		0	0.5	9.6	2.4	0	8.8	2.4	11.3	0	1.2	1.3	0.4	8.4	2.4	8.9	2.1	0.8	2.2	17.4	2.1	17.6
	BIB-CBT vs P-CBT		24.4	0.5	6.3	2	0	9.6	1.4	4.6	24.4	1.6	1.9	0.2	4.7	2	8.5	1.8	2.6	1.6	0.8	0.4	0.8
	BIB-CBT vs G-CBT		0	2.2	5.8	0.1	0	5.4	0.6	1.9	32.5	10.9	14	5.5	5.1	0.1	7.2	1.4	4.5	1	0.5	0.9	0.5
	BIB-CBT vs G-CBT+P		0	0.2	15.7	1.7	0	9.1	1	3.6	29.7	3.6	3.1	0.7	10.4	1.7	12.6	2.1	2.9	0.8	0.4	0.4	0.4
	BIB-CBT vs I+G-BT		0	0.2	4.2	15.6	0	7.4	1	2.7	26.8	0.7	2.1	1.2	3.5	11.2	7.6	1.8	7.9	2	1	2.1	1
	BIB-CBT vs I+G-CBT		0	0.5	6.3	2	24.4	9.6	1.4	4.6	24.4	1.6	1.9	0.2	4.7	2	8.5	1.8	2.6	1.6	0.8	0.4	0.8
	BIB-CBT vs I-CBT+P		0	0.1	2.8	0.6	0	4.5	1	1.5	39.7	1.2	1.3	0.1	4	0.6	29.1	2.7	2	3.7	1.9	1.4	1.9
	BIB-CBT vs Int-CBT		0	0.2	1	0.3	0	5.5	0.1	7.1	32.6	0.9	1.4	0.3	0	0.3	15.9	6.2	3.6	10.5	5.3	3.5	5.3
	BIB-CBT vs PBO		0	0.1	1	5.6	0	4.4	0.5	0.2	34.1	0.8	2.7	3.6	1.9	5.6	6.7	2	18.2	2.9	1.5	6.6	1.5
	BIB-CBT vs TAU		0	0.1	0.5	0.4	0	4.1	0.3	3.9	28.8	0.9	1.1	0.1	1.3	0.4	17.4	3.8	2.4	3.7	15.3	2.1	13.5
	P-CBT vs G-CBT		29.9	2.6	13	2.5	0	6.8	1.1	3.9	0	11.9	10.6	4.8	1.1	2.5	3.9	1	1	1.1	0.5	1.3	0.5
	P-CBT vs G-CBT+P		40.8	0.5	32	1	0	3.6	0.9	2.7	0	2.3	1.1	0.6	6.5	1	3	0.2	0.4	1.6	0.8	0	0.8
	P-CBT vs I+G-BT		34.9	0.4	3.5	23.2	0	4.1	0.7	3	0	1.4	0	1.8	2.1	11.7	2.4	0.2	6.7	0.3	0.2	3.2	0.2
	P-CBT vs I+G-CBT		50	0	0	0	50	0	0	0	0	0	0	0	0	0	0	0	0	0	0	0	0
	P-CBT vs I-CBT		32.3	0.6	8.3	2.7	0	12.7	1.9	6	0	2.1	2.5	0.3	6.2	2.7	11.3	2.4	3.4	2.1	1.1	0.5	1.1
	P-CBT vs I-CBT+P		31	0.5	10.1	2.1	0	8.7	2.6	6.9	0	1.1	1.4	0.2	9.1	2.1	11.9	0.2	1.7	4.9	2.5	0.6	2.5
	P-CBT vs Int-CBT		31.1	0.4	7.1	2.2	0	7	1.7	12.6	0	1.1	1	0.5	6	2.2	4.3	3.6	0.2	8	4	2.9	4
	P-CBT vs PBO		29.1	0.7	6.6	7.2	0	7.7	1.3	5.6	0	2.6	0.1	3.3	4	7.2	4.4	0.4	12.5	0.6	0.3	6	0.3
	P-CBT vs TAU		26	0.4	7.1	1.8	0	6.5	1.8	8.3	0	0.9	1	0.3	6.2	1.8	6.6	1.5	0.6	1.6	12.9	1.6	13.1
	G-CBT vs I+G-BT		0	2.6	11.5	19.9	0	3.8	0.6	1.5	0	12.3	12.1	7.3	0.9	14.4	2.1	0.9	5.4	1.5	0.8	1.7	0.8

© 2018 Zhou X et al. JAMA Psychiatry.

	G-CBT vs I+G-CBT		0	2.6	13	2.5	29.9	6.8	1.1	3.9	0	11.9	10.6	4.8	1.1	2.5	3.9	1	1	1.1	0.5	1.3	0.5
	G-CBT vs I-CBT+P		0	2.8	4.3	0.7	0	2.1	1.8	3.8	0	14.4	15.7	6.6	10.2	0.7	20.3	1.1	3.5	4.9	2.4	2.5	2.4
	G-CBT vs Int-CBT		0	2.5	7.3	0.4	0	0.1	0.6	9.7	0	12.8	13.6	6.2	5.5	0.4	9.4	5.2	1	10.3	5.2	4.8	5.2
	G-CBT vs TAU		0	2.1	4.8	0.4	0	0.7	0.9	5.7	0	10.8	11.6	5.1	6	0.4	11.3	2.7	1.7	2.9	15.3	3	14.4
	G-CBT+P vs I+G-BT		0	0.1	25.5	23.9	0	1.1	0.1	0.8	0	3.6	1	2.5	8.2	13.4	5.3	0.1	7.4	1.8	0.9	3.4	0.9
	G-CBT+P vs I+G-CBT		0	0.5	32	1	40.8	3.6	0.9	2.7	0	2.3	1.1	0.6	6.5	1	3	0.2	0.4	1.6	0.8	0	0.8
	G-CBT+P vs I-CBT		0	0.3	22.3	2.4	0	12.9	1.5	5.2	0	5.1	4.4	1	14.8	2.4	17.9	2.9	4.1	1.1	0.5	0.6	0.5
	G-CBT+P vs Int-CBT		0	0	21.6	1.8	0	5.3	1.3	13.2	0	3.6	2.3	1.3	13.6	1.8	2.4	4.6	0.5	11.5	5.8	3.6	5.8
	G-CBT+P vs PBO		0	0.3	19	7.6	0	6	0.7	4.3	0	5	0.9	4.4	10.1	7.6	7.7	0.3	14.9	2	1	7.1	1
	G-CBT+P vs TAU		0	0.1	16.7	1.5	0	5.3	1.5	8.3	0	3	2.1	0.9	13	1.5	5.8	2.1	0.4	3.4	16.9	1.9	15.7
	I+G-BT vs I+G-CBT		0	0.4	3.5	23.2	34.9	4.1	0.7	3	0	1.4	0	1.8	2.1	11.7	2.4	0.2	6.7	0.3	0.2	3.2	0.2
	I+G-BT vs I-CBT		0	0.3	5.7	21.3	0	10.1	1.4	3.7	0	0.9	2.8	1.6	4.8	15.3	10.3	2.5	10.8	2.7	1.4	2.9	1.4
	I+G-BT vs I-CBT+P		0	0.2	7.4	19.5	0	5.3	2.1	4.5	0	0.2	1.5	1.5	7.5	13.1	14.7	0	8	5.5	2.7	3.7	2.7
	I+G-BT vs Int-CBT		0	0.1	4.2	19.8	0	3.6	1.2	10.7	0	0.1	1.1	1.1	4.4	13.6	6.9	4.1	6.2	8.3	4.2	6.2	4.2
	I+G-BT vs TAU		0	0.1	4.7	16.3	0	3.7	1.3	6.4	0	0.1	1.1	1.1	4.9	11.1	8.8	1.8	5.8	1.5	13.5	4.2	13.9
	I+G-CBT vs I-CBT		0	0.6	8.3	2.7	32.3	12.7	1.9	6	0	2.1	2.5	0.3	6.2	2.7	11.3	2.4	3.4	2.1	1.1	0.5	1.1
	I+G-CBT vs I-CBT+P		0	0.5	10.1	2.1	31	8.7	2.6	6.9	0	1.1	1.4	0.2	9.1	2.1	11.9	0.2	1.7	4.9	2.5	0.6	2.5
	I+G-CBT vs Int-CBT		0	0.4	7.1	2.2	31.1	7	1.7	12.6	0	1.1	1	0.5	6	2.2	4.3	3.6	0.2	8	4	2.9	4
	I+G-CBT vs PBO		0	0.7	6.6	7.2	29.1	7.7	1.3	5.6	0	2.6	0.1	3.3	4	7.2	4.4	0.4	12.5	0.6	0.3	6	0.3
	I+G-CBT vs TAU		0	0.4	7.1	1.8	26	6.5	1.8	8.3	0	0.9	1	0.3	6.2	1.8	6.6	1.5	0.6	1.6	12.9	1.6	13.1
	I-CBT vs TAU		0	0.2	0.7	0.5	0	5.8	0.4	5.4	0	1.2	1.6	0.2	1.9	0.5	24.4	5.4	3.3	5.2	21.4	3	19
	I-CBT+P vs PBO		0	0.3	4	7.1	0	0.7	1.5	1.2	0	2.2	1.8	4.3	6.1	7.1	21.2	0.4	19.1	7	3.5	9	3.5
	PBO vs TAU		0	0.3	1.5	5.7	0	0.4	0.8	4.1	0	1.7	1.3	3.3	3.3	5.7	13	2.4	14.5	1.4	15.6	8.6	16.4
**Total**			6.2	0.8	8.9	5.9	6.2	6.3	1.3	5.6	6.2	3.7	3.5	2.2	5.6	4.7	9.5	2	5.3	3.8	4.7	3	4.7

© 2018 Zhou X et al. JAMA Psychiatry.

Mean overall change in symptoms at short-term follow-up

	Comparison	N of trials	WL vs G-BT	WL vs G-CBT	WL vs G-CBT+P	WL vs I-CBT	WL vs PBO	WL vs P-CBT	BIB-CBT vs G-CBT+P	BIB-CBT vs I-CBT	BIB-CBT vs I-CBT+P	P-CBT vs G-CBT+P	G-BT vs G-CBT	G-CBT vs G-CBT+P	G-CBT vs I-CBT	G-CBT vs NT	G-CBT vs PBO	G-CBT vs TAU	G-CBT+P vs PBO	I-BT+P vs I-CBT+P	I-BT+P vs PBO	I-CBT vs I-CBT+P	I-CBT vs Int-CBT	I-CBT vs PBO	I-CBT vs TAU	I-CBT+P vs PBO
Direct comparison	WL vs G-BT	1	24	9.6	3.2	3.2	5.4	2.7	0.9	0.6	0.4	2.7	24.1	6.3	5.8	0	0.9	1.5	1.3	0.6	0.6	2.1	0	1.4	1.5	1.1
	WL vs G-CBT	3	8.2	15.5	5.1	5.2	8.6	4.4	1.5	0.9	0.6	4.4	8.2	10.1	9.4	0	1.4	2.4	2.1	1	1	3.4	0	2.3	2.4	1.7
	WL vs G-CBT+P	1	4.5	8.4	8.3	4.9	10.6	7.1	7.9	3.6	4.4	7.1	4.5	9.8	2.2	0	0.3	0.6	10.7	0.2	0.2	3.9	0	0.2	0.6	0.3
	WL vs I-CBT	1	4.3	8.1	4.7	6.9	9.5	4	7.7	3.9	3.8	4	4.3	0.3	9.6	0	0.1	2.5	1.3	2.2	2.2	9.5	0	5.1	2.5	3.6
	WL vs PBO	1	3.9	7.3	5.5	5.1	16.2	4.7	1.4	0.1	1.3	4.7	3.9	3.6	4.8	0	1.5	1.2	12.3	2.7	2.7	5.8	0	5.5	1.2	4.4
	WL vs P-CBT	1	2.8	5.3	5.3	3.1	6.7	22.1	5	2.3	2.8	23.3	2.8	6.2	1.4	0	0.2	0.4	6.8	0.1	0.1	2.5	0	0.2	0.4	0.2
	BIB-CBT vs G-CBT+P	1	0.2	0.5	1.5	1.5	0.5	1.3	47.3	6.2	8.3	1.3	0.2	4.8	4.4	0	0	1.1	7	1.8	1.8	3.5	0	2.7	1.1	3
	BIB-CBT vs I-CBT	1	0.4	0.7	1.8	2	0.1	1.5	16.5	12.5	14.6	1.5	0.4	6	5.8	0	0.1	1.5	7.2	1.1	1.1	17.5	0	4.3	1.5	1.8
	BIB-CBT vs I-CBT+P	2	0.2	0.4	1.5	1.4	0.9	1.3	15.3	10.1	18.6	1.3	0.2	4.7	4.1	0	0.1	1.1	7.8	2.6	2.6	18.7	0	2	1.1	4.2
	G-BT vs G-CBT	1	19.1	7.6	2.5	2.6	4.3	2.2	0.8	0.4	0.3	2.2	39.7	5	4.6	0	0.7	1.2	1.1	0.5	0.5	1.7	0	1.1	1.2	0.8
	G-CBT vs G-CBT+P	1	3.1	5.9	3.5	0.1	2.5	3	9.2	4.3	4.9	3	3.1	18.8	10.8	0	1.6	2.8	8.5	0.8	0.8	7	0	2.3	2.8	1.3
	G-CBT vs I-CBT	2	2.7	5.1	0.7	3.4	3.1	0.6	7.9	3.9	4	0.6	2.7	10.1	20.7	0	1.6	5.3	3.6	1.6	1.6	8.3	0	4	5.3	2.7
	G-CBT vs NT	1	0	0	0	0	0	0	0	0	0	0	0	0	0	99.9	0	0	0	0	0	0	0	0	0	0
	G-CBT vs PBO	2	3.4	6.3	0.8	0.4	7.8	0.7	0	0.7	0.7	0.7	3.4	12.1	12.6	0	2.6	3.2	13.4	3.4	3.4	8.3	0	7.2	3.2	5.6
	G-CBT vs TAU	1	1.3	2.5	0.4	1.7	1.5	0.3	3.9	1.9	2	0.3	1.3	5	10.2	0	0.8	36.1	1.8	0.8	0.8	4.1	0	2	19.9	1.3
	G-CBT+P vs PBO	1	0.5	1	3.1	0.4	6.9	2.6	10.8	4.2	6.6	2.6	0.5	6.9	3.1	0	1.4	0.8	26.8	3.4	3.4	2.3	0	6.3	0.8	5.5
	I-BT+P vs I-CBT+P	1	0.4	0.8	0.1	1.1	2.4	0.1	4.5	1	3.4	0.1	0.4	1	2.2	0	0.6	0.6	5.4	36.7	18.7	9.6	0	4.7	0.6	5.6
	I-BT+P vs PBO	1	0.5	1	0.1	1.3	3	0.1	5.5	1.3	4.2	0.1	0.5	1.3	2.8	0	0.7	0.7	6.6	23	22.2	11.8	0	5.8	0.7	6.9
	I-CBT vs I-CBT+P	9	0.3	0.7	0.5	1.2	1.3	0.4	2.2	4.2	6.4	0.4	0.3	2.3	2.9	0	0.4	0.8	0.9	2.5	2.5	61.2	0	3.9	0.8	4.1
	I-CBT vs Int-CBT	1	0	0	0	0	0	0	0	0	0	0	0	0	0	0	0	0	0	0	0	0	100	0	0	0
	I-CBT vs PBO	1	1	1.9	0.1	2.7	5.5	0.1	7.4	4.4	3	0.1	1	3.4	6.1	0	1.4	1.6	11	5.2	5.2	16.9	0	11.6	1.6	8.6
	I-CBT vs TAU	1	1.6	3.1	0.5	2	1.9	0.4	4.8	2.4	2.4	0.4	1.6	6.1	12.5	0	0.9	24.3	2.2	1	1	5	0	2.4	21.8	1.6
	I-CBT+P vs PBO	1	0.8	1.5	0.1	2	4.7	0.1	8.7	2	6.7	0.1	0.8	2	4.4	0	1.1	1.1	10.4	6.6	6.6	18.7	0	9.2	1.1	10.9
	P-CBT vs G-CBT+P	1	2.2	4.2	4.2	2.5	5.3	18.5	4	1.8	2.2	38.3	2.2	4.9	1.1	0	0.1	0.3	5.4	0.1	0.1	2	0	0.1	0.3	0.1
Indirect comparison	WL vs BIB-CBT		3.5	6.6	5.5	4.5	8.3	4.7	19.3	6	7.8	4.7	3.5	4.8	4	0	0.2	1	4.4	1.1	1.1	4.9	0	1.3	1	1.8
	WL vs I-BT+P		3	5.6	3.8	4.3	9.1	3.2	4.5	0.9	3.6	3.2	3	1.6	5.1	0	0.6	1.3	4.1	16.5	12.4	11.5	0	0	1.3	1.4
	WL vs I-CBT+P		3.6	6.7	4.2	5.4	8.7	3.6	7.6	1.6	6	3.6	3.6	1.2	6.9	0	0.3	1.8	1.5	2.9	2.9	18.4	0	2.7	1.8	4.8
	WL vs Int-CBT		3.1	5.9	3.4	5	6.9	2.9	5.6	2.9	2.7	2.9	3.1	0.2	6.9	0	0.1	1.8	0.9	1.6	1.6	6.9	27.3	3.7	1.8	2.6
	WL vs NT		5.6	10.5	3.5	3.5	5.9	3	1	0.6	0.4	3	5.6	6.9	6.4	32	1	1.6	1.5	0.7	0.7	2.3	0	1.6	1.6	1.2
	WL vs TAU		5	9.5	3.9	4.7	7.1	3.3	3.4	1.8	1.6	3.3	5	4.2	0.6	0	0.6	19.8	0.5	1.2	1.2	4.9	0	2.8	13.6	2
	BIB-CBT vs P-CBT		1.6	2.9	1.8	2.4	3.7	12.3	24.8	4.7	6.2	23.4	1.6	0.3	3.2	0	0.1	0.8	0.6	1.1	1.1	3.3	0	1.5	0.8	1.8
	BIB-CBT vs G-BT		11.2	0	2.8	2	3.9	2.4	16	5.5	6.9	2.4	17.2	7.8	6.9	0	0.7	1.8	3	0.5	0.5	5.4	0	0.2	1.8	0.9
	BIB-CBT vs G-CBT		2.5	4.7	2.1	1	2.4	1.8	20	7.3	9	1.8	2.5	13.1	11.7	0	1.3	3	3.1	0.4	0.4	8	0	0.4	3	0.6
	BIB-CBT vs I-BT+P		0.1	0.2	1.2	0.4	2.2	1	14.6	7.2	12.2	1	0.1	3	1.8	0	0.4	0.5	9.4	20.6	13.5	8.5	0	1.3	0.5	0.2
	BIB-CBT vs Int-CBT		0.3	0.5	1.2	1.4	0.1	1	11.5	8.7	10.2	1	0.3	4.2	4	0	0.1	1	5	0.8	0.8	12.2	30.4	3	1	1.3
	BIB-CBT vs NT		1.8	3.5	1.5	0.7	1.8	1.3	14.7	5.3	6.6	1.3	1.8	9.6	8.6	26.6	1	2.2	2.3	0.3	0.3	5.8	0	0.3	2.2	0.5
	BIB-CBT vs PBO		0.6	1.2	1.6	0.7	5.5	1.4	23.4	7.8	11.3	1.4	0.6	2.6	0.4	0	1.2	0.1	17.9	4.1	4.1	0.5	0	7.1	0.1	6.7
	BIB-CBT vs TAU		1.4	2.6	1.5	0.1	1.2	1.3	14.8	7.2	8.7	1.3	1.4	8.3	4.3	0	0.7	17.3	3.6	0.1	0.1	9	0	1.4	13.5	0.2
	G-BT vs G-CBT+P		13.3	0.3	4.1	1.5	4.4	3.5	6.7	3.1	3.6	3.5	20.2	11.9	5.8	0	0.8	1.5	7.3	0.3	0.3	4.5	0	1.2	1.5	0.5
	G-BT vs I-BT+P		9.7	0.2	1.6	2	5	1.3	3.3	0.5	2.9	1.3	14.8	4.6	7.2	0	0.9	1.9	4.2	14.3	10.2	10.8	0	0.8	1.9	0.7
	G-BT vs I-CBT		12.5	0.5	1.9	3.8	4.6	1.6	6	3	3	1.6	20	4.5	12.2	0	0.7	3.1	2	1.4	1.4	6.8	0	3.5	3.1	2.4
	G-BT vs I-CBT+P		10.8	0.2	1.8	2.8	4.4	1.6	6	1	5	1.6	16.9	4.7	9.3	0	0.8	2.4	2.1	2.2	2.1	17	0	1.5	2.4	3.5
	G-BT vs Int-CBT		9.4	0.4	1.5	2.9	3.5	1.2	4.6	2.3	2.3	1.2	15.1	3.4	9.2	0	0.5	2.4	1.5	1.1	1.1	5.2	24.5	2.6	2.4	1.8
	G-BT vs NT		12	4.8	1.6	1.6	2.7	1.4	0.5	0.3	0.2	1.4	25	3.1	2.9	37	0.4	0.7	0.7	0.3	0.3	1.1	0	0.7	0.7	0.5
	G-BT vs PBO		13.1	0.9	2	1.7	8.6	1.7	0.4	0.3	0.7	1.7	19	6.9	7.5	0	1.7	1.9	10.1	2.4	2.4	5.7	0	5.1	1.9	4
	G-BT vs TAU		11.9	2.9	1.7	2.5	3.4	1.4	2.8	1.4	1.4	1.4	22.3	0.2	3.6	0	0.1	21.4	0.5	0.8	0.8	3.5	0	1.9	12.9	1.3
	G-CBT vs I-BT+P		2.1	4	0.6	1.1	3.9	0.5	3.7	0.3	3.3	0.5	2.1	8.1	11.1	0	1.5	2.9	5.6	17.7	12.1	14	0	1.5	2.9	0.4
	G-CBT vs I-CBT+P		2.3	4.3	0.8	2	3.1	0.7	7.2	1	6.2	0.7	2.3	9	14.8	0	1.4	3.8	3.3	2.5	2.5	22.9	0	1.3	3.8	4

© 2018 Zhou X et al. JAMA Psychiatry.

	G-CBT vs Int-CBT		1.9	3.5	0.5	2.3	2.2	0.4	5.4	2.7	2.7	0.4	1.9	7	14.2	0	1.1	3.7	2.5	1.1	1.1	5.7	31.4	2.8	3.7	1.8
	G-CBT+P vs I-BT+P		0	0	1.9	1.2	2.4	1.7	11	3.7	7.4	1.7	0	5.4	4	0	0.4	1	12.7	18.8	13.9	10.1	0	0.1	1	1.4
	G-CBT+P vs I-CBT		0.5	0.9	2.5	2.8	0.4	2.1	15.1	7.2	7.8	2.1	0.5	8.4	7.9	0	0.1	2	10.8	2.1	2.1	13.4	0	5.5	2	3.5
	G-CBT+P vs I-CBT+P		0.3	0.6	2.1	2	1	1.8	15.3	4.8	10.5	1.8	0.3	6.8	6	0	0.1	1.6	10.7	3.2	3.2	17.7	0	3.3	1.6	5.3
	G-CBT+P vs Int-CBT		0.4	0.7	1.8	2	0.3	1.5	10.8	5.2	5.6	1.5	0.4	6.1	5.7	0	0.1	1.5	7.8	1.5	1.5	9.6	28	4	1.5	2.5
	G-CBT+P vs NT		2.2	4.1	2.4	0.1	1.7	2.1	6.4	3	3.4	2.1	2.2	13.2	7.5	30.1	1.1	1.9	6	0.6	0.6	4.9	0	1.6	1.9	0.9
	G-CBT+P vs TAU		1.6	2.9	2.4	0.9	1	2	9.1	4.3	4.7	2	1.6	11.1	2.2	0	0.7	18.5	7.4	1	1	7.5	0	2.9	13.4	1.7
	I-BT+P vs I-CBT		0.5	0.9	0.2	1.4	2.5	0.2	2.1	3.1	1	0.2	0.5	2	3.3	0	0.6	0.8	4.5	25.8	15.2	26.7	0	5.7	0.8	1.9
	I-BT+P vs Int-CBT		0.4	0.7	0.1	1	1.8	0.1	1.5	2.2	0.7	0.1	0.4	1.4	2.3	0	0.5	0.6	3.2	18.3	10.8	19	29.1	4	0.6	1.4
	I-BT+P vs NT		1.7	3.1	0.5	0.9	3	0.4	2.8	0.3	2.6	0.4	1.7	6.2	8.6	22.9	1.1	2.2	4.3	13.6	9.3	10.8	0	1.2	2.2	0.3
	I-BT+P vs TAU		1.3	2.4	0.4	0.2	2.8	0.3	1.4	0.6	2.1	0.3	1.3	4.9	5.2	0	1	14.8	4.2	16.5	10.6	14.7	0	2.3	12.2	0.3
	I-CBT vs NT		1.9	3.5	0.5	2.3	2.2	0.4	5.4	2.7	2.7	0.4	1.9	7	14.2	31.4	1.1	3.7	2.5	1.1	1.1	5.7	0	2.8	3.7	1.8
	I-CBT+P vs Int-CBT		0.2	0.4	0.3	0.7	0.8	0.2	1.3	2.4	3.7	0.2	0.2	1.3	1.7	0	0.2	0.4	0.5	1.4	1.4	35.1	42.6	2.2	0.4	2.3
	I-CBT+P vs NT		1.7	3.2	0.6	1.5	2.3	0.5	5.3	0.8	4.6	0.5	1.7	6.7	10.9	26.3	1	2.8	2.4	1.8	1.8	16.9	0	0.9	2.8	3
	I-CBT+P vs TAU		1.4	2.6	0.5	0.9	2	0.5	4.5	0.2	4.7	0.5	1.4	5.5	7.8	0	0.9	18.2	2	1.8	1.8	24.2	0	0	15.6	3
	Int-CBT vs NT		1.4	2.7	0.4	1.8	1.6	0.3	4.1	2	2.1	0.3	1.4	5.3	10.8	23.9	0.8	2.8	1.9	0.9	0.9	4.3	23.9	2.1	2.8	1.4
	Int-CBT vs PBO		0.7	1.3	0.1	1.9	3.8	0.1	5.2	3.1	2.1	0.1	0.7	2.4	4.3	0	1	1.1	7.7	3.7	3.7	11.8	30.2	8.1	1.1	6
	Int-CBT vs TAU		1.1	2.1	0.3	1.4	1.3	0.3	3.3	1.6	1.7	0.3	1.1	4.2	8.6	0	0.6	16.7	1.5	0.7	0.7	3.4	31.6	1.7	14.9	1.1
	NT vs PBO		2.4	4.5	0.6	0.3	5.6	0.5	0	0.5	0.5	0.5	2.4	8.6	9	28.7	1.9	2.3	9.6	2.4	2.4	6	0	5.1	2.3	4
	NT vs TAU		0.9	1.6	0.2	1.1	1	0.2	2.5	1.2	1.3	0.2	0.9	3.2	6.6	35.9	0.5	23.1	1.1	0.5	0.5	2.6	0	1.3	12.8	0.9
	PBO vs TAU		1.9	3.6	0.4	0.6	5.3	0.3	2.2	1.6	0.6	0.3	1.9	6.7	4.1	0	1.7	17.9	9.6	3.2	3.2	8.9	0	6.8	13.9	5.2
	P-CBT vs G-BT		13.5	2.5	1.5	0.1	1.1	13.3	4	1.9	2.1	17.5	17.3	8.2	4.7	0	0.7	1.2	3.8	0.3	0.3	3	0	1	1.2	0.6
	P-CBT vs G-CBT		3.9	7.3	0.2	1.4	1.2	13.6	4.9	2.4	2.6	20.9	3.9	12.1	8	0	1.2	2.1	3.6	0.7	0.7	4.4	0	1.8	2.1	1.1
	P-CBT vs I-BT+P		1	1.9	0.4	2	4.2	9.5	6.7	2	4.7	15.8	1	2	3.6	0	0.4	0.9	7.4	14.6	10.8	8.7	0	0.1	0.9	1.1
	P-CBT vs I-CBT		1.6	2.9	0.3	3.4	3.1	11.3	9.4	4.6	4.8	18.4	1.6	3.8	6.6	0	0	1.7	5.4	1.7	1.7	9.2	0	4.2	1.7	2.7
	P-CBT vs I-CBT+P		1.3	2.5	0.4	2.7	3.4	10.4	9.6	2.7	6.9	17.3	1.3	2.7	5.1	0	0.1	1.3	5.4	2.5	2.5	14.3	0	2.4	1.3	4
	P-CBT vs Int-CBT		1.2	2.3	0.2	2.6	2.4	8.7	7.2	3.5	3.7	14.2	1.2	3	5.1	0	0	1.3	4.2	1.3	1.3	7.1	22.9	3.2	1.3	2.1
	P-CBT vs NT		2.9	5.4	0.2	1.1	0.9	10.1	3.7	1.8	1.9	15.5	2.9	9	5.9	25.6	0.9	1.5	2.7	0.5	0.5	3.3	0	1.3	1.5	0.8
	P-CBT vs PBO		1	2	0.4	1.8	8.3	13.6	5.2	1.9	3.3	22.3	1	1.8	2.9	0	1.1	0.8	15.7	2.3	2.3	2.9	0	4.5	0.8	3.9
	P-CBT vs TAU		2.3	4.4	0	1.9	1.7	10.3	5.6	2.7	2.9	16.2	2.3	6.9	1.3	0	0.5	15.5	3.6	0.9	0.9	5.3	0	2.3	11	1.5
**Total**			3.6	3.2	1.6	2	3.7	3.3	7.1	2.9	4.2	4.5	4.8	5.6	6.4	5.3	0.7	4.5	5.2	4.4	3.4	9.3	5.3	2.7	3.7	2.3

© 2018 Zhou X et al. JAMA Psychiatry.

Mean overall change in symptoms at long-term follow-up

	Comparison	N of trials	WL vs G-CBT	WL vs PBO	G-BT vs G-CBT	G-CBT vs G-CBT+P	G-CBT vs I-CBT	G-CBT vs PBO	G-CBT vs TAU	I-BT+P vs I-CBT+P	I-BT+P vs PBO	I-CBT vs I-CBT+P	I-CBT vs PBO	I-CBT+P vs PBO	P-CBT vs G-CBT+P
Direct comparison	WL vs G-CBT	1	17	29.6	0	0	14.7	14.9	0	1.4	1.4	7.8	6.8	6.5	0
	WL vs PBO	1	16.4	54	0	0	8.1	8.3	0	0.8	0.8	4.3	3.8	3.6	0
	P-CBT vs G-CBT+P	1	0	0	0	0	0	0	0	0	0	0	0	0	100
	G-BT vs G-CBT	1	0	0	100	0	0	0	0	0	0	0	0	0	0
	G-CBT vs G-CBT+P	3	0	0	0	100	0	0	0	0	0	0	0	0	0
	G-CBT vs I-CBT	2	5.7	5.7	0	0	30.6	18.6	0	2.3	2.3	12.9	11.3	10.7	0
	G-CBT vs PBO	1	7.3	7.3	0	0	23.5	23.9	0	2.2	2.2	12.5	10.9	10.3	0
	G-CBT vs TAU	1	0	0	0	0	0	0	100	0	0	0	0	0	0
	I-BT+P vs I-CBT+P	1	0.5	0.5	0	0	2.1	1.6	0	42.7	23	8.8	6.7	14.2	0
	I-BT+P vs PBO	1	0.6	0.6	0	0	2.7	2.1	0	29.7	25.8	11.3	8.6	18.4	0
	I-CBT vs I-CBT+P	6	0.9	0.9	0	0	3.7	2.8	0	2.7	2.7	62.2	11.6	12.6	0
	I-CBT vs PBO	1	1.8	1.8	0	0	7.5	5.8	0	4.7	4.7	27.3	23.9	22.6	0
	I-CBT+P vs PBO	2	1.2	1.2	0	0	5.2	4	0	7.4	7.4	21.7	16.5	35.3	0
Indirect comparison	WL vs G-BT		11.6	20.2	31.8	0	10	10.2	0	0.9	0.9	5.3	4.7	4.4	0
	WL vs G-CBT+P		11.6	20.2	0	31.8	10	10.2	0	0.9	0.9	5.3	4.7	4.4	0
	WL vs I-BT+P		8.4	26.1	0	0	5.7	2.8	0	18.9	15.7	9.2	3.5	9.7	0
	WL vs I-CBT		10.5	29.6	0	0	9.8	0.8	0	2.8	2.8	16.2	14.2	13.4	0
	WL vs I-CBT+P		9.8	29.2	0	0	7.7	2.2	0	4.1	4.1	15.6	7.9	19.4	0
	WL vs P-CBT		8.8	15.3	0	24.1	7.6	7.7	0	0.7	0.7	4	3.5	3.3	24.1
	WL vs TAU		11.6	20.2	0	0	10	10.2	31.8	0.9	0.9	5.3	4.7	4.4	0
	G-BT vs G-CBT+P		0	0	50	50	0	0	0	0	0	0	0	0	0
	G-BT vs I-BT+P		3.3	3.3	26.7	0	12.8	10.7	0	15.4	11.4	11.6	1.2	3.8	0
	G-BT vs I-CBT		3.7	3.7	35.4	0	19.8	12	0	1.5	1.5	8.4	7.3	6.9	0
	G-BT vs I-CBT+P		3.5	3.5	30.8	0	15.7	11.6	0	2.3	2.3	17.5	1.7	11	0
	G-BT vs PBO		4.7	4.7	35.3	0	15.2	15.4	0	1.4	1.4	8.1	7.1	6.7	0
	G-BT vs TAU		0	0	50	0	0	0	50	0	0	0	0	0	0
	G-CBT vs I-BT+P		4.4	4.4	0	0	17.5	14.6	0	21	15.5	15.8	1.7	5.2	0
	G-CBT vs I-CBT+P		5.1	5.1	0	0	22.7	16.7	0	3.4	3.4	25.2	2.5	15.9	0
	G-CBT+P vs I-BT+P		3.3	3.3	0	26.7	12.8	10.7	0	15.4	11.4	11.6	1.2	3.8	0
	G-CBT+P vs I-CBT		3.7	3.7	0	35.4	19.8	12	0	1.5	1.5	8.4	7.3	6.9	0
	G-CBT+P vs I-CBT+P		3.5	3.5	0	30.8	15.7	11.6	0	2.3	2.3	17.5	1.7	11	0
	G-CBT+P vs PBO		4.7	4.7	0	35.3	15.2	15.4	0	1.4	1.4	8.1	7.1	6.7	0
	G-CBT+P vs TAU		0	0	0	50	0	0	50	0	0	0	0	0	0
	I-BT+P vs I-CBT		0.9	0.9	0	0	3.7	2.8	0	28.6	17.9	31.1	11.7	2.5	0
	I-BT+P vs TAU		3.3	3.3	0	0	12.8	10.7	26.7	15.4	11.4	11.6	1.2	3.8	0
	I-CBT vs TAU		3.7	3.7	0	0	19.8	12	35.4	1.5	1.5	8.4	7.3	6.9	0
	I-CBT+P vs TAU		3.5	3.5	0	0	15.7	11.6	30.8	2.3	2.3	17.5	1.7	11	0
	PBO vs TAU		4.7	4.7	0	0	15.2	15.4	35.3	1.4	1.4	8.1	7.1	6.7	0
	P-CBT vs G-BT		0	0	33.3	33.3	0	0	0	0	0	0	0	0	33.3
	P-CBT vs G-CBT		0	0	0	50	0	0	0	0	0	0	0	0	50
	P-CBT vs I-BT+P		2.6	2.6	0	21.1	10.1	8.4	0	12.1	9	9.1	1	3	21.1
	P-CBT vs I-CBT		2.7	2.7	0	26.2	14.6	8.9	0	1.1	1.1	6.2	5.4	5.1	26.2
	P-CBT vs I-CBT+P		2.7	2.7	0	23.6	12	8.8	0	1.8	1.8	13.3	1.3	8.4	23.6
	P-CBT vs PBO		3.5	3.5	0	26.1	11.2	11.4	0	1	1	6	5.2	4.9	26.1
	P-CBT vs TAU		0	0	0	33.3	0	0	33.3	0	0	0	0	0	33.3
**Total**			4.5	7.4	7.7	13.8	10.8	8.5	7.7	5.6	4.4	10.2	4.6	6.8	7.7

© 2018 Zhou X et al. JAMA Psychiatry.

b. Contributions summary of risk of bias assessments for each outcome

Risk of bias assessment[*] for any direct comparisons included in the network for each outcome

	Mean overall change in symptoms at post-treatment	Mean overall change in symptoms at follow-up	All-cause discontinuation	Mean overall change in quality of life and functional improvement	Mean overall change in symptoms at short-term follow-up	Mean overall change in symptoms at long-term follow-up
WL vs BIB-CBT	Moderate	NA	Moderate	NA	NA	NA
WL vs G-BT	Moderate	NA	Moderate	NA	Moderate	NA
WL vs G-CBT	Moderate	Moderate	Moderate	Moderate	Moderate	Moderate
WL vs G-CBT+P	Moderate	Moderate	Moderate	Moderate	Moderate	NA
WL vs I-BT+P	Moderate	NA	NA	NA	NA	NA
WL vs I-CBT	Moderate	High	High	Moderate	High	NA
WL vs I-CBT+P	Moderate	NA	Moderate	High	NA	NA
WL vs I+G-BT	Moderate	NA	Moderate	Moderate	NA	NA
WL vs I+G-CBT	Moderate	NA	Low	Low	NA	NA
WL vs Int-CBT	Moderate	NA	Moderate	Moderate	NA	NA
WL vs PBO	Moderate	Moderate	NA	NA	Moderate	Moderate
WL vs P-CBT	Moderate	High	Moderate	High	High	NA
BIB-CBT vs G-CBT+P	Moderate	Moderate	Moderate	NA	Moderate	NA
BIB-CBT vs I-CBT	Moderate	Moderate	Moderate	Moderate	Moderate	NA
BIB-CBT vs I-CBT+P	High	High	Moderate	NA	High	NA
G-BT vs G-CBT	Moderate	Moderate	NA	NA	Moderate	Moderate
G-CBT vs G-CBT+P	Moderate	Moderate	Moderate	Moderate	Moderate	Moderate
G-CBT vs I-CBT	Moderate	High	High	High	Moderate	High
G-CBT vs NT	Moderate	Moderate	Moderate	NA	Moderate	NA
G-CBT vs PBO	Moderate	Moderate	Moderate	Moderate	Moderate	Moderate
G-CBT vs TAU	Moderate	Moderate	Moderate	NA	Moderate	Moderate
G-CBT+P vs I-CBT+P	Moderate	NA	Moderate	Moderate	NA	NA
G-CBT+P vs Int-CBT	Moderate	NA	Moderate	NA	NA	NA
G-CBT+P vs PBO	High	High	High	NA	High	NA

© 2018 Zhou X et al. JAMA Psychiatry.

I-BT+P vs I-CBT	Moderate	NA	Moderate	NA	NA	NA
I-BT+P vs I-CBT+P	Moderate	Moderate	Moderate	NA	Moderate	Moderate
I-BT+P vs PBO	Moderate	Moderate	Moderate	NA	Moderate	Moderate
I-CBT vs I-CBT+P	Moderate	Moderate	Moderate	Moderate	Moderate	Moderate
I-CBT vs Int-CBT	Moderate	Moderate	Moderate	Moderate	Moderate	NA
I-CBT vs PBO	Moderate	Moderate	Moderate	High	Moderate	Moderate
I-CBT vs TAU	High	High	High	NA	High	NA
I-CBT+P vs Int-CBT	High	NA	High	High	NA	NA
I-CBT+P vs PBO	Moderate	Moderate	Moderate	NA	Moderate	Moderate
I-CBT+P vs TAU	Moderate	NA	Moderate	Low	NA	NA
I+G-BT vs PBO	Moderate	NA	Moderate	Moderate	NA	NA
Int-CBT vs PBO	Moderate	NA	Moderate	Moderate	NA	NA
Int-CBT vs TAU	Moderate	NA	Moderate	Moderate	NA	NA
P-CBT vs G-CBT	Moderate	NA	NA	NA	NA	NA
P-CBT vs G-CBT+P	Moderate	Moderate	Moderate	NA	Moderate	Moderate

[*]The overall risk of bias for each direct comparison was based on the risk of bias of individual studies contributing to that comparison, and was classified as high, moderate or low. We rated study quality as follows: *high risk study* (3 or more items rated as high risk of bias); *low risk study* (5 or more items rated as low risk and no more than one as high risk); *moderate risk study* (all remaining situations).
BIB-CBT =bibliotherapy cognitive-behavioral therapy, G-BT=group behavioral therapy, G-CBT=group cognitive-behavioral therapy, G-CBT+P=group cognitive-behavioral therapy with parental involvement, I-BT+P=individual behavioral therapy with parental involvement, I-CBT=individual cognitive-behavioral therapy, I-CBT+P=individual cognitive-behavioral therapy with parental involvement, I+G-BT=individual and group behavioral therapy, I+G-CBT=individual and group cognitive-behavioral therapy, Int-CBT=internet-assisted cognitive-behavioral therapy, NA= no available, NT=no-treatment, PBO=psychological placebo, P-CBT=parent-only cognitive-behavioral therapy, TAU=treatment as usual, WL=waitlist.

© 2018 Zhou X et al. JAMA Psychiatry.

The contribution of direct comparisons to mixed or indirect comparisons by risk of bias classification and outcome*

	Mean overall change in symptoms at post-treatment			Mean overall change in symptoms at follow-up			All-cause discontinuation			Mean overall change in quality of life and functional improvement			Mean overall change in symptoms at short-term follow-up			Mean overall change in symptoms at long-term follow-up		
	Low (%)	Moderate (%)	High (%)	Low (%)	Moderate (%)	High (%)	Low (%)	Moderate (%)	High (%)	Low (%)	Moderate (%)	High (%)	Low (%)	Moderate (%)	High (%)	Low (%)	Moderate (%)	High (%)
WL vs BIB-CBT	0	87.3	12.6	0	74.2	25.6	0	95.4	4.6	1.1	89.2	9.9	0	77.6	22.4	…	…	…
WL vs G-BT	0	97.5	2.5	0	81	19	0	99.8	0	…	…	…	0	90.8	9.1	0	90	10
WL vs G-CBT	0	96.7	3.5	0	72.2	27.6	0	86.9	13.2	0.8	79.6	19.8	0	85.1	14.7	0	85.4	14.7
WL vs G-CBT+P	0	92.6	7.5	0	68.5	31.7	0	92.3	7.5	1.4	91.7	6.9	0	72.6	27.7	0	90	10
WL vs I+G-BT	0	95	5	…	…	…	0	91.6	8.5	0.2	87.7	12	…	…	…	…	…	…
WL vs I+G-CBT	0	100	0	…	…	…	99.8	0	0	100	0	0	…	…	…	…	…	…
WL vs I-BT+P	0	89.1	11.2	0	78.7	21.3	0	90.5	9.5	…	…	…	0	83.5	16.5	0	94.3	5.7
WL vs I-CBT	0	94.6	5.7	0	73.6	26.5	0	79.9	20.3	1.6	83.9	14.5	0	81.6	18.5	0	90.3	9.8
WL vs I-CBT+P	0	94.6	5.6	0	75.4	24.9	0	87.6	12.2	3.6	81.2	15.3	0	81.5	18.3	0	92.3	7.7
WL vs Int-CBT	0	93	7	0	80.7	19	0	88.9	11.2	5.8	78.6	15.8	0	86.5	13.3	…	…	…
WL vs NT	0	87.9	12.2	0	81	19	0	90.9	9	…	…	…	0	90.1	10	…	…	…
WL vs PBO	0	86.9	13	0	69.6	30.5	0	87.3	12.7	0.4	79.2	20.3	0	75.2	24.6	0	92	8.1
WL vs P-CBT	0	95.2	4.7	0	60.7	39.2	0	96.5	3.5	0	0	100	0	64.8	35.2	0	92.2	7.6
WL vs TAU	0	88.5	11.6	0	72.9	27.2	0	88.2	12.3	17.4	75.7	6.7	0	76.3	23.7	0	90	10
BIB-CBT vs G-BT	0	92.1	8	0	82	17.9	0	97.4	2.9	…	…	…	0	83.7	16.1	…	…	…
BIB-CBT vs G-CBT	0	91.2	8.7	0	74.9	24.9	0	90.4	9.4	0.5	79.5	20.1	0	82.2	17.9	…	…	…
BIB-CBT vs G-CBT+P	0	90.9	9.1	0	76.6	23.5	0	96.5	3.6	0.4	91.9	7.8	0	80.8	19.2	…	…	…
BIB-CBT vs I+G-BT	0	92.8	7.1	…	…	…	0	93.7	6.3	1	86	13	…	…	…	…	…	…
BIB-CBT vs I+G-CBT	0	90.9	9.3	…	…	…	38.9	58.5	2.9	25.2	67.4	7.5	…	…	…	…	…	…
BIB-CBT vs I-BT+P	0	89.5	10.7	0	75	25	0	92.5	7.1	…	…	…	0	76.4	23.5	…	…	…
BIB-CBT vs I-CBT	0	90.6	9.1	0	68.5	31.5	0	84.6	15.2	0	100	0	0	73.1	26.8	…	…	…
BIB-CBT vs I-CBT+P	0	86.5	13.3	0	67.2	32.9	0	91.3	8.5	1.9	90.2	8	0	70	30.2	…	…	…
BIB-CBT vs Int-CBT	0	90.7	9	0	77.9	21.9	0	93.3	6.7	5.3	79.1	15.6	0	81.4	18.6	…	…	…
BIB-CBT vs NT	0	88.7	11.4	0	82	17.9	0	92.9	6.9	…	…	…	0	86.9	13.1	…	…	…
BIB-CBT vs PBO	0	84.8	15.6	0	69	30.9	0	90.3	9.8	1.5	74.1	24.3	0	68.9	31.4	…	…	…
BIB-CBT vs P-CBT	0	97.5	2.5	0	74.6	25.7	0	98	1.7	0.8	67.4	31.9	0	77.7	22.3	…	…	…

© 2018 Zhou X et al. JAMA Psychiatry.

BIB-CBT vs TAU	0	93.3	6.7	0	69.4	30.9	0	90.9	9.2	15.3	77.3	7.5	0	72.8	27.2	…	…	…
G-BT vs G-CBT	0	90.3	9.4	0	100	0	0	90.9	9	…	…	…	0	92.7	7.4	0	100	0
G-BT vs G-CBT+P	0	96	3.8	0	80.8	18.9	0	95	4.6	…	…	…	0	82.4	17.4	0	100	0
G-BT vs I+G-BT	0	96.4	3.6	…	…	…	0	93.8	6.3	…	…	…	…	…	…	…	…	…
G-BT vs I+G-CBT	0	97.6	1.9	…	…	…	50	50	0	…	…	…	…	…	…	…	…	…
G-BT vs I-BT+P	0	95.9	4.1	0	82.1	17.9	0	92.6	7	…	…	…	0	87.8	12.3	0	87.4	12.8
G-BT vs I-CBT	0	96.3	3.9	0	77.4	22.6	0	86	14	…	…	…	0	86.1	13.5	0	80.4	19.8
G-BT vs I-CBT+P	0	95.9	4.4	0	78.8	20.8	0	91.9	8.2	…	…	…	0	86.2	13.9	0	84.2	15.7
G-BT vs Int-CBT	0	94.5	5.3	0	82.4	17.5	0	93.2	6.8	…	…	…	0	89.8	10.3	…	…	…
G-BT vs NT	0	99.8	0.2	0	100	0	0	93.5	6.8	…	…	…	0	95.3	4.6	…	…	…
G-BT vs PBO	0	93	6.9	0	78.2	21.8	0	91.2	9	…	…	…	0	83.6	16.1	0	84.8	15.2
G-BT vs TAU	0	93.1	7	0	79.3	20.8	0	91.4	8.5	…	…	…	0	81.4	18.7	0	100	0
G-CBT vs G-CBT+P	0	84.8	15.2	0	71.5	28.5	0	88.8	11.2	0.1	82.3	17.6	0	80.8	19.3	0	100	0
G-CBT vs I+G-BT	0	95.1	4.6	…	…	…	0	93.6	6.3	0.8	79.7	19.6	…	…	…	…	…	…
G-CBT vs I+G-CBT	0	97.5	2.5	…	…	…	31.8	59.2	9	30.4	55.8	13.8	…	…	…	…	…	…
G-CBT vs I-BT+P	0	95	5.3	0	76.6	23.2	0	92.7	7.4	…	…	…	0	86.5	13.4	0	82.6	17.5
G-CBT vs I-CBT	0	97.8	2.3	0	68.2	32.1	0	82.2	17.9	0.7	69.4	29.9	0	82.6	16.9	0	69.5	30.6
G-CBT vs I-CBT+P	0	94.1	5.9	0	71.5	28.4	0	90.1	10	2.4	71.9	25.9	0	83.9	16	0	77.3	22.7
G-CBT vs Int-CBT	0	93	6.9	0	77.4	22.6	0	87	12.7	5.2	69.5	25.5	0	88.4	11.6	…	…	…
G-CBT vs NT	0	97.3	2.9	0	99.9	0	0	99.9	0	…	…	…	0	99.9	0	…	…	…
G-CBT vs PBO	0	99.5	0.6	0	69.5	30.4	0	87.8	12.1	1.1	65.9	32.9	0	81.5	18.4	0	76.6	23.5
G-CBT vs TAU	0	94.6	5.5	0	67.8	32.1	0	88.6	11.6	15.3	67.4	17.1	0	74.2	25.7	0	100	0
G-CBT+P vs I+G-BT	0	92.7	7.2	…	…	…	0	92.6	7.8	0.9	88.8	10.3	…	…	…	…	…	…
G-CBT+P vs I+G-CBT	0	94.7	5.3	…	…	…	37.2	57.8	4.6	41.6	54.2	4	…	…	…	…	…	…
G-CBT+P vs I-BT+P	0	91.7	8.1	0	72.8	27.1	0	90.8	9.1	…	…	…	0	75.8	24	0	87.4	12.8
G-CBT+P vs I-CBT	0	91.9	8.2	0	67.9	32.2	0	82.3	17.7	0.5	88.3	11.1	0	74.2	25.5	0	80.4	19.8
G-CBT+P vs I-CBT+P	0	94.7	5.1	0	68.5	31.7	0	88.6	11.5	2.7	83.7	13.3	0	73.4	26.6	0	84.2	15.7
G-CBT+P vs Int-CBT	0	100	0	0	77	23	0	90.8	9.2	5.8	78.6	15.6	0	81.6	18.4	…	…	…
G-CBT+P vs NT	0	96.2	3.6	0	80.8	18.9	0	92.1	7.9	…	…	…	0	86.5	13.5	…	…	…
G-CBT+P vs PBO	0	90.9	9.4	0	61.2	38.9	0	88	11.8	1	80.4	18.5	0	62.7	37.2	0	84.8	15.2
G-CBT+P vs TAU	0	87.2	12.6	0	68	31.9	0	88.8	10.9	16.9	75.8	7.4	0	71.5	28.4	0	100	0
I+G-BT vs I+G-CBT	0	96.7	3.3	…	…	…	25.8	68	6.3	35.1	57.2	7.7	…	…	…	…	…	…

© 2018 Zhou X et al. JAMA Psychiatry.

I+G-BT vs Int-CBT	0	91.2	8.8	⋯	⋯	⋯	0	90.8	9.1	4.2	79	16.8	⋯	⋯	⋯	⋯	⋯	⋯
I+G-BT vs NT	0	96.7	3.5	⋯	⋯	⋯	0	95.8	4.5	⋯	⋯	⋯	⋯	⋯	⋯	⋯	⋯	⋯
I+G-BT vs PBO	0	90.4	9.2	⋯	⋯	⋯	0	95.7	4.4	0.3	86.8	13.3	⋯	⋯	⋯	⋯	⋯	⋯
I+G-BT vs TAU	0	91.5	8.7	⋯	⋯	⋯	0	92.4	7.4	13.5	77.1	9.7	⋯	⋯	⋯	⋯	⋯	⋯
I+G-CBT vs Int-CBT	0	94.6	5.5	⋯	⋯	⋯	39.8	53.4	6.8	35.1	53.9	10.9	⋯	⋯	⋯	⋯	⋯	⋯
I+G-CBT vs NT	0	97.8	2	⋯	⋯	⋯	24.1	69.4	6.8	⋯	⋯	⋯	⋯	⋯	⋯	⋯	⋯	⋯
I+G-CBT vs PBO	0	93.6	6.4	⋯	⋯	⋯	29	62.2	9	29.4	56	14.5	⋯	⋯	⋯	⋯	⋯	⋯
I+G-CBT vs TAU	0	94.5	5.8	⋯	⋯	⋯	29.3	62	8.5	38.9	56.1	5	⋯	⋯	⋯	⋯	⋯	⋯
I-BT+P vs I+G-BT	0	97.2	2.8	⋯	⋯	⋯	0	96.3	3.9	⋯	⋯	⋯	⋯	⋯	⋯	⋯	⋯	⋯
I-BT+P vs I+G-CBT	0	95.9	3.9	⋯	⋯	⋯	25.7	66.9	7	⋯	⋯	⋯	⋯	⋯	⋯	⋯	⋯	⋯
I-BT+P vs I-CBT	0	89.1	11	0	93	7.1	0	89.4	10.4	⋯	⋯	⋯	0	92	7.9	0	96.4	3.7
I-BT+P vs I-CBT+P	0	88.7	11.6	0	92.8	7.2	0	95.5	4.4	⋯	⋯	⋯	0	89.4	10.6	0	98	2.1
I-BT+P vs Int-CBT	0	90.9	9.2	0	95	5	0	90.1	10.1	⋯	⋯	⋯	0	94.6	5.6	⋯	⋯	⋯
I-BT+P vs NT	0	96.1	4	0	82.1	17.9	0	94.8	5.4	⋯	⋯	⋯	0	89.7	10.4	⋯	⋯	⋯
I-BT+P vs PBO	0	87.2	12.8	0	91	9	0	94.4	5.4	⋯	⋯	⋯	0	87.2	12.9	0	97.1	2.7
I-BT+P vs TAU	0	90.1	9.9	0	75.5	24.3	0	90.9	9	⋯	⋯	⋯	0	80.8	19	0	87.4	12.8
I-CBT vs I+G-BT	0	95.3	4.6	⋯	⋯	⋯	0	87.7	12.1	1.4	80.8	17.7	⋯	⋯	⋯	⋯	⋯	⋯
I-CBT vs I+G-CBT	0	96.2	3.9	⋯	⋯	⋯	31.3	54.7	14	33.4	56.9	9.9	⋯	⋯	⋯	⋯	⋯	⋯
I-CBT vs I-CBT+P	0	94.7	5.1	0	90.8	9.3	0	85.1	15	3.1	83.5	13.3	0	90.5	9.7	0	96.4	3.7
I-CBT vs Int-CBT	0	97.6	2.6	0	100	0	0	81.1	18.8	7.8	69.1	23.1	0	100	0	⋯	⋯	⋯
I-CBT vs NT	0	96.1	3.8	0	77.4	22.6	0	87.9	12.4	⋯	⋯	⋯	0	88.4	11.6	⋯	⋯	⋯
I-CBT vs PBO	0	95	4.8	0	84.6	15.6	0	83.6	16.7	2.2	61	36.8	0	81.4	18.4	0	92.6	7.5
I-CBT vs TAU	0	94.1	6	0	61.8	38.2	0	82.1	17.9	21.4	68.2	10.5	0	71.1	28.8	0	80.4	19.8
I-CBT+P vs I+G-BT	0	94	6.3	⋯	⋯	⋯	0	93.8	6.2	2.7	80.3	17.1	⋯	⋯	⋯	⋯	⋯	⋯
I-CBT+P vs I+G-CBT	0	95.3	4.5	⋯	⋯	⋯	32.7	59	8.2	33.5	56	10.6	⋯	⋯	⋯	⋯	⋯	⋯
I-CBT+P vs Int-CBT	0	94.4	5.5	0	94.7	5.1	0	87.5	12.5	11.4	60.7	27.9	0	94.4	5.5	⋯	⋯	⋯
I-CBT+P vs NT	0	95.7	4.2	0	78.8	20.8	0	92.9	7	⋯	⋯	⋯	0	88.2	11.8	⋯	⋯	⋯
I-CBT+P vs PBO	0	90.1	9.7	0	83.9	16.2	0	91.2	8.8	3.5	67.1	29.4	0	79.3	20.3	0	94.7	5.2
I-CBT+P vs TAU	0	91.5	8.6	0	69.3	30.8	0	89.4	10.6	32.8	51.7	15.5	0	76.3	23.7	0	84.2	15.7
Int-CBT vs NT	0	94.5	5.3	0	82.4	17.5	0	90.7	9.3	⋯	⋯	⋯	0	91	8.9	⋯	⋯	⋯
Int-CBT vs PBO	0	89	11.2	0	90	10	0	87.2	12.7	4.8	67	28.5	0	87.3	12.9	⋯	⋯	⋯
Int-CBT vs TAU	0	88.8	11.5	0	73.6	26.5	0	86.8	13.2	22.2	63.3	14.4	0	80.4	19.8	⋯	⋯	⋯

© 2018 Zhou X et al. JAMA Psychiatry.

NT vs PBO	0	93.6	6.7	0	78.2	21.8	0	92.2	7.6	⋯	⋯	⋯	0	86.9	13.2	⋯	⋯	⋯
NT vs TAU	0	93	6.9	0	79.3	20.8	0	91.9	8.2	⋯	⋯	⋯	0	83.6	16.5	⋯	⋯	⋯
PBO vs TAU	0	85.6	14.4	0	69.9	30.1	0	89.1	11.2	15.6	66.4	18	0	74.9	25	0	84.8	15.2
P-CBT vs G-BT	0	84.2	15.9	0	77.9	21.8	0	97.5	2.2	⋯	⋯	⋯	0	79.3	20.5	0	99.9	0
P-CBT vs G-CBT	0	84.2	15.7	0	70.2	30	0	90.2	9.3	0.5	55.8	43.7	0	76.8	23.3	0	100	0
P-CBT vs G-CBT+P	0	87.6	12.5	0	68.9	30.9	0	96.1	3.5	0.8	54.2	44.8	0	71	28.9	0	100	0
P-CBT vs I+G-BT	0	97.6	1.9	⋯	⋯	⋯	0	93.3	6.6	0.2	57.2	42.6	⋯	⋯	⋯	⋯	⋯	⋯
P-CBT vs I+G-CBT	0	94.7	5.1	⋯	⋯	⋯	36.8	60.7	2.2	50	0	50	⋯	⋯	⋯	⋯	⋯	⋯
P-CBT vs I-BT+P	0	96.6	3.4	0	72.4	27.7	0	92.3	7.8	⋯	⋯	⋯	0	75.2	24.5	0	90	10.1
P-CBT vs I-CBT	0	94.4	5.9	0	68	32	0	85.2	14.7	1.1	56.9	42.2	0	73.5	26.6	0	85.6	14.6
P-CBT vs I-CBT+P	0	94.3	5.6	0	68.8	31.2	0	91	9.1	2.5	56	41.6	0	73.4	26.7	0	88	12
P-CBT vs Int-CBT	0	93.2	6.8	0	75.4	24.7	0	93	7.1	4	53.9	42	0	79.5	20.5	⋯	⋯	⋯
P-CBT vs NT	0	91.6	8.3	0	77.9	21.8	0	93	7	⋯	⋯	⋯	0	82.6	17.3	⋯	⋯	⋯
P-CBT vs PBO	0	98.8	1.7	0	62.8	37.2	0	90	9.8	0.3	56	43.6	0	64.6	35.2	0	88.7	11.2
P-CBT vs TAU	0	91.4	8.9	0	68.4	31.7	0	90.9	9.1	12.9	56.1	31	0	70.3	29.7	0	99.9	0
Total	0	94.8	5	0	76.5	23.3	4.8	86.2	8.8	10.9	69.1	20.1	0	81.3	18.4	0	88.9	10.8

© 2018 Zhou X et al. JAMA Psychiatry.

Contributions summary of studies at risk of bias assessment for the mean overall change in symptoms at post-treatment

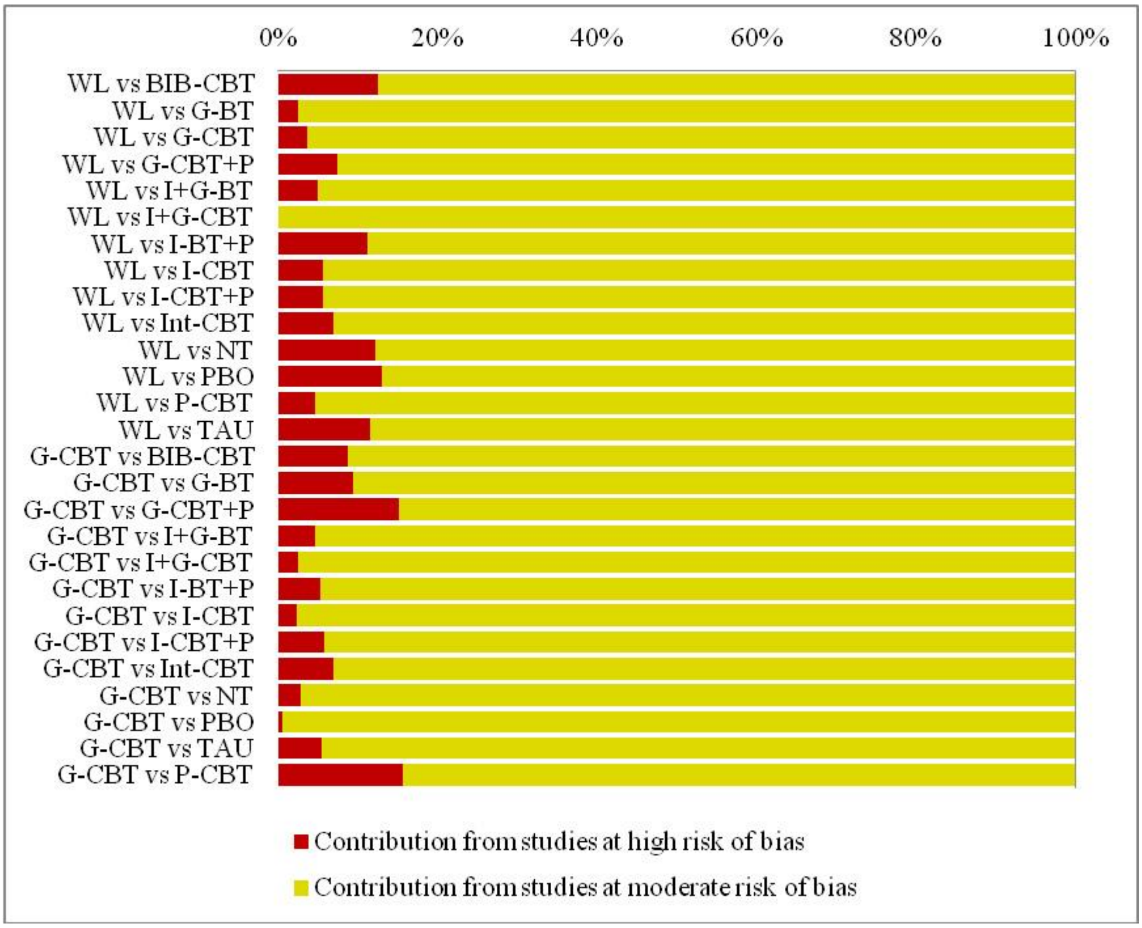

© 2018 Zhou X et al. JAMA Psychiatry.

Contributions summary of studies at risk of bias assessment for mean overall change in symptoms at follow-up

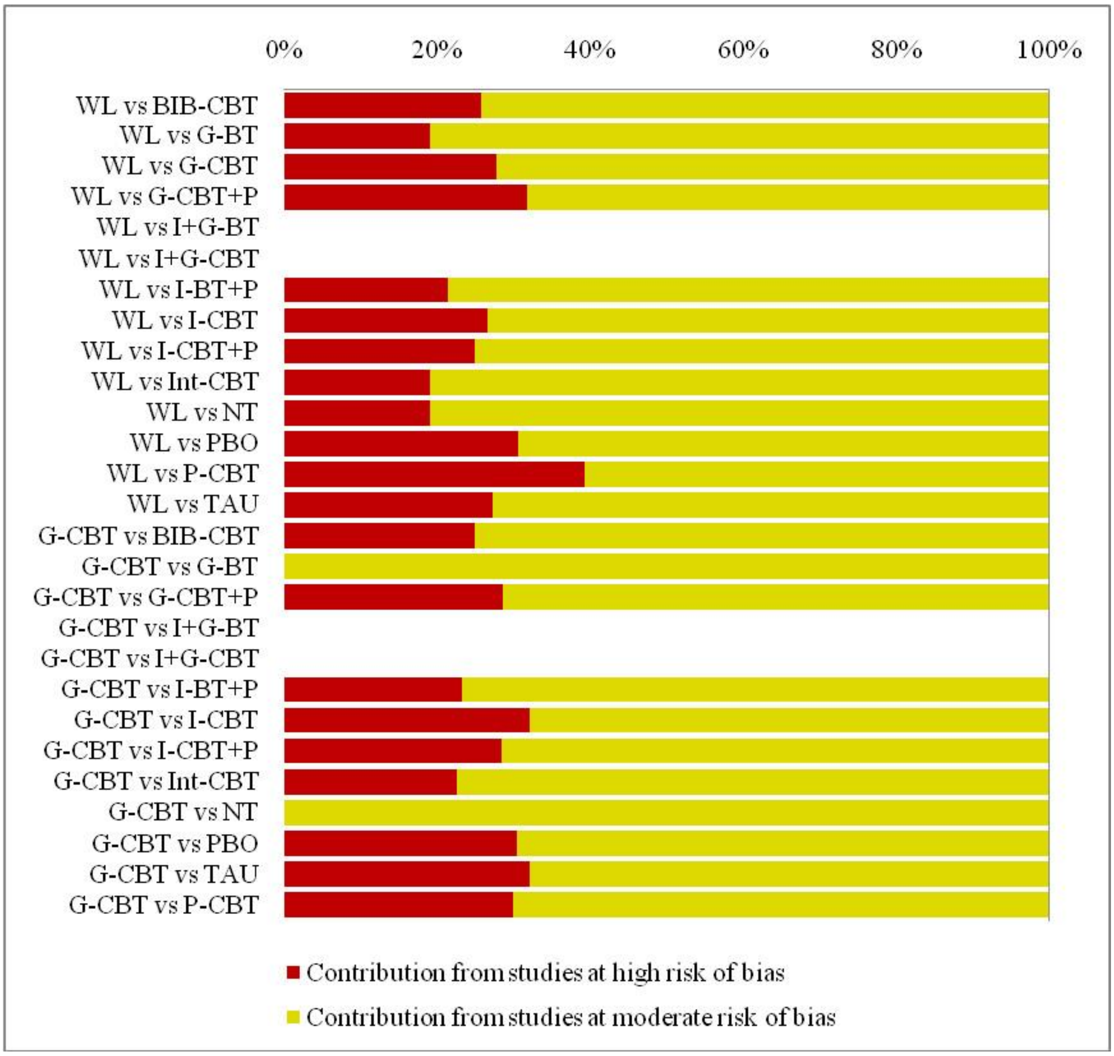

© 2018 Zhou X et al. JAMA Psychiatry.

Contributions summary of studies at risk of bias assessment for all-cause discontinuation

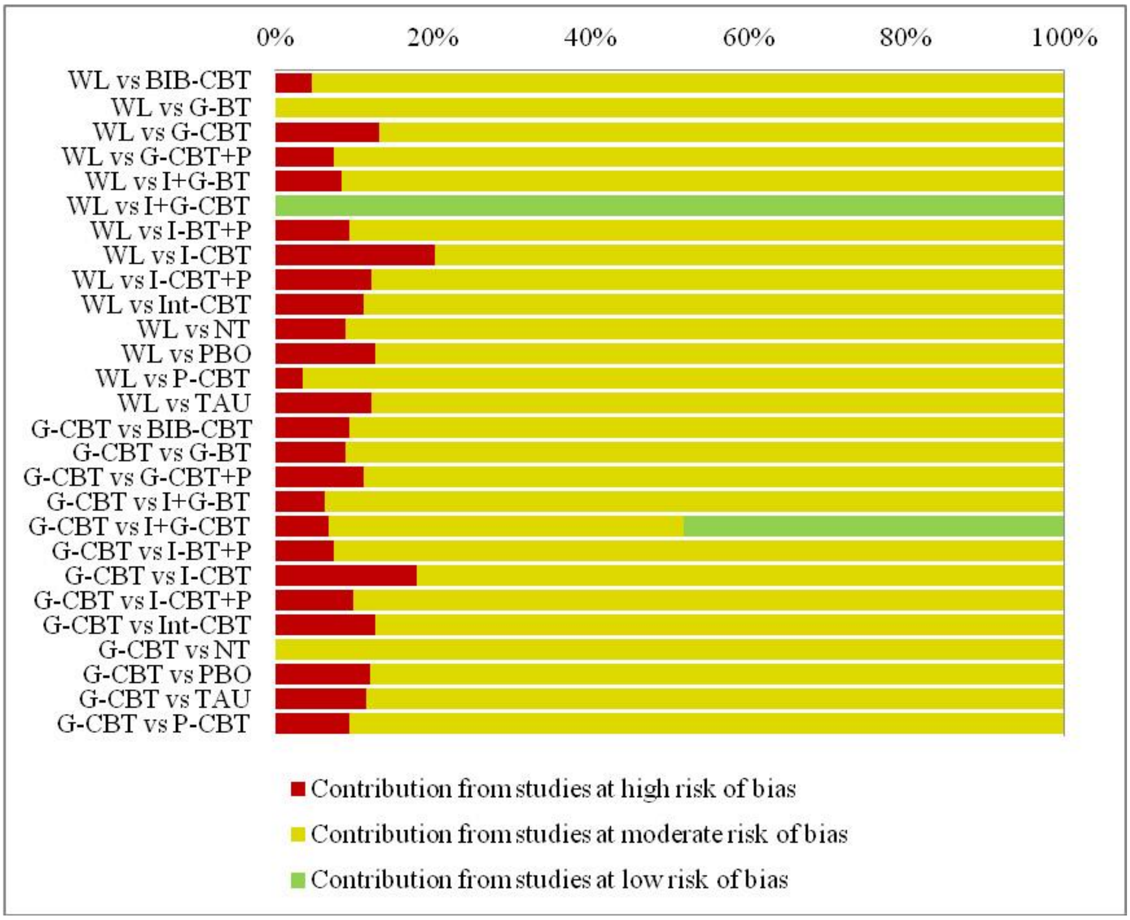

© 2018 Zhou X et al. JAMA Psychiatry.

Contributions summary of studies at risk of bias assessment for mean overall change in quality of life and functional improvement

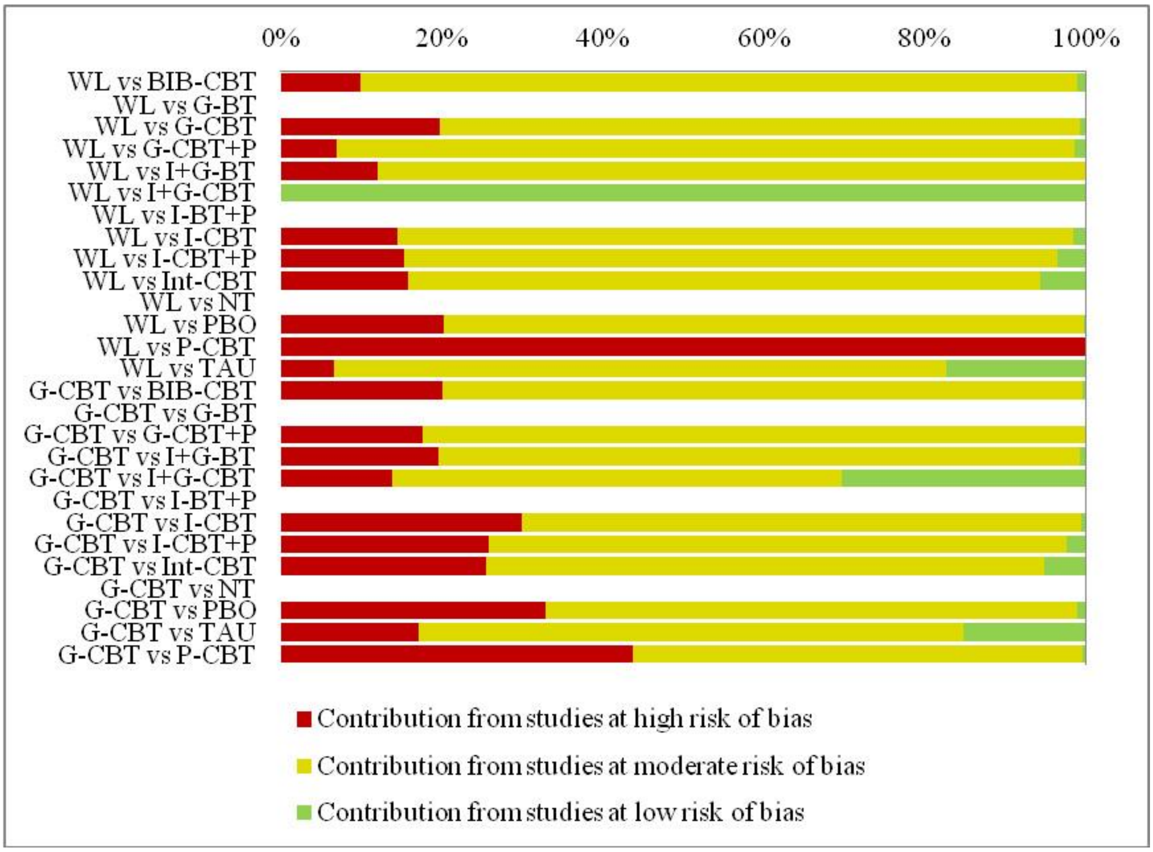

© 2018 Zhou X et al. JAMA Psychiatry.

Contributions summary of studies at risk of bias assessment for mean overall change in anxiety symptoms at short-term follow-up

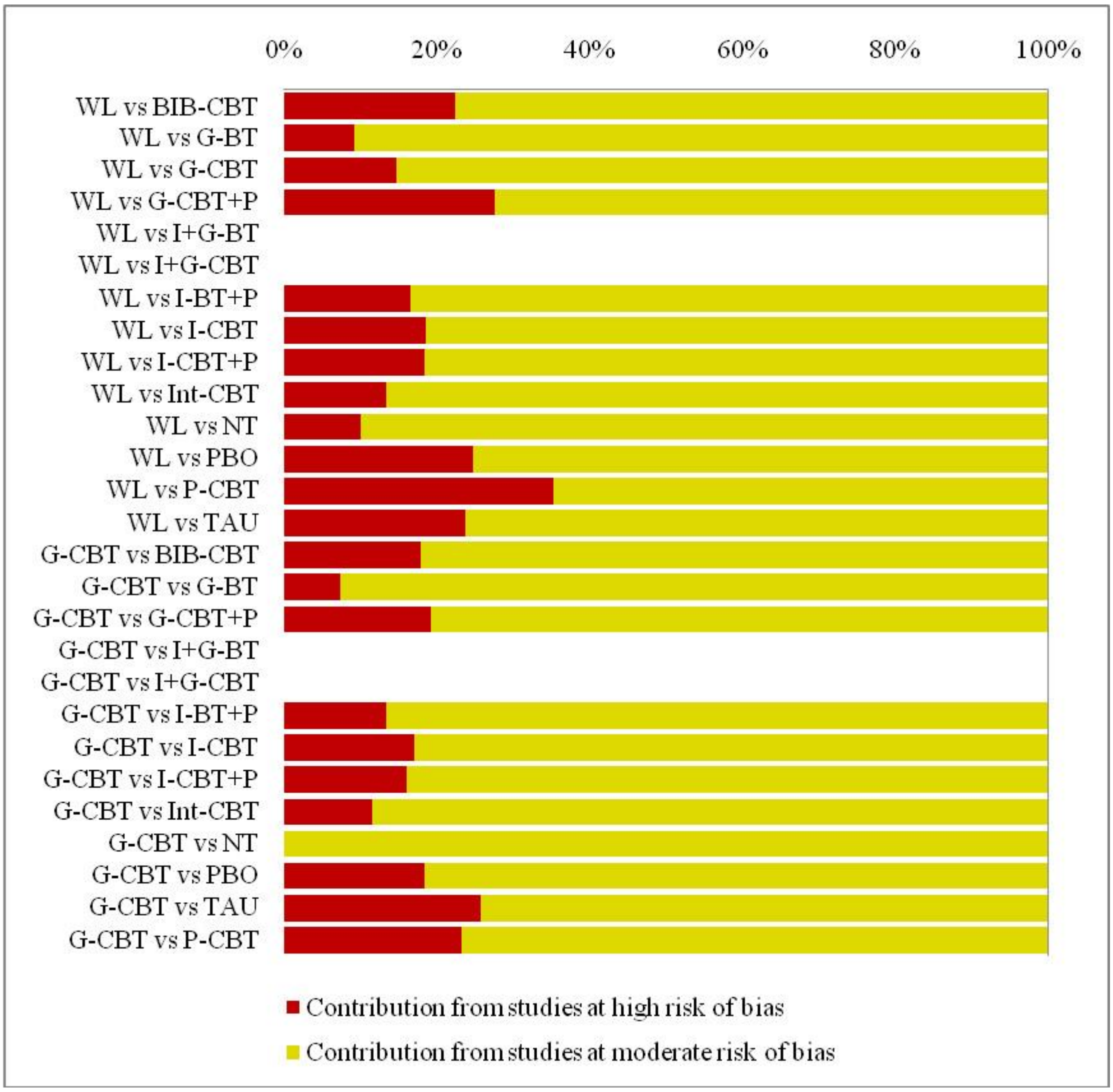

© 2018 Zhou X et al. JAMA Psychiatry.

Contributions summary of studies at risk of bias assessment for mean overall change in anxiety symptoms at long-term follow-up

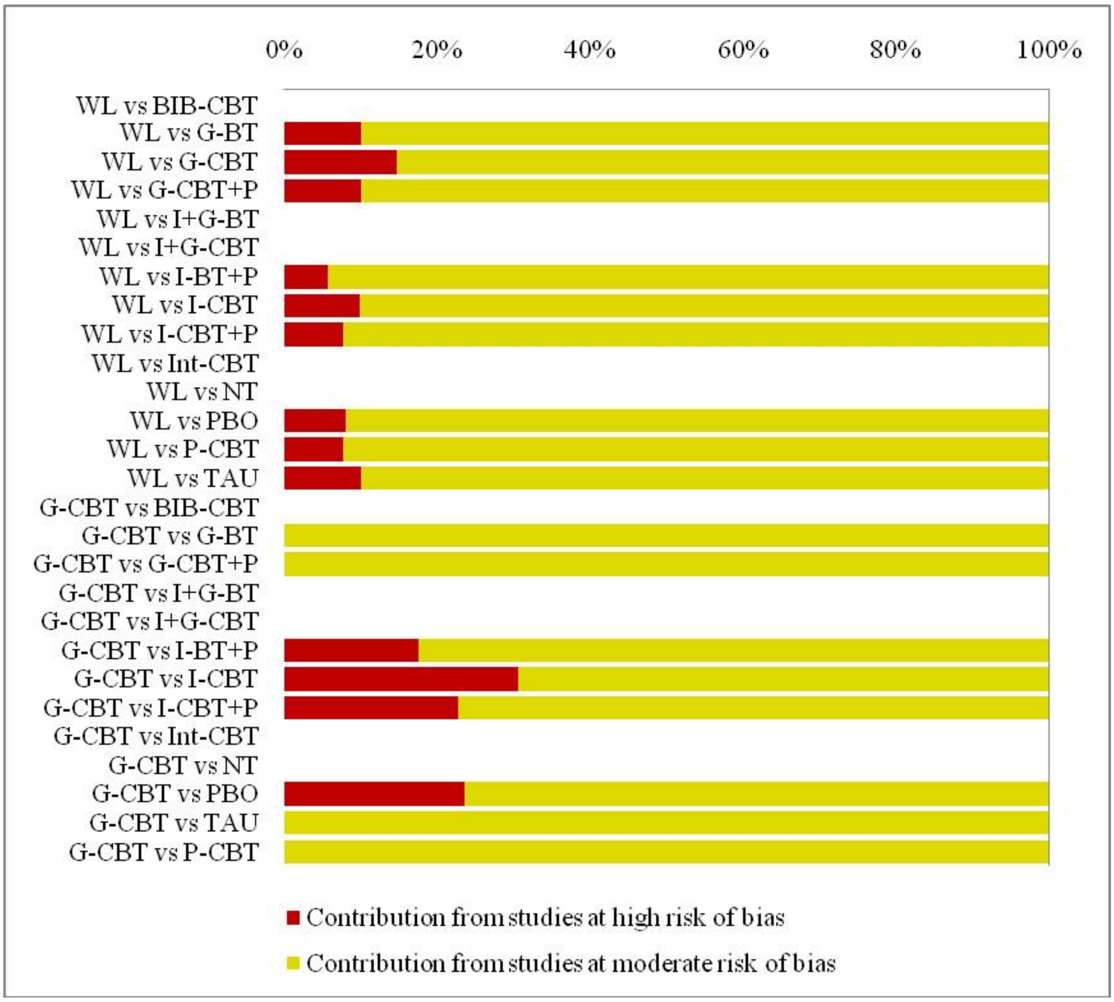

Legend: BIB-CBT=bibliotherapy cognitive-behavioral therapy, G-BT=group behavioral therapy, G-CBT=group cognitive-behavioral therapy, G-CBT+P=group cognitive-behavioral therapy with parental involvement, I-BT+P=individual behavioral therapy with parental involvement, I-CBT=individual cognitive-behavioral therapy, I-CBT+P=individual cognitive-behavioral therapy with parental involvement, I+G-BT=individual and group behavioral therapy, I+G-CBT=individual and group cognitive-behavioral therapy, Int-CBT=internet-assisted cognitive-behavioral therapy, NT=no-treatment, PBO=psychological placebo, P-CBT=parent-only cognitive-behavioral therapy, TAU=treatment as usual, WL=waitlist.

© 2018 Zhou X et al. JAMA Psychiatry.

eTable 1. Clinical and Methodological Characteristics of Included Studies

**Source**	**Type of Anxiety**	**Diagnostic Criteria**	**Treatment Conditions and Sample Size**	**Age, Mean (range), y**	**Proportion of Female, %**	**Recruiting Area (country)**	**Setting**	**Baseline Severity Scale**	**Baseline Severity, Mean (SD)**	**Treatment Duration, wk**	**Sessions, No.**			**Follow-up Duration, mo**	**Effect Sizes for Efficacy at Post-Treatment**
											**No. of Total Sessions**	**No. of Child Sessions**	**No. of Parental Sessions**		
Afshari et al 2014	Sep	ADIS-IV C/P	G-CBT = 24[a]; NT = 10	10.6 (9 to 13)	50	Iran	Outpatient clinic	SCARED-C	52.75 (8.16)	10 or 12	10 or 12	10	0 or 2	3	−1.79
Arendt et al 2016	Sep, GAD, SAD, SP, OCD, PD w/o Agor, Agor w/o Panic	ADIS-IV C/P	G-CBT+*P* = 56; WL = 53	11.78 (7 to 16)	57	Denmark	University clinic	SCAS-C-44	39.17 (16.57)	10	10	10	10	3[b], 12	−0.69

© 2018 Zhou X et al. JAMA Psychiatry.

Azadeh et al 2016	SAD	DSM-V	G-CBT = 15; NT = 15	15.4 (15 to 16)	100	Iran	Not stated	SASA-28	98.58 (6.78)	10	10	10	NA	NA	−0.42
Baer et al 2005	SAD	ADIS-IV C	I+G-BT = 6; WL = 6	15.5 (13 to 18)	58	Canada	Outpatient clinic	SPAI-C	96.47 (22.00)	12	12	12	1	NA	−1.32
Barrett et al 1996	OAD, Sep, SAD	ADIS-III-R C/P	I-CBT = 28; I-CBT+*P* = 25; WL = 26	9.3 (7 to 14)	43	Australia	University clinic	RCMAS-37	12.52 (6.13)	12	12/12	12/12	NA/12	6, 12	0.20/−0.32/−0.54[c]
Barrett et al 1998	OADs, Sep, SAD	ADIS-III-R C/P	G-CBT = 23; G-CBT+*P* = 17; WL = 20	(7 to 14)	47	Australia	University clinic	FSSC-R-80	133.94 (10.68)	12	12/12	12/12	NA/12	12	0.17/−1.70/−2.15[c]

© 2018 Zhou X et al. JAMA Psychiatry.

Barrington et al 2005	PD, Sep, SAD, SP, GAD, Anx-NOS	ADIS-IV C	G-CBT = 28; TAU = 26	10.0 (7 to 14)	65	Australia	Community clinic	RCMAS	15.69 (6.46)	12	12	12	NA	3, 9	−0.01
Beidel et al 2000	SAD	ADIS-IV C	I+G-BT = 36; PBO = 31	10.3 (8 to 12)	57	United States	University clinic	STAIC-trait	32.75 (8.33)	12	24	24	NA	6[b]	0.09
Bergman et al 2013	SM	ADIS-IV P	I-BT+*P* = 12; WL = 9	5.4 (4 to 8)	48	United States	Not stated	SASC-P-18	56.43 (10.36)	24/12	24	20	20	3[b]	−0.98
Bodden et al 2008	GAD, Sep, SAD, PD, SP	ADIS-IV C/P	I-CBT = 64; I-CBT+*P* = 64	12.4 (8 to 17)	59	Netherlands	Community clinic	STAIC	37.63 (8.21)	13	13/13	13/8	NA/10	3	−0.18
Cartwright-Hatton et al 2011	Sep, SAD, SP,	ADIS-IV P	P-CBT = 38; WL = 36	2 · 6.6 (7 to 9)	57	United Kingdom	University clinic	MASC-C	59.43 (10.56)	10	10	NA	10	12[b]	0.31

© 2018 Zhou X et al. JAMA Psychiatry.

	PD, GAD														
Chalfantv 2007	Sep, GAD, SP, SAD, PD	ADIS-IV C/P	G-CBT+*P* = 28; WL = 19	10.8 (8 to 13)	26	Australia	Outpatient clinic	RCMAS-37	16.56 (3.72)	21	12	12	12	NA	−2.74
Chavira et al 2014	SAD, Sep, SP, and GAD	ADIS-IV C/P	I-CBT = 24; BIB-CBT = 24	9.6 (8 to 13)	56	United States	Community clinic or bibliotherapy	SCARED-C-41	26.35 (15.04)	12 to 16	10/10	10/10	NA/NA	3	−0.29
Chiu et al 2013	SAD, Sep, GAD	ADIS-IV C/P	I-CBT+*P* = 22; WL = 18	8.5 (5 to 12)	45	United States	School	MASC-C-39	60.57 (15.60)	10 to 16 /12	10 to 16	10 to 16	Most of the sessions (82.1%)	NA	−0.67
Chu et al 2016	Sep, GAD, SAD	ADIS-IV-TR C/P	G-BT = 21; WL = 14	12.0 (12 to 14)	71	United States	School	SCARED-C-41	29.21 (11.05)	15	12 to 15	12 to 15	NA	4[b]	−0.55
Cobham et al 1998	SAD, OAD, GAD,	ADIS-IV C/P and	G-CBT = 35[a]; G-	9.6 (7 to 14)	49	Australia	University clinic	RCMAS-37	15.28 (7.22)	14	10/14	10/10	NA/4	6, 12	−0.29

© 2018 Zhou X et al. JAMA Psychiatry.

	SP, SAD, Agor	ADIS-III-R C/P	CBT+P = 32[a]												
Cobham et al 2012	GAD, Sep, SAD, SP, Agor, PD	ADIS-IV C/P	I-CBT+P = 23; BIB-CBT = 20; WL = 12	9.9 (7 to 14)	45	Australia	University clinic or bibliotherapy	RCMAS	14.89 (5.85)	12/12	12/12	6/12	6/NA	3, 6	0.39/−0.85/−1.25[c]
Cobham et al 2017	GAD, SAD, Sep, SP, OCD	ADIS-IV C/P	P-CBT = 33; WL = 30	9.3 (7 to 14)	49	Australia	University clinic	SCAS-C	42.23 (13.92)	6	6	NA	6	3[b], 6, 12	−1.00
Conaughton et al 2017	SAD, GAD, SP	ADIS-IV C/P	Int-CBT = 21; WL = 21	9.74 (8 to 12)	14	Australia	Internet	SCAS-C-44	38.79 (13.50)	10 to 14	16	10	6	3[b]	−0.46
Cornwall et al 1996	SP	ADIS-III-R C/P	I-CBT = 12; WL = 12	8.3 (7 to 10)	50	Australia	University clinic	RCMAS	14.93 (6.32)	6	6	6	NA	3[b]	−0.71

© 2018 Zhou X et al. JAMA Psychiatry.

Creswell et al 2015	Sep, GAD, SAD, SP, PD w/o Agor, Agor w/o Panic, SM, Anx-NOS	ADIS-IV-C/P	I-CBT = 71; I-CBT+ *P* = 140[a]	10.2 (7 to 12)	52	United Kingdom	University clinic	SCAS-C-44	40.23 (18.99)	16	16/(16 or 18)	8	NA/(8 or10)	6, 12	−0.35
de Groot et al 2007	GAD, SAD, Sep, SP	ADIS-IV C/P	I-CBT+ *P* = 14; G-CBT+ *P* = 15	8.9 (7 to 12)	34	Australia	University clinic	SCAS-C-45	33.79 (16.87)	12	12/12	6/6	6/6	3[d], 6	−0.19
Donovan et al 2014	SAD, Sep, SP, GAD, SM	ADIS-IV P	Int-CBT = 23; WL = 29	4.1 (3 to 6)	54	Australia	Internet	CSR	5.57 (1.18)	10	6	NA	6	6[d]	−0.96
Ebrahiminejad et al 2016	SAD	SCID	G-CBT =	14.4 (7th to	100	Iran	School	SPIN	25.94 (7.57)	8	8	8	NA	NA	−0.53

© 2018 Zhou X et al. JAMA Psychiatry.

			15; NT = 15	8th grades)											
Esbjørn et al 2015	Sep, SP, GAD, and SAD	ADIS-IV C/P	I-CBT = 26; I-CBT+ *P* = 28	9.6 (7 to 12)	48	Denmark	University clinic	SCARED-R-C-69	42.28 (22.52)	14	14/14	14/8	2/8	6	0.01
Flannery-Schroeder et al 2000	GAD, Sep, SAD	ADIS-IV C/P	I-CBT = 18; G-CBT = 13; WL = 14	(8 to 14)	49	United States	University clinic	RCMAS-48	52.77 (11.58)	18/18/9	18/18	18/18	NA/NA	3, 12	−0.24/−0.73/−0.49[c]
Fujii et al 2013	Sep, SAD, GAD	ADIS-IV C/P	I-CBT+ *P* = 10; TAU = 6	8.8 (7 to 11)	25	United States	University clinic or Community clinic	CSR	5.58 (0.53)	32/16	32	32	32	NA	−2.45
Garcia-Lopez et al 2014	SAD	ADIS-IV C/P	G-CBT = 33; G-CBT+ *P* = 27	15.4 (13 to 18)	65	Spain	School	CSR	5.65 (1.26)	12	12/12	12/12	NA/5	12	−0.36

© 2018 Zhou X et al. JAMA Psychiatry.

Ginsburg et al 2002	GAD, SP, SAD	ADIS-IV C	G-CBT = 6; PBO = 6	15.6 (14 to 17)	83	United States	School	SCARED-C-41	34.43 (19.58)	10	10	10	NA	NA	−0.37
Ginsburg et al 2012	GAD, SAD, Sep, SP, Anx-NOS	ADIS-IV C/P	I-CBT = 17; TAU = 15	10.3 (7 to 17)	63	United States	School	SCARED-C-41	34.64 (11.61)	12	0 to 13 Mean: 7.29	0 to 13 Mean: 7.29	3	1	0.38
Hancock et al 2016	Sep, GAD, SAD, SP and Agor w/o Panic	ADIS-IV C	G-CBT+*P* = 131[a]; WL = 62	11.2 (7 to 17)	58	Australia	Outpatient clinic	MASC-C	61.14 (12.69)	10	10	10	10	3[b]	−0.68
Hayward et al 2000	SAD	ADIS-IV C/P	G-CBT = 12; NT = 23	15.8 (14 to 17)	100	United States	Not stated	CSR	5.13 (1.78)	16	16	16	NA	12[b]	−1.25
Herbert et al 2009	SAD	ADIS-IV C	I-CBT = 24; G-CBT =	14.7 (12 to 17)	56	United States	University clinic	SPAI-C-26	39.68 (16.82)	12	12/12	12/12	NA/NA	6	−0.41/−0.37/0.07[c]

© 2018 Zhou X et al. JAMA Psychiatry.

			23; PBO = 26												
Hirshfeld-Becker et al 2010	Sep, SAD, GAD, Agor, SP	K-SADS	I-CBT+ *P* = 34; WL = 30	5.4 (4 to 7)	53	United States	Outpatient clinic	CBCL-Internalizing	64.70 (7.90)	24	20	13	20	12[b]	−0.50
Holmes et al 2014	GAD	ADIS-IV-TR C/P	G-CBT+ *P* = 20; WL = 22	9.6 (7 to 12)	67	Australia	University clinic	SCAS-C-44	37.99 (14.02)	10/12	10	10	7	3[b]	−0.58
Hudson et al 2009	GAD, PD, Sep, SAD, SP	ADIS-IV C/P	G-CBT+ *P* = 60; PBO = 52	10.2 (7 to 16)	43	Australia	University clinic	SCAS-C-38	35.05 (17.03)	10	10	10	10	3	0.03
Ingul et al 2014	SAD	ADIS-IV C	I-CBT = 25; G-CBT = 33;	14.5 (13 to 16)	56	Norway	Not stated	SCARED-C-41	24.18 (13.44)	12/10/10	12/10	12/10	NA/NA	12	−1.18/−1.03/0.19[c]

© 2018 Zhou X et al. JAMA Psychiatry.

			PBO = 18												
Kendall et al 1994	OAD, Sep, AD	ADIS-III-R C/P	I-CBT = 30; WL = 30	(9 to 13)	40	United States	University clinic	RCMAS-37	53.85 (9.91)	16 to 20/8	16 to 20	16 to 20	NA	12[b]	−1.12
Kendall et al 1997	OAD, Sep, AD	ADIS-III-R C/P	I-CBT = 75; WL = 43	(9 to 13)	38	United States	University clinic	RCMAS-37	55.44 (9.90)	16 to 20/8	16 to 20	16 to 20	NA	12[b]	−0.59
Kendall et al 2008	GAD, Sep, SAD	ADIS-IV C/P	I-CBT = 55; I-CBT+*P* = 56; PBO = 50	10.3 (7 to 14)	44	United States	University clinic	MASC-C-39	47.69 (20.45)	16	16/16	16/16	2/16	12	0.00/−0.26/−0.26[c]
Khanna et al 2010	Sep, SAD, GAD, SP and PD	ADIS-IV P	Int-CBT = 16; ICBT = 17; PBO = 16	10.1 (7 to 13)	33	United States	Community or internet	MASC-C-39	49.19 (13.88)	15	12/12	12/12	2/NA	3	−0.17/−0.43/−0.27[c]

© 2018 Zhou X et al. JAMA Psychiatry.

Last et al 1998	SP or SAD, Sep, AD, OAD, PD	K-SADS	I-CBT = 32; PBO = 24	12.0 (6 to 17)	60	United States	University clinic	STAIC-M	39.22 (11.08)	12	12	12	NA	1[b]	0.35
Lau et al 2010	GAD, Sep, SAD.	K-SADS	G-CBT = 24; WL = 21	8.6 (6 to 11)	47	China	Community clinic	SCAS-C	35.56 (12.60)	12 to 14	9	9	NA	3[b], 6	−0.93
Leong et al 2009	GAD, Sep, SAD, SP, Agor w/o Panic	ADIS-IV C/P	I-CBT+*P* = 15; BIB-CBT = 15	9.3 (7 to 14)	37	Australia	University clinic or bibliotherapy	RCMAS	15.73 (7.35)	12	12/12	6/12	6/NA	3, 6	0.55
Liber et al 2008	Sep, GAD, SAD, SP	ADIS-IV C/P	I-CBT+*P* = 65; G-CBT+*P* = 62	10.0 (8 to 12)	44	Netherlands	University clinic	MASC-C-39	51.13 (18.37)	17	10/10	10/10	4/4	NA	0.03

© 2018 Zhou X et al. JAMA Psychiatry.

Lyneham et al 2006	GAD, Sep, SAD, SP, PD	ADIS-IV C	BIB-CBT = 78[a]; WL = 22	9.4 (6 to 12)	49	Australia	Bibliotherapy	RCMAS	15.34 (6.84)	12	12	12	NA	12[b]	−0.77
Manassis et al 2002	GAD, Sep, SP, SAD, PD	DICA-R	I-CBT+*P* = 43; G-CBT+*P* = 43	10.0 (8 to 12)	46	Canada	Outpatient clinic	MASC-C-39	52.82 (10.19)	12	12/12	12/12	12/12	NA	−0.18
March et al 2009	Sep, GAD, SAD, SP	ADIS-IV C/P	Int-CBT = 40; WL = 33	9.4 (7 to 12)	55	Australia	Internet	SCAS-C-38	39.35 (16.04)	10	10	10	6/NA	6[d]	−0.25
Masia-Warner et al 2005	SAD	ADIS-IV C/P	G-CBT = 21; WL = 21	14.8 (13 to 17)	74	United States	School	CSR	5.40 (1.19)	12	12	12	NA	9[d]	−2.09
Masia-Warner et al 2007	SAD	ADIS-IV C/P	G-CBT = 19; PBO = 17	15.1 (14 to 16)	83	United States	School	CSR	5.10 (0.94)	12	12	12	NA	6[d]	−2.04

© 2018 Zhou X et al. JAMA Psychiatry.

Masia-Warner et al 2011	Sep, SAD, GAD, SP, Anx-NOS	ADIS-IV C/P	I-CBT = 20; WL = 20	12.4 (8 to 16)	65	United States	Outpatient clinic	CSR	5.75 (1.00)	10/8	12	12	3	3[d]	−2.72
Masia-Warner et al 2016	SAD	ADIS-IV C/P	G-CBT = 95[a]; PBO = 43	15.4 (13 to 17)	68	United States	School	CSR	5.57 (0.93)	12	12	12	NA	5[d]	−0.63
McConachie et al 2013	Sep, GAD, SAD, SP, Anx-NOS, Agor w/o Panic	ADIS-IV-TR C	G-CBT+*P* = 17; WL = 15	11.7 (9 to 13)	13	United Kingdom	Community clinic	SCAS-C-38	39.81 (21.03)	7	7	7	7	3, 6	0.10
Melfsen et al 2011	SAD	DIPS-K	I-CBT = 21; WL = 23	10.7 (8 to 14)	48	Germany	Not stated	CSR	5.25 (0.95)	20/16	24	20	4	NA[d]	−1.12

© 2018 Zhou X et al. JAMA Psychiatry.

Mendlowitz et al 1999	DSM-IV anxiety disorders	DICA-R-P	G-CBT = 23; G-CBT+*P* = 18; P-CBT = 21	9.8 (7 to 12)	57	Canada	Outpatient clinic	RCMAS-48	46.69 (9.92)	12	12/12/12	12/12/NA	NA/12/12	NA	0.08/−0.25/−0.33[c]
Monga et al 2015	DSM-IV anxiety disorders	ADIS-IV P	G-CBT+*P* = 45; P-CBT = 32	6.8 (5 to 7)	62	Canada	University clinic	SCARED-P	29.20 (10.63)	12	11/11	11/11	11/11	6, 12	−0.13
Muris et al 2001	Sep, GAD, SAD	DISC 2.3	I-CBT = 17; G-CBT = 19	9.9 (8 to 13)	75	Netherlands	School	STAIC-trait	44.08 (7.61)	6	12/12	12/12	NA/NA	NA	0.43
Muris et al 2002a	Sep, GAD, SAD	DISC 2.3	G-CBT = 10; PBO = 10	10.0 (9 to 12)	65	Netherlands	School	STAIC-trait	43.00 (8.98)	6	12	12	NA	NA	−1.10

© 2018 Zhou X et al. JAMA Psychiatry.

Muris et al 2002b	Sep, GAD, SAD	DISC 2.3	G-CBT = 11; PBO = 13	9.3 (8 to 12)	63	Netherlands	School	SCARED-C-66	75.08 (19.07)	6	6	6	NA	NA	−0.31
Nauta et al 2001	Sep, SAD, GAD	ADIS-IV C/P	I-CBT = 9; I-CBT+*P* = 9	10.2 (8 to 15)	44	Netherlands	Outpatient clinic	Fear Questionnaire	31.10 (15.66)	12	12/12	12/12	2/7	3, 15	0.36
Nauta et al 2003	Sep, SAD, GAD, or Agor w/o Panic	ADIS-IV C/P	I-CBT = 37; I-CBT+*P* = 39	11.0 (7 to 18)	51	Netherlands	University clinic	SCAS-C-44	32.07 (14.43)	12	12/12	12/12	2/7	3, 12	−0.08
Oerbeck et al 2014	SM	ADIS-IV C/P	I-CBT = 12; WL = 12	6.5 (3 to 9)	67	Norway	School	SMQ-32	0.80 (0.35)	12	21	21	3	6[e,b], 12	−1.70
Olivares et al 2005	SAD	ADIS-IV C	G-CBT = 17; WL = 17	15.0 (14 to 17)	59	Spain	School	SPAI-C	129.60 (20.21)	12	12	12	NA	6	−2.97

© 2018 Zhou X et al. JAMA Psychiatry.

Olivares et al 2014	SAD	ADIS-IV C	G-CBT = 78[a]; WL = 39	15.4 (14 to 18)	65	Spain	School	SAS-A	64.50 (5.53)	12	12	12	NA	6[b], 12	−3.83
Ortbandt et al 2009	Sep, GAD, SAD	DIPS-K	I+G-CBT = 10; WL = 9	9.7 (7 to 12)	53	Germany	Outpatient clinic	DISYPS-KJ: FBB-ANG	1.07 (0.34)	10 to 20	10 to 20	10 to 20	5	NA	−1.04
Öst et al 2015	SAD	ADIS-IV C/P	I+G-BT = 32[a]; WL = 23	11.6 (8 to 14)	62	Sweden	Not stated	MASC-C-39	55.07 (18.52)	12	24	24	0,8	12[b]	−0.57
Özyurt et al 2016	Sep, GAD, SAD, SP	K-SADS-PL	P-CBT = 37; WL = 37	9.7 (8 to 12)	21	Turkey	University clinic	SCARED-C-41	32.27 (10.19)	8	8	NA	8	2-4	−1.08
Pina et al 2012	Sep, GAD, SAD, SP	ADIS-IV C/P	I-CBT = 29; I-CBT+*P* = 59	10.4 (NA)	51	United States	University clinic	RCMAS-28	12.47 (9.23)	12	12/12	12/12	NA/12	6	0.33

© 2018 Zhou X et al. JAMA Psychiatry.

Pincus et al 2010	PD w/o Agor	ADIS-IV C/P	I-CBT = 13; PBO = 13	15.8 (14 to 17)	76	United States	University clinic	MASC-C-39	59.52 (20.97)	12/8	11	11	NA	3[b], 6	−0.82
Rapee et al 2006	GAD, Sep, SAD, PD, SP	ADIS-IV C/P	G-CBT+*P* = 90; BIB-CBT = 90; WL = 87	9.5 (6 to 12)	40	Australia	University clinic or bibliotherapy	SCAS–C-38	33.44 (18.56)	12	9/9	9/9	9/NA	3	−0.06/0.02/0.09[c]
Rosa-Alcázar et al 2009	SAD	ADIS-IV C	G-CBT = 20; PBO = 37[a]; WL = 20	14.9 (14 to 17)	75	Spain	School	SPAI-C	123.93 (13.81)	12	12	12	NA	6, 12	−1.42/−3.38/−1.07[c]
Sánchez-García et al 2009	SAD	ADIS-IV C/P	G-CBT = 28; G-BT = 29;	11.9 (10 to 14)	73	Spain	Not stated	SPAI-C	28.34 (6.11)	12	12/12	12/12	NA/NA	6, 12	0.25/−2.20/−2.46[c]

© 2018 Zhou X et al. JAMA Psychiatry.

			WL = 25												
Schneider et al 2011	Sep	DIPS-K	I-CBT+*P* = 21; WL = 22	6.2 (5 to 7)	58	Switzerland	University clinic	RCMAS-37	0.41 (0.21)	16	16	12	12	1[b]	−0.89
Schneider et al 2013	Sep	DIPS-K	I-CBT = 33; I-CBT+*P* = 31	10.4 (8 to 13)	52	Switzerland	University clinic	RCMAS-37	0.42 (0.22)	16	16/16	16/12	1/12	1, 12	0.31
Sciberras et al 2015	GAD, SAD, or Sep	ADIS-IV C	I-CBT+*P* = 6; TAU = 6	11.0 (8 to 12)	8	Australia	Outpatient clinic	SCAS-C	32.95 (17.65)	12	10	10	10	NA	−0.48
Shortt et al 2001	GAD, SAD, or Sep	DISC-AP	G-CBT+*P* = 54; WL = 17	7.9 (6 to 10)	59	Australia	Not stated	RCMAS	12.27 (2.10)	10	10	10	4	12[d]	−4.28

© 2018 Zhou X et al. JAMA Psychiatry.

Silk et al 2013	Sep, GAD, SAD	K-SADS	I-CBT = 30; PBO = 17	10.5 (9 to 13)	51	United States	University clinic	PARS	20.43 (4.58)	16	16	14	2/2	NA	−0.71
Silverman et al 1999a	SP, SAD, Agor	ADIS-III-R C/P	I-BT+*P* = 40; I-CBT+*P* = 41; PBO = 23	(6 to 16)9.8	48	United States	University clinic	RCMAS-37	11.57 (6.45)	10	10/10	10/10	10/10	3, 6, 12	0.24/−0.26/−0.51[c]
Silverman et al 1999b	OAD, GAD, SAD	ADIS-III-R C/P	G-CBT+*P* = 37; WL = 19	10.0 (6 to 16)	39	United States	University clinic	RCMAS	13.75 (7.77)	12/8 to 10	12	12	12	3[b], 6, 12	−0.43
Silverman et al 2009	Sep, SP, GAD, SAD, PD w/o Agor	ADIS-IV C/P	I-CBT = 60; I-CBT+*P* = 59	9.9 (7 to 16)	57	United States	Research clinic	RCMAS-37	13.03 (6.79)	12 to 14	12 to 14/12 to 14	12 to 14/12 to 14	NA/12-14	12	−0.10

© 2018 Zhou X et al. JAMA Psychiatry.

Siqueland et al 2005	GAD, SAD	ADIS-IV C	I-CBT = 6; I-CBT+ *P* = 5	14.9 (12 to 17)	27	United States	University clinic	BAI	21.71 (11.76)	16	16/16	16/16	2/12-13	6	−0.59
Smith et al 2014	Sep, SAD,SP, GAD	ADIS-IV P	P-CBT = 18; WL = 13	9.8 (7 to 13)	39	United States	University clinic	MASC-C-39	56.37 (16.70)	10	10	10	10	3[b]	−0.19
Spence et al 2000	SAD	ADIS-III-R C/P	G-CBT = 19; G-CBT+ *P* = 17; WL = 14	10.7 (7 to 14)	38	Australia	University clinic	RCMAS	14.05 (6.06)	12	12/12	12/12	NA/12	6[d], 12	0.01/−0.80/−0.73[c]
Spence et al 2006	Sep, GAD, SAD, SP	ADIS-IV P	G-CBT+ *P* = 22; Int-CBT = 27; WL = 23	9.9 (7 to 14)	42	Australia	University clinic or internet	RCMAS	52.78 (12.16)	10	10/10	10/10	6/6	6[d], 12	−0.26/−0.72/−0.39[c]

© 2018 Zhou X et al. JAMA Psychiatry.

Spence et al 2011	GAD, Sep, SAD, SP	ADIS-IV C/P	Int-CBT = 44; I-CBT+*P* = 44; WL = 27	14.0 (12 to 18)	59	Australia	University clinic or internet	SCAS-C-38	40.22 (17.37)	12	10/10	10/10	5/5	6[d], 12	−0.17/−0.21/−0.04[c]
Spence et al 2017	SAD	ADIS-5 C/P	Int-CBT = 95; WL = 30	11.28 (8 to 17)	60	Australia	Internet	SPAI-C	28.02 (12.22)	10	15 to 16	10	5-6	6[b]	−0.39
Storch et al 2013	Sep, SAD, GAD	ADIS-IV-TR C/P	I-CBT+*P* = 24; TAU = 21	8.9 (7 to 11)	20	United States	University clinic	MASC-P	60.73 (12.08)	16	16	16	16	3[b]	−0.15
Storch et al 2015a	Sep, GAD, SAD	ADIS-IV C/P	I-CBT+*P* = 16; TAU = 15	12.7 (11 to 16)	19	United States	University clinic	MASC-P-39	63.32 (16.22)	16	16	16	16	1[b]	0.02

© 2018 Zhou X et al. JAMA Psychiatry.

Storch et al 2015b	Sep, GAD, SAD, SP, PD	ADIS-IV-C/P	Int-CBT = 49; TAU = 51	9.8 (7 to 13)	44	United States	Community clinic or internet	MASC-C	54.25 (18.29)	12	12	12	2/NA	1[b]	−0.31
Thirlwall et al 2013	GAD, SAD, Sep, PD/Agor, SP	ADIS-IV C/P	BIB-CBT = 125[a]; WL = 69	(7 to 12)	48	United Kingdom	Community clinic or bibliotherapy	SCAS-C-38	38.35 (18.11)	8	8	8	8/NA	6[b]	0.12
Tillfors et al 2011	SAD	SCID	Int-CBT = 10; WL = 9	16.5 (15 to 21)[f]	89	Sweden	Internet	BAI	19.16 (8.47)	9	9	9	NA	12[b]	−0.92
Treadwell et al 1996	OAD, Sep, AD	ADIS-III-R C	I-CBT = 35; WL = 36	11.7 (8 to 13)	40	United States	University clinic	NASSQ	27.75 (7.95)	16	16	16	NA	NA	−0.61
Vigerland et al 2016	Sep, GAD, PD, SAD, SP	ADIS-IV C/P	Int-CBT = 46; WL = 47	10.1 (8 to 12)	55	Sweden	Internet	SCAS-C-44	35.14 (13.45)	10	4 to 11	4	7/NA	3[b]	0.03

© 2018 Zhou X et al. JAMA Psychiatry.

Waters et al 2009	GAD, Sep, SAD, SP	ADIS-IV C/P	G-CBT+ *P* = 31; P-CBT = 38; WL = 11	6.8 (4 to 8)	53	Australia	University clinic	CSR	6.76 (1.13)	10	10/10	10/NA	10/10	6[d], 12	−0.22/−1.49/−1.18[c]
Wergeland et al 2014	Sep, SAD, GAD	ADIS-IV C/P	I-CBT+ *P* = 77; G-CBT+ *P* = 67; WL = 38	11.5 (8 to 15)	53	Norway	Community clinic	SCAS-C-38	36.09 (16.72)	Mean = 13.5/Mean = 10.7/Mean=10	10/10	10/10	4/4	12[d]	0.01/−0.03/−0.04[c]
White et al 2013	Sep, GAD, SAD, SP,	ADIS-IV C/P	I+G-CBT = 15; WL = 15	14.6 (12 to 17)	23	United States	University clinic	PARS	14.67 (5.29)	14	12 to 20	12 to 20	NA	NA	−0.35
Whiteside et al 2015	Sep, GAD, SAD	ADIS-IV C	I-BT+*P* = 7; I-	10.2 (7 to 14)	71	United States	Outpatient clinic	SCAS-C	41.45 (12.42)	6	6/6	6/6	6/NA	3[b]	−0.97

© 2018 Zhou X et al. JAMA Psychiatry.

			CBT = 7												
Wood et al 2006	Sep, SAD, GAD, SP	ADIS-IV C/P	I-CBT = 20; I-CBT+*P* = 20	9.8 (6 to 13)	40	United States	University clinic	MASC-C-39	52.12 (14.87)	12 to 16	12 to 16/12 to 16	12 to 16/12 to 16	1/12-16	12	0.00
Wood et al 2009	Sep, SAD, GAD	ADIS-IV C/P	I-CBT+*P* = 17; WL = 23	9.2 (7 to 11)	33	United States	University clinic	MASC-C-39	55.53 (16.63)	16/12	16	16	16/NA	3[b]	−0.10
Wood et al 2015	SAD, Sep, GAD	ADIS-IV C/P	I-CBT+*P* = 19; WL = 14	12.3 (11 to 15)	30	United States	University clinic	MASC-P-39	61.39 (15.17)	16	16	16	16/NA	1[b]	−0.46
Wuthrich et al 2012	Anx-NOS, GAD, PD, Sep, SAD, SP	ADIS-IV C/P	Int-CBT = 24; WL = 19	15.2 (14 to 17)	63	Australia	Internet	SCAS-C-38	36.19 (17.58)	12	8	8	NA	3[b]	−0.54

© 2018 Zhou X et al. JAMA Psychiatry.

Abbreviations: AD, avoidant disorder; ADIS, The Anxiety Disorders Interview Schedule; Agor, agoraphobia; Agor w/o Panic, agoraphobia with or without panic; Anx-NOS, anxiety not otherwise specified; BIB-CBT, bibliotherapy cognitive, behavioral therapy; DICA, Diagnostic Interview for Children and Adolescents; DISC, Diagnostic Interview Schedule for Children; DIPS-K, German version of the Anxiety Disorders Interview Schedule (ADIS) for Children; DSM, Diagnostic and Statistical Manual of Mental Disorders; GAD, generalized anxiety disorder; G-BT, group behavioral therapy; G-CBT, group cognitive, behavioral therapy; G-CBT+P, froup cognitive, behavioral therapy with parental involvement; I-BT+P, individual behavioral therapy with parental involvement; I-CBT, individual cognitive, behavioral therapy; I-CBT+P, individual cognitive, behavioral therapy with parental involvement; I+G-BT, individual and group behavioral therapy; I+G-CBT, individual and group cognitive, behavioral therapy; Int-CBT, internet, assisted cognitive, behavioral therapy; K-SADS, Kiddie, Schedule for Affective Disorders and Schizophrenia; NT, no treatment; OAD, over-anxious disorder; PBO, psychological placebo; P-CBT, parent, only cognitive, behavioral therapy; PD, panic disorder; PD w/o Agor, panic disorder with or without agoraphobia; SAD, social anxiety disorder/social phobia; SCID, short version of the Structured Clinical Interview for DSM, IV; Sep, separation anxiety disorder; SD, standard deviation; SM, selective mutism; SP, specific/simple phobia; TAU, treatment as usual; WL, wait list.

[a]Combined group.

[b]The authors stated that only one patient was 21 y old.

[c]There were booster sessions in the follow-up period.

[d]Data from authors or other sources.

[e]In the follow-up period, only one group of data are available.

[f]The three effect sizes were those with intervention of 1st vs 2nd, 1st vs 3rd, and 2nd vs 3rd, respectively.

© 2018 Zhou X et al. JAMA Psychiatry.

eTable 2. Evaluation of the Quality of Evidence Using GRADE Framework for Primary Outcomes

The confidence in SMD for mean overall change in symptoms at post-treatment compared with PBO by GRADE system*

Comparison	Study limitations[a]	Imprecision[b]	Heterogeneity and Inconsistency[c]	Indirectness[d]	Publication bias[e]	Confidence in SMD for overall change in anxiety symptoms
BIB-CBT vs. PBO	16.5% of the estimate from studies at high risk, and 83.7% at moderate risk.	SMD –0.03, 95% CrI -0.68 to 0.61	No head-to-head study and no heterogeneity. Only indirect comparison, and no node-splitting inconsistency.	The treatment effects were not significantly influenced by clinical modifiers in the subgroup analyses.	Undetectable by the routine method. The comparison-adjusted funnel plot for the network is not suggestive of any dominant publication bias.	Low (Downgrade by two levels due to study limitations, and imprecision)
G-BT vs. PBO	7.4% of the estimate from studies at high risk, and 92.7% at moderate risk.	SMD -0.77, 95% CrI -1.76 to 0.22	No head-to-head study and no heterogeneity. Only indirect comparison, and no node-splitting inconsistency.	The treatment effects were not significantly influenced by clinical modifiers in the subgroup analyses.	Undetectable by the routine method. The comparison-adjusted funnel plot for the network is not suggestive of any dominant publication bias.	Low (Downgrade by two levels due to study limitations, and imprecision)
G-CBT vs. PBO	9.9% of the estimate from studies at high risk, and 90.0% at moderate risk.	SMD -0.76, 95% CrI -1.16 to -0.36	High heterogeneity according to I^2 (76.5%) and P-value (0.0001) in direct comparisons. No inconsistency between the direct and indirect estimate (Node-split p=0.7012 and tau= 0.5785).	The treatment effects were not significantly influenced by clinical modifiers in the subgroup analyses.	Undetectable by the routine method. The comparison-adjusted funnel plot for the network is not suggestive of any dominant publication bias.	Low (Downgrade by two levels due to study limitations, and heterogeneity)
G-CBT+P	13.5% of the estimate	SMD -0.33, 95%	Only one head-to-head study,	The treatment effects	Undetectable by the routine	Very low (Downgrade

© 2018 Zhou X et al. JAMA Psychiatry.

vs. PBO	from studies at high risk, and 86.7% at moderate risk.	CrI -0.78 to 0.13	and no heterogeneity.<br>No inconsistency between the direct and indirect estimate (Node-split p= 0.5426 and tau= 0.5758).	were not significantly influenced by clinical modifiers in the subgroup analyses. Only one monocenter study.	method. The comparison-adjusted funnel plot for the network is not suggestive of any dominant publication bias.	by three levels due to study limitations, imprecision, and indirectness)
I-BT+P vs. PBO	6.4% of the estimate from studies at high risk, and 93.7% at moderate risk.	SMD -0.42, 95% CrI -1.29 to 0.44	Only one head-to-head study, and no heterogeneity.<br>No inconsistency between the direct and indirect estimate (Node-split p= 0.7679 and tau= 0.5788).	The treatment effects were not significantly influenced by clinical modifiers in the subgroup analyses. Only one monocenter study.	Undetectable by the routine method. The comparison-adjusted funnel plot for the network is not suggestive of any dominant publication bias.	Very low (Downgrade by three levels due to study limitations, imprecision, and indirectness)
I-CBT vs. PBO	12.1 of the estimate from studies at high risk, and 87.9% at moderate risk.	SMD -0.32, 95% CrI -0.72 to 0.07	Moderate heterogeneity according to I^2 (47.6%) and P-value (0.0753) in direct comparisons.<br>No inconsistency between the direct and indirect estimate (Node-split p= 0.5891 and tau= 0.5782).	The treatment effects were not significantly influenced by clinical modifiers in the subgroup analyses.	Undetectable by the routine method. The comparison-adjusted funnel plot for the network is not suggestive of any dominant publication bias.	Very low (Downgrade by three levels due to study limitations, imprecision, and heterogeneity)
I-CBT+P vs. PBO	11.6% of the estimate from studies at high risk, and 88.6% at moderate risk.	SMD -0.18, 95% CrI -0.61 to 0.25	Mild heterogeneity according to I^2 (0%) and P-value (0.5002) in direct comparisons.<br>No inconsistency between the direct and indirect estimate	The treatment effects were not significantly influenced by clinical modifiers in the subgroup analyses.	Undetectable by the routine method. The comparison-adjusted funnel plot for the network is not suggestive of any dominant publication bias.	Low (Downgrade by two levels due to study limitations, and imprecision)

© 2018 Zhou X et al. JAMA Psychiatry.

			(Node-split p=0.6177 and tau= 0.5784).			
I+G-BT vs. PBO	7.6% of the estimate from studies at high risk, and 92.3% at moderate risk.	SMD -0.06, 95% CrI -0.94 to 0.82	Only one head-to-head study, and no heterogeneity.<br>No inconsistency between the direct and indirect estimate (Node-split p= 0.7595 and tau= 0.5790).	The treatment effects were not significantly influenced by clinical modifiers in the subgroup analyses. Only one monocenter study.	Undetectable by the routine method. The comparison-adjusted funnel plot for the network is not suggestive of any dominant publication bias.	Very Low (Downgrade by three levels due to study limitations, study imprecision, and indirectness)
I+G-CBT vs. PBO	9.3% of the estimate from studies at high risk, and 90.5% at moderate risk.	SMD 0.03, 95% CrI -1.10 to 1.16	No head-to-head study and no heterogeneity.<br>Only indirect comparison, and no node-splitting inconsistency.	The treatment effects were not significantly influenced by clinical modifiers in the subgroup analyses.	Undetectable by the routine method. The comparison-adjusted funnel plot for the network is not suggestive of any dominant publication bias.	Low (Downgrade by two levels due to study limitations, and imprecision)
Int-CBT vs. PBO	17.1% of the estimate from studies at high risk, and 83.1% at moderate risk.	SMD 0.06, 95% CrI -0.48 to 0.60	Only one head-to-head study, and no heterogeneity.<br>No inconsistency between the direct and indirect estimate (Node-split p= 0.4719 and tau= 0.5773).	The treatment effects were not significantly influenced by clinical modifiers in the subgroup analyses. Only one monocenter study.	Undetectable by the routine method. The comparison-adjusted funnel plot for the network is not suggestive of any dominant publication bias.	Very low (Downgrade by three levels due to study limitations, imprecision, and indirectness)
NT vs. PBO	7.1% of the estimate from studies at high risk, and 93.0% at moderate risk.	SMD 0.18, 95% CrI -0.66 to 1.03	No head-to-head study and no heterogeneity.<br>Only indirect comparison, and no node-splitting inconsistency.	The treatment effects were not significantly influenced by clinical modifiers in the subgroup analyses.	Undetectable by the routine method. The comparison-adjusted funnel plot for the network is not suggestive of any dominant publication bias.	Low (Downgrade by two levels due to study limitations, and imprecision)

© 2018 Zhou X et al. JAMA Psychiatry.

P-CBT vs. PBO	9.8% of the estimate from studies at high risk, and 90.2% at moderate risk.	SMD -0.04, 95% CrI -0.67 to 0.60	No head-to-head study and no heterogeneity.<br>Only indirect comparison, and no node-splitting inconsistency.	The treatment effects were not significantly influenced by clinical modifiers in the subgroup analyses.	Undetectable by the routine method. The comparison-adjusted funnel plot for the network is not suggestive of any dominant publication bias.	Low (Downgrade by two levels due to study limitations, and imprecision)
TAU vs. PBO	16.1% of the estimate from studies at high risk, and 83.9% at moderate risk.	SMD 0.08, 95% CrI -0.58 to 0.74	No head-to-head study and no heterogeneity.<br>Only indirect comparison, and no node-splitting inconsistency.	The treatment effects were not significantly influenced by clinical modifiers in the subgroup analyses.	Undetectable by the routine method. The comparison-adjusted funnel plot for the network is not suggestive of any dominant publication bias.	Low (Downgrade by two levels due to study limitations, and imprecision)
WL vs. PBO	13.6% of the estimate from studies at high risk, and 86.1% at moderate risk.	SMD 0.67, 95% CrI 0.27 to 1.07	Only one head-to-head study, and no heterogeneity.<br>No inconsistency between the direct and indirect estimate (Node-split p= 0.5305 and tau= 0.5781).	The treatment effects were not significantly influenced by clinical modifiers in the subgroup analyses. Only one monocenter study.	Undetectable by the routine method. The comparison-adjusted funnel plot for the network is not suggestive of any dominant publication bias.	Low (Downgrade by two levels due to study limitations, and indirectness)
Ranking of treatment	8.7% from studies at high risk, and 91.4% at moderate risk.	SUCRA plots suggested the imprecision in a ranking of treatments.	High heterogeneity for mean overall change in symptoms in network meta-analyses according to global I^2 (98.89%).<br>No significant inconsistency in test of global inconsistency (P = 0.6031), and few	The overall effects were not significantly influenced by clinical modifiers in the subgroup analyses.	The comparison-adjusted funnel plot suggested no dominant publication bias.	Very low (Downgrade by three levels due to study limitations, imprecision, and heterogeneity)

© 2018 Zhou X et al. JAMA Psychiatry.

			inconsistency (11.5%) in local inconsistency.			

* Salanti G, Del Giovane C, Chaimani A, Caldwell DM, Higgins JP. Evaluating the quality of evidence from a network meta-analysis. PLoS One. 2014 Jul 3;9(7):e99682. [a] The consideration for study limitations was based on the relative contributions of risk of bias in direct evidence. We downgraded by one level for moderate risk, and two levels for high risk of bias. [b] The consideration for imprecision was based on the width of the confidence interval. [c] The consideration for heterogeneity and inconsistency was based on the corresponding p value. We downgraded by one level for significant heterogeneity and inconsistency, respectively. [d] The consideration for indirectness was based on the results of subgroup analysis. We downgraded by one level if the confidence interval between the two subroups is not overlap. In some cases, there are both statistically significant differences for the nonoverlapping two subgroups, we didn't downgrade these cases. [e] The consideration for publication bias was based on the comparison-adjusted funnel plot and Egger's test. Abbreviation: BIB-CBT=bibliotherapy cognitive-behavioral therapy, CrI=credibility interval, G-BT=group behavioral therapy, G-CBT=group cognitive-behavioral therapy, G-CBT+P=group cognitive-behavioral therapy with parental involvement, I-BT+P=individual behavioral therapy with parental involvement, I-CBT=individual cognitive-behavioral therapy, I-CBT+P=individual cognitive-behavioral therapy with parental involvement, I+G-BT=individual and group behavioral therapy, I+G-CBT=individual and group cognitive-behavioral therapy, Int-CBT=internet-assisted cognitive-behavioral therapy, NT=no-treatment, PBO=psychological placebo, P-CBT=parent-only cognitive-behavioral therapy, SMD=standardized mean difference, TAU=treatment as usual, WL=waitlist.

© 2018 Zhou X et al. JAMA Psychiatry.

The confidence in SMD for mean overall change in symptoms at follow-up compared with PBO by GRADE system*

Comparison	Study limitations[a]	Imprecision[b]	Heterogeneity and Inconsistency[c]	Indirectness[d]	Publication bias[e]	Confidence in SMD for mean overall change in symptoms at follow-up
BIB-CBT vs. PBO	30.9% of the estimate from studies at high risk, and 69.0% at moderate risk.	SMD -0.35, 95% CrI -1.26 to 0.56	No head-to-head study and no heterogeneity.<br>Only indirect comparison, and no node-splitting inconsistency.	The treatment effects were not significantly influenced by clinical modifiers in the subgroup analyses.	Undetectable by the routine method. The comparison-adjusted funnel plot for the network is not suggestive of any dominant publication bias.	Low (Downgrade by two levels due to study limitations, and imprecision)
G-BT vs. PBO	21.8% of the estimate from studies at high risk, and 78.2% at moderate risk.	SMD -0.04, 95% CrI -1.58 to 1.50	No head-to-head study and no heterogeneity.<br>Only indirect comparison, and no node-splitting inconsistency.	The treatment effects were not significantly influenced by clinical modifiers in the subgroup analyses.	Undetectable by the routine method. The comparison-adjusted funnel plot for the network is not suggestive of any dominant publication bias.	Low (Downgrade by two levels due to study limitations, and imprecision)
G-CBT vs. PBO	30.4% of the estimate from studies at high risk, and 69.5% at moderate risk.	SMD -0.36, 95% CrI -1.05 to 0.33	High heterogeneity according to I^2 (90.4%) and P-value (0.0012) in direct comparisons.<br>There is an inconsistency between direct and indirect estimate (Node-split p= 0.0241 and tau= 0.4584).	The treatment effects were not significantly influenced by clinical modifiers in the subgroup analyses.	Undetectable by the routine method. The comparison-adjusted funnel plot for the network is not suggestive of any dominant publication bias.	Very low (Downgrade by four levels due to study limitations, imprecision, heterogeneity and inconsistency)

© 2018 Zhou X et al. JAMA Psychiatry.

G-CBT+P vs. PBO	38.9% of the estimate from studies at high risk, and 61.2% at moderate risk.	SMD -0.34, 95% CrI -1.10 to 0.41	Only one head-to-head study, and no heterogeneity.<br>No inconsistency between direct and indirect estimate (Node-split p= 0.6995 and tau= 0.5446).	The treatment effects were not significantly influenced by clinical modifiers in the subgroup analyses. Only one monocenter study.	Undetectable by the routine method. The comparison-adjusted funnel plot for the network is not suggestive of any dominant publication bias.	Very low (Downgrade by three levels due to study limitations, imprecision, and indirectness)
I-BT+P vs. PBO	9.0% of the estimate from studies at high risk, and 91.0% at moderate risk.	SMD -0.48, 95% CrI -1.73 to 0.76	Only one head-to-head study, and no heterogeneity.<br>No inconsistency between direct and indirect estimate (Node-split p= 0.8266 and tau= 0.5460).	The treatment effects were not significantly influenced by clinical modifiers in the subgroup analyses. Only one monocenter study.	Undetectable by the routine method. The comparison-adjusted funnel plot for the network is not suggestive of any dominant publication bias.	Very low (Downgrade by three levels due to study limitations, imprecision, and indirectness)
I-CBT vs. PBO	15.6% of the estimate from studies at high risk, and 84.6% at moderate risk.	SMD -0.33, 95% CrI -0.97 to 0.32	Mild heterogeneity according to I^2 (0.00%) and P-value (0.8425) in direct comparisons.<br>No inconsistency between direct and indirect estimate (Node-split p= 0.4502 and tau= 0.5386).	The treatment effects were not significantly influenced by clinical modifiers in the subgroup analyses.	Undetectable by the routine method. The comparison-adjusted funnel plot for the network is not suggestive of any dominant publication bias.	Low (Downgrade by two levels due to study limitations, and imprecision)
I-CBT+P vs. PBO	16.2% of the estimate from studies at high risk, and 83.9% at moderate risk.	SMD -0.31, 95% CrI -0.99 to 0.36	Mild heterogeneity according to I^2 (0.00%) and P-value (0.4542) in direct comparisons.<br>No inconsistency between direct and indirect estimate (Node-	The treatment effects were not significantly influenced by clinical modifiers in the subgroup analyses.	Undetectable by the routine method. The comparison-adjusted funnel plot for the network is not suggestive of any dominant publication	Low (Downgrade by two levels due to study limitations, and imprecision)

© 2018 Zhou X et al. JAMA Psychiatry.

			split p= 0.3737 and tau= 0.5319).		bias.	
Int-CBT vs. PBO	10.0% of the estimate from studies at high risk, and 90.0% at moderate risk.	SMD -0.51, 95% CrI -2.12 to 1.10	No head-to-head study and no heterogeneity.<br>Only indirect comparison, and no node-splitting inconsistency.	The treatment effects were not significantly influenced by clinical modifiers in the subgroup analyses.	Undetectable by the routine method. The comparison-adjusted funnel plot for the network is not suggestive of any dominant publication bias.	Low (Downgrade by two levels due to study limitations, and imprecision)
NT vs. PBO	21.8% of the estimate from studies at high risk, and 78.2% at moderate risk.	SMD 2.29, 95% CrI 0.58 to 3.99	No head-to-head study and no heterogeneity.<br>Only indirect comparison, and no node-splitting inconsistency.	The treatment effects were not significantly influenced by clinical modifiers in the subgroup analyses.	Undetectable by the routine method. The comparison-adjusted funnel plot for the network is not suggestive of any dominant publication bias.	Moderate (Downgrade by one level due to study limitations)
P-CBT vs. PBO	37.2% of the estimate from studies at high risk, and 62.8% at moderate risk.	SMD -0.51, 95% CrI -1.71 to 0.70	No head-to-head study and no heterogeneity.<br>Only indirect comparison, and no node-splitting inconsistency.	The treatment effects were not significantly influenced by clinical modifiers in the subgroup analyses.	Undetectable by the routine method. The comparison-adjusted funnel plot for the network is not suggestive of any dominant publication bias.	Low (Downgrade by two levels due to study limitations, and imprecision)
TAU vs. PBO	30.1% of the estimate from studies at high risk, and 69.9% at moderate risk.	SMD -0.40, 95% CrI -1.58 to 0.76	No head-to-head study and no heterogeneity.<br>Only indirect comparison, and no node-splitting inconsistency.	The treatment effects were not significantly influenced by clinical modifiers in the	Undetectable by the routine method. The comparison-adjusted funnel plot for the network is not suggestive of	Low (Downgrade by two levels due to study limitations, and imprecision)

© 2018 Zhou X et al. JAMA Psychiatry.

				subgroup analyses.	any dominant publication bias.	
WL vs. PBO	30.5% of the estimate from studies at high risk, and 69.6% at moderate risk.	SMD 1.33, 95% CrI 0.50 to 2.15	Only one head-to-head study, and no heterogeneity.<br>No inconsistency between direct and indirect estimate (Node-split p= 0.6764 and tau= 0.5409).	The treatment effect was significantly influenced by clinical modifiers (e.g., sex ratio) in the subgroup analyses. Only one monocenter study.	Undetectable by the routine method. The comparison-adjusted funnel plot for the network is not suggestive of any dominant publication bias.	Low (Downgrade by two levels due to study limitations, and indirectness)
Ranking of treatment	23.3% from studies at high risk, and 76.5% at moderate risk.	SUCRA plots suggested the imprecision in a ranking of treatments.	High heterogeneity for discontinuation due to adverse events in network meta-analyses according to global I^2 (97.99%). Significant inconsistency in test of global inconsistency (P < 0.0001), and 37.5% inconsistency in local inconsistency.	The overall effects were significantly influenced by several clinical modifiers in the subgroup analyses.	The comparison-adjusted funnel plot suggested no dominant publication bias.	Very low (Downgrade by five levels due to study limitations, imprecision, heterogeneity, inconsistency, and indirectness)

* Salanti G, Del Giovane C, Chaimani A, Caldwell DM, Higgins JP. Evaluating the quality of evidence from a network meta-analysis. PLoS One. 2014 Jul 3;9(7):e99682. [a] The consideration for study limitations was based on the relative contributions of risk of bias in direct evidence. We downgraded by one level for moderate risk, and two levels for high risk of bias. [b] The consideration for imprecision was based on the width of the confidence interval. [c] The consideration for heterogeneity and inconsistency was based on the corresponding p value. We downgraded by one level for significant heterogeneity and inconsistency, respectively. [d] The consideration for indirectness was based on the results of subgroup analysis. We downgraded by one level if the confidence interval between the two subroups is not overlap. In some cases, there are both statistically significant differences for the nonoverlapping two subgroups, we didn't downgrade these cases. [e] The consideration for publication bias was based on the comparison-adjusted funnel plot and Egger’s test. Abbreviation: BIB-CBT=bibliotherapy

© 2018 Zhou X et al. JAMA Psychiatry.

cognitive-behavioral therapy, CrI=credibility interval, G-BT=group behavioral therapy, G-CBT=group cognitive-behavioral therapy, G-CBT+P=group cognitive-behavioral therapy with parental involvement, I-BT+P=individual behavioral therapy with parental involvement, I-CBT=individual cognitive-behavioral therapy, I-CBT+P=individual cognitive-behavioral therapy with parental involvement, Int-CBT=internet-assisted cognitive-behavioral therapy, NT=no-treatment, OR=odds ratio, PBO=psychological placebo, P-CBT=parent-only cognitive-behavioral therapy, TAU=treatment as usual, WL=waitlist.

© 2018 Zhou X et al. JAMA Psychiatry.
